# Stereocontrolled Ring-Opening of Oxazolidinone-Fused Aziridines for the Synthesis of 2‑Amino Ethers

**DOI:** 10.1021/acs.joc.5c01068

**Published:** 2025-07-07

**Authors:** Tsung-Lun Tsai, Chin-Hsun Wu, Chih-Ling Lo, Chi-Sheng Wen, Duen-Ren Hou

**Affiliations:** Department of Chemistry, 34911 National Central University, 300 Jhong-Da Rd., Jhong-Li, Taoyuan 320317, Taiwan

## Abstract

We report a stereocontrolled method for synthesizing 2-amino ethers via the acid-catalyzed ring-opening of oxazolidinone-fused aziridines with alcohols. Mono-, di-, and trisubstituted aziridines all participate in this transformation, with the substitution pattern and the class of alcohol significantly influencing the outcome. High diastereoselectivity was observed with primary and secondary alcohols, while tertiary alcohols often led to elimination or epimerization, depending on the aziridine substrate. This methodology was further applied to the synthesis of the neuraminidase inhibitor A-315675. This method offers a broad scope and high diastereoselectivity in many cases, making it a useful strategy for the construction of 2-amino ether frameworks.

## Graphical abstract

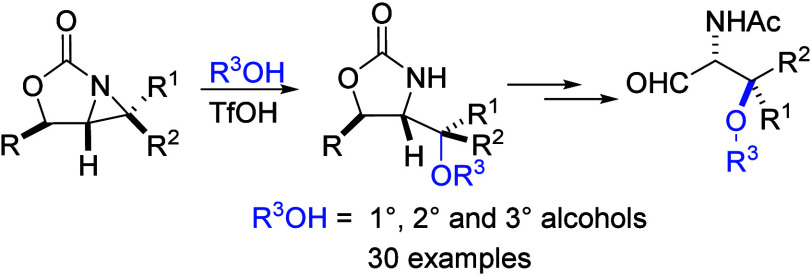

## Introduction

The motif of 2-amino ethers, often derived from corresponding vicinal amino alcohols, is found in various glycosphingolipids and medicines (Figure 1).^1,2^ Since natural, vicinal amino alcohols are convenient chiral building blocks,^3^ it is not surprising that their derivatives, 2-amino ethers, are frequently prepared as chiral chelating agents or catalysts in resolution or enantioselective reactions.^4,5^ The Williamson ether synthesis is a common protocol for preparing 2-amino ethers using vicinal amino alcohols and alkyl halides;^6^ however, the reaction proceeds well only with primary halides or sulfonates.^7^ The ring-opening of aziridines with alcohols represents another straightforward method for preparing 2-amino ethers.^8^ When oxygen nucleophiles were used, Lewis acids were often required to activate aziridines bearing electron-withdrawing groups (EWGs) on the nitrogen, such as *N*-sulfonyl groups. (Scheme 1).^9,10^ In addition to the assistance of EWGs, the ring-opening reactions of 2-oxazolidinone-fused aziridines also benefit from the release of the strain energy. Indeed, this method has been applied to access natural products and biologically active molecules.^11,12^ However, its use to generate 2-amino ethers was rarely utilized and limited to simple alcohols, such as methanol.^11a,13^ In this work, we explore the ring-opening reactions of mono-, di- and trisubstituted 2-oxazolidinone-fused aziridines with primary, secondary and tertiary alcohols to form corresponding 2-amino ethers. The conservative stereochemistry of this reaction enables an efficient, formal synthesis of a potent neuraminidase inhibitor, A-315675,^14^ utilizing one of the resulted 2-amino ether products.

**Figure 1:**
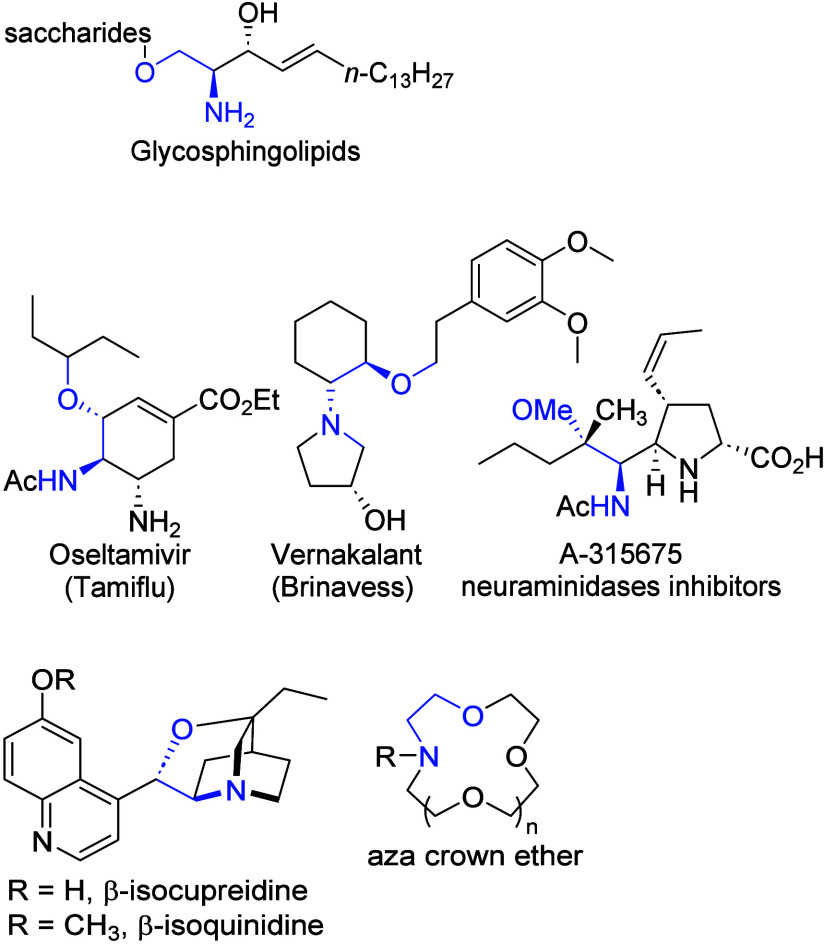
Representative 2-amino ethers.

**Scheme 1:**
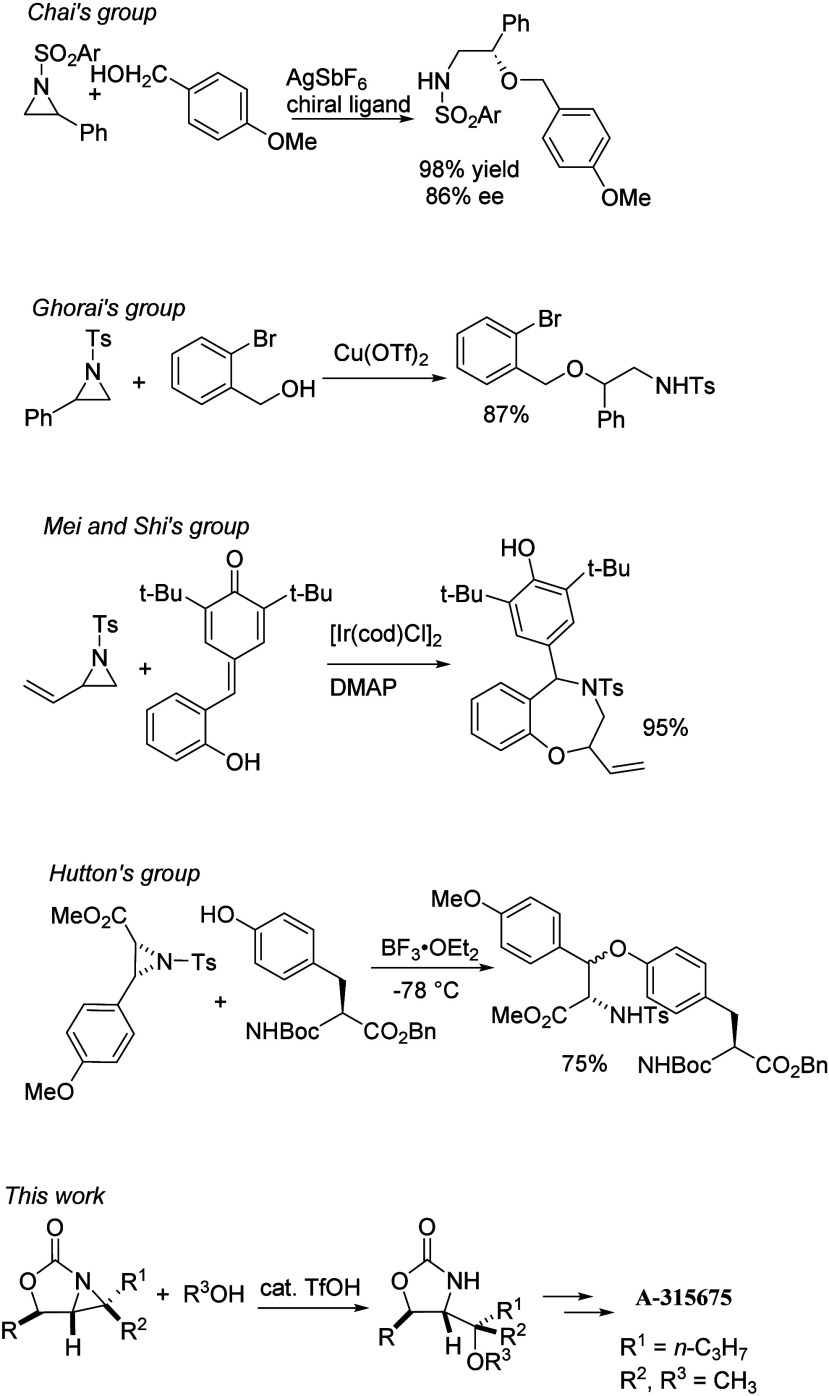
Recent Examples in the Synthesis of 2-Amino Ethers

## Results and Discussion

2-Oxazolidinone-fused aziridines **1** were prepared from phenylacetaldehyde (**2**) in four steps (Scheme 2). The addition of vinyl and 2-methyl-1-propenyl magnesium bromides to **2** gave allylic alcohols **3a** and **3b**, respectively. The two adducts were converted to **1a** and **1b** through the formations of azidoformate **4a** and carbamate **4b** respectively, and the following intramolecular aziridinations.^11a,15,16^ We noticed that the additive, 2,6-di-*tert*-butyl-*p*-cresol (BHT, 1 equiv) increased the yield of **1a** significantly. However, two diastereomeric aziridines **1a** and **1a′** were formed in a 4:1 ratio. Fortunately, aziridines **1a** and **1a′** could be separated by column chromatography, and their stereochemistry were unambiguously deduced from the X-ray crystallography of their ring-opening adducts, *trans*- and *cis*-oxazolidinone **5a** and **5a′**, respectively (Figure 2 and Figure S1, Supporting Information). On the other hand, dimethyl substituted aziridine **1b**, derived from **4b**, was labile and quickly decomposed during purification steps. However, we were able to isolate its ring-opening products **5b** and **5c**, immediately after the formation of **1b** and the treatment of methanol with 20 mol % of trifluoromethanesulfonic acid (*vide infra*). The chloride ion, remaining from the workup procedure used to obtain **4b**, should be responsible for the formation of **5c**. In contrast to the diastereomeric mixture of **5a** and **5a′** produced, both adducts **5b** and **5c** were generated diastereomerically pure, suggesting that the steric hindrance of the dimethyl substituents in substrate **4b** is helpful to the stereoselective formation of the aziridine.^11a^

**Figure 2:**
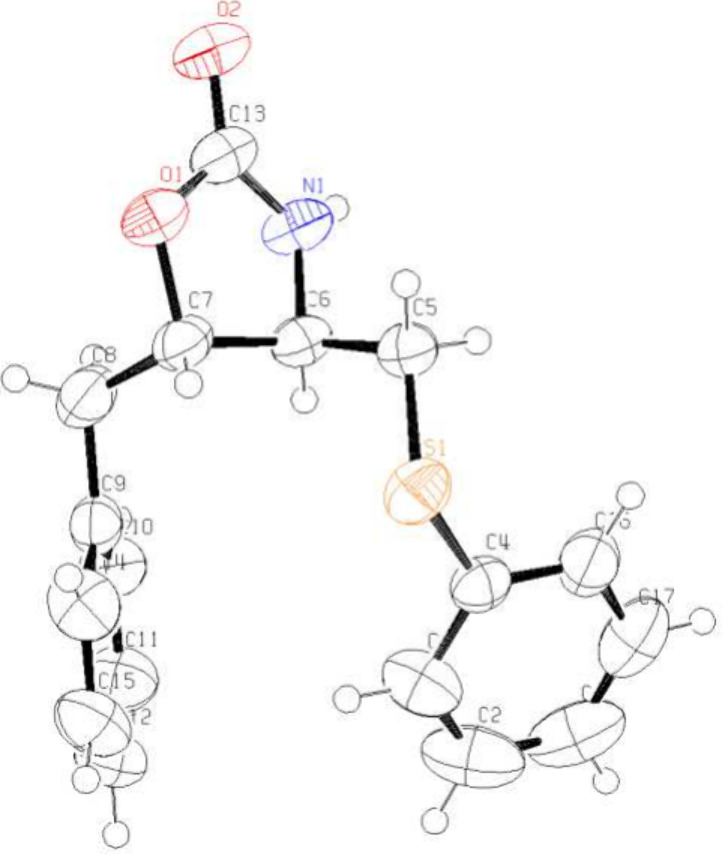
Oak Ridge thermal ellipsoid plot of **5a** with ellipsoids set to 50% probability.

**Scheme 2:**
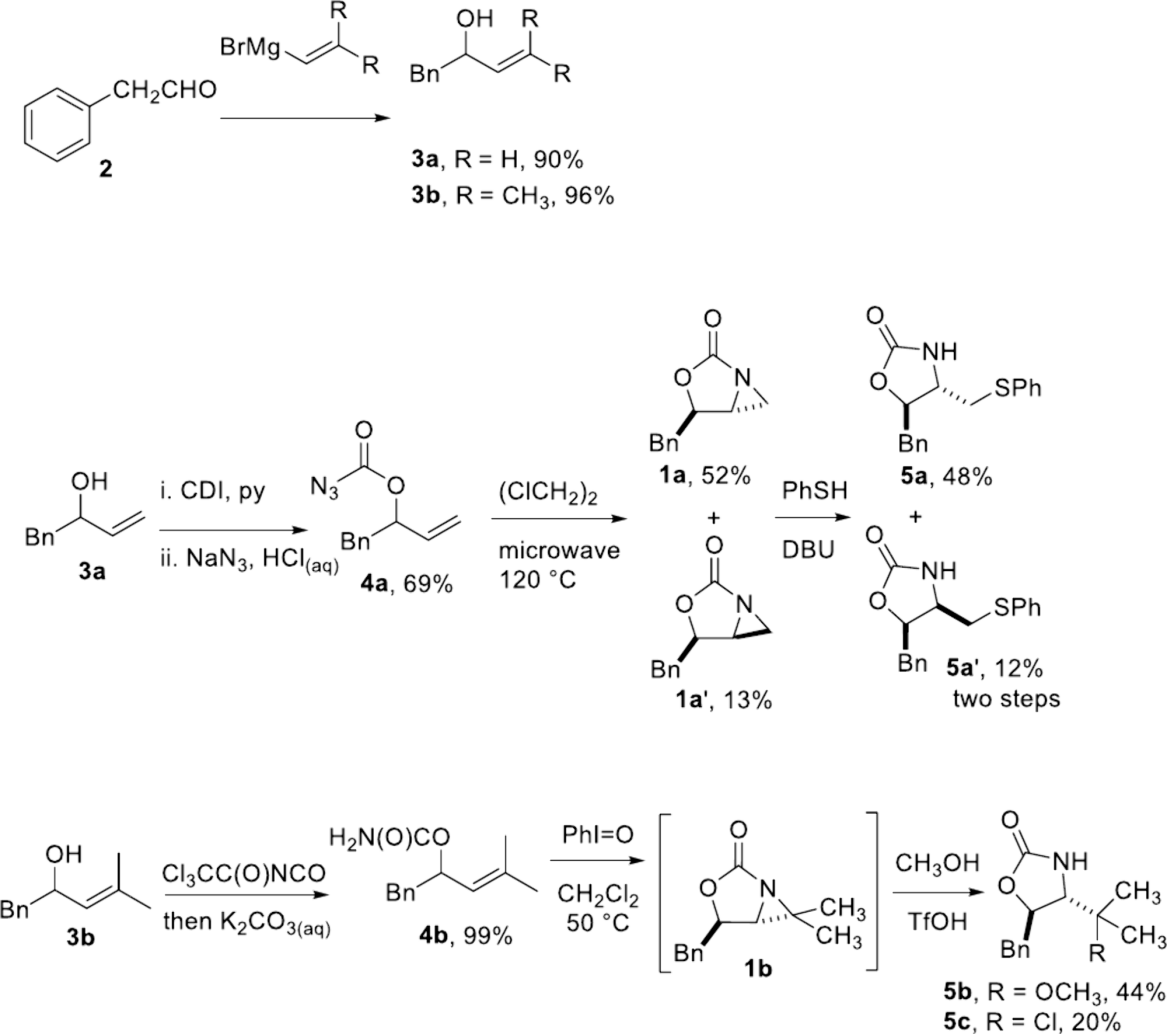
Synthesis of 2-Oxazolidinone-Fused Aziridines **1a** and **1b** and Their Ring-Opening

Aziridine **1c**, prepared from geraniol (**6**), was an alternative compound to replace labile **1b** as a trisubstituted, 2-oxazolidinone-fused aziridine to study the ring-opening reaction (Scheme 3).^12a^ In addition, aziridine **1d**, a 1,2-disubstituted aziridine, was prepared from cinnamyl alcohol (**7**) by the same sequence of reactions. Both **1c** and **1d** were generated as a single diastereomer.

**Scheme 3:**
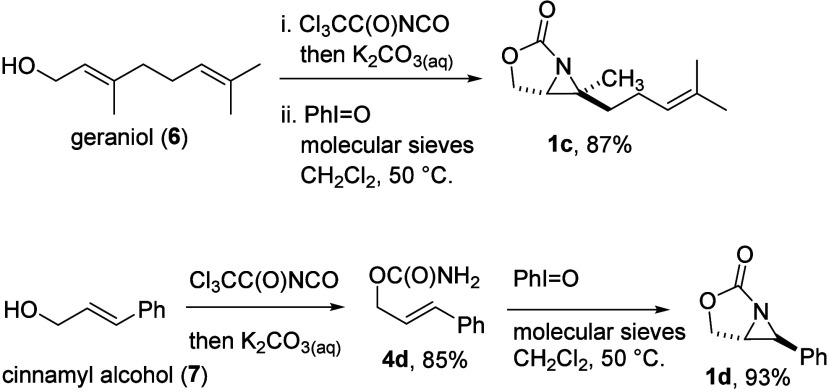
Synthesis of 2-Oxazolidinone-Fused Aziridines **1c** and **1d**

With these *mono-*, *di*- and *tri*substituted aziridines (**1a**, **1d** and **1c**, respectively) in hands, we first screened various acids and bases to promote the ring-opening reaction of **1a** in isopropyl alcohol (Table 1). Under a basic or neutral condition, the desired product **8a** was not formed (entries 1–3). Various metal ions capable of forming complexes with oxazolidinones were screened for their activity in this ring-opening reaction.^17^ We found that the highly stabilized and low nucleophilic triflate anion was helpful to promote this reaction.^18^ Indeed, copper­(II), tin­(II), indium­(III) and scandium­(III) triflates gave moderate to good yields of **8a** (entries 9–12). Applying microwave heating was also helpful in improving the yields and efficiency (entries 9 and 10). However, the ring-opening reactions could be carried out at room temperature, assisted by boron trifluoride diethyl etherate or triflic acid (entries 13–15), and as few as 20 mol % of triflic acid was required (entry 15). Thus, this condition, 20 mol % of TfOH, 25 °C, 1 h, was applied to prepare other 2-amino ethers.

**Table 1:**
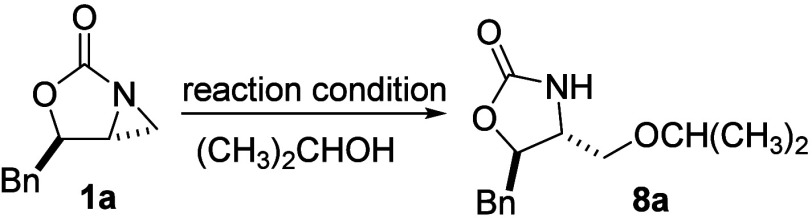
Reaction Conditions for the Ring-Opening of **1a** in Isopropyl Alcohol^a^

entry	reagent/condition	**8a** (%)
1	none	0
2	NaH (1.0 equiv), 25 °C, 1 h	0
3	NEt_3_ (1.0 equiv), 50 °C, 1 h	0
4	Ti[OCH(CH_3_)_2_]_4_ (1.0 equiv), reflux, 16 h	3
5	Al[OCH(CH_3_)_2_]_3_ (1.0 equiv), reflux, 16 h	8
6	Mg(ClO_4_)_2_ (1.0 equiv), 45 °C, 16 h	23
7	Mg(OTf)_2_ (1.0 equiv), 50 °C, 20 min^b^	10
8	Cu(OTf)_2_ (1.0 equiv), 25 °C, 3 h	3
9	Cu(OTf)_2_ (1.0 equiv), 50 °C, 20 min^b^	71
10	Sn(OTf)_2_ (1.0 equiv), 50 °C, 20 min^b^	45
11	In(OTf)_3_ (1.0 equiv), 25 °C, 1 h	56
12	Sc(OTf)_3_ (1.0 equiv), 25 °C, 1 h	65
13	BF_3_ · O(C_2_H_5_)_2_ (1.0 equiv), 25 °C, 1 h	65
14	TfOH (1.0 equiv), 25 °C, 1 h	71
15	TfOH (0.2 equiv), 25 °C, 1 h	75

a A mixture of **1a** (0.10 mmol), the reagent (0.10 mmol), and isopropyl alcohol (0.5 mL) was stirred at the indicated temperature and duration.

b Microwave heating.

In methanol and ethanol, the corresponding ring-opening products **8b** and **8c** were generated with 89 and 85% yield, respectively (entries 1 and 2, Table 2). However, only 50% yield of **8d** was harvested when the standard protocol was applied to 1-butanol (entry 3). The lower solubility of **1a** in 1-butanol and the higher viscosity of 1-butanol, comparing with methanol and ethanol, may account for this low yield.^19^ To address the limitations of using 1-butanol in the dual roles of solvent and reactant, anhydrous dichloromethane was employed as the reaction solvent (0.14 *M* of **1a**), while 1-butanol was used as the nucleophile in 10-fold excess. This modification increased the yield of **8d** to 62% (in parentheses, entry 3). This protocol was also helpful for the reactions with secondary alcohols, such as 3-pentanol and cyclopentanol (entries 4 and 5). Additional results of applying common laboratory solvents for the reaction were summarized in Table S1 (Supporting Information). Interestingly, the reaction went smoothly in neat cyclohexanol to give **8g** (68%, entry 6). The ring-opening reaction with tertiary alcohols, including *tert*-butyl alcohol and 1-adamantanol, were more challenging (entries 7 and 8), and the yields were up to 43% (**8h**) after many attempts. This condition was also applied to prepare 2-amino-ester **8j** (60%), in which the adduct was derived from **1a** and acetic acid (entry 9).

**Table 2:**
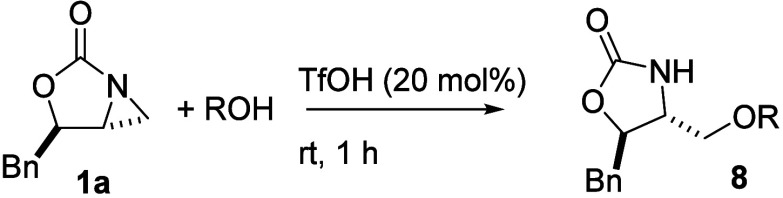

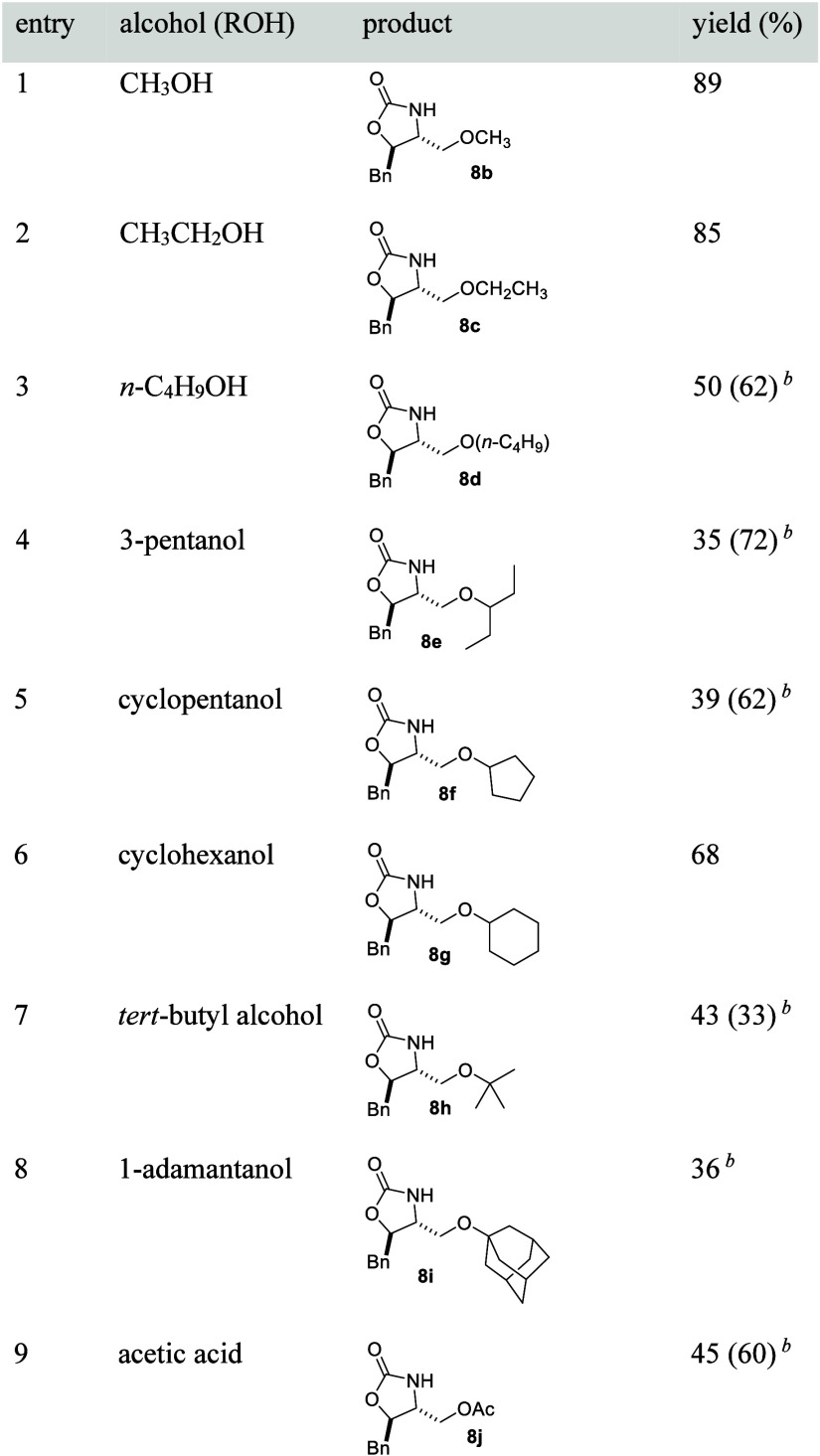
Ring-Opening Reaction of **1a** with 1–3° Alcohols^a^

a A mixture of **1a** (0.06 mmol), TfOH (0.01 mmol), and alcohol (0.5 mL) was stirred at 25 °C for 1 h.

b A mixture of **1a** (0.06 mmol), TfOH (0.01 mmol), alcohol (0.6 mmol), and CH_2_Cl_2_ (0.5 mL) was stirred at 25 °C for 1 h.

The results derived from the ring-opening reaction of *tri*-substituted aziridine **1c** are summarized in Table 3. Good yields of **9a** and **9b** were obtained from the reactions in methanol and ethanol, respectively (entries 1 and 2). The stereochemistry of compound **9a** was confirmed by X-ray crystallographic analysis of its derivative **9aa** (Figure 3), which was obtained *via* hydrolysis of the oxazolidinone moiety and subsequent formation of a benzamide (eq 1). This stereochemical outcome is consistent with a mechanism involving inversion of configuration and an S_N_2-like arrangement of the incoming methanol and the departing carbamate group during the ring-opening of aziridine **1c**, suggesting that both acidic and basic conditions proceed through the same pathway to afford the same stereochemical result.^12a^ As shown in Table 2, a better yield of **9c**, adduct of **1c** and 1-butanol, was derived from the reaction conducted in dichloromethane (entry 3 versus 4). All secondary alcohols gave their corresponding adducts **9d**–**9g** in 70–78% yields (entries 5–8). However, tertiary alcohols, such as *tert*-butyl alcohol and 1-adamantanol, did not undergo the addition but elimination reactions occurred to afford product **10** (entries 9 and 10). The addition reaction between the bulky tertiary alcohols and *tri*-substituted aziridine **1c** was clearly hindered. On the other hand, acetic acid and water, both more acidic than common alcohols, gave the adducts **9h** and **9i** in good yields (entries 11 and 12). All the addition products (**9a–9g**, **9h**, and **9i**) were obtained as diastereomerically pure compounds, as confirmed by their NMR spectra (Supporting Information).

**Figure 3:**
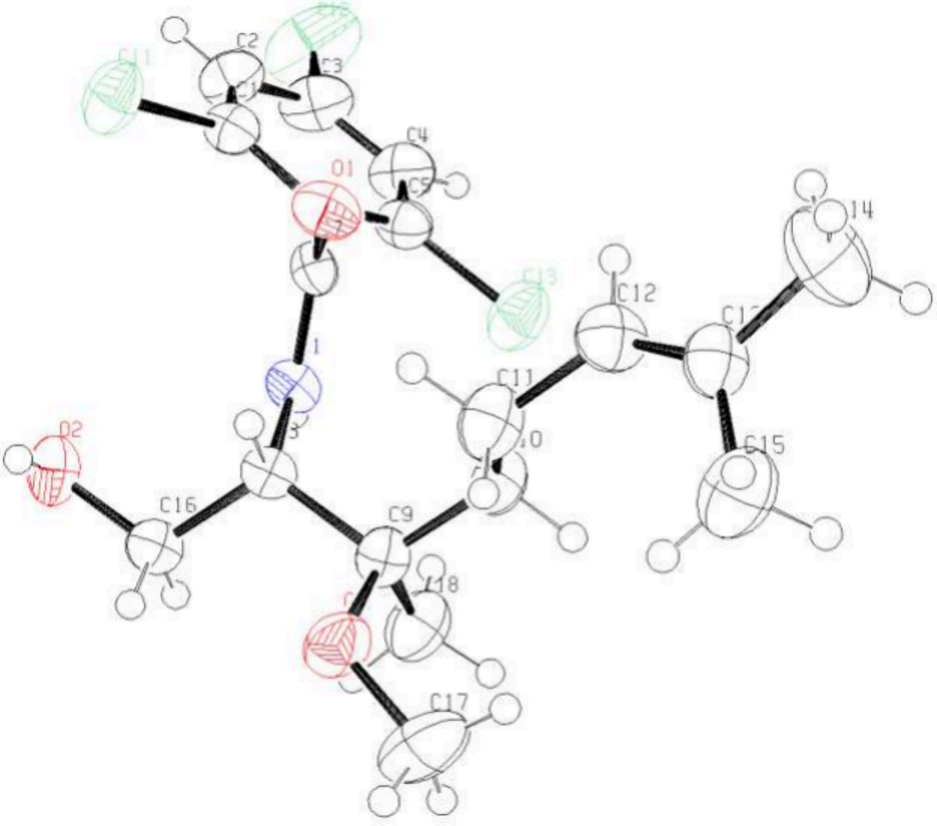
Oak Ridge thermal ellipsoid plot of **9aa** with ellipsoids set to 50% probability.

**Table 3:**
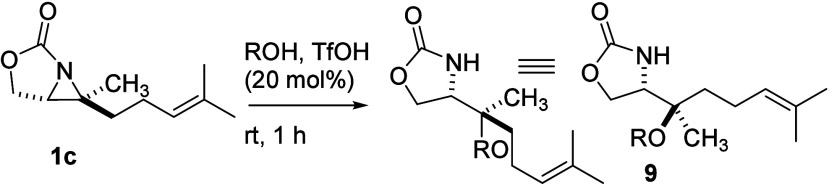
Ring-Opening Reaction of **1c** with 1–3° Alcohols^a^

entry	alcohol (ROH)	product	**9** (%)​
1	CH_3_OH	**9a**, R = CH_3_	87 ​(85)^b^
2	CH_3_CH_2_OH	**9b**, R = CH_2_CH_3_	85​
3	*n*-C_4_H_9_OH	**9c**, R = *n*-C_4_H_9_	51​
4^*b*^	*n*-C_4_H_9_OH	**9c**, R = *n*-C_4_H_9_	65​^c^
5	isopropyl alcohol	**9d**, R = *i*-C_3_H_7_	78​
6	3-pentanol	**9e**, R = 3-pentyl	75​^c^
7	cyclopentanol	**9f**, R = cyclopentyl	70 ^*c​*^
8	cyclohexanol	**9g**, R = cyclohexyl	75​
9	*tert*-butyl alcohol	**10**	75​
10	1-adamantanol	**10**	45​
11	acetic acid	**9h**, R = acetyl	69​^c^
12	water	**9i**, R = H	81​^c^

a A mixture of **1c** (0.20 mmol), TfOH (0.04 mmol), and alcohol (1.0 mL) was stirred at 25 °C for 1 h.

b The reaction was conducted with 1 mmol **1c**.

c A mixture of **1c** (0.21 mmol), TfOH (0.04 mmol), alcohol (2.1 mmol), and CH_2_Cl_2_ (0.5 mL) was stirred at 25 °C for 1 h.

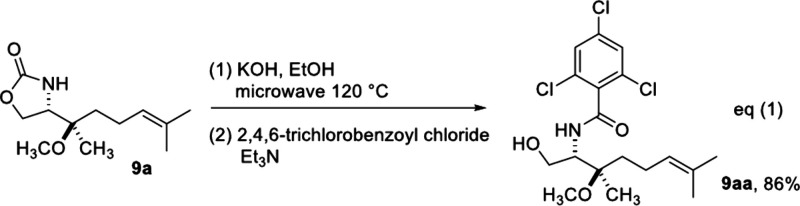

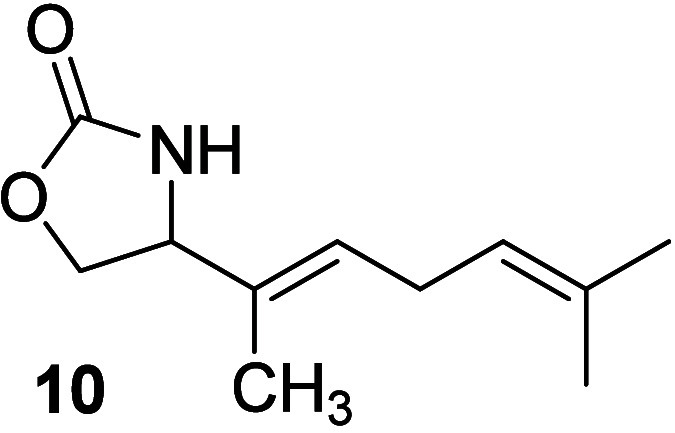





*Di*-substituted aziridine **1d** has a short shelf life and is prone to decomposition during purification by column chromatography. Consequently, the ring-opening reactions of **1d** were conducted immediately after its formation, without further purification. The yields of the two-step reactions to form adducts **11** are summarized in Table 4. Primary alcohols, including methanol, ethanol and 1-butanol, gave the products **11a**–**c**, respectively with 92–71% yields (entries 1–3). Secondary alcohols produced the adducts **11d**–**g** in a range of 72–46% yields (entries 4–7) with excellent diastereomeric purity on the ^1^H and ^13^C NMR spectra. The X-ray crystallography of **11d** was obtained (Figure 4) and the expected *anti*-relationship between the oxazolidinone moiety and the isopropoxy group was also observed. However, this stereochemical conservativeness was lost for the ring-opening reactions with 3° alcohols (entries 8 and 9). Although *tert*-butyl alcohol and 1-adamantanol gave the corresponding adducts **11h** and **11i** in 36 and 30% yields, respectively, two diastereomers were observed in NMR spectra of their crude products (dr ∼ 2:1). Indeed, the major diastereomer of **11i** was later confirmed as *syn,* by X-ray crystallography (Figure 5). This result indicated that epimerization occurred at the benzylic carbon due to the long lifetime of benzyl carbocation^20^ and sterically hindered alkoxy groups.

**Figure 4:**
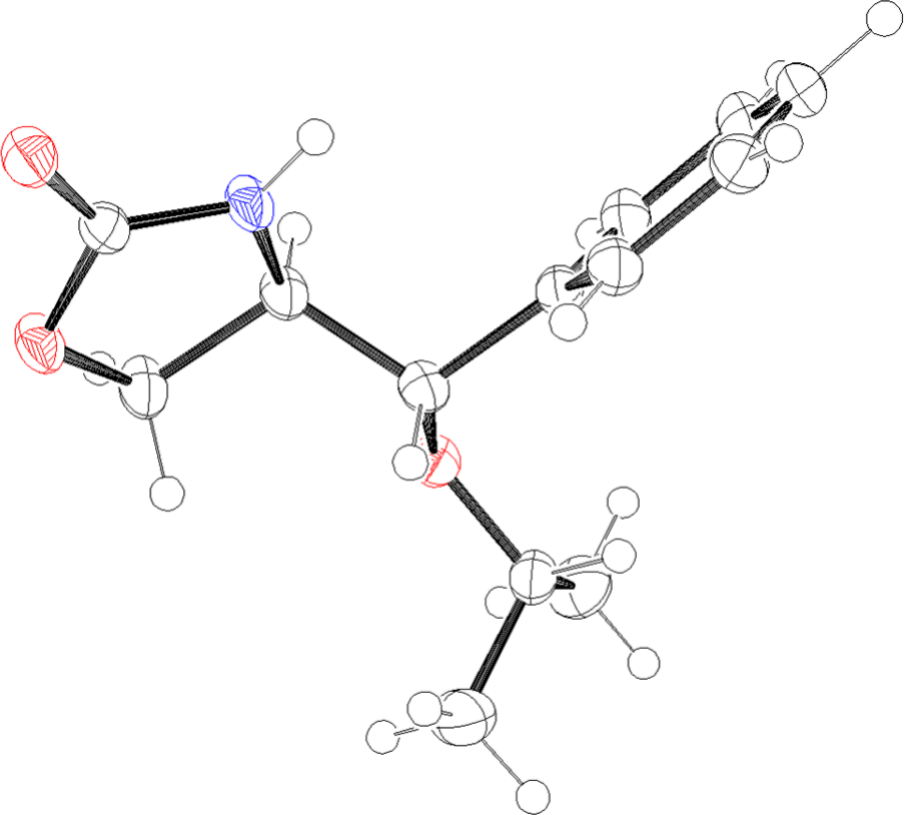
Oak Ridge thermal ellipsoid plot of **11d** with ellipsoids set to 50% probability.

**Figure 5:**
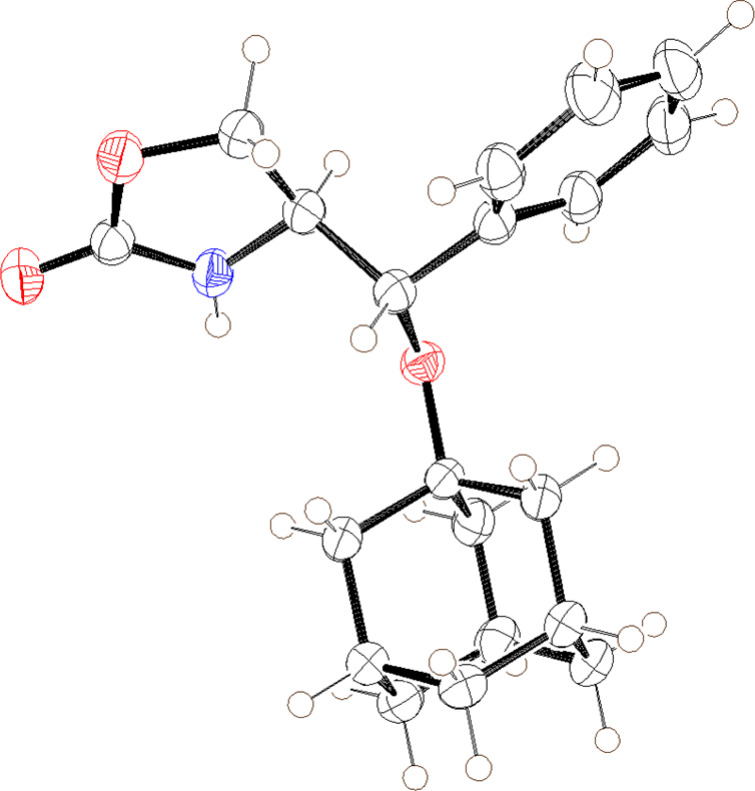
Oak Ridge thermal ellipsoid plot of **11i** with ellipsoids set to 50% probability.

**Table 4:**
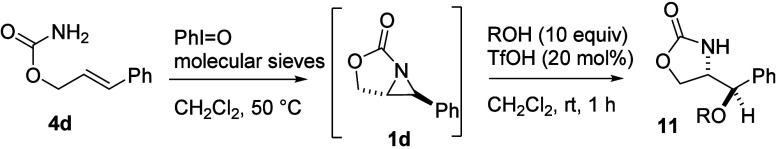
Ring-Opening Reaction of **1d** with 1–3° Alcohols^a^

entry	alcohol (ROH)	product	**11** (%)
1	CH_3_OH	**11a**, R = CH_3_	82
2	CH_3_CH_2_OH	**11b**, R = CH_2_CH_3_	70
3	*n*-C_4_H_9_OH	**11c**, R = *n*-C_4_H_9_	71
4^*b*^	isopropyl alcohol	**11d**, R = *i*-C_3_H_7_	61
5	3-pentanol	**11e**, R = 3-pentyl	46
6	cyclopentanol	**11f**, R = cyclopentyl	72
7	cyclohexanol	**11g**, R = cyclohexyl	63
8	*tert*-butyl alcohol	**11h**	36
9	1-adamantanol	**11i**	30

a A mixture of freshly prepared **1d** (0.11 mmol), TfOH (0.02 mmol), alcohol (1.1 mmol), and CH_2_Cl_2_(0.6 mL) was stirred at 25 °C for 1 h.

This aziridine ring-opening reaction was employed to synthesize a key intermediate in the preparation of A-315675 (Scheme 4). Starting from D-mannitol, 1,3-dioxolane-4-carbaldehyde **12** was obtained in two steps.^21^ A subsequent Wittig reaction yielded alkene **13** (*Z*/*E* = 5:4), which was then hydrolyzed, protected with a TBS group, and converted to aziridine **17** following the sequence outlined in Schemes 2 and 3. Acid-catalyzed ring-opening of **17** in methanol afforded oxazolidinone ether **18**, which subsequently underwent oxazolidinone hydrolysis, acetamide formation, and oxidative cleavage of the vicinal diol to yield precursor **20**, previously synthesized in 11 steps starting from d-serine.^22^ This chiral pool synthesis, based on D-mannitol, facilitated further modification of the synthesized amino ethers due to its dihydroxylated carbon skeleton, as exemplified by the transformation of compound **19** into aldehyde **20**. Although the *E*/*Z* isomers of **13** and their derived diastereomers could not be separated in our hands, the NMR spectra of the major isomer in **20** matched those reported in the literature.^22,23^ Importantly, this synthetic route offers an alternative approach to preparing **20**, highlighting the utility of aziridine ring-opening in accessing chiral 2-amino ethers.

**Scheme 4:**
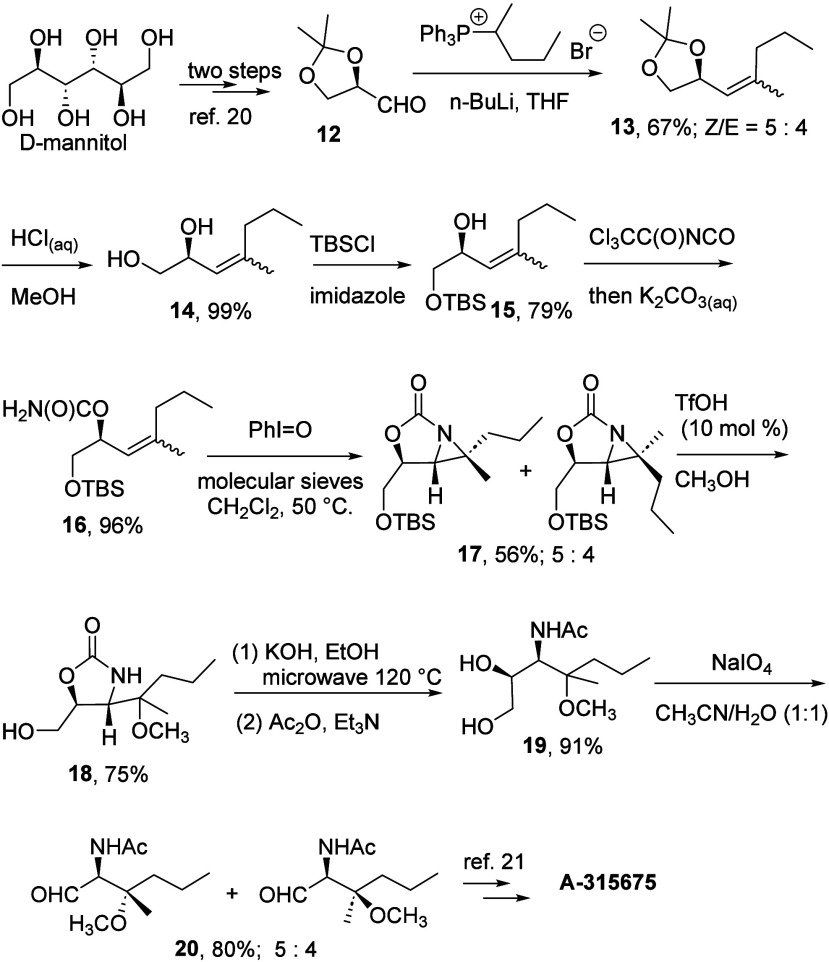
Formal Synthesis of A-315675

## Conclusion

In summary, we have demonstrated that the *acid*-catalyzed, ring-opening of oxazolidinone-fused aziridines with alcohols is an effective strategy for synthesizing 2-amino ethers. While *mono*-, *di*-, and *tri*-substituted aziridines are all reactive in this transformation, the nature of the alcohol significantly influences the outcome. For *mono*-substituted aziridines, primary, secondary, and tertiary alcohols consistently yielded the corresponding 2-amino ethers (Table 2). *Tri*-substituted aziridines reacted smoothly with primary and secondary alcohols to afford the desired ethers with excellent diastereoselectivity, whereas tertiary alcohols led predominantly to elimination products (Table 3). In the case of *di*-substituted aziridines, all three classes of alcohols participated in the addition reaction, but high diastereoselectivity was observed only with primary and secondary alcohols. Reactions with tertiary alcohols resulted in epimerization (Table 4). X-ray crystallography and NMR analysis provided clear resolution of the stereochemical outcomes for all three types of aziridine ring-opening reactions. This methodology was successfully applied to the formal synthesis of the neuraminidase inhibitor A-315675. We believe this stereocontrolled approach to accessing 2-amino ethers holds strong potential for future applications in synthetic chemistry.

## Experimental Section

### General Information

Reagents, such as vinylmagnesium bromide solution (1.0 *M* in THF), 2-methyl-1-propenylmagnesium bromide solution (0.5 *M* in THF), alcohols and 2-bromopentane, were purchased from commercial sources (ACS grade) and used without further purification. Thin-layer chromatography (TLC) was conducted using precoated silica gel 60 F_254_ plates containing a fluorescent indicator; spots were examined under UV light or revealed by KMnO4 solution; purification by chromatography was conducted using silica gel (230–400 mesh). All microwave irradiation experiments were conducted in a CEM Discover microwave reactor, equipped with an IR temperature sensor and in a sealed, borosilicate MW vessels. The ^1^H and ^13^C {^1^H} NMR spectra were recorded in a CDCl_3_ solution using a Bruker Ascend 600 NMR or Bruker Avance 500, 300 NMR spectrometers. Chemical shifts for ^1^H NMR and ^13^C {^1^H} NMR spectra are reported in δ units (parts per million) with reference to residual solvent peaks. Structural assignments were made with additional information from gCOSY, gHSQC, and gHMBC experiments. High-resolution mass spectrometry (HRMS) data were recorded on a JMS-700 quadrupole mass spectrometer. The data for single X-ray crystallography were recorded on a Bruker D8 QUEST CCD diffractometer with Mo K*a* radiation (*l* = 0.71073 Å), and the structures were solved with APEX3 program. Aziridine **1c** was prepared according to literature procedure.^12a^

### Safety Statement

Caution! Triflic acid is a highly corrosive acid and must be handled with extreme care, using a well-ventilated fume hood and appropriate personal protective equipment. The synthesis of compound **4a** involves the *in situ* generation of hydrazoic acid from sodium azide and hydrochloric acid. Hydrazoic acid is acutely toxic and highly explosive.^24^ To minimize risk, the reaction should be performed under a dilute condition with continuous nitrogen purging of the headspace to prevent condensation of hydrazoic acid. Additionally, the entire setup must be operated behind a protective blast shield.

#### 1-Phenylbut-3-en-2-ol (**3a**)

A solution of phenylacetaldehyde (1.0 g, 8.32 mmol) in anhydrous THF (8 mL) was added to a solution of vinylmagnesium bromide (1.0 *M* in THF, 12.5 mL, 12.5 mmol) at −30 °C. The reaction mixture was stirred at −30 °C for 30 min, another 2 h at room temperature, neutralized with hydrochloric acid (2 *N*, 6 mL), diluted with water (30 mL) and extracted with ethyl acetate (30 mL, 20 mL × 2). The combined organic layers were dried over sodium sulfate, filtered and concentrated. The crude product was purified by column chromatography (SiO_2_, EtOAc/hexanes, 1:3, *R*_*f*_ 0.43) to give compound **3a** (1.10 g, 7.43 mmol, 90%) as a colorless liquid. ^1^H NMR (300 MHz, CDCl_3_) δ 7.38–7.26 (m, 5H), 6.0–5.90 (m, 1H), 5.29 (d, *J* = 17.2 Hz, 1H), 5.17 (d, *J* = 10.3 Hz, 1H), 4.38–4.32 (m, 1H), 2.88–2.85 (m, 2H), 2.51 (s, 1H); ^13^C­{^1^H} NMR (75 MHz, CDCl_3_) δ 140.0, 137.7, 129.5, 128.2, 126.2, 114.6, 73.4, 43.6. The spectroscopic data were consistent with the reported values.^25^

#### 4-Methyl-1-phenylpent-3-en-2-ol (**3b**)

A solution of phenylacetaldehyde (0.50 g, 4.16 mmol) in anhydrous THF (4 mL) was added to a solution of 2-methyl-1-propenylmagnesium bromide (0.5 *M* in THF, 10 mL, 5.0 mmol) at −30 °C. The reaction mixture was stirred at −30 °C for 30 min, another 2 h at room temperature, neutralized with hydrochloric acid (2 *N*, 6 mL), diluted with water (20 mL) and extracted with ethyl acetate (20 mL, 15 mL × 2). The combined organic layers were dried over sodium sulfate, filtered and concentrated. The crude product was purified by column chromatography (SiO_2_, EtOAc/hexanes, 1:3, *R*_*f*_ 0.45) to give compound **3b** (0.70 g, 3.97 mmol, 96%) as a colorless liquid. ^1^H NMR (500 MHz, CDCl_3_) δ 7.34–7.25 (m, 5H), 5.28–5.26 (m, 1H), 4.60–4.56 (m, 1H), 2.86–2.78 (m, 2H), 1.75 (s, 3H), 1.60 (s, 3H); ^13^C­{^1^H} NMR (126 MHz, CDCl_3_) δ 138.1, 135.2, 129.5, 128.3, 127.0, 126.2, 69.6, 44.1, 25.6, 18.0; HRMS (ESI) calcd for [M + H]^+^ (C_12_H_17_O) 177.1274, found 177.1263.

#### 1-Phenylbut-3-en-2-yl carbonazidate (**4a**)

1,1′-Carbonyldiimidazole (CDI, 820.6 mg, 5.06 mmol) was added to a solution of **3a** (300.0 mg, 2.02 mmol), pyridine (320.2 mg, 4.05 mmol) and benzene (20 mL) at 0 °C. The reaction mixture was warmed up to room temperature, stirred for another16 h, added with water (20 mL) and extracted with ethyl acetate (10 mL × 2). The combined organic layers were washed with saturated NaCl_(aq)_ (10 mL), dried over sodium sulfate, filtered and concentrated. The crude product was purified by column chromatography (SiO_2_, EtOAc/hexanes, 1:3, *R*_*f*_ 0.28) to give 1-phenylbut-3-en-2-yl 1*H*-imidazole-1-carboxylate (462.2 mg, 1.90 mmol, 94%) as a colorless liquid. ^1^H NMR (500 MHz, CDCl_3_) δ 8.09 (s, 1H), 7.38 (s, 1H), 7.31–7.21 (m, 5H), 7.05 (s, 1H), 5.94–5.87 (m, 1H), 5.61 (q, *J* = 7.0 Hz, 1H), 5.37–5.29 (m, 2H), 3.14–3.05 (m, 2H); ^13^C­{^1^H} NMR (126 MHz, CDCl_3_) δ 147.8, 137.0, 135.6, 134.1, 130.6, 129.4, 128.5, 127.0, 119.1, 117.0, 79.8, 40.7; HRMS (ESI) calcd for [M + H]^+^ (C_14_H_15_N_2_O_2_) 243.1128, found 243.1120. The above intermediate (462.2 mg, 1.90 mmol) was dissolved in DMF (9.0 mL), added with sodium azide (620.1 mg, 9.54 mmol) and concentrated HCl_(aq)_ (12 *N*, 1.4 mL, 16.8 mmol) at 0 °C. The reaction mixture was stirred at room temperature for 3 h, diluted with water (30 mL) and extracted with ethyl acetate (30 mL, 10 mL × 2). The combined organic layers were washed with saturated NaCl_(aq)_ (30 mL), dried over sodium sulfate, filtered and concentrated. The crude product was purified by column chromatography (SiO_2_, EtOAc/hexanes, 1:3, *R*_*f*_ 0.76) to give **4a** (300.9 mg, 1.39 mmol, 69%; two steps) as a colorless liquid. ^1^H NMR (500 MHz, CDCl_3_) δ 7.37–7.26 (m, 5H), 5.92–5.85 (m, 1H), 5.49–5.46 (m, 1H), 5.35 (dd, *J* = 17.0 Hz, *J* = 1.0 Hz, 1H), 5.28 (dd, *J* = 10.5 Hz, *J* = 1.0 Hz, 1H), 3.08 (dd, *J* = 13.5 Hz, *J* = 7.5 Hz, 1H), 2.99 (dd, *J* = 13.5 Hz, *J* = 6.0 Hz, 1H); ^13^C­{^1^H} NMR (126 MHz, CDCl_3_) δ 156.5, 135.9, 134.3, 129.3, 128.3, 126.7, 118.3, 79.8, 40.5; HRMS (ESI) calcd for [M + Na]^+^ (C_11_H_11_N_3_NaO_2_) 240.0743, found 240.0738.

#### (4R*,5R*)-4-Benzyl-3-oxa-1-azabicyclo­[3.1.0]­hexan-2-one (**1a**)

A borosilicate microwave vessel (10 mL) containing a solution of **4a** (34.9 mg, 0.16 mmol), 2,6-di-*tert*-butyl-4-methylphenol (BHT, 35.4 mg, 0.16 mmol) and 1,2-dichloroethane (7 mL) was sealed with a silicon rubber septum, placed in the microwave reactor, and stirred at 120 °C (monitored by the equipped IR temperature sensor) for 30 min at 300 W. After cooling to rt, the reaction mixture was concentrated and the crude product was purified by column chromatography (SiO_2_, EtOAc/hexanes, 1:1, *R*_*f*_ 0.55) to give **1a** (15.9 mg, 0.084 mmol, 52%) as a colorless liquid. ^1^H NMR (500 MHz, CDCl_3_) δ 7.37–7.25 (m, 5H), 4.81 (t, *J* = 6.5 Hz, 1H), 3.22–3.18 (m, 1H), 3.04–3.01 (m, 1H), 3.0–2.98 (m, 1H), 2.49 (d, *J* = 5.0 Hz, 1H), 2.15 (d, *J* = 4.0 Hz, 1H); ^13^C­{^1^H} NMR (126 MHz, CDCl_3_) δ 166.6, 134.2, 129.5, 128.9, 127.4, 78.6, 41.1, 41.0, 35.3; HRMS (ESI) calcd for [M + H]^+^ (C_11_H_12_NO_2_) 190.0862, found 190.0864. (4R*,5S*)-4-Benzyl-3-oxa-1-azabicyclo­[3.1.0]­hexan-2-one (**1a′**) was also isolated (4.0 mg, 0.021 mmol, 13%) as a colorless liquid during column chromatography (*R*_*f*_ 0.56). ^1^H NMR (500 MHz, CDCl_3_) δ 7.36–7.23 (m, 5H), 4.93 (dd, *J* = 11.8, 6.8 Hz, 1H), 3.17 (dd, *J* = 14.0, 6.4 Hz, 1H), 3.03 (dd, *J* = 9.2, 4.7 Hz, 1H), 2.92 (dd, *J* = 13.9, 7.5 Hz, 1H), 2.49 (dd, *J* = 4.9, 0.7 Hz, 1H), 2.27 (d, *J* = 4.5 Hz, 1H); ^13^C­{^1^H} NMR (126 MHz, CDCl_3_) 134.8, 129.0, 128.9, 127.4, 77.9, 40.7, 38.5, 32.8.

#### (4S*,5R*)-5-Benzyl-4-((phenylthio)­methyl)­oxazolidin-2-one (**5a**)

A borosilicate microwave vessel (10 mL) containing a solution of **4a** (44.7 mg, 0.21 mmol), 2,6-di-*tert*-butyl-4-methylphenol (BHT, 46.2 mg, 0.21 mmol) and 1,2-dichloroethane (7 mL) was sealed with a silicon rubber septum, placed in the microwave reactor, and stirred at 120 °C (monitored by the equipped IR temperature sensor) for 30 min at 300 W. After cooling to rt, the reaction mixture was concentrated and the crude product was purified by column chromatography (SiO_2_, EtOAc/hexanes, 1:1) to give a mixture of **1a** and **1a′** (25.3 mg, 0.13 mmol, 65%). Thiophenol (48.0 μL, 51.8 mg, 0.47 mmol) was added to a solution of the above mixture, THF (1.0 mL) and 1,8-diazabicyclo[5.4.0]­undec-7-ene (DBU, 6.6 μL, 71.6 mg, 0.47 mmol) at 0 °C. The reaction mixture was stirred at rt for 1 h and concentrated. The crude product was purified by column chromatography (SiO_2_, EtOAc/hexanes, 1:1) to give **5a** (19.2 mg, 0.06 mmol, 48%, *R*_*f*_ 0.43) as a white solid. ^1^H NMR (500 MHz, CDCl_3_) δ 7.31–7.25 (m, 5H), 7.19–7.11 (m, 5H), 5.63 (s, 1H), 4.54 (q, *J* = 5.7 Hz, 1H), 3.19–3.15 (q, *J* = 5.7 Hz, 1H), 3.11 (dd, *J* = 13.9, 6.0 Hz, 1H), 2.89 (dd, *J* = 13.9, 7.3 Hz, 1H), 2.84 (dd, *J* = 14.0, 7.8 Hz, 1H), 2.76 (dd, *J* = 13.8, 5.6 Hz, 1H); ^13^C­{^1^H} NMR (126 MHz, CDCl_3_) δ 188.3, 134.5, 129.4, 128.9, 127.4, 79.6, 55.9, 54.1, 40.4; HRMS (ESI) calcd for [M + H]^+^ (C_17_H_18_NO_2_S) 300.1053, found 300.1043. (4R*,5R*)-5-Benzyl-4-((phenylthio)­methyl)­oxazolidin-2-one (**5a′**, 4.8 mg, 0.066 mmol, 12%, *R*_*f*_ 0.50) was also ioslated. ^1^H NMR (500 MHz, CDCl_3_) δ 7.34–7.24 (m, 5H), 5.59 (br, 1H), 5.63 (s, 1H), 4.92–4.88 (m, 1H), 3.84–3.80 (m, 1H), 3.22 (dd, *J* = 13.6, 3.3 Hz, 1H), 2.89 (dd, *J* = 13.9, 7.3, 1H), 3.17 (dd, *J* = 14.5, 8.9 Hz, 1H), 2.95–2.91 (m, 2H). Single crystals of **5a** and **5a′** suitable for X-ray analysis were grown by gas diffusion between hexanes and a dichloromethane solution at 25 °C.

#### 4-Methyl-1-phenylpent-3-en-2-yl Carbamate (**4b**)

Trichloroacetyl isocyanate (94.0 μL, 148.8 mg, 0.74 mmol) was added to a solution of **3b** (106.9 mg, 0.61 mmol) and dichloromethane (2.9 mL) at rt. The reaction mixture was stirred at rt for 16 h, added with potassium carbonate (94.0 mg, 0.68 mmol), methanol (0.68 mL), water (0.68 mL), stirred for another 3 h and concentrated. The residue was diluted with water (5 mL), extracted with dichloromethane (5 mL × 3), and the combined organic layers were washed with saturated NaCl_(aq)_ (10 mL), dried over sodium sulfate, filtered and concentrated to give **4b** (133.0 mg, 0.61 mmol, 99%) as a colorless liquid. ^1^H NMR (500 MHz, CDCl_3_) δ 7.28–7.19 (m, 5H), 5.57 (q, *J* = 6.7 Hz, 1H), 5.15 (d, *J* = 9.0 Hz, 1H), 4.78 (br, 2H), 2.99 (dd, *J* = 13.5, 6.0 Hz, 1H), 2.8 (dd, *J* = 13.5, 7.0 Hz, 1H), 1.69 (s, 3H), 1.53 (s, 3H); ^13^C­{^1^H} NMR (126 MHz, CDCl_3_) δ 156.6, 137.4, 137.2, 129.6, 128.1, 126.3, 72.8, 41.4, 25.6, 18.2; HRMS (ESI) calcd for [M + H]^+^ (C_13_H_18_NO_2_) 220.1332, found 220.1327.

#### (4S*,5R*)-5-Benzyl-4-(2-methoxypropan-2-yl)­oxazolidin-2-one (**5b**)

Iodosobenzene (46.0 mg, 0.21 mmol) was added to a solution of **4b** (22.9 mg, 0.10 mmol), molecular sieves (3 Å, 86 mg) and 1,2-dichloroethane (1.0 mL). The reaction mixture was stirred at 50 °C (oil bath) for 2 h, filtered with a pad of Celite and concentrated to give crude **1b** (22.4 mg). The solution of the crude **1b** in methanol was added with trifluoromethanesulfonic acid (1.8 μL, 3.06 mg, 0.02 mmol) at 0 °C, stirred for 1 h and concentrated. The crude product was purified by column chromatography (SiO_2_, EtOAc/hexanes, 1:1, *R*_*f*_ 0.13) to give **5b** (11.3 mg, 0.05 mmol, 44%) as a colorless liquid. ^1^H NMR (500 MHz, CDCl_3_) δ 7.33–7.25 (m, 5H), 5.95 (br, 1H), 4.58–4.55 (m, 1H), 3.42–3.41 (m, 1H), 3.12 (s, 3H), 3.07 (dd, *J* = 14.0, 6.5 Hz, 1H), 2.93 (dd, *J* = 14.0, 6.0 Hz, 1H), 1.04 (s, 3H), 0.91 (s, 3H); ^13^C­{^1^H} NMR (126 MHz, CDCl_3_) δ 159.0, 135.4, 129.7, 128.6, 127.1, 78.4, 75.7, 63.8, 49.3, 41.6, 19.2; HRMS (ESI) calcd for [M + H]^+^ (C_14_H_20_NO_3_) 250.1438, found 250.1434. (4S*,5R*)-5-Benzyl-4-(2-chloropropan-2-yl)­oxazolidin-2-one (**5c**) was also isolated (5.1 mg, 0.021 mmol, 20%) as a viscous liquid during column chromatography (*R*_*f*_ 0.60). ^1^H NMR (500 MHz, CDCl_3_) δ 7.35–7.25 (m, 5H), 6.67 (br, 1H), 4.67–4.65 (m, 1H), 3.61–3.60 (m, 1H), 3.09 (dd, *J* = 14.0, 6.0 Hz, 1H), 2.98 (dd, *J* = 14.0, 6.0 Hz, 1H), 1.39 (d, *J* = 10.5 Hz, 6H); ^13^C­{^1^H} NMR (126 MHz, CDCl_3_) δ 158.8, 134.8, 129.7, 129.6, 128.8, 127.3, 79.0, 69.6, 65.6, 41.5, 27.7, 26.8; HRMS (ESI) calcd for [M + H]^+^ (C_13_H_17_ClNO_2_) 254.0941, found 254.0942.

#### Cinnamyl Carbamate (**4d**)

Trichloroacetyl isocyanate (58.2 μL, 75.7 mg, 0.49 mmol) was added to a solution of cinnamyl alcohol **7** (50.4 mg, 0.38 mmol) and dichloromethane (1.4 mL) at rt. The reaction mixture was stirred at rt for 16 h, added with potassium carbonate (63.6 mg, 0.46 mmol), methanol (0.46 mL), water (0.3 mL), stirred for another 3 h and concentrated. The residue was diluted with water (5 mL), extracted with dichloromethane (5 mL × 3), and the combined organic layers were washed with saturated NaCl_(aq)_ (10 mL), dried over sodium sulfate, filtered and concentrated to give carbamate (56.6 mg, 0.32 mmol, 85%). ^1^H NMR (300 MHz, CDCl_3_) δ 7.40–7.23 (m, 5H), 6.66 (d, *J* = 15.0 Hz, 1H), 6.34–6.24 (m, 1H), 4.85 (brs, 2H), 4.72 (t, *J* = 3.0 Hz, 2H); ^13^C­{^1^H} (75 MHz, CDCl_3_) δ 156.8, 136.2, 133.8, 128.6, 128.0, 126.6, 123.5, 65.6. The spectroscopic data were consistent with the reported values.^26^

#### (5R*,6S*)-6-Phenyl-3-oxa-1-azabicyclo­[3.1.0]­hexan-2-one (**1d**)

Iodosobenzene (124.1 mg, 0.56 mmol) was added to a suspension of **4d** (50.0 mg, 0.28 mmol) and molecular sieves (3 Å, 250 mg) in dichloromethane (1.4 mL). The reaction mixture was stirred at 50 °C (oil bath) for 3 h, filtered with a pad of Celite and concentrated. The crude product was purified by column chromatography (SiO_2_, EtOAc/hexanes, 1:2, *R*_*f*_ 0.38) to give **1d** (45.8 mg, 0.26 mmol, 93%) as a white solid. Mp 100.0–105.0 °C; ^1^H NMR (300 MHz, CDCl_3_) δ 7.36–7.32 (m, 3H), 7.30–7.26 (m, 2H), 4.64 (dd, *J* = 9.5 Hz, *J* = 3.0 Hz,1H), 4.54 (dd, *J* = 9.5 Hz, *J* = 6.0 Hz, 1H), 3.37 (d, *J* = 3.0 Hz, 1H), 3.25–3.22 (m, 1H); ^13^C­{^1^H} (126 MHz, CDCl_3_) δ 166.6, 134.4, 128.7 (2C), 126.3, 67.1, 48.3, 46.3. The spectroscopic data were consistent with the reported values.^27^

#### (4R*,5R*)-5-Benzyl-4-(isopropoxymethyl)­oxazolidin-2-one (**8a**)

Trifluoromethanesulfonic acid (1.3 μL, 2.3 mg, 0.015 mmol) was added to a solution of **1a** (14.2 mg, 0.08 mmol) and isopropyl alcohol (0.5 mL) at 0 °C. The reaction mixture was stirred at rt for 1 h and concentrated. The crude product was purified by column chromatography (SiO_2_, EtOAc/hexanes, 1:1, *R*_*f*_ 0.50) to give **8a** (14.0 mg, 0.06 mmol, 75%) as a colorless liquid. ^1^H NMR (500 MHz, CDCl_3_) δ 7.34–7.23 (m, 5H), 5.37 (s, 1H), 4.46 (q, *J* = 6.0 Hz, 1H), 3.68 (q, *J* = 5.0 Hz, 1H), 3.52–3.47 (m, 1H), 3.27–3.24 (m, 1H), 3.19 (dd, *J* = 9.0, 4.5 Hz, 1H), 3.12 (dd, *J* = 14.0, 6.0 Hz, 1H), 2.96 (dd, *J* = 14.0, 6.5 Hz, 1H), 1.83 (t, *J* = 5.0 Hz, 6H); ^13^C­{^1^H} NMR (126 MHz, CDCl_3_) δ 158.4, 135.1, 129.5, 128.7, 127.1, 79.4, 72.4, 69.9, 56.5, 40.5, 21.9, 21.8; HRMS (ESI) calcd for [M + H]^+^ (C_14_H_20_NO_3_) 250.1438, found 250.1424.

#### (4R*,5R*)-5-Benzyl-4-(methoxymethyl)­oxazolidin-2-one (**8b**)

Trifluoromethanesulfonic acid (1.1 μL, 1.9 mg, 0.01 mmol) was added to a solution of **1a** (12.3 mg, 0.07 mmol) and methanol (0.5 mL) at 0 °C. The reaction mixture was stirred at rt for 1 h and concentrated. The crude product was purified by column chromatography (SiO_2_, EtOAc/hexanes, 1:1, *R*_*f*_ 0.13) to give **8b** (12.8 mg, 0.06 mmol, 89%) as a colorless liquid. ^1^H NMR (500 MHz, CDCl_3_) δ 7.34–7.23 (m, 5H), 5.38 (br, 1H), 4.47 (ddd, *J* = 6.3, 6.3, 6.3 Hz, 1H), 3.70 (ddd, *J* = 5.0, 5.0, 5.0 Hz, 1H), 3.29 (s, 3H), 3.25 *J* = 3.22 (m, 1H), 3.17 *J* = 3.11 (m, 2H), 2.97 *J* = 2.93 (m, 1H); ^13^C­{^1^H} NMR (126 MHz, CDCl_3_) δ 158.7, 135.1, 129.5, 128.7, 127.1, 79.2, 74.2, 59.2, 56.2, 40.5; HRMS (ESI) calcd for [M + H]^+^ (C_12_H_16_NO_3_) 222.1125, found 222.1116.

#### (4R*,5R*)-5-Benzyl-4-(ethoxymethyl)­oxazolidin-2-one (**8c**)

Trifluoromethanesulfonic acid (1.1 μL, 1.9 mg, 0.01 mmol) was added to a solution of **1a** (11.2 mg, 0.06 mmol) and ethanol (0.5 mL) at 0 °C. The reaction mixture was stirred at rt for 1 h and concentrated. The crude product was purified by column chromatography (SiO_2_, EtOAc/hexanes, 1:1, *R*_*f*_ 0.14) to give **8c** (11.8 mg, 0.05 mmol, 85%) as a colorless liquid. ^1^H NMR (500 MHz, CDCl_3_) δ 7.33–7.23 (m, 5H), 5.60 (s, 1H), 4.47 (ddd, *J* = 5.9, 5.9, 5.9 Hz, 1H), 3.70 (ddd, *J* = 5.4, 5.4, 5.4 Hz, 1H), 3.44–3.39 (m, 2H), 3.29–3.26 (m, 1H), 3.22–3.19 (m, 1H), 3.14–3.10 (m, 1H), 2.97–2.93 (m, 1H), 1.13 (t, *J* = 7.1 Hz, 3H); ^13^C­{^1^H} NMR (126 MHz, CDCl_3_) δ 158.5, 135.1, 129.5, 128.7, 127.1, 79.3, 72.2, 67.0, 56.3, 40.5, 14.9; HRMS (ESI) calcd for [M + H]^+^ (C_13_H_18_NO_3_) 236.1281, found 236.1279.

#### (4R*,5R*)-5-Benzyl-4-(butoxymethyl)­oxazolidin-2-one (**8d**)

Trifluoromethanesulfonic acid (1.0 μL, 1.7 mg, 0.01 mmol) was added to a solution of **1a** (10.5 mg, 0.06 mmol), 1-butanol (41.3 mg, 0.55 mmol) and dichloromethane (0.5 mL) at 0 °C. The reaction mixture was stirred at rt for 1 h and concentrated. The crude product was purified by column chromatography (SiO_2_, EtOAc/hexanes, 1:1, *R*_*f*_ 0.50) to give **8d** (9.1 mg, 0.03 mmol, 62%) as a colorless liquid. ^1^H NMR (500 MHz, CDCl_3_) δ 7.33–7.22 (m, 5H), 5.45 (s, 1H), 4.47 (ddd, *J* = 6.3, 6.3, 6.3 Hz, 1H), 3.70 (ddd, *J* = 5.2, 5.2, 5.2 Hz, 1H), 3.38–3.34 (m, 2H), 3.28–3.25 (m, 1H), 3.21–3.18 (m, 1H), 3.14–3.10 (m, 1H), 2.97–2.93 (m, 1H), 1.51–1.45 (m, 2H), 1.35–1.27 (m, 2H), 0.89 (t, *J* = 7.4 Hz, 3H); ^13^C­{^1^H} NMR (126 MHz, CDCl_3_) δ 158.5, 135.1, 129.5, 128.7, 127.2, 79.4, 72.4, 71.5, 56.3, 40.5, 31.5, 19.2, 13.8; HRMS (ESI) calcd for [M + H]^+^ (C_15_H_22_NO_3_) 264.1594, found 264.1586.

#### (4R*,5R*)-5-Benzyl-4-((pentan-3-yloxy)­methyl)­oxazolidin-2-one (**8e**)

Trifluoromethanesulfonic acid (0.9 μL, 1.5 mg, 0.01 mmol) was added to a solution of **1a** (10.5 mg, 0.055 mmol), 3-pentanol (46.7 mg, 0.53 mmol) and dichloromethane (0.5 mL) at 0 °C. The reaction mixture was stirred at rt for 1 h and concentrated. The crude product was purified by column chromatography (SiO_2_, EtOAc/hexanes, 1:1, *R*_*f*_ 0.50) to give **8e** (10.5 mg, 0.04 mmol, 72%) as a colorless liquid. ^1^H NMR (500 MHz, CDCl_3_) δ 7.33–7.23 (m, 5H), 5.59 (d, *J* = 6.8 Hz, 1H), 4.48 (ddd, *J* = 6.4, 6.4, 6.4 Hz, 1H), 3.69 (ddd, *J* = 5.6, 5.6, 5.6 Hz, 1H), 3.29–3.26 (m, 1H), 3.20–3.18 (m, 1H), 3.14–3.10 (m, 1H), 3.06–3.03 (m, 1H), 2.98–2.94 (m, 1H), 1.44–1.39 (m, 4H), 0.85–0.81 (m, 6H); ^13^C­{^1^H} NMR (126 MHz, CDCl_3_) δ 158.6, 135.2, 129.5, 128.7, 127.1, 82.7, 79.5, 70.4, 56.7, 40.6, 25.7, 25.6, 9.5, 9.4; HRMS (ESI) calcd for [M + H]^+^ (C_16_H_24_NO_3_) 278.1751, found 278.1740.

#### (4R*,5R*)-5-Benzyl-4-((cyclopentyloxy)­methyl)­oxazolidin-2-one (**8f**)

Trifluoromethanesulfonic acid (1.0 μL, 1.4 mg, 0.01 mmol) was added to a solution of **1a** (9.0 mg, 0.05 mmol), cyclopentanol (41.3 mg, 0.48 mmol) and dichloromethane (0.5 mL) at 0 °C. The reaction mixture was stirred at rt for 1 h and concentrated. The crude product was purified by column chromatography (SiO_2_, EtOAc/hexanes, 1:1, *R*_*f*_ 0.50) to give **8f** (8.0 mg, 0.03 mmol, 62%) as a colorless liquid. ^1^H NMR (500 MHz, CDCl_3_) δ 7.36–7.25 (m, 5H), 5.43 (s, 1H), 4.45 (ddd, *J* = 6.3, 6.3, 6.3 Hz, 1H), 3.80 (m, 1H), 3.67 (ddd, *J* = 5.2, 5.2, 5.2 Hz, 1H), 3.25–3.22 (m, 1H), 3.17–3.14 (m, 1H), 3.14–3.10 (m, 1H), 2.97–2.93 (m, 1H), 1.63–1.49 (m, 8H); ^13^C­{^1^H} NMR (126 MHz, CDCl_3_) δ 158.4, 135.2, 129.5, 128.7, 127.1 82.2, 79.5, 70.5, 56.4, 40.6, 32.1, 23.4; HRMS (ESI) calcd for [M + H]^+^ (C_16_H_22_NO_3_) 276.1594, found 276.1589.

#### (4R*,5R*)-5-Benzyl-4-((cyclohexyloxy)­methyl)­oxazolidin-2-one (**8g**)

Trifluoromethanesulfonic acid (1.0 μL, 1.6 mg, 0.01 mmol) was added to a solution of **1a** (10.2 mg, 0.05 mmol) and cyclohexanol (0.5 mL) at 0 °C. The reaction mixture was stirred at rt for 1 h and concentrated. The crude product was purified by column chromatography (SiO_2_, EtOAc/hexanes, 1:1, *R*_*f*_ 0.50) to give **8g** (10.6 mg, 0.04 mmol, 68%) as a colorless liquid. ^1^H NMR (500 MHz, CDCl_3_) δ 7.33–7.23 (m, 5H), 5.31 (s, 1H), 4.46 (ddd, *J* = 6.0, 6.0, 6.0 Hz, 1H), 3.69–3.67 (m, 1H), 3.32–3.28 (m, 1H), 3.24–3.22 (m, 1H), 3.17–3.10 (m, 2H), 2.98–2.94 (m, 1H), 1.76–1.67 (m, 4H), 1.25–1.20 (m, 6H); ^13^C­{^1^H} NMR (126 MHz, CDCl_3_) δ 158.4, 135.2, 129.5, 129.0, 128.7, 127.1, 126.9, 79.5, 78.1, 69.7, 56.6, 40.6, 31.8, 25.6, 23.7; HRMS (ESI) calcd for [M + H]^+^ (C_17_H_24_NO_3_) 290.1751, found 290.1739.

#### (4R*,5R*)-5-Benzyl-4-(*tert*-butoxymethyl)­oxazolidin-2-one (**8h**)

Trifluoromethanesulfonic acid (1.1 μL, 1.9 mg, 0.01 mmol) was added to a solution of **1a** (12.0 mg, 0.06 mmol) and *tert-*butyl alcohol (0.5 mL) at 0 °C. The reaction mixture was stirred at rt for 1 h and concentrated. The crude product was purified by column chromatography (SiO_2_, EtOAc/hexanes, 1:1, *R*_*f*_ 0.57) to give **8h** (5.5 mg, 0.02 mmol, 43%) as a colorless liquid. ^1^H NMR (500 MHz, CDCl_3_) δ 7.33–7.23 (m, 5H), 5.40 (s, 1H), 4.46 (ddd, *J* = 6.0, 6.0, 6.0 Hz, 1H), 3.63 (ddd, *J* = 5.3, 5.3, 5.3 Hz, 1H), 3.22 (t, *J* = 8.1 Hz, 1H), 3.14–3.10 (m, 2H), 2.98–2.94 (m, 1H), 1.11 (s, 9H); ^13^C­{^1^H} NMR (126 MHz, CDCl_3_) δ 158.5, 135.3, 129.5, 128.7, 127.1, 79.6, 73.5, 64.0, 56.9, 40.6, 27.3; HRMS (ESI) calcd for [M + H]^+^ (C_15_H_22_NO_3_) 264.1594, found 264.1589.

#### (4R*,5R*)-4-((((1S*,3S*)-Adamantan-1-yl)­oxy)­methyl)-5-benzyloxazolidin-2-one (**8i**)

Trifluoromethanesulfonic acid (1.5 μL, 2.6 mg, 0.02 mmol) was added to a solution of **1a** (16.3 mg, 0.09 mmol), 1-adamantanol (131.3 mg, 0.86 mmol) and dichloromethane (1.0 mL) at 0 °C. The reaction mixture was stirred at rt for 1 h and concentrated. The crude product was purified by column chromatography (SiO_2_, EtOAc/hexanes, 1:1, *R*_*f*_ 0.50) to give **8i** (10.7 mg, 0.03 mmol, 36%) as a colorless liquid. ^1^H NMR (500 MHz, CDCl_3_) δ 7.34–7.23 (m, 5H), 5.39 (s, 1H), 4.45 (ddd, *J* = 6.5, 6.5, 6.5 Hz, 1H), 3.65–3.61 (m, 1H), 3.31–3.27 (m, 1H), 3.20–3.17 (m, 1H), 3.14–3.10 (m, 1H), 2.98–2.94 (m, 1H), 1.64–1.56 (m, 15H); ^13^C­{^1^H} NMR (126 MHz, CDCl_3_) δ 158.5, 135.3, 129.5, 128.7, 127.1, 79.7, 72.8, 64.2, 56.9, 41.5, 41.4, 40.6, 36.3, 30.4; HRMS (ESI) calcd for [M + H]^+^ (C_21_H_28_NO_3_) 342.2064, found 342.2057.

#### ((4R*,5R*)-5-Benzyl-2-oxooxazolidin-4-yl)­methyl Acetate (**8j**)

Trifluoromethanesulfonic acid (1.0 μL, 1.6 mg, 0.01 mmol) was added to a solution of **1a** (10.2 mg, 0.05 mmol), acetic acid (64.9 mg, 1.08 mmol) and dichloromethane (0.5 mL) at 0 °C. The reaction mixture was stirred at rt for 1 h and concentrated. The crude product was purified by column chromatography (SiO_2_, EtOAc/hexanes, 1:1, *R*_*f*_ 0.15) to give **8j** (8.1 mg, 0.03 mmol, 60%) as a colorless liquid. ^1^H NMR (500 MHz, CDCl_3_) δ 7.35–7.22 (m, 5H), 5.63 (s, 1H), 4.45 (ddd, *J* = 5.8, 5.8, 5.8 Hz, 1H), 3.99–3.96 (m, 1H), 3.87–3.84 (m, 1H), 3.77 (ddd, *J* = 5.2, 5.2, 5.2 Hz, 1H), 3.18–3.14 (m, 1H), 2.97–2.92 (m, 1H), 2.0 (s, 3H); ^13^C­{^1^H} NMR (126 MHz, CDCl_3_) δ 170.5, 158.2, 134.5, 129.5, 128.9, 127.4, 79.2, 64.7, 55.2, 40.4, 20.5; HRMS (ESI) calcd for [M + H]^+^ (C_13_H_16_NO_4_) 250.1074, found 250.1072.

#### (S*)-4-((R*)-2-Methoxy-6-methylhept-5-en-2-yl)­oxazolidin-2-one (**9a**)

Trifluoromethanesulfonic acid (3.5 μL, 6.0 mg, 0.04 mmol) was added to a solution of **1c** (38.5 mg, 0.20 mmol) and methanol (1.0 mL) at 0 °C. The reaction mixture was stirred at rt for 1 h and concentrated. The crude product was purified by column chromatography (SiO_2_, EtOAc/hexanes, 1:1, *R*_*f*_ 0.32) to give **9a** (39.1 mg, 0.17 mmol, 87%) as a colorless liquid. ^1^H NMR (500 MHz, CDCl_3_) δ 6.64 (br, 1H), 5.07 (m, 1H), 4.40–4.34 (m, 2H), 3.89 (dd, *J* = 8.5, 5.5 Hz, 1H), 3.19 (s, 3H), 1.96 (q, *J* = 7.0 Hz, 2H), 1.67 (s, 3H), 1.60 (s, 3H), 1.50 (q, *J* = 7.5 Hz, 2H), 1.12 (s, 3H) ; ^13^C NMR (126 MHz, CDCl_3_) δ 160.4, 132.1, 123.6, 66.3, 56.9, 49.5, 34.0, 25.6, 21.4, 17.6, 17.2; HRMS (ESI) calcd for [M + H]^+^ (C_12_H_22_NO_3_) 228.1594, found 228.1594. For the reaction conducted at 1 mmol scale, the above procedure was followed with **1c** (199.4 mg, 1.02 mmol), trifluoromethanesulfonic acid (18.0 μL, 30.7 mg, 0.20 mmol) and methanol (5.1 mL). The reaction mixture was stirred at rt for 1 h. Product **9a** (196.8 mg, 0.87 mmol, 85%) was isolated after column chromatography.

#### 2,4,6-Trichloro-*N*-((2S*,3R*)-1-hydroxy-3-methoxy-3,7-dimethyloct-6-en-2-yl)­benzamide (**9aa**)

Compound **9a** (60.1 mg, 0.26 mmol), potassium hydroxide (593.4 mg, 10.6 mmol), ethanol (1.3 mL) and water (0.13 mL) were added to a microwave reaction vessel. The resulted solution was sealed, heated to 120 °C (microwave oven, 300 W), stirred at 120 °C for 20 min, cooled to rt, diluted with water (15 mL) and extracted with dichloromethane (15 mL × 3). The combined organic layers were washed with saturated NaCl_(aq)_ (10 mL), dried over sodium sulfate, filtered and concentrated to give (2S*,3R*)-2-amino-3-methoxy-3,7-dimethyloct-6-en-1-ol (50.5 mg, 0.25 mmol, 95%) as a light-yellow liquid. The amino-alcohol (40.3 mg, 0.20 mmol) was redissolved in dichloromethane (1.0 mL), added with triethylamine (55.8 μL, 40.5 mg, 0.40 mmol) and 2,4,6-trichlorobenzoyl chloride (37.5 μL, 58.6 mg, 0.24 mmol) at 0 °C. The reaction mixture was stirred at rt for 16 h, added with water (10 mL) and extracted with dichloromethane (15 mL × 3). The combined organic layers were dried over sodium sulfate, filtered and concentrated. The crude product was purified by column chromatography (SiO_2_, EtOAc/hexanes, 1:1, *R*_*f*_ 0.73) to give **9aa** (74.4 mg, 0.18 mmol, 86%; two steps) as a colorless solid. Mp 136.0–139.0 °C; ^1^H NMR (500 MHz, CDCl_3_) δ 7.36 (s, 2H), 6.56 (d, *J* = 8.6 Hz, 1H), 5.09 (t, *J* = 6.5 Hz, 1H), 4.27–4.24 (m, 1H), 4.05 (dd, *J* = 3.1 Hz, *J* = 11.7 Hz, 1H), 3.78 (dd, *J* = 3.3 Hz, *J* = 11.7 Hz, 1H), 3.22 (s, 3H), 2.17 (brs, 1H), 2.03 (t, *J* = 5.6 Hz, 2H), 1.86–1.78 (m, 1H), 1.70 (s, 3H), 1.62 (s, 4H), 1.36 (s, 3H); ^13^C NMR (126 MHz, CDCl_3_) δ 163.9, 135.8, 134.7, 132.9, 132.5, 128.2, 123.3, 80.8, 62.8, 55.4, 49.1, 35.6, 25.6, 22.9, 19.8, 17.7; HRMS (ESI) calcd for [M + H]^+^ (C_18_H_25_Cl_3_NO_3_) 408.0895, found 408.0873. Single crystals suitable for X-ray analysis were grown by liquid diffusion between hexanes and a solution of **9aa** (20 mg) in chloroform (0.5 mL) at 25 °C.

#### (S*)-4-((R*)-2-Ethoxy-6-methylhept-5-en-2-yl)­oxazolidin-2-one (**9b**)

Trifluoromethanesulfonic acid (1.8 μL, 3.1 mg, 0.02 mmol) was added to a solution of **1c** (21.5 mg, 0.11 mmol) and ethanol (1.0 mL) at 0 °C. The reaction mixture was stirred at rt for 1 h and concentrated. The crude product was purified by column chromatography (SiO_2_, EtOAc/hexanes, 1:1, *R*_*f*_ 0.32) to give **9b** (22.6 mg, 0.094 mmol, 85%) as a colorless liquid. ^1^H NMR (500 MHz, CDCl_3_) δ 6.37 (br, 1H), 5.08 (t, *J* = 7.5 Hz, 1H), 4.42–4.35 (m, 2H), 3.89 (dd, *J* = 8.7 Hz, *J* = 5.1 Hz, 1H), 3.44–3.32 (m, 2H), 1.97 (q, *J* = 7.5 Hz, 2H), 1.67 (s, 3H), 1.60 (s, 3H), 1.55–1.43 (m, 2H), 1.13 (s, 3H), 1.12 (s, 3H); ^13^C NMR (126 MHz, CDCl_3_) δ 160.3, 132.1, 123.7, 76.8, 66.4, 57.1, 57.0, 34.6, 25.6, 21.5, 17.8,17.6, 15.8; HRMS (ESI) calcd for [M + H]^+^ (C_13_H_24_NO_3_) 242.1750, found 242.1746.

#### (S*)-4-((R*)-2-Butoxy-6-methylhept-5-en-2-yl)­oxazolidin-2-one (**9c**)

Trifluoromethanesulfonic acid (3.6 μL, 6.1 mg, 0.04 mmol) was added to a solution of **1c** (40.2 mg, 0.21 mmol), 1-butanol (152.7 mg, 2.06 mmol) and dichloromethane (0.5 mL) at 0 °C. The reaction mixture was stirred at rt for 1 h and concentrated. The crude product was purified by column chromatography (SiO_2_, EtOAc/hexanes, 1:1, *R*_*f*_ 0.55) to give **9c** (36.1 mg, 0.14 mmol, 65%) as a colorless liquid. ^1^H NMR (500 MHz, CDCl_3_) δ 6.67 (br, 1H), 5.08–5.06 (m, 1H), 4.41–4.33 (m, 2H), 3.88 (dd, *J* = 8.8 Hz, *J* = 5.0 Hz, 1H), 3.36–3.25 (m, 2H), 1.96 (q, *J* = 7.5 Hz, 2H), 1.66 (s, 3H), 1.59 (s, 3H), 1.50–1.44 (m, 4H), 1.37–1.30 (m, 2H), 1.12 (s, 3H), 0.89 (t, *J* = 7.4 Hz, 3H); ^13^C NMR (126 MHz, CDCl_3_) δ 160.5, 132.9, 123.8, 76.6, 66.4, 61.2, 57.1, 34.6, 32.4, 25.6, 21.5, 19.3, 17.7,17.6, 13.9; HRMS (ESI) calcd for [M + H]^+^ (C_15_H_28_NO_3_) 270.2064, found 270.2062.

#### (S*)-4-((R*)-2-Isopropoxy-6-methylhept-5-en-2-yl)­oxazolidin-2-one (**9d**)

Trifluoromethanesulfonic acid (2.9 μL, 4.9 mg, 0.03 mmol) was added to a solution of **1c** (32.5 mg, 0.17 mmol) and isopropyl alcohol (1.0 mL) at 0 °C. The reaction mixture was stirred at rt for 1 h and concentrated. The crude product was purified by column chromatography (SiO_2_, EtOAc/hexanes, 1:1, *R*_*f*_ 0.61) to give **9d** (33.2 mg, 0.14 mmol, 78%) as a colorless liquid. ^1^H NMR (500 MHz, CDCl_3_) δ 6.11 (br, 1H), 5.06 (t, *J* = 6.9 Hz, 1H), 4.37–4.36 (m, 2H), 3.87–3.81 (m, 2H), 2.03–1.98 (m, 2H), 1.69–1.67 (m, 3H), 1.60 (s, 3H), 1.47–1.43­(m, 2H), 1.16 (s, 3H), 1.11–1..09 (m, 6H); ^13^C NMR (126 MHz, CDCl_3_) δ 160.1, 132.1, 123.8, 77.4, 66.5, 64.1, 57.7, 35.8, 25.6, 25.0, 24.8, 21.8, 18.3, 17.7; HRMS (ESI) calcd for [M + H]^+^ (C_14_H_26_NO_3_) 256.1907, found 256.1893.

#### (S*)-4-((R*)-6-Methyl-2-(pentan-3-yloxy)­hept-5-en-2-yl)­oxazolidin-2-one (**9e**)

Trifluoromethanesulfonic acid (2.7 μL, 4.6 mg, 0.03 mmol) was added to a solution of **1c** (30.2 mg, 0.15 mmol), 3-pentanol (136.6 mg, 1.55 mmol) and dichloromethane (0.5 mL) at 0 °C. The reaction mixture was stirred at rt for 1 h and concentrated. The crude product was purified by column chromatography (SiO_2_, EtOAc/hexanes, 1:1, *R*_*f*_ 0.56) to give **9e** (32.9 mg, 0.11 mmol, 75%) as a colorless liquid. ^1^H NMR (500 MHz, CDCl_3_) δ 5.93 (br, 1H), 5.05 (t, *J* = 6.5 Hz, 1H), 4.41–4.34 (m, 2H), 3.84 (dd, *J* = 8.3 Hz, *J* = 5.7 Hz, 1H), 3.49–3.44 (m, 1H), 2.0 (q, *J* = 7.7 Hz, 2H), 1.68 (s, 3H), 1.60 (s, 3H), 1.50–1.42 (m, 6H), 1.16 (s, 3H), 0.85–0.82 (m, 6H); ^13^C NMR (126 MHz, CDCl_3_) δ 160.0, 132.2, 123.8, 77.2, 73.1, 66.5, 58.1, 36.1, 27.2, 25.6, 22.1, 18.5, 17.7, 9.1, 9.0; HRMS (ESI) calcd for [M + H]^+^ (C_16_H_30_NO_3_) 284.2220, found 284.2210.

#### (S*)-4-((R*)-2-(Cyclopentyloxy)-6-methylhept-5-en-2-yl)­oxazolidin-2-one (**9f**)

Trifluoromethanesulfonic acid (2.1 μL, 3.6 mg, 0.02 mmol) was added to a solution of **1c** (22.9 mg, 0.12 mmol), cyclopentanol (100.8 mg, 1.17 mmol) and dichloromethane (0.5 mL) at 0 °C. The reaction mixture was stirred at rt for 1 h and concentrated. The crude product was purified by column chromatography (SiO_2_, EtOAc/hexanes, 1:1, *R*_*f*_ 0.73) to give **9f** (22.3 mg, 0.08 mmol, 70%) as a colorless liquid. ^1^H NMR (500 MHz, CDCl_3_) δ 6.19 (br, 1H), 5.07 (m, 1H), 4.37–4.35 (m, 2H), 4.05 (m, 1H), 3.84–3.82 (m, 2H), 2.0–1.96 (m, 2H), 1.75–1.72 (m, 4H), 1.67 (s, 3H), 1.60 (s, 3H), 1.48–1.45 (m, 6H), 1.14 (s, 3H); ^13^C NMR (126 MHz, CDCl_3_) δ 160.2, 132.0, 123.8, 77.2, 73.7, 66.5, 57.6, 35.6, 34.9, 34.6, 25.6, 23.5, 23.5, 21.7, 18.8, 17.6; HRMS (ESI) calcd for [M + H]^+^ (C_16_H_28_NO_3_) 282.2064, found 282.2052.

#### (S*)-4-((R*)-2-(Cyclohexyloxy)-6-methylhept-5-en-2-yl)­oxazolidin-2-one (**9g**)

Trifluoromethanesulfonic acid (3.2 μL, 5.4 mg, 0.04 mmol) was added to a solution of **1c** (35.1 mg, 0.18 mmol) and cyclohexanol (1.0 mL) at 0 °C. The reaction mixture was stirred at rt for 1 h and concentrated. The crude product was purified by column chromatography (SiO_2_, EtOAc/hexanes, 1:1, *R*_*f*_ 0.63) to give **9g** (37.2 mg, 0.11 mmol, 75%) as a colorless liquid. ^1^H NMR (500 MHz, CDCl_3_) δ 6.35 (br, 1H), 5.05 (t, *J* = 6.2 Hz, 1H), 4.40–4.33 (m, 2H), 3.82 (dd, *J* = 8.5 Hz, *J* = 5.3 Hz, 1H), 3.47–3.45 (m, 1H), 2.00–1.97 (m, 2H), 1.67–1.64 (m, 6H), 1.60–1.59 (m, 4H), 1.45–1.43­(m, 4H), 1.28–1.25 (m, 4H), 1.53 (s, 3H); ^13^C NMR (126 MHz, CDCl_3_) δ 160.3, 132.0, 123.8, 77.4, 69.8, 66.5, 57.8, 35.9, 35.1, 34.9, 25.6, 25.5, 24.3, 21.9, 18.3, 17.7; HRMS (ESI) calcd for [M + H]^+^ (C_17_H_30_NO_3_) 296.2220, found 296.2214.

#### (*E*)-4-(6-Methylhepta-2,5-dien-2-yl)­oxazolidin-2-one (**10**)

Trifluoromethanesulfonic acid (2.3 μL, 3.9 mg, 0.03 mmol) was added to a solution of **1c** (20.3 mg, 0.10 mmol), *tert*-butyl alcohol (77.1 mg, 1.04 mmol) and dichloromethane (0.5 mL) at 0 °C. The reaction mixture was stirred at rt for 1 h and concentrated. The crude product was purified by column chromatography (SiO_2_, EtOAc/hexanes, 1:1, *R*_*f*_ 0.56) to give **10** (15.1 mg, 0.075 mmol, 75%) as a colorless liquid. ^1^H NMR (500 MHz, CDCl_3_) δ 5.41 (t, *J* = 6.5 Hz, 1H), 5.25 (br, 1H), 5.05 (t, *J* = 7.0 Hz, 1H), 4.48 (t, *J* = 8.8 Hz, 1H), 4.33 (t, *J* = 7.0 Hz, 1H), 4.10–4.07 (m, 1H), 2.72 (t, *J* = 6.5 Hz, 2H), 1.69 (s, 3H), 1.67 (s, 3H), 1.63 (s, 3H); ^13^C NMR (126 MHz, CDCl_3_) δ 159.9, 132.6, 131.7, 128.2, 121.5, 69.0, 59.3, 26.8, 25.6, 17.7, 11.0; HRMS (ESI) calcd for [M + H]^+^ (C_11_H_18_NO_2_) 196.1332, found 196.1332.

#### (R*)-6-Methyl-2-((S*)-2-oxooxazolidin-4-yl)­hept-5-en-2-yl Acetate (**9h**)

Trifluoromethanesulfonic acid (2.3 μL, 3.9 mg, 0.03 mmol) was added to a solution of **1c** (33.7 mg, 0.17 mmol), acetic acid (103.9 mg, 1.73 mmol) and dichloromethane (0.5 mL) at 0 °C. The reaction mixture was stirred at rt for 1 h and concentrated. The crude product was purified by column chromatography (SiO_2_, EtOAc/hexanes, 1:1, *R*_*f*_ 0.60) to give **9h** (30.5 mg, 0.12 mmol, 69%) as a colorless liquid. ^1^H NMR (500 MHz, CDCl_3_) δ 6.61 (br, 1H), 5.06 (m, 1H), 4.42–4.29 (m, 3H), 2.01 (s, 3H), 1.99 (m, 2H), 1.78–1.74 (m, 2H), 1.67 (s, 3H), 1.58 (s, 3H), 1.43 (s, 3H); ^13^C NMR (126 MHz, CDCl_3_) δ 170.0, 159.9, 132.6, 122.9, 84.1, 66.1, 57.3, 34.0, 25.6, 22.0, 21.8, 19.3, 17.6; HRMS (ESI) calcd for [M + Na]^+^ (C_13_H_21_NO_4_Na) 278.1363, found 278.1365.

#### (S*)-4-((R*)-2-Hydroxy-6-methylhept-5-en-2-yl)­oxazolidin-2-one (**9i**)

Trifluoromethanesulfonic acid (2.0 μL, 3.4 mg, 0.02 mmol) was added to a solution of **1c** (22.0 mg, 0.11 mmol), water (20.4 mg, 1.13 mmol) and dichloromethane (0.5 mL) at 0 °C. The reaction mixture was stirred at rt for 1 h and concentrated. The crude product was purified by column chromatography (SiO_2_, EtOAc/hexanes, 1:1, *R*_*f*_ 0.3) to give **9i** (19.5 mg, 0.09 mmol, 81%) as a colorless liquid. ^1^H NMR (500 MHz, CDCl_3_) δ 6.47 (br, 1H), 5.09 (t, *J* = 6.0 Hz, 1H), 4.40–4.38­(m, 2H), 3.78 (t, *J* = 7.5 Hz, 1H), 2.12–2.04 (m, 2H), 1.68 (s, 3H), 1.62 (s, 3H), 1.50–1.44 (m, 1H), 1.41–1.34 (m, 1H), 1.20 (s, 3H); ^13^C NMR (126 MHz, CDCl_3_) δ 160.5, 132.7, 123.6, 72.6, 66.2, 60.3, 36.8, 25.7, 22.8, 21.8, 17.7; HRMS (ESI) calcd for [M + Na]^+^ (C_11_H_19_NO_3_Na) 236.1257, found 236.1260.

#### (S*)-4-((R*)-Methoxy­(phenyl)­methyl)­oxazolidin-2-one (**11a**)

Iodosobenzene (48.5 mg, 0.22 mmol) was added to a suspension of **4d** (20.0 mg, 0.11 mmol) and molecular sieves (3 Å, 97 mg) in dichloromethane (0.6 mL). The reaction mixture was stirred at 50 °C (oil bath) for 3 h, filtered with a pad of Celite and concentrated. The residue was dissolved in dichloromethane (0.6 mL), and the resulting solution was added with methanol (45 μL, 35.2 mg, 1.1 mmol), cooled in an ice–water bath, added with trifluoromethanesulfonic acid (2.0 μL, 3.3 mg, 0.02 mmol), stirred at rt for 1 h and concentrated. The crude product was purified by column chromatography (SiO_2_, EtOAc/hexanes, 1:2, *R*_*f*_ 0.43) to give **11a** (18.5 mg, 0.09 mmol, 82%) as a colorless liquid. ^1^H NMR (500 MHz, CDCl_3_) δ 7.42–7.35 (m, 3H), 7.31–7.26 (m, 2H), 4.86 (br, 1H), 4.52–4.41 (m, 2H), 4.07 (d, *J* = 5.0 Hz, 1H), 3.92–3.88 (m, 1H), 3.24 (s, 3H); ^13^C­{^1^H} NMR (126 MHz, CDCl_3_) δ 159.0, 137.0, 129.1, 129.0, 127.3, 85.0, 68.1, 57.0, 56.9; HRMS (ESI) *m*/*z*: calcd for [M + Na]^+^ (C_11_H_13_NO_3_Na) 230.0788, found 230.0789.

#### (S*)-4-((R*)-Ethoxy­(phenyl)­methyl)­oxazolidin-2-one (**11b**)

Iodosobenzene (74.5 mg, 0.34 mmol) was added to a suspension of **4d** (30.0 mg, 0.17 mmol) and molecular sieves (3 Å, 150 mg) in dichloromethane (0.9 mL). The reaction mixture was stirred at 50 °C (oil bath) for 3 h, filtered with a pad of Celite and concentrated. The residue was dissolved in dichloromethane (0.9 mL), and the resulting solution was added with methanol (100 μL, 78.4 mg, 1.7 mmol), cooled in an ice–water bath, added with trifluoromethanesulfonic acid (3.0 μL, 5.1 mg, 0.03 mmol), stirred at rt for 1 h and concentrated. The crude product was purified by column chromatography (SiO_2_, EtOAc/hexanes, 1:2, *R*_*f*_ 0.27) to give **11b** (26.2 mg, 0.12 mmol, 70%) as a colorless liquid. ^1^H NMR (300 MHz, CDCl_3_) δ 7.41–7.26 (m, 5H), 5.31 (br, 1H), 4.49–4.39 (m, 2H), 4.18 (d, *J* = 6.0 Hz, 1H), 4.02–3.86 (m, 1H), 3.48–3.28 (t, *J* = 7.5 Hz, 3H), 3.48–3.28 (m, 2H); ^13^C­{^1^H} NMR (75 MHz, CDCl_3_) δ 159.2, 137.7, 128.9, 128.7, 127.1, 83.0, 68.0, 64.6, 57.0, 15.0; HRMS (ESI) *m*/*z*: calcd for [M + Na]^+^ (C_12_H_15_NO_3_Na) 244.0944, found 244.0938.

#### (S*)-4-((R*)-Butoxy­(phenyl)­methyl)­oxazolidin-2-one (**11c**)

Iodosobenzene (74.5 mg, 0.34 mmol) was added to a suspension of **4d** (30.0 mg, 0.17 mmol) and molecular sieves (3 Å, 149 mg) in dichloromethane (0.8 mL). The reaction mixture was stirred at 50 °C (oil bath) for 3 h, filtered with a pad of Celite and concentrated. The residue was dissolved in dichloromethane (0.8 mL), and the resulting solution was added with 1-butanol (125.5 mg, 1.7 mmol), cooled in an ice–water bath, added with trifluoromethanesulfonic acid (3.0 μL, 5.1 mg, 0.03 mmol), stirred at rt for 1 h and concentrated. The crude product was purified by column chromatography (SiO_2_, EtOAc/hexanes, 1:2, *R*_*f*_ 0.40) to give **11c** (30.3 mg, 0.12 mmol, 71%) as a colorless liquid. ^1^H NMR (300 MHz, CDCl_3_) δ 7.42–7.26 (m, 5H), 5.22 (brs, 1H), 4.50–4.39 (m, 2H), 4.14 (t, *J* = 7.5 Hz, 1H), 3.92–3.85 (m, 1H), 3.40–3.22 (m, 2H), 1.56–1.47 (m, 2H), 1.39–1.27 (m, 2H), 0.87 (t, *J* = 7.5 Hz, 3H); ^13^C­{^1^H} NMR (75 MHz, CDCl_3_) δ 159.2, 137.8, 128.9, 128.7, 127.1, 83.3, 69.0, 68.0, 57.1, 31.6, 19.2, 13.8; HRMS (ESI) *m*/*z*: calcd for [M + H]^+^ (C_14_H_20_NO_3_) 250.1437, found 250.1447.

#### (S*)-4-((R*)-Isopropoxy­(phenyl)­methyl)­oxazolidin-2-one (**11d**)

Iodosobenzene (74.5 mg, 0.34 mmol) was added to a suspension of **4d** (30.0 mg, 0.17 mmol) and molecular sieves (3 Å, 150 mg) in dichloromethane (0.9 mL). The reaction mixture was stirred at 50 °C (oil bath) for 3 h, filtered with a pad of Celite and concentrated. The residue was dissolved in dichloromethane (0.9 mL), and the resulting solution was added with isopropyl alcohol (130 μL, 102.2 mg, 1.7 mmol), cooled in an ice–water bath, added with trifluoromethanesulfonic acid (3.0 μL, 5.1 mg, 0.03 mmol), stirred at rt for 1 h and concentrated. The crude product was purified by column chromatography (SiO_2_, EtOAc/hexanes, 1:2, *R*_*f*_ 0.20) to give **11d** (23.9 mg, 0.12 mmol, 61%) as a colorless liquid. ^1^H NMR (500 MHz, CDCl_3_) δ 7.40–7.31 (m, 5H), 4.87 (brs, 1H), 4.48 (t, *J* = 7.5 Hz, 1H), 4.40 (dd, *J* = 5.0 Hz, *J* = 10.0 Hz, 1H), 4.27 (d, *J* = 10.0 Hz, 1H), 3.87–3.83 (m, 1H), 3.55–3.50 (m, 1H), 1.15 (d, *J* = 6.0 Hz, 3H), 1.07 (d, *J* = 6.0 Hz, 3H); ^13^C­{^1^H} NMR (126 MHz, CDCl_3_) δ 159.0, 138.5, 128.9, 128.8, 127.2, 80.5, 69.5, 68.3, 57.2, 23.3, 20.9; HRMS (ESI) *m*/*z*: calcd for [M + Na]^+^ (C_13_H_17_NO_3_Na) 258.1101, found 258.1095.

#### (S*)-4-((R*)-(Pentan-3-yloxy)­(phenyl)­methyl)­oxazolidin-2-one (**11e**)

Iodosobenzene (49.3 mg, 0.22 mmol) was added to a suspension of **4d** (20.0 mg, 0.11 mmol) and molecular sieves (3 Å, 99 mg) in dichloromethane (0.6 mL). The reaction mixture was stirred at 50 °C (oil bath) for 3 h, filtered with a pad of Celite and concentrated. The residue was dissolved in dichloromethane (0.6 mL), and the resulting solution was added with 3-pentanol (99.5 mg, 1.1 mmol), cooled in an ice–water bath, added with trifluoromethanesulfonic acid (2.0 μL, 3.4 mg, 0.02 mmol), stirred at rt for 1 h and concentrated. The crude product was purified by column chromatography (SiO_2_, EtOAc/hexanes, 1:1, *R*_*f*_ 0.70) to give **11e** (12.3 mg, 0.05 mmol, 46%) as a colorless liquid. ^1^H NMR (500 MHz, CDCl_3_) δ 7.40–7.26 (m, 5H), 4.68 (brs, 1H), 4.52 (t, *J* = 7.0 Hz, 1H), 4.43 (dd, *J* = 10.0, 5.0 Hz, 1H), 4.28 (d, *J* = 5.0 Hz, 1H), 3.93 (dt, *J* = 10.0, 5.0 Hz, 1H), 3.16 (dd, *J* = 10.0, 5.0 Hz, 1H), 1.60–1.52 (m, 2H), 1.41–1.35 (m, 2H), 0.87 (t, *J* = 4.5 Hz, 3H), 0.77 (s, 3H); ^13^C­{^1^H} NMR (126 MHz, CDCl_3_) δ 158.9, 138.4, 129.1, 128.9, 127.6, 80.6, 79.0, 68.4, 57.3, 26.1, 24.2, 9.7, 8.6; HRMS (ESI) *m*/*z*: calcd for [M + Na]^+^ (C_15_H_21_NO_3_Na) 286.1414, found 286.1413.

#### (S*)-4-((R*)-(Cyclopentyloxy)­(phenyl)­methyl)­oxazolidin-2-one (**11f**)

Iodosobenzene (74.5 mg, 0.34 mmol) was added to a suspension of **4d** (30.0 mg, 0.17 mmol) and molecular sieves (3 Å, 149 mg) in dichloromethane (0.9 mL). The reaction mixture was stirred at 50 °C (oil bath) for 3 h, filtered with a pad of Celite and concentrated. The residue was dissolved in dichloromethane (0.9 mL), and the resulting solution was added with cyclopentanol (146.4 mg, 1.7 mmol), cooled in an ice–water bath, added with trifluoromethanesulfonic acid (3.0 μL, 5.1 mg, 0.03 mmol), stirred at rt for 1 h and concentrated. The crude product was purified by column chromatography (SiO_2_, EtOAc/hexanes, 1:2, *R*_*f*_ 0.25) to give **11f** (31.9 mg, 0.12 mmol, 72%) as a colorless liquid. ^1^H NMR (300 MHz, CDCl_3_) δ 7.40–7.26 (m, 5H), 5.47 (br, 1H), 4.38 (dd, *J* = 9.0, 7.5 Hz, 2H), 4.23 (t, *J* = 7.0 Hz, 1H), 3.85 (dd, *J* = 12.0, 6.0 Hz, 2H), 1.69–1.56 (m, 4H), 1.51–1.39 (m, 4H); ^13^C­{^1^H} NMR (75 MHz, CDCl_3_) δ 159.4, 138.2, 128.8, 128.5, 127.1, 80.7, 79.3, 67.7, 57.2, 33.0, 31.3, 23.2; HRMS (FAB) *m*/*z*: calcd for [M + H]^+^ (C_15_H_20_NO_3_) 262.1443, found 262.1443.

#### (S*)-4-((R*)-(Cyclohexyloxy)­(phenyl)­methyl)­oxazolidin-2-one (**11g**)

Iodosobenzene (99.3 mg, 0.45 mmol) was added to a suspension of **4d** (40.0 mg, 0.23 mmol) and molecular sieves (3 Å, 198 mg) in dichloromethane (1.2 mL). The reaction mixture was stirred at 50 °C (oil bath) for 3 h, filtered with a pad of Celite and concentrated. The residue was dissolved in dichloromethane (1.2 mL), and the resulting solution was added with cyclopentanol (226.0 mg, 2.26 mmol), cooled in an ice–water bath, added with trifluoromethanesulfonic acid (4.0 μL, 6.8 mg, 0.05 mmol), stirred at rt for 1 h and concentrated. The crude product was purified by column chromatography (SiO_2_, EtOAc/hexanes, 1:2, *R*_*f*_ 0.23) to give **11g** (39.4 mg, 0.14 mmol, 63%) as a colorless liquid. ^1^H NMR (500 MHz, CDCl_3_) δ 7.43–7.29 (m, 5H), 4.85 (brs, 1H), 4.53–4.51 (m, 1H), 4.49–4.43 (m, 1H), 4.36–4.33 (m, 1H), 3.90–3.86 (m, 1H), 3.26–3.23 (m, 1H), 1.94–1.92 (m, 1H), 1.74–1.68 (m, 4H), 1.49 (s, 1H), 1.37–1.27 (m, 2H), 1.24–1.16 (m, 2H); ^13^C­{^1^H} NMR (126 MHz, CDCl_3_) δ 159.0, 138.7, 128.9, 128.7, 127.2, 80.2, 75.3, 68.3, 57.3, 33.4, 31.0, 25.6, 23.9, 23.7; HRMS (ESI) *m*/*z*: calcd for [M + Na]^+^ (C_16_H_21_NO_3_Na) 298.1414, found 298.1410.

#### (S*)-4-((R*)-*tert*-Butoxy­(phenyl)­methyl)­oxazolidin-2-one (**11h**)

Iodosobenzene (124.2 mg, 0.56 mmol) was added to a suspension of **4d** (50.0 mg, 0.28 mmol) and molecular sieves (3 Å, 248 mg) in dichloromethane (1.4 mL). The reaction mixture was stirred at 50 °C (oil bath) for 3 h, filtered with a pad of Celite and concentrated. The residue was dissolved in dichloromethane (1.4 mL), and the resulting solution was added with *tert*-butyl alcohol (209.1 mg, 2.82 mmol), cooled in an ice–water bath, added with trifluoromethanesulfonic acid (5.0 μL, 8.5 mg, 0.06 mmol), stirred at rt for 1 h and concentrated. The crude product was purified by column chromatography (SiO_2_, EtOAc/hexanes, 1:2, *R*_*f*_ 0.24) to give **11h** (25.3 mg, 0.10 mmol, 36%) as a colorless liquid. ^1^H NMR (300 MHz, CDCl_3_) δ 7.40–7.26 (m, 5H), 5.13 (brs, 1H), 4.43–4.33 (m, 3H), 3.80 (dd, *J* = 13.5 Hz, *J* = 6.0 Hz, 1H), 1.11 (s, 9H); ^13^C­{^1^H} NMR (75 MHz, CDCl_3_) δ 159.5, 141.2, 128.7, 128.1, 126.6, 75.4, 75.3, 67.5, 57.9, 28.7; HRMS (ESI) *m*/*z*: calcd for [M + Na]^+^ (C_14_H_19_NO_3_Na) 272.1257, found 272.1252.

#### (4S*)-4-((1R*)-(((1S*,3S*)-Adamantan-1-yl)­oxy)­(phenyl)­methyl)­oxazolidin-2-one (**11i**)

Iodosobenzene (149.0 mg, 0.68 mmol) was added to a suspension of **4d** (60.0 mg, 0.34 mmol) and molecular sieves (3 Å, 298 mg) in dichloromethane (1.7 mL). The reaction mixture was stirred at 50 °C (oil bath) for 3 h, filtered with a pad of Celite and concentrated. The residue was dissolved in dichloromethane (1.7 mL), and the resulting solution was added with 1-adamantanol (518.1 mg, 3.4 mmol), cooled in an ice–water bath, added with trifluoromethanesulfonic acid (6.0 μL, 10.2 mg, 0.07 mmol), stirred at rt for 1 h and concentrated. The crude product was purified by column chromatography (SiO_2_, EtOAc/hexanes, 1:2, *R*_*f*_ 0.24) to give **11i** (33.6 mg, 0.10 mmol, 30%) as a colorless liquid. ^1^H NMR (500 MHz, CDCl_3_) δ 7.40–7.27 (m, 5H), 5.29 (br, 1H), 4.56 (d, *J* = 10.0 Hz, 1H), 4.44–4.40 (m, 1H), 4.32 (t, *J* = 9.0 Hz, 1H), 3.80–3.76 (m, 1H), 2.06 (s, 3H), 1.70–1.50 (m, 12H); ^13^C­{^1^H} NMR (126 MHz, CDCl_3_) δ 159.4, 141.5, 128.6, 128.0, 126.6, 74.6, 73.6, 67.6, 57.9, 42.7, 36.1, 30.5. HRMS (FAB) *m*/*z*: calcd for [M + H]^+^ (C_20_H_26_NO_3_) 328.1913, found 328.1906.

#### (*S*)-2,2-Dimethyl-4-(2-methylpent-1-en-1-yl)-1,3-dioxolane (**13**)

A solution of 2-bromopentane (1.71 mL, 2.07 g, 13.6 mmol), triphenylphosphine (3.0 g, 11.4 mmol) and acetonitrile (3 mL) was placed in a sealed tube, heated in a 90 °C bath (oil) for 120 h and concentrated to give pentan-2-yltriphenylphosphonium bormoide (4.15 g, 10.0 mmol, 88%) as a colorless solid (mp 183.0–185.0 °C). *n*-BuLi (1.6 *M* in hexanes, 1.06 mL, 1.71 mmol) was added to a solution of pentan-2-yltriphenylphosphonium bormoide (768.8 mg, 1.86 mmol) and THF (7.0 mL) at −30 °C. The reaction mixture was stirred at −30 °C for 30 min, rasied to 0 °C and added with aldehyde **12** (201.7 mg, 1.55 mmol, in 3 mL of THF), stirred at 0 °C for another 2 h, quenched with water (5 mL) and extracted with diethyl ether (10 mL × 3). The combined organic layers were washed with saturated NaCl_(aq)_ (5 mL), dried over sodium sulfate, filtered and concentrated. The crude product was purified by column chromatography (SiO_2_, EtOAc/hexanes, 1:20, *R*_*f*_ 0.52) to give **13** (191.4 mg, 1.04 mmol, 67%; *E/Z* = 4:5) as a colorless liquid. [α]^20^_D_ = +4.6 (*c* = 0.35, CH_2_Cl_2_); ^1^H NMR (500 MHz, CDCl_3_) δ 5.17–5.15 (m, 1H), 4.83–4.73 (m, 1H), 4.04–3.98 (m, 1H), 3.45–3.48 (m, 1H), 2.14–1.96 (m, 2H), 1.72 (s, *Z-*allylic CH_3_), 1.68 (s, *E-*allylic CH_3_), 1.47–1.34 (m, 8H), 0.88–0.86 (m, 3H); ^13^C NMR (126 MHz, CDCl_3_) δ 142.4, 142.0, 122.6, 121.8, 108.7, 73.0, 72.7, 69.5, 69.5, 41.7, 34.2, 26.8, 26.0, 23.5, 21.4, 20.6, 16.5, 13.8, 13.7; HRMS (ESI) *m*/*z*: [M + H]^+^ calcd for C_11_H_21_O_2_ 185.1532, found 185.1536.

#### (*S*)-4-Methylhept-3-ene-1,2-diol (**14**)

A solution of **13** (191.4 mg, 1.04 mmol) and methanol (5 mL) was added HCl_(aq)_ (1.0 *M*, 1.0 mL, 1.0 mmol), and the resulted solution was stirred at rt for 60 min, neutralized with triethylamine (101.2 mg, 1.0 mmol) and concentrated. The crude product was purified by column chromatography (SiO_2_, EtOAc/hexanes, 1:1, *R*_*f*_ 0.27) to give **14** (148.4 mg, 1.03 mmol, 99%) as a colorless liquid. [α]^20^_D_ = +19.1 (*c* = 0.09, CH_2_Cl_2_); ^1^H NMR (300 MHz, CDCl_3_) δ 5.13 (brs, 1H), 4.47 (brs, 1H), 3.54 (brs, 2H), 3.46 (brs, 2H), 2.71 (brs, 2H), 2.10–1.94 (m, 2H), 1.70 (s, *Z-*allylic CH_3_), 1.67 (s, *E-*allylic CH_3_), 1.49–1.24 (m, 2H), 0.91–0.83 (m, 3H); ^13^C NMR (75 MHz, CDCl_3_) δ 141.7, 141.1, 123.6, 122.9, 69.4, 69.0, 66.6, 66.4, 41.6­(*E*), 34.4­(*Z*), 23.4, 21.3, 20.6, 16.6, 13.9, 13.6;HRMS (ESI) *m*/*z*: [M + H]^+^ calcd for C_8_H_17_O_2_ 145.1223, found 145.1226.

#### (*S*)-1-((*tert*-Butyldimethylsilyl)­oxy)-4-methylhept-3-en-2-ol (**15**)

*tert*-Butyldimethylsilyl chloride (186.3 mg, 1.24 mmol) was added to a solution of **14** (148.4 mg, 1.03 mmol), imidazole (105.2 mg, 1.55 mmol) ans dichloromethane (6.0 mL) at 0 °C. The reaction mixture was stirred at rt for another 2 h, added with water (5 mL) and extracted with dichloromethane (5 mL × 3). The combined organic layers were washed with saturated NaCl_(aq)_ (5 mL), dried over sodium sulfate, filtered and concentrated. The crude product was purified by column chromatography (SiO_2_, EtOAc/hexanes, 1:3, *R*_*f*_ 0.59) to give **15** (210.3 mg, 0.81 mmol, 79%) as a colorless liquid. [α]^20^_D_ = +14.1 (*c* = 1.25, CH_2_Cl_2_); ^1^H NMR (300 MHz, *E/Z* = 0.6/1, CDCl_3_) δ 5.08 (d, 1H, J = 8.7 Hz), 4.44–4.36 (m, 1H), 3.55–3.50 (m, 1H), 3.42–3.34 (m, 1H), 2.54 (brs, 1H), 2.13–1.90 (m, 2H), 1.69 (s, *Z-*allylic CH_3_), 1.65 (s, *E-*allylic CH_3_), 1.49–1.26 (m, 2H), 0.88 (s, 9H), 0.85 (s, 3H), 0.05 (s, 6H); ^13^C NMR (75 MHz, CDCl_3_) δ 141.1, 140.5, 123.5, 122.8, 69.1, 68.7, 67.2, 66.9, 41.6, 34.4, 25.8, 23.3, 21.4, 20.6, 16.6, 13.9, 13.6, −5.33, −5.40; HRMS (ESI) *m*/*z*: [M + H]^+^ calcd for C_14_H_31_O_2_Si 259.2071, found 259.2087.

#### (*S*)-1-((*tert*-Butyldimethylsilyl)­oxy)-4-methylhept-3-en-2-yl carbamate (**16**)

Trichloroacetyl isocyanate (183.1 mg, 0.97 mmol) was added to a solution of **15** (210.3 mg, 0.81 mmol) and dichloromethane (5 mL). The reaction mixture was stirred at rt for 2 h, then added with methanol (1 mL), water (1 mL) and potassium carbonate (559.7 mg, 4.05 mmol), stirred for another 2 h and concentrated. The residue was added with water (3 mL) and extracted with dichloromethane (10 mL × 2). The combined organic layers were washed with saturated NaCl_(aq)_ (5 mL), dried over sodium sulfate, filtered and concentrated. The crude product was purified by column chromatography (SiO_2_, EtOAc/hexanes, 1:3, *R*_*f*_ 0.47) to give **16** (234.4 mg, 0.78 mmol, 96%) as a colorless liquid. [α]^20^_D_ = −5.05 (*c* = 0.095, CH_2_Cl_2_); ^1^H NMR (300 MHz, *E/Z* = 0.95/1, CDCl_3_) δ 5.49–5.41 (m, 1H), 5.11 (t, *J* = 9.4 Hz, 1H), 4.80 (brs, 2H), 3.68–3.58 (m, 2H), 2.23–1.94 (m, 2H), 1.71 (s, 3H), 1.51–1.24 (m, 2H), 0.87 (s, 12H), 0.04 (s, 6H); ^13^C NMR (75 MHz, CDCl_3_) δ 156.7, 156.6, 142.7, 142.1, 120.7, 120.1, 72.8, 72.3, 65.3, 65.0, 41.6­(*E*), 34.5­(*Z*), 25.8, 23.4, 21.3, 20.6, 18.3, 16.8, 13.9, 13.6, −0.38; HRMS (ESI) *m*/*z*: [M + H]^+^ calcd for C_15_H_32_NO_3_Si 302.2135, found 302.2146.

#### (4*S*,5*R*)-4-(((*tert*-Butyldimethylsilyl)­oxy)­methyl)-6-methyl-6-propyl-3-oxa-1-azabicyclo­[3.1.0]­hexan-2-one (**17**)

Iodosobenzene (220.01 mg, 1.0 mmol) was added to a solution of **16** (151.3 mg, 0.5 mmol) and dichloromethane (5 mL). The reaction mixture was stirred for 8 h at 50 °C (oil bath), cooled to rt, filtered with a pad of Celite and concentrated. The crude product was purified by column chromatography (SiO_2_, EtOAc/hexanes, 1:3, *R*_*f*_ 0.76) to give **17** (83.9 mg, 0.28 mmol, 56%) as a colorless liquid. [α]^20^_D_ = +7.29 (*c* = 0.38, CH_2_Cl_2_); ^1^H NMR (300 MHz, CDCl_3_) δ 4.39–4.35 (m, 1H), 3.85–3.83­(m, 2H), 4.15 (t, *J* = 1.8 Hz, 1H), 1.74–1.41 (m, 4H), 1.35 (s, 3H), 0.98–0.90 (m, 3H), 0.89 (s, 9H), 0.08 (s, 6H); ^13^C NMR (75 MHz, CDCl_3_, major diastereomer) δ 165.0, 75.7, 63.7, 52.4, 51.1, 40.7, 30.9, 25.8, 22.7, 18.3, 14.1, 13.2, −5.5; HRMS (ESI) *m*/*z*: [M + H]^+^ calcd for C_15_H_30_NO_3_Si 300.1976, found 300.1989.

#### (4*S*,5*S*)-5-(Hydroxymethyl)-4-(2-methoxypentan-2-yl)­oxazolidin-2-one (**18**)

A solution of **17** (83.9 mg, 0.28 mmol) and methanol (1.0 mL) was added with trifluoromethanesulfonic acid (12.6 mg, 0.084 mmol) at 0 °C. The reaction mixture was stirred at rt for 1 h and concentrated. The crude product was purified by column chromatography (SiO_2_, EtOAc, *R*_*f*_ 0.57) to give **18** (45.6 mg, 0.21 mmol, 75%) as a colorless liquid. [α]^20^_D_ = +7.64 (*c* = 0.57, CH_2_Cl_2_); ^1^H NMR (300 MHz, CDCl_3,_ major diastereomer) δ 6.29 (brs, 1H), 4.41­(brs, 1H), 3.81–3.74 (m, 2H), 3.62–3.59 (m, 2H), 3.18 (s, 3H), 2.45 (brs, 1H), 1.43–1.40 (m, 2H), 1.33–1.24 (m, 2H), 1.10 (s, 3H), 0.91 (t, *J* = 7.0 Hz, 3H); ^13^C NMR (75 MHz, CDCl_3_, major diastereomer) δ 159.6, 82.0, 78.5, 64.1, 58.8, 49.4, 35.2, 17.4, 16.1, 14.5; HRMS (ESI) *m*/*z*: [M + H]^+^ calcd for C_10_H_20_NO_4_ 218.1384, found 218.1387.

#### *N*-((2*S*)-1,2-Dihydroxy-4-methoxy-4-methylheptan-3-yl)­acetamide (**19**)

Compound **18** (45.6 mg, 0.21 mmol), potassium hydroxide (117.8 mg, 2.1 mmol), ethanol (4.0 mL) and water (1.0 mL) were added to a microwave reaction vessel. The resulted solution was sealed, heated to 120 °C (microwave oven, 300 W), stirred at 120 °C for 10 min, cooled to rt, extracted with ethyl acetate (7 mL × 3). The combined organic layers were washed with saturated NaCl_(aq)_ (5 mL), dried over sodium sulfate, filtered and concentrated. The residue was redissolved in dichloromethane (1.0 mL), added with triethylamine (58.5 μL, 0.42 mmol) and acetic anhydride (25.7 mg, 0.25 mmol). The reaction mixture was stirred at rt for another 2 h, added with water (1 mL) and extracted with dichloromethane (5 mL × 3). The combined organic layers were dried over sodium sulfate, filtered and concentrated. The crude product was purified by column chromatography (SiO_2_, CH_3_OH/CH_2_Cl_2_, 1:10, *R*_*f*_ 0.46) to give **19** (44.6 mg, 0.19 mmol, 91%) as a colorless liquid. ^1^H NMR (300 MHz, CDCl_3_, major diastereomer) δ 6.20 (d, *J* = 9.0 Hz, 1H), 4.19–4.10 (m, 1H), 4.04 (d, *J* = 9.7 Hz, 1H), 3.84 (brs, 1H), 3.58–3.51 (m, 1H), 3.25 (s, 3H), 3.22–3.17 (m, 1H), 2.07­(s, 3H), 1.67–1.41 (m, 1H), 1.38 (s, 3H), 1.34–1.05 (m, 1H), 1.12–1.11 (m, 3H), 0.98–0.86 (m, 3H); ^13^C NMR (75 MHz, CDCl_3_, major diastereomer) δ 171.7, 81.7, 70.9, 62.3, 52.2, 48.8, 38.1, 22.9, 20.1, 17.7, 14.8; HRMS (ESI) *m*/*z*: [M + H]^+^ calcd for C_11_H_24_NO_4_ 234.1694, found 234.1700.

#### *N*-(3-Methoxy-3-methyl-1-oxohexan-2-yl)­acetamide (**20**)

Sodium periodate (52.8 mg, 0.25 mmol) was added to a solution of **19** (44.6 mg, 0.19 mmol), acetonitrile (1.0 mL) and water (0.5 mL) at rt. The reaction mixture was stirred for 2 h, added with water (1 mL) and extracted with dichloromethane (5 mL × 3). The combined organic layers were dried over sodium sulfate, filtered and concentrated. The crude product was purified by column chromatography (SiO_2_, EtOAc/hexanes, 3:1, *R*_*f*_ 0.53) to give **20** (30.6 mg, 0.15 mmol, 80%; dr = 5:4) as a colorless liquid. [α]^20^_D_ = −52.9 (*c* = 0.24, CH_2_Cl_2_); ^1^H NMR (300 MHz, CDCl_3_) δ 9.73 (s, 1H), 9.68 (s, 1H), 6.16 (brs, 1H), 4.70 (d, *J* = 8.0 Hz, 1H,), 4.50 (d, *J* = 7.4 Hz, 1.5H), 2.07 (s, 3H), 2.06 (s, 3H), 1.68–1.40 (m, 4H), 1.31 (s, 3H), 1.28–1.24 (m, 3H), 1.15 (s, 3H), 0.95–0.88 (m, 3H); ^13^C NMR (126 MHz, CDCl_3_) δ 200.3, 199.9, 170.6, 170.4, 79.5, 78.8, 63.3, 62.6, 49.6, 49.4, 37.9, 37.6, 23.1, 23.0, 19.9, 16.8, 14.5; HRMS (ESI) *m*/*z*: [M + H]^+^ calcd for C_10_H_20_NO_3_ 202.1443, found 202.1434. The spectroscopic data were consistent with the reported values.^22^

## Supplementary Material





## Data Availability

The data underlying this study are available in the published article and its Supporting Information.

## Notes

Crystal data and structure refinement for **5a**, **5a′**, **9aa**, **11d**, and **11i** and copies of ^1^H and ^13^C­{^1^H} NMR spectra for all synthesized compounds (PDF)
FAIR data, including the primary NMR FID files, for compounds **1a**, **1a′**, **1d**, **3a**, **3b**, **4a**, **4b**, **5a**, **5a′**, **5b**, **5c**, **8a**–**8i**, **9a**–**9i**, **9aa**, **10**, **11a**–**11i** and **12**–**20** (ZIP)
