# Supplementary material for: Stereocontrolled Ring-Opening of Oxazolidinone-Fused Aziridines for the Synthesis of 2‑Amino Ethers
Source: J Org Chem. 2025 Jul 7;90(28):10017–30. doi: 10.1021/acs.joc.5c01068 (PMC12281577; doi:10.1021/acs.joc.5c01068)
Supplement: Supplementary file 1 [file jo5c01068_si_001.pdf]

Supporting Information

Stereo-controlled Ring-Opening of Oxazolidinone-Fused Aziridines for the Synthesis of 2-Amino Ethers.

Tsung-Lun Tsai, Chin-Hsun Wu, Chih-Ling Lo, Chi-Sheng Wen and Duen-Ren Hou*

Department of Chemistry, National Central University, No. 300 Jhong-Da Rd., Jhong-li, Taoyuan, Taiwan, 320317.

Table of Contents

Table S1. Screening of solvents for the ring-opening reaction of **1a** with isopropyl alcohol. ……S7

Table S2. Crystal structure and refinement data for compound **5a**………………….………..S8

Table S3. Crystal structure and refinement data for compound **5a'**…………………..……..S9

Figure S1. ORTEP of **5a'**……. ……………………………………………..………………..…S10

Table S4. Crystal structure and refinement data for compound **9aa**……. …………………...S11

Table S5. Crystal structure and refinement data for compound **11d**……. …………………...S12

Table S6. Crystal structure and refinement data for compound **11i**…… ……………………..S13

^{1}H NMR of compound **3a**……...........….....................…...S14

^{13}C {^{1}H} NMR of compound **3a**…...........…...S15

^{1}H NMR of compound **3b**..........…........…...................…...S16

^{13}C {^{1}H} NMR of compound **3b.**...........…...S17

^{1}H NMR of compound **4a**..........…........…...................…...S18

^{13}C {^{1}H} NMR of compound **4a**……...S19

^{1}H NMR of compound **1a**……......…........….................…...S20

^{13}C {^{1}H} NMR of compound **1a**...…...……………..S21

^{1}H NMR of compound **1a'**……......…........….................…...S22

^{13}C {^{1}H} NMR of compound **1a'**…........…...…….S23

^{1}H NMR of compound **5a**……......…........….................…...S24

^{13}C {^{1}H} NMR of compound **5a**……......…...….S25

^{1}H NMR of compound **5a'**……......…........….................…...S26

^{1}H NMR of compound **4b**……......…........….................…...S27

^{13}C {^{1}H} NMR of compound **4b**…..…S28

^{1}H NMR of compound **5b**……......…........….................…...S29

^{13}C {^{1}H} NMR of compound **5b**........…...….S30

^{1}H NMR of compound **5c**……..….........…..........................…............................S31

^{13}C {^{1}H} NMR of compound **5c**...……S32

^{1}H NMR of compound **4d**……..….........…..........................…............................S33

^{13}C {^{1}H} NMR of compound **4d**..........…...…..…S34

^{1}H NMR of compound **1d**……..….........…..........................…............................S35

^{13}C {^{1}H} NMR of compound **1d**..........…...……S36

^{1}H NMR of compound **8a**……..….........…..........................…..............................S37

^{13}C {^{1}H} NMR of compound **8a**.....…...……S38

^{1}H NMR of compound **8b**.....…..........…..........................…..................................S39

^{13}C {^{1}H} NMR of compound **8b**..........…...……S40

^{1}H NMR of compound **8c**……......….........…..........................…..........................S41

^{13}C {^{1}H} NMR of compound **8c**...……S42

^{1}H NMR of compound **8d**……..….........…..........................…..............................S43

^{13}C {^{1}H} NMR of compound **8d**...……S44

^{1}H NMR of compound **8e**……..….........…..........................…..............................S45

^{13}C {^{1}H} NMR of compound **8e**..........…...……S46

^{1}H NMR of compound **8f**……......….........…..........................…...........................S47

^{13}C {^{1}H} NMR of compound **8f**…...……S48

^{1}H NMR of compound **8g**……..….........…..........................…..............................S49

^{13}C {^{1}H} NMR of compound **8g**..........…...……S50

^{1}H NMR of compound **8h**……..….........…..........................…..............................S51

^{13}C {^{1}H} NMR of compound **8h**...……S52

^{1}H NMR of compound **8i**……..….........…..........................…...............................S53

^{13}C {^{1}H} NMR of compound **8i**..……S54

^{1}H NMR of compound **8j**……..….........…..........................…...............................S55

^{13}C {^{1}H} NMR of compound **8j**..……S56

^{1}H NMR of compound **9a**……......…...........….........................….............................S57

^{13}C {^{1}H} NMR of compound **9a**..……S58

^{1}H NMR of compound **9aa**……......…...........….........................….............................S59

^{13}C {^{1}H} NMR of compound **9aa**..……S60

^{1}H NMR of compound **9b**……......…...........….........................….............................S61

^{13}C {^{1}H} NMR of compound **9b**..……S62

^{1}H NMR of compound **9c**...….............…...…...................S63

^{13}C {^{1}H} NMR of compound **9c**………………………….......……....................S64

^{1}H NMR of compound **9d**...….............…...…...................S65

^{13}C {^{1}H} NMR of compound **9d**………………………….......……....................S66

^{1}H NMR of compound **9e**...….............…...…...................S67

^{13}C {^{1}H} NMR of compound **9e**………………………….......……....................S68

^{1}H NMR of compound **9f**...….............…...…...................S69

^{13}C {^{1}H} NMR of compound **9f**………………………….......……....................S70

^{1}H NMR of compound **9g**...….............…...…...................S71

^{13}C {^{1}H} NMR of compound **9g**………………………….......……....................S72

^{1}H NMR of compound **10**...….............…...…...................S73

^{13}C {^{1}H} NMR of compound **10**………………………….......……....................S74

^{1}H-^{1}H COSY of compound **10**………………...……….......……....................S75

^{1}H NMR of compound **9h**...….............…...…...................S76

^{13}C {^{1}H} NMR of compound **9h**………………………….......……....................S77

^{1}H NMR of compound **9i**...….............…...…...................S78

^{13}C {^{1}H} NMR of compound **9i**………………………….......……....................S79

^{1}H NMR of compound **11a**...….............…...…...................S80

^{13}C {^{1}H} NMR of compound **11a**………………………….......……....................S81

^{1}H NMR of compound **11b**...….............…...…...................S82

^{13}C {^{1}H} NMR of compound **11b**……………………….......……....................S83

^{1}H NMR of compound **11c**....…........…..…..................S84

^{13}C {^{1}H} NMR of compound **11c**……………………….......……....................S85

^{1}H NMR of compound **11d**....…........…..…..................S86

^{13}C {^{1}H} NMR of compound **11d**……………………….......……....................S87

^{1}H NMR of compound **11e**....…........…..…..................S88

^{13}C {^{1}H} NMR of compound **11e**……………………….......……....................S89

^{1}H NMR of compound **11f**....…........…..…..................S90

^{13}C {^{1}H} NMR of compound **11f**……………………….......……....................S91

^{1}H NMR of compound **11g**....…........…..…..................S92

^{13}C {^{1}H} NMR of compound **11g**……………………….......……....................S93

^{1}H NMR of compound **11h** *syn*....…........…..…............S94

^{13}C {^{1}H} NMR of compound **11h** *syn*..………………….......……....................S95

^{1}H NMR of compound **11i** *syn*..…........…......................................…..................S96

^{13}C {^{1}H} NMR of compound **11i** *syn*..………………….......……....................S97

^{1}H NMR of compound **13**....…........…..…..................S98

^{13}C {^{1}H} NMR of compound **13**……………………….......……....................S99

^{1}H NMR of compound **14**....…........…..…..................S100

^{13}C {^{1}H} NMR of compound **14**……………………….......……....................S101

^{1}H NMR of compound **15**....…........…..…..................S102

^{13}C {^{1}H} NMR of compound **15**……………………….......……....................S103

^{1}H NMR of compound **16**....…........…..…..................S104

^{13}C {^{1}H} NMR of compound **16**……………………….......……....................S105

^{1}H NMR of compound **17**....…........…..…..................S106

^{13}C {^{1}H} NMR of compound **17**……………………….......……....................S107

^{1}H NMR of compound **18**....…........…..…..................S108

^{13}C {^{1}H} NMR of compound **18**……………………….......…….....................S109

^{1}H NMR of compound **19**....….........….......................................….................S110

^{13}C {^{1}H} NMR of compound **19**……………………….......…….....................S111

^{1}H NMR of compound **20**....….........….......................................….................S112

^{13}C {^{1}H} NMR of compound **20**……………………….......…….....................S113

Table S1. Screening of solvents for the ring-opening reaction of compound **1a** with isopropyl alcohol.[a]

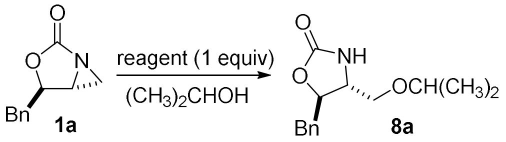

entry	Lewis acid	solvent	condition	**8a** (%)
1	TfOH (0.2 equiv)	CH_2Cl_2	25 °C, 1 h	75
2	$Cu(OTf)_2$, (0.5 equiv)	CH_2Cl_2	50 °C,[b] 5 min	65
3	$Cu(OTf)_2$, (0.5 equiv)	$(CH_2Cl)_2$	50 °C,[b] 5 min	50
4	$Cu(OTf)_2$, (0.5 equiv)	$CHCl_3$	50 °C,[b] 5 min	50
5	$Cu(OTf)_2$, (0.5 equiv)	benzene	50 °C,[b] 5 min	40
6	$Cu(OTf)_2$, (0.5 equiv)	toluene	50 °C,[b] 5 min	33
7	CuF_2, (1.0 equiv)	$CHCl_3$	80 °C,[b] 5 min	0
8	CuF_2, (1.0 equiv)	toluene	80 °C,[b] 5 min	0

[a]To a solution of **1a** (18.9 mg, 0.10 mmol) and a solvent (0.5 mL) in an ice-water bath, Lewis acid (0.10 mmol) was added. After stirred at the specified reaction temperature and time, the reaction mixture was concentrated, diluted with water (3 mL) and extracted with ethyl acetate (3 mL × 3). The combined organic layers were dried over sodium sulfate, concentrated and purified by column chromatography to isolate **8a**.

[b]Microwave heating.

Table S2. Crystal structure and refinement data for compound **5a** (CCDC2402518). [a]

```
Bond precision:   C-C = 0.0031 A          Wavelength=0.71073
Cell:          a=34.1500(6)  b=8.6460(2)     c=10.6815(2)
               alpha=90      beta=105.311(1) gamma=90
Temperature: 296 K
                  Calculated                  Reported
Volume            3041.89(11)                 3041.89(11)
Space group       C 2/c                       C 2/c
Hall group        -C 2yc                      -C 2yc
Moiety formula    C17 H17 N O2 S              ?
Sum formula       C17 H17 N O2 S              C17 H17 N O2 S
Mr                299.38                      299.37
Dx,g cm-3         1.307                       1.307
Z                 8                           8
Mu (mm-1)         0.216                       0.216
F000              1264.0                      1264.0
F000'             1265.49
h,k,lmax          45,11,14                    45,11,14
Nref              3798                        3789
Tmin,Tmax         0.975,0.982                 0.690,0.746
Tmin'             0.952
Correction method= # Reported T Limits: Tmin=0.690
Tmax=0.746 AbsCorr = 'N
Data completeness= 0.998   Theta(max)= 28.316
R(reflections)= 0.0435( 2878)          wR2(reflections)=
                                       0.1209( 3789)
S = 1.022          Npar= 196
```

[a]Single crystals of compound **5a** suitable for X-ray crystallographic analysis were obtained *via* vapor diffusion between hexanes (~4 mL) and a dichloromethane solution of pure **5a** (20.0 mg in ~0.5 mL) at 25 °C.

Table S3. Crystal structure and refinement data for compound **5a'** (CCDC2402520). [a]

Bond precision: C-C = 0.0033 A Wavelength=0.71073

Cell: a=9.1527(4) b=9.3248(4) c=9.6765(4)

alpha=100.779(2) beta=106.494(2) gamma=102.896(2)

Temperature: 100 K

	Calculated	Reported
Volume	743.69(6)	743.69(6)
Space group	P -1	P -1
Hall group	-P 1	-P 1
Moiety formula	C17 H17 N O2 S	?
Sum formula	C17 H17 N O2 S	C17 H17 N O2 S
Mr	299.38	299.37
Dx,g cm-3	1.337	1.337
Z	2	2
Mu (mm-1)	0.221	0.221
F000	316.0	316.0
F000'	316.37	
h,k,lmax	10,11,11	10,11,11
Nref	2627	2618
Tmin,Tmax	0.960,0.980	0.707,0.746
Tmin'	0.960	

Correction method= # Reported T Limits: Tmin=0.707 Tmax=0.746 AbsCorr = 'N

Data completeness= 0.997 Theta(max)= 25.019

R(reflections)= 0.0474(2467) wR2(reflections)= 0.1189(2618)

S = 1.084 Npar= 190

[a] Single crystals of compound **5a'** suitable for X-ray crystallographic analysis were obtained *via* vapor diffusion between hexanes (~4 mL) and a dichloromethane solution of pure **5a'** (17.0 mg in ~0.4 mL) at 25 °C.

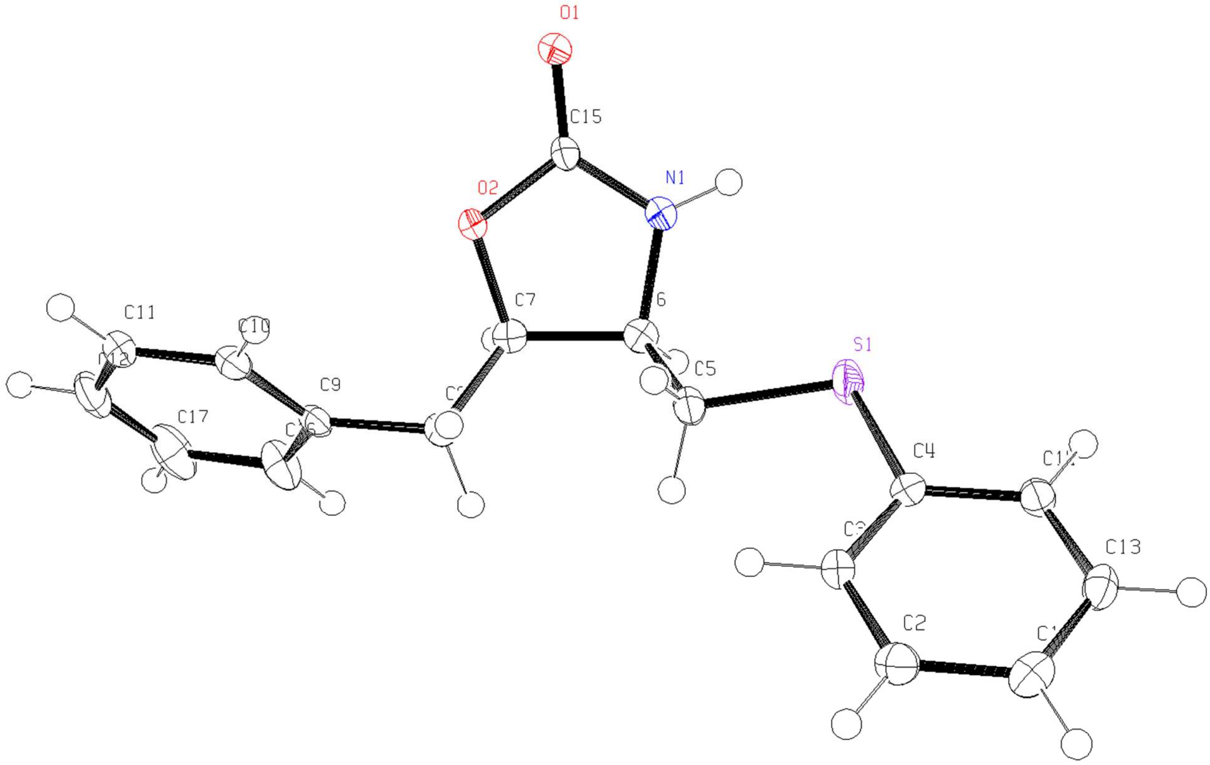

Figure S1. ORTEP of compound **5a'**.

Table S4. Crystal structure and refinement data for compound **9aa** (CCDC2447519). [a]

Bond precision:	C-C = 0.0051 A	Wavelength=0.71073
Cell:	a=7.2197(2) b=12.4059(6) c=22.9396(10)	
	alpha=90 beta=98.094(1) gamma=90	
Temperature:	298 K	
	Calculated	Reported
Volume	2034.16(14)	2034.16(14)
Space group	P 21/n	P 21/n
Hall group	-P 2yn	-P 2yn
Moiety formula	C18 H24 Cl3 N O3	C18 H24 Cl3 N O3
Sum formula	C18 H24 Cl3 N O3	C18 H24 Cl3 N O3
Mr	408.73	408.73
Dx,g cm-3	1.335	1.335
Z	4	4
Mu (mm-1)	0.467	0.467
F000	856.0	856.0
F000'	858.09	
h,k,lmax	8,14,27	8,14,27
Nref	3596	3594
Tmin,Tmax	0.970,0.980	0.662,0.745
Tmin'	0.953	

Correction method= # Reported T Limits: Tmin=0.662 Tmax=0.745 AbsCorr = MULTI-SCAN

Data completeness= 0.999 Theta(max)= 25.028

R(reflections)= 0.0687(2868) wR2(reflections)= 0.1314(3594)

S = 1.190 Npar= 233

[a] Single crystals suitable for X-ray analysis were grown by liquid diffusion between hexanes and a solution of **9aa** (20 mg) in chloroform (0.5 mL) at 25 °C.

Table S5. Crystal structure and refinement data for compound **11d** (CCDC2402252). [a]

Bond precision: C-C = 0.0018 A Wavelength=1.54184

Cell: a=6.35952(13) b=8.69132(19) c=11.7144(3)
alpha=86.7337(18) beta=74.9506(19) gamma=79.3119(18)

Temperature: 100 K

	Calculated	Reported
Volume	614.40(3)	614.40(2)
Space group	P -1	P -1
Hall group	-P 1	-P 1
Moiety formula	C13 H17 N O3	C13 H17 N O3
Sum formula	C13 H17 N O3	C13 H17 N O3
Mr	235.28	235.27
Dx,g cm-3	1.272	1.272
Z	2	2
Mu (mm-1)	0.737	0.737
F000	252.0	252.0
F000'	252.79	
h,k,lmax	7,10,14	7,10,14
Nref	2449	2368
Tmin,Tmax	0.923,0.964	0.949,1.000
Tmin'	0.915	

Correction method= # Reported T Limits: Tmin=0.949 Tmax=1.000 AbsCorr = MULTI-SCAN

Data completeness= 0.967 Theta(max)= 72.734

R(reflections)= 0.0357(2207) wR2(reflections)= 0.0908(2368)

S = 1.030 Npar= 157

[a] Single crystals of compound **11d** suitable for X-ray crystallographic analysis were obtained *via* liquid-liquid diffusion between hexanes (~3 mL) and a d-chloroform solution of pure **11d** (15.0 mg in 0.3 mL) at 25 °C.

Table S6. Crystal structure and refinement data for compound **11i** (CCDC2402253). [a]

Bond precision:	C-C = 0.0018 A		Wavelength=1.54184
Cell:	a=6.77721(7)	b=9.56765(9)	c=40.5223(3)
	alpha=94.5941(7)	beta=93.1260(7)	gamma=98.4251(8)
Temperature:	100 K		
	Calculated		Reported
Volume	2584.98(4)		2584.98(4)
Space group	P -1		P -1
Hall group	-P 1		-P 1
Moiety formula	C20 H25 N O3		C20 H25 N O3
Sum formula	C20 H25 N O3		C20 H25 N O3
Mr	327.41		327.41
Dx,g cm-3	1.262		1.262
Z	6		6
Mu (mm-1)	0.673		0.673
F000	1056.0		1056.0
F000'	1059.10		
h,k,lmax	8,11,50		8,11,49
Nref	10338		10022
Tmin,Tmax	0.984,0.987		0.558,1.000
Tmin'	0.880		

Correction method= # Reported T Limits: Tmin=0.558 Tmax=1.000 AbsCorr = MULTI-SCAN

Data completeness= 0.969 Theta(max)= 72.967

R(reflections)= 0.0368(8587) wR2(reflections)= 0.0987(10022)

S = 1.067 Npar= 662

[a] Single crystals of compound **11i** suitable for X-ray crystallographic analysis were obtained *via* liquid-liquid diffusion between hexanes (~4 mL) and a dichloromethane solution of pure **11d** (30.0 mg in 0.4 mL) at 25 °C.

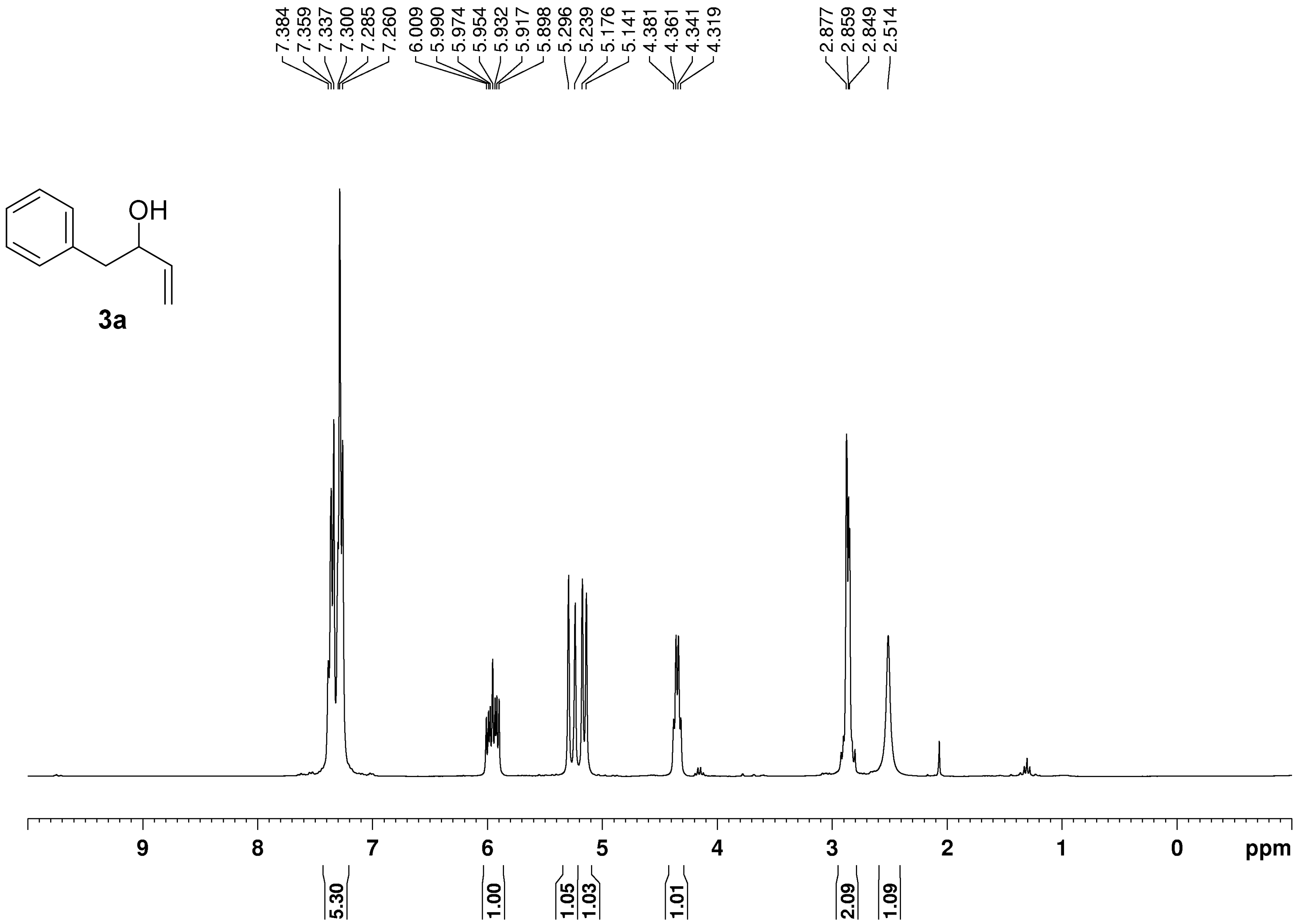

^{1}H NMR of compound **3a** (300 MHz, $CDCl_3$)

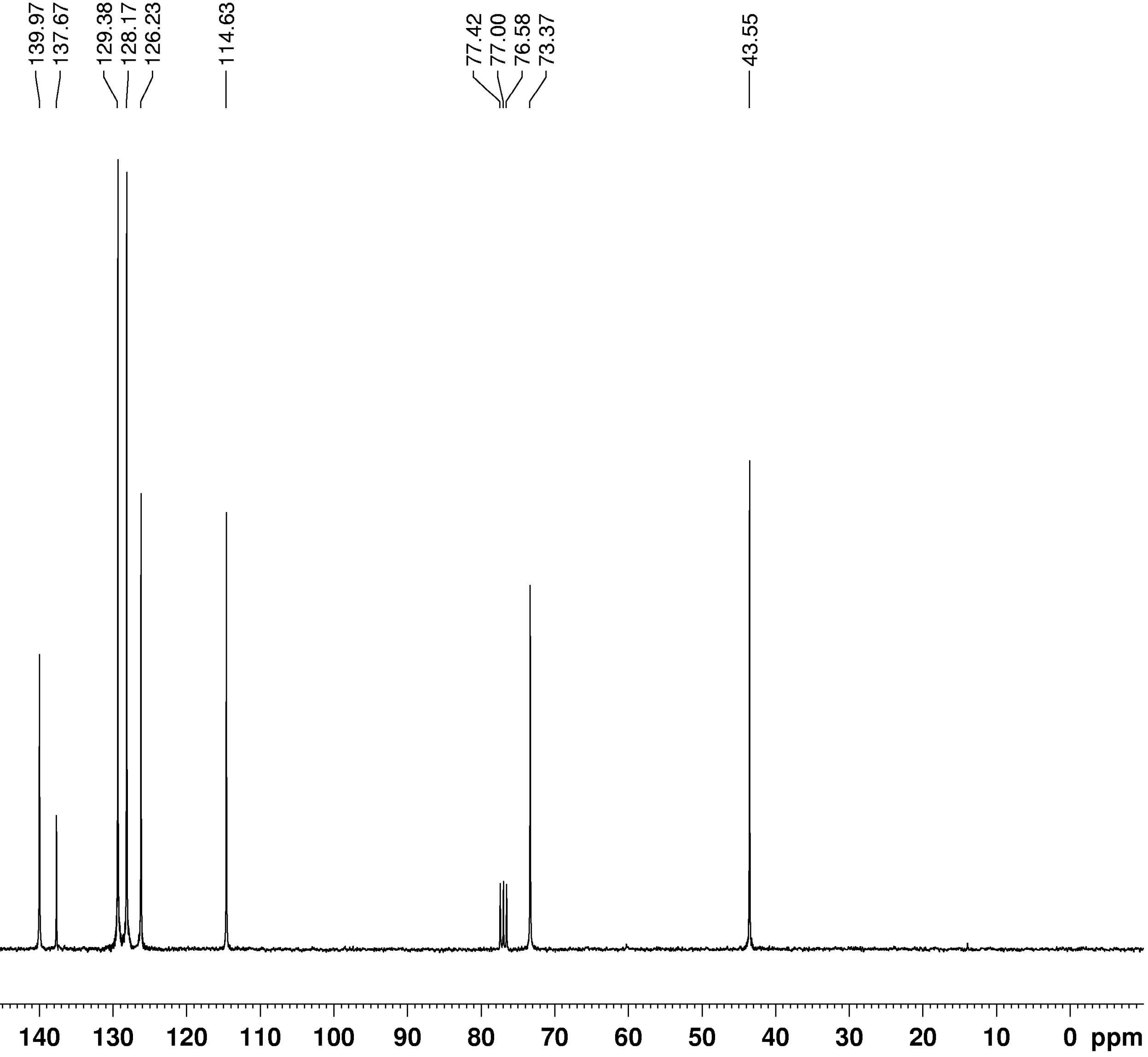

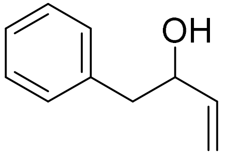

3a

$^{13}C\ \{^{1}H\}$ NMR of compound **3a** (75 MHz, $CDCl_3$)

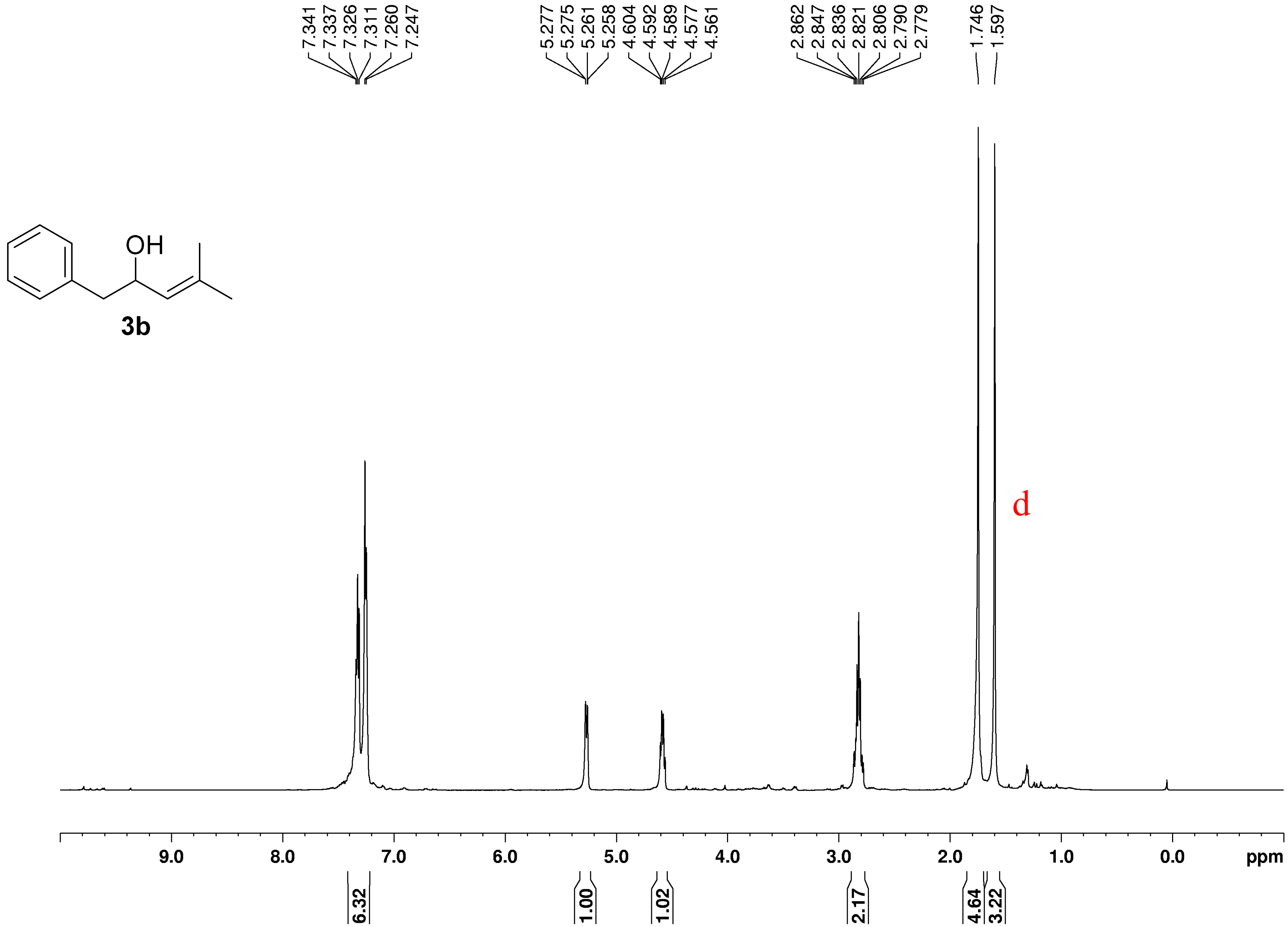

^{1}H NMR of compound **3b** (500 MHz, $CDCl_3$)

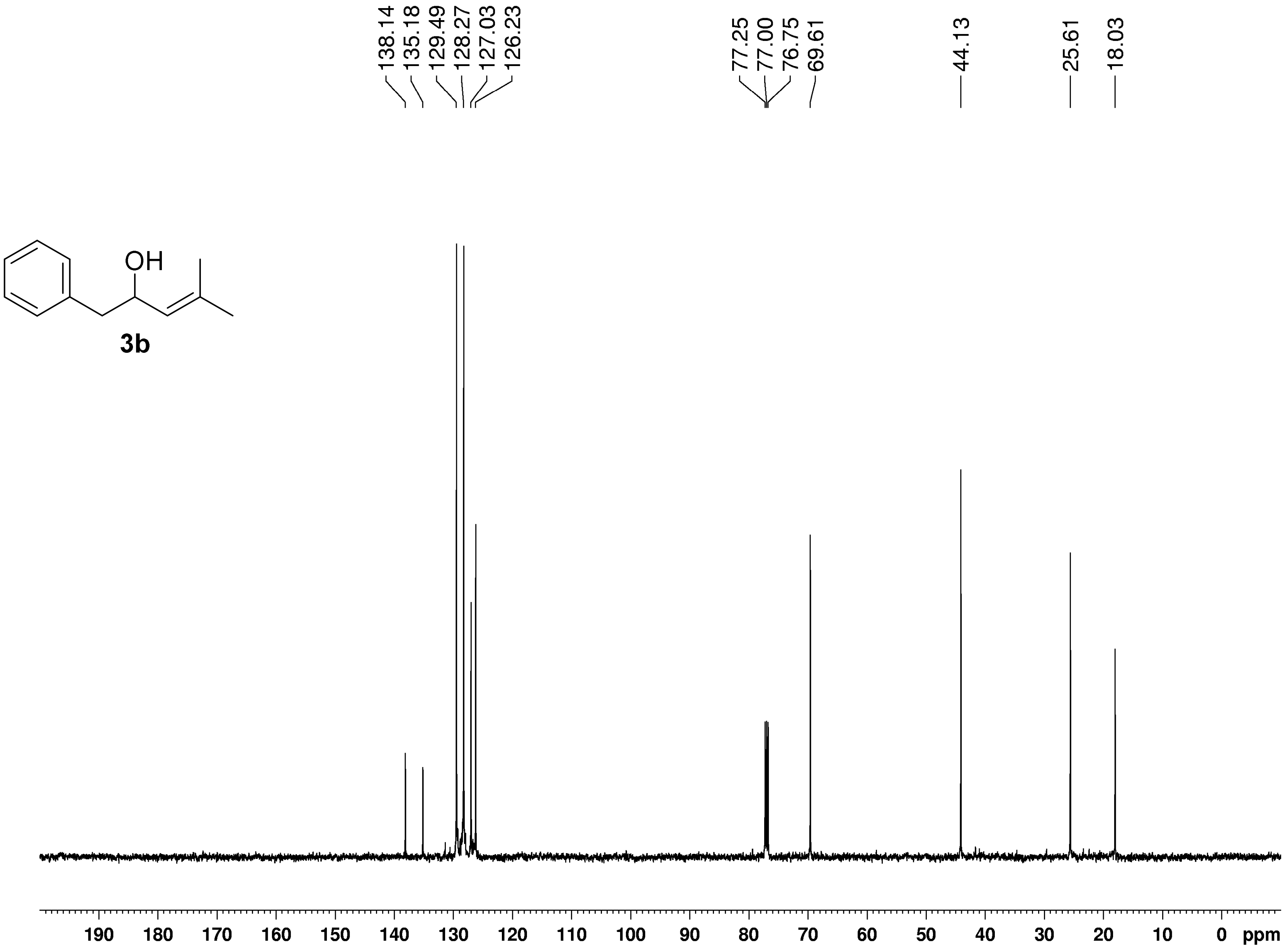

^{13}C {^{1}H} NMR of compound **3b** (126 MHz, $CDCl_3$)

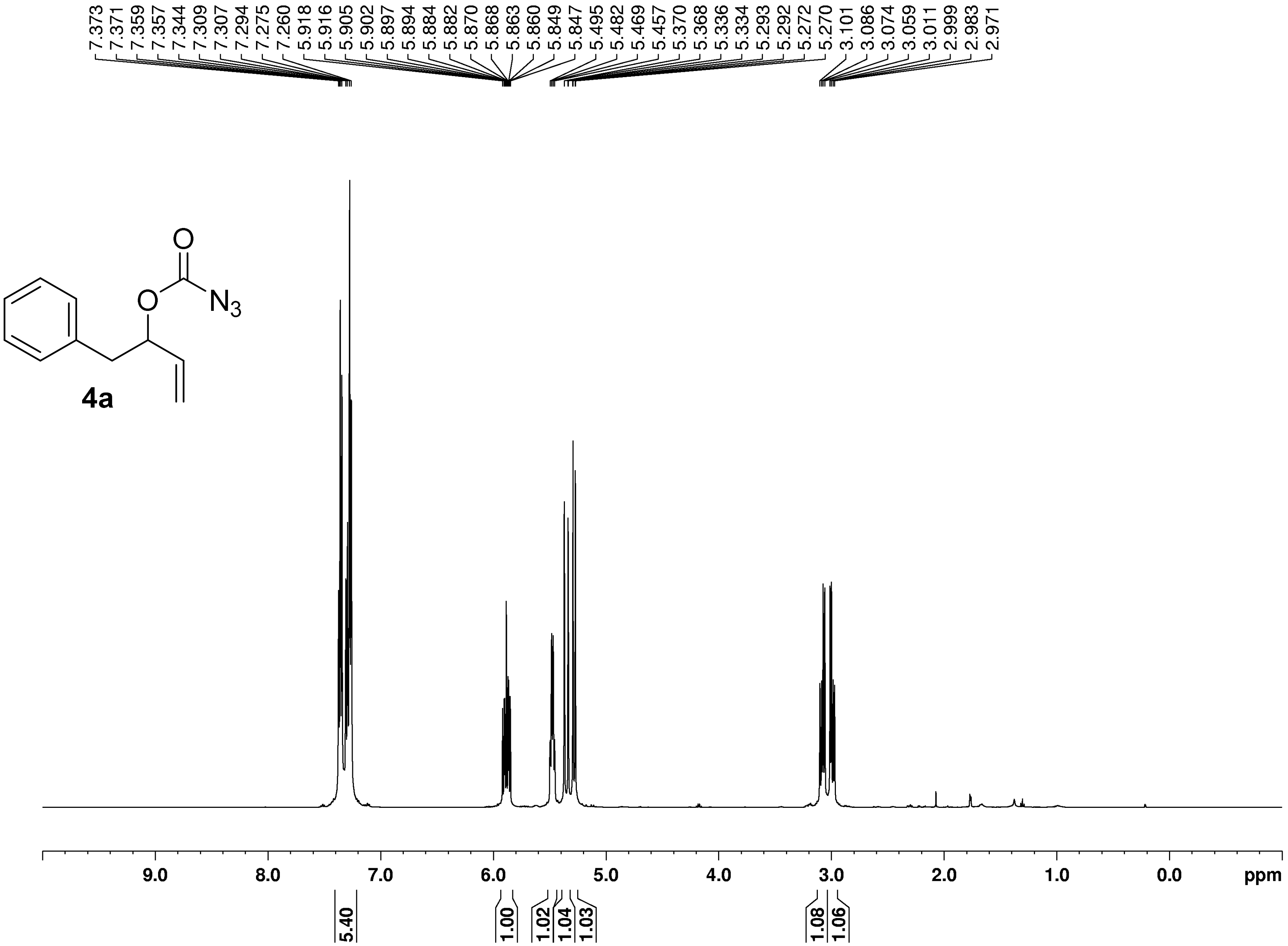

^{1}H NMR of compound **4a** (500 MHz, $CDCl_3$)

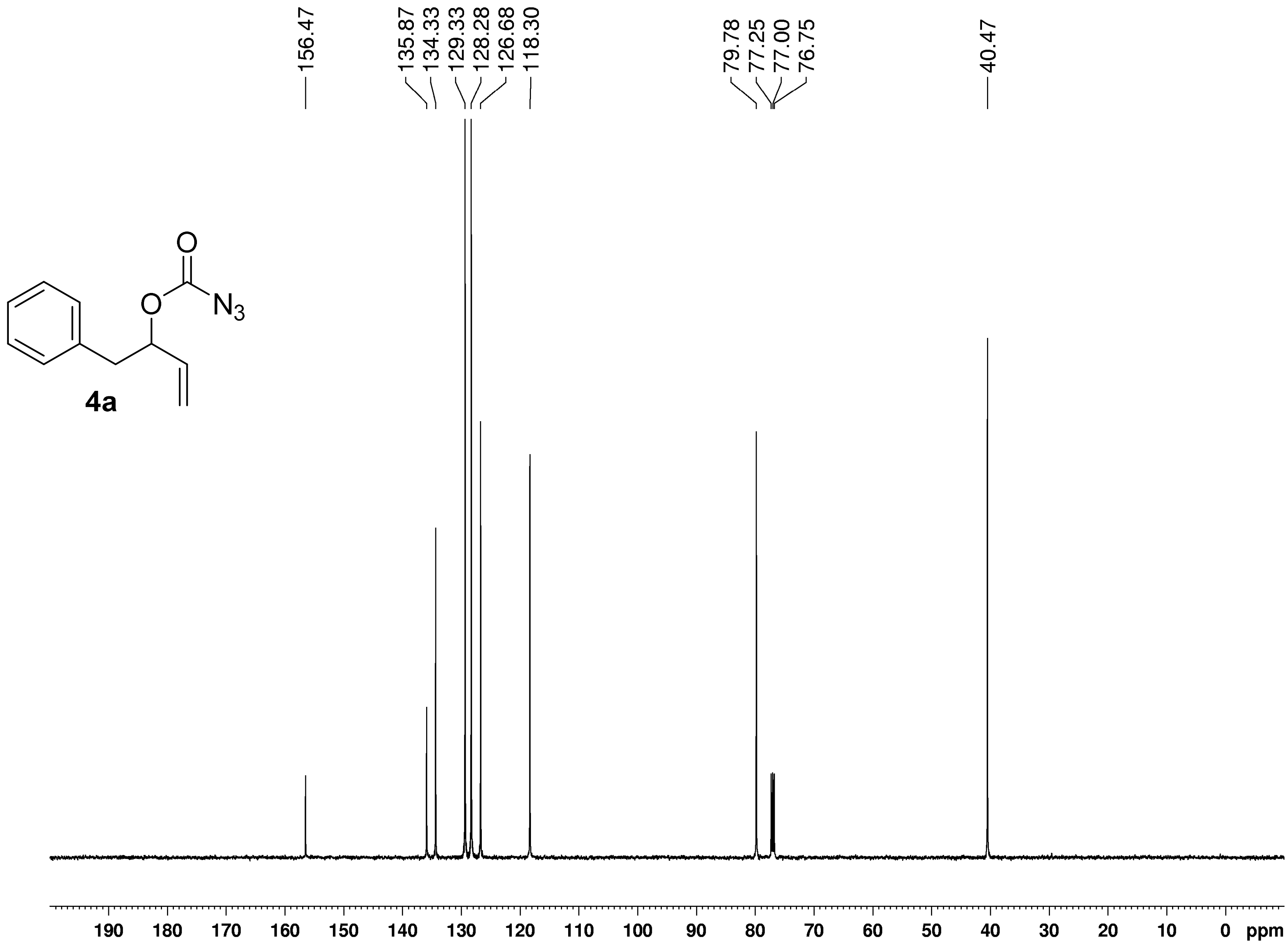

$^{13}C\{^{1}H\}$ NMR of compound **4a** (126 MHz, $CDCl_3$)

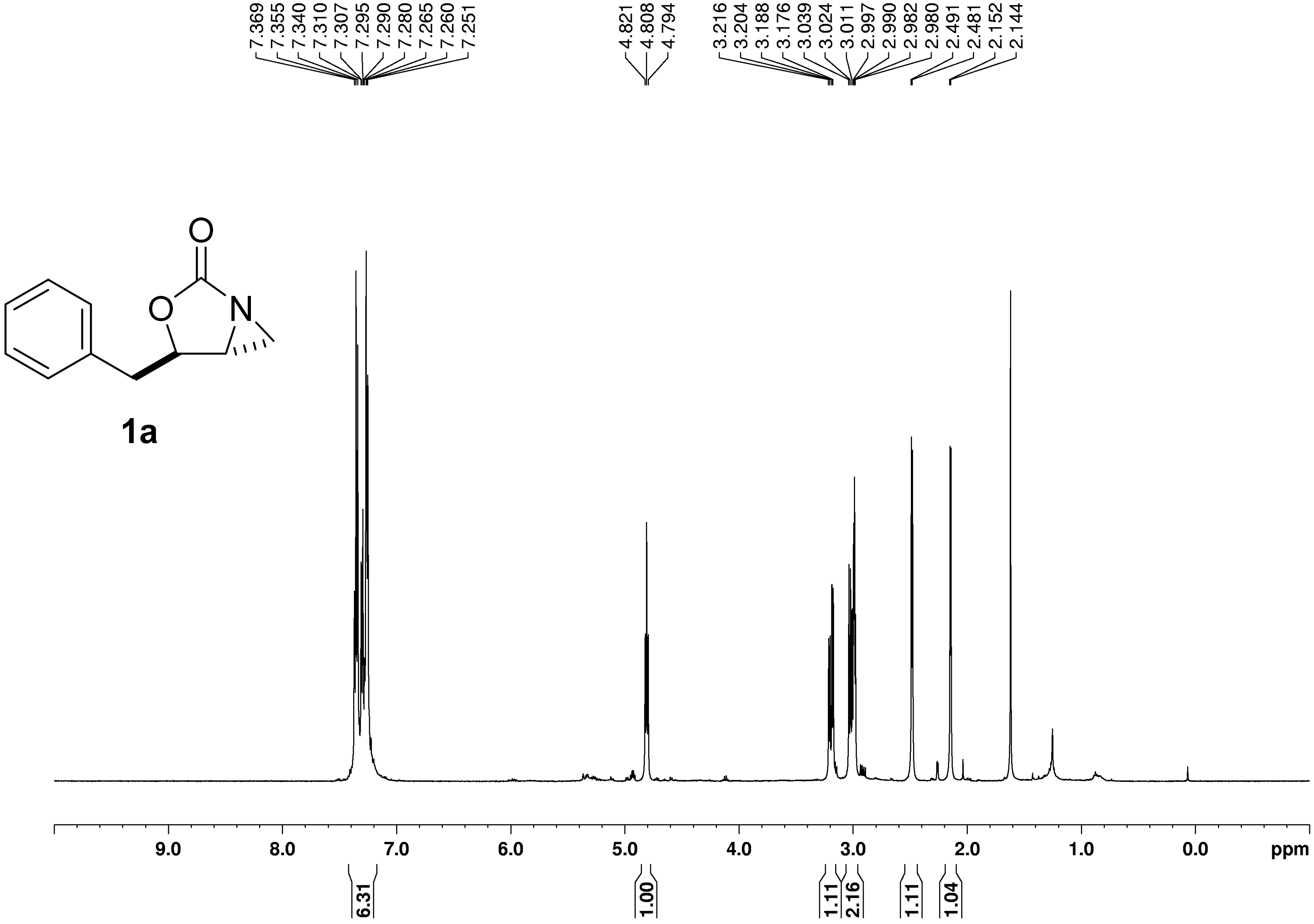

1a

^{1}H NMR of compound **1a** (500 MHz, $CDCl_3$)

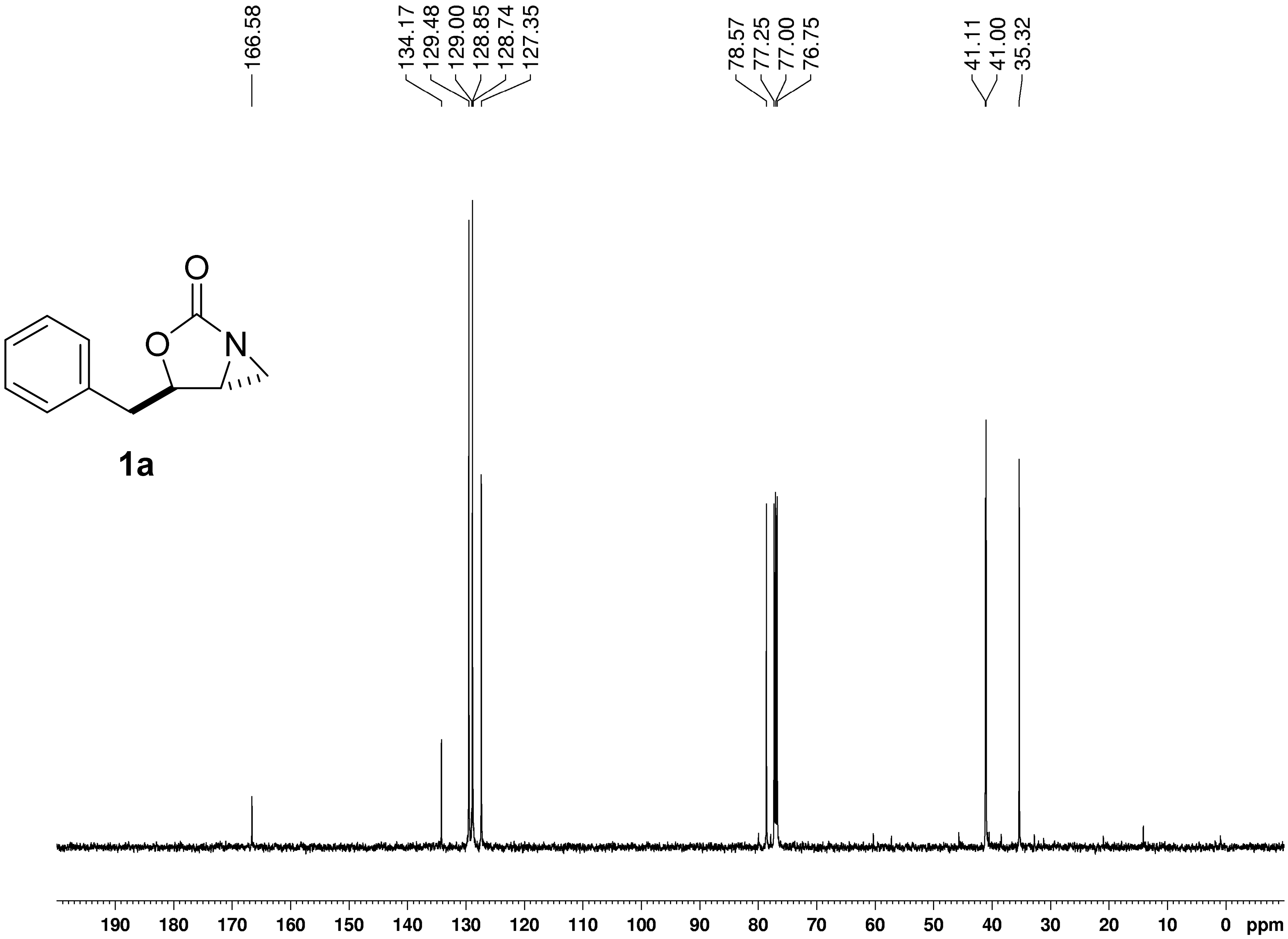

$^{13}C\{^1H\}$ NMR of compound **1a** (126 MHz, $CDCl_3$)

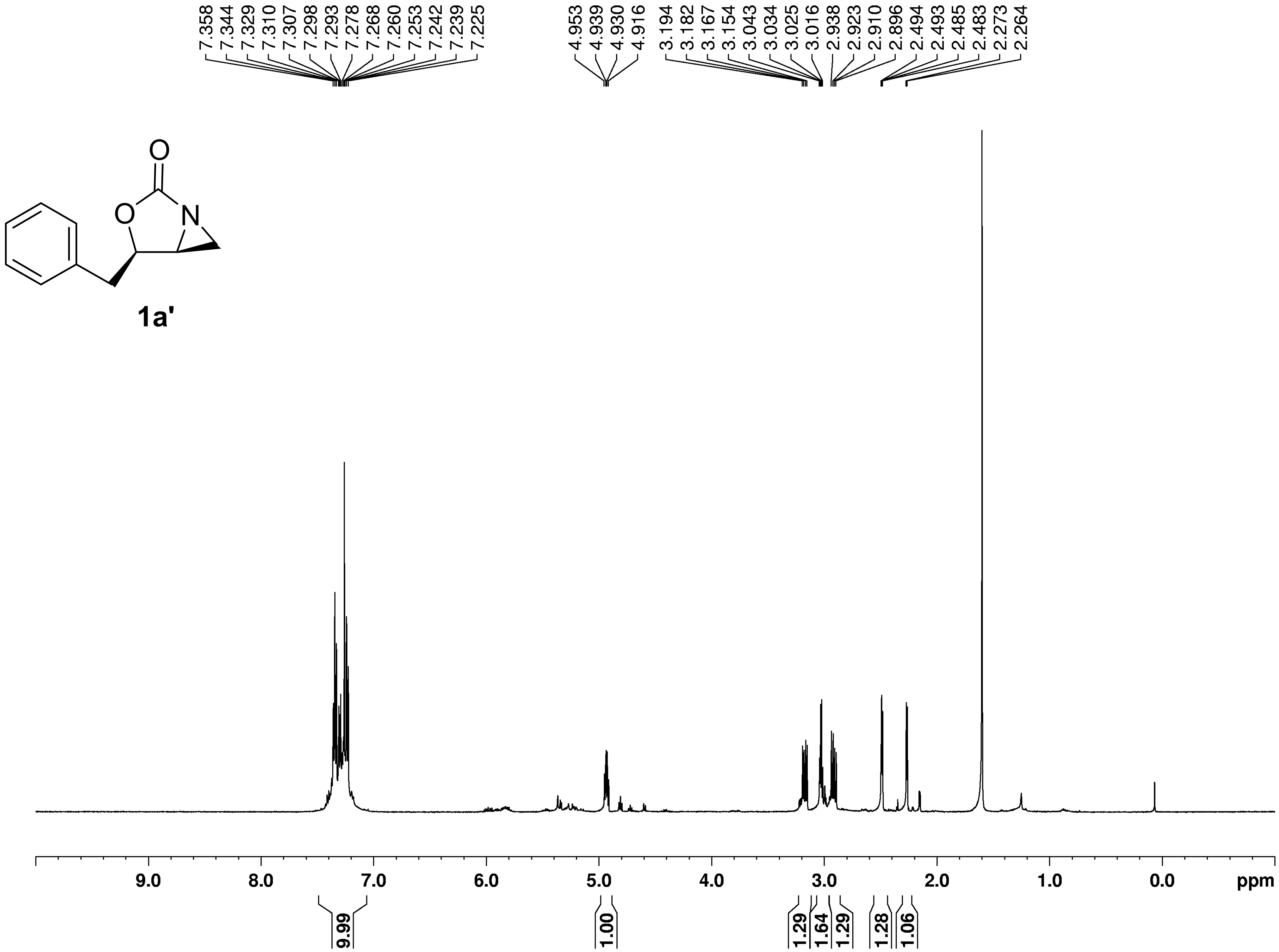

^{1}H NMR of compound **1a’** (500 MHz, $CDCl_3$)

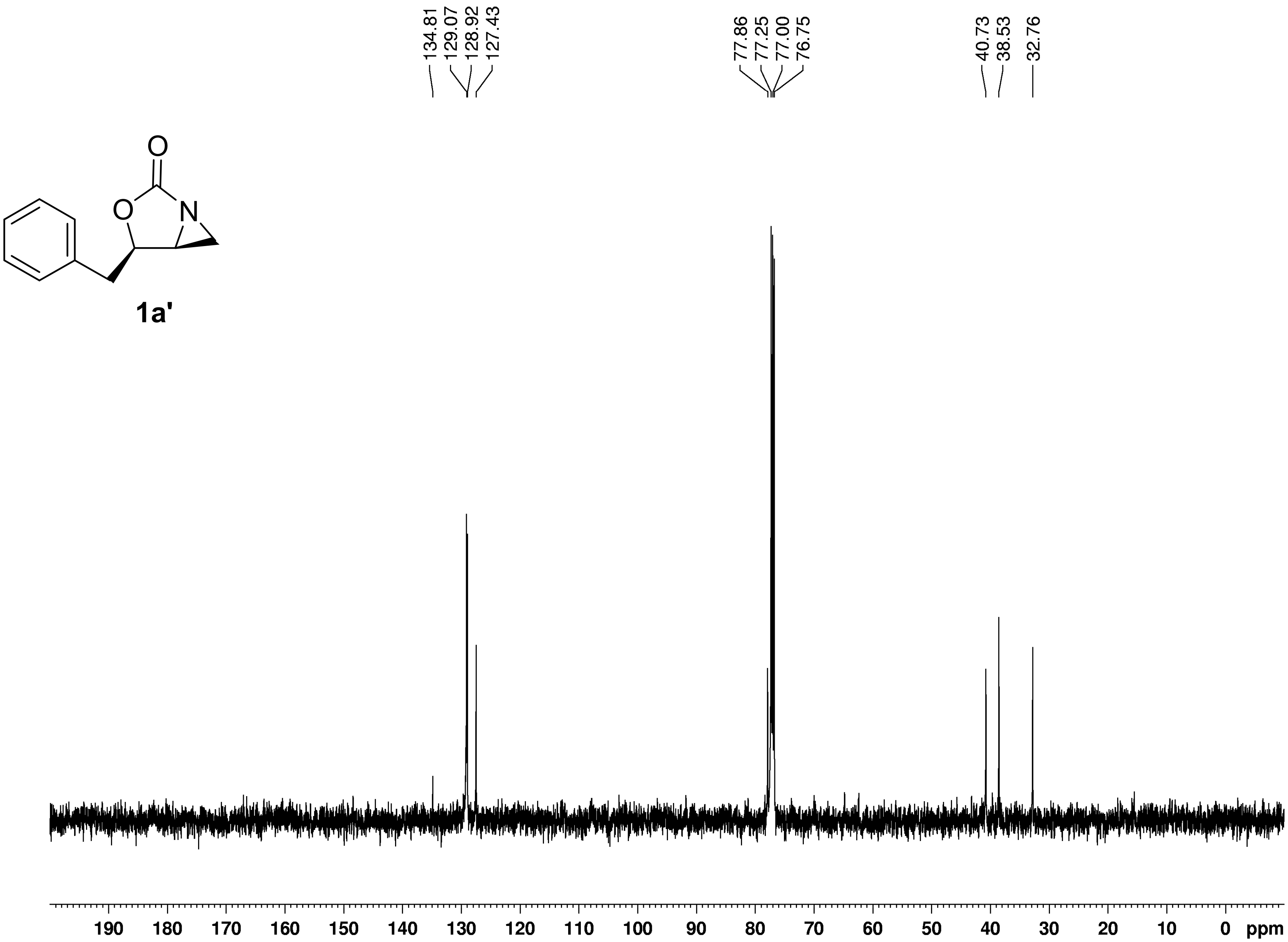

$^{13}C\{^{1}H\}$ NMR of compound **1a’** (126 MHz, $CDCl_3$)

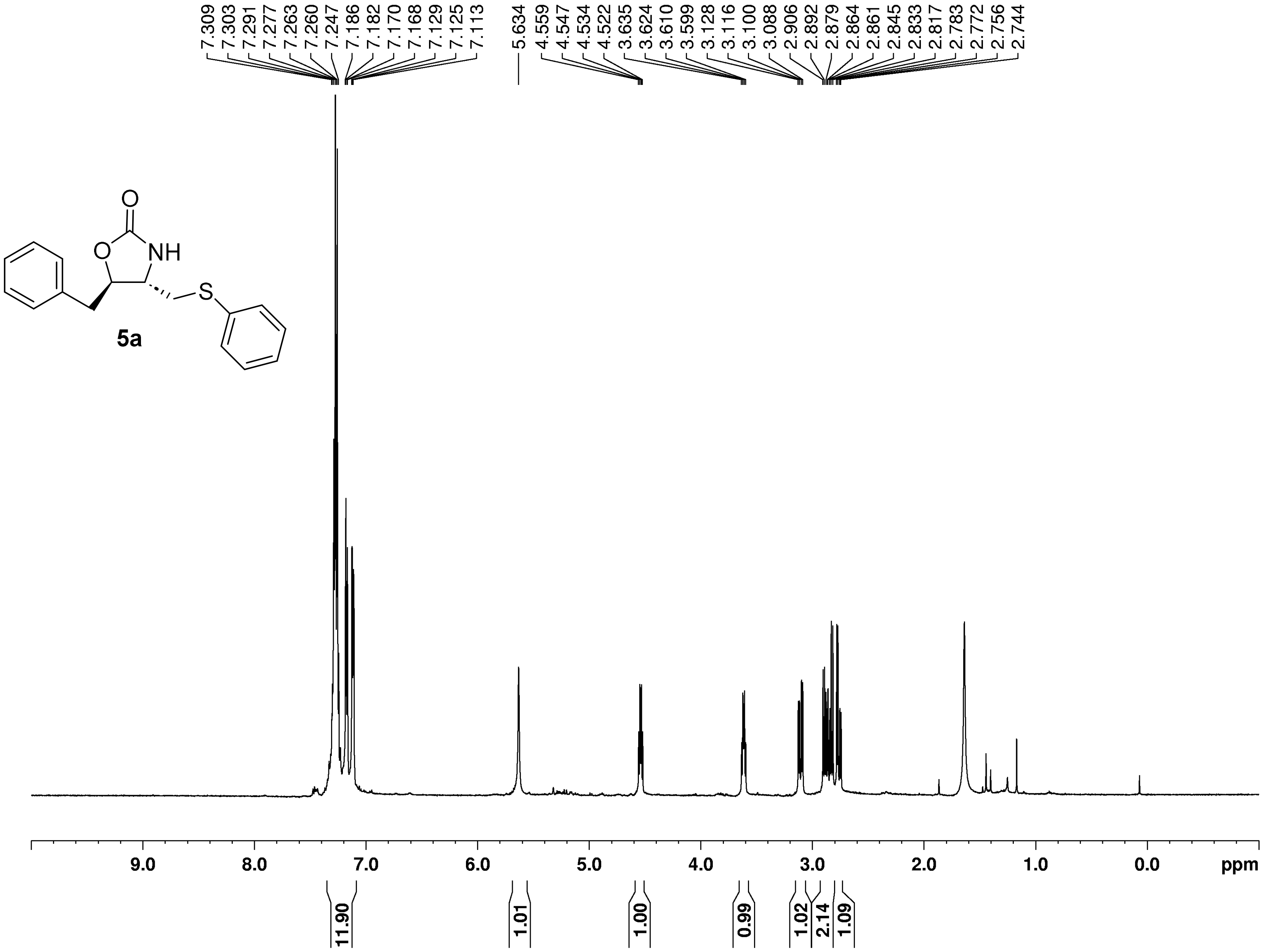

^{1}H NMR of compound **5a** (500 MHz, $CDCl_3$)

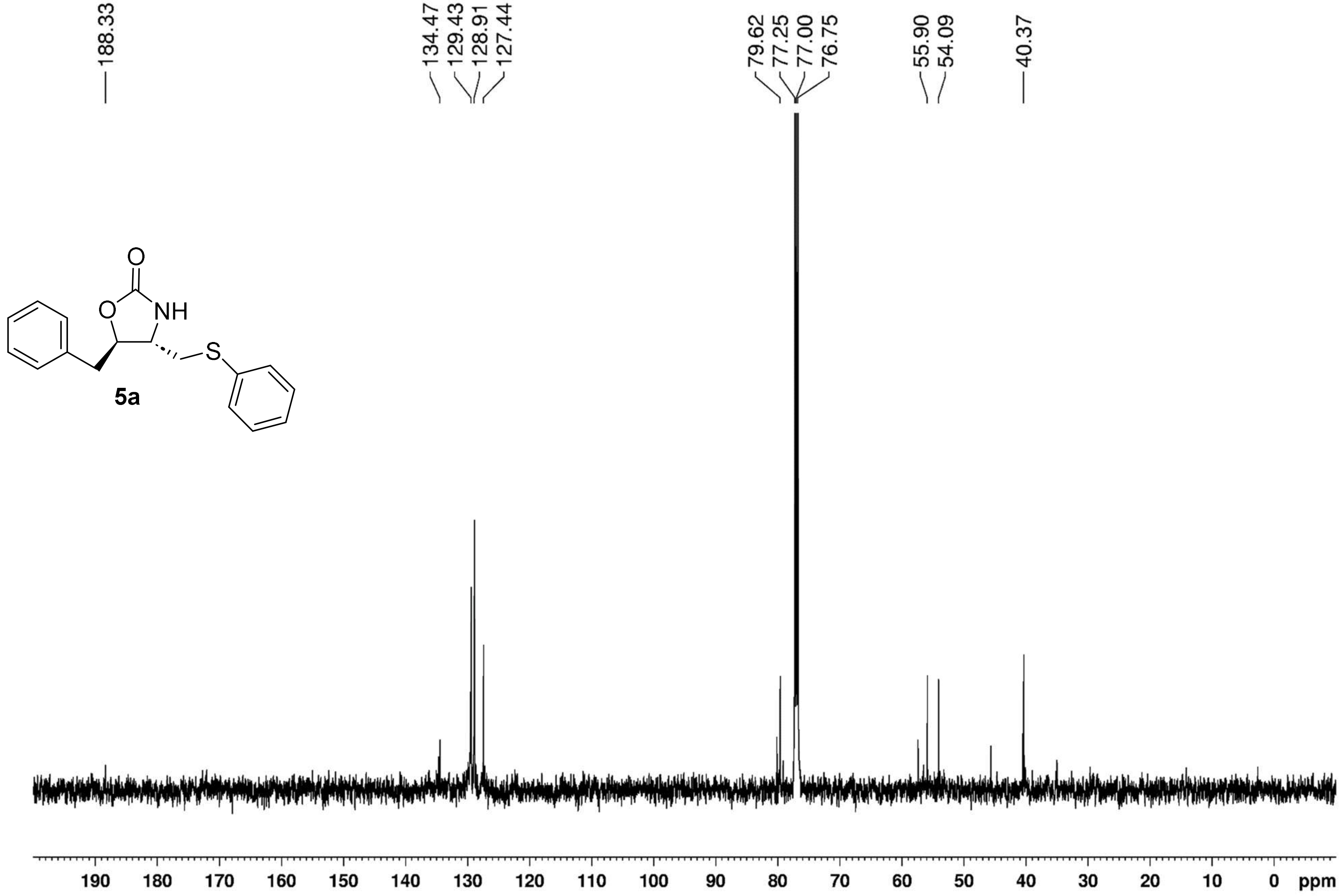

$^{13}C\{^{1}H\}$ NMR of compound (126 MHz, $CDCl_3$)

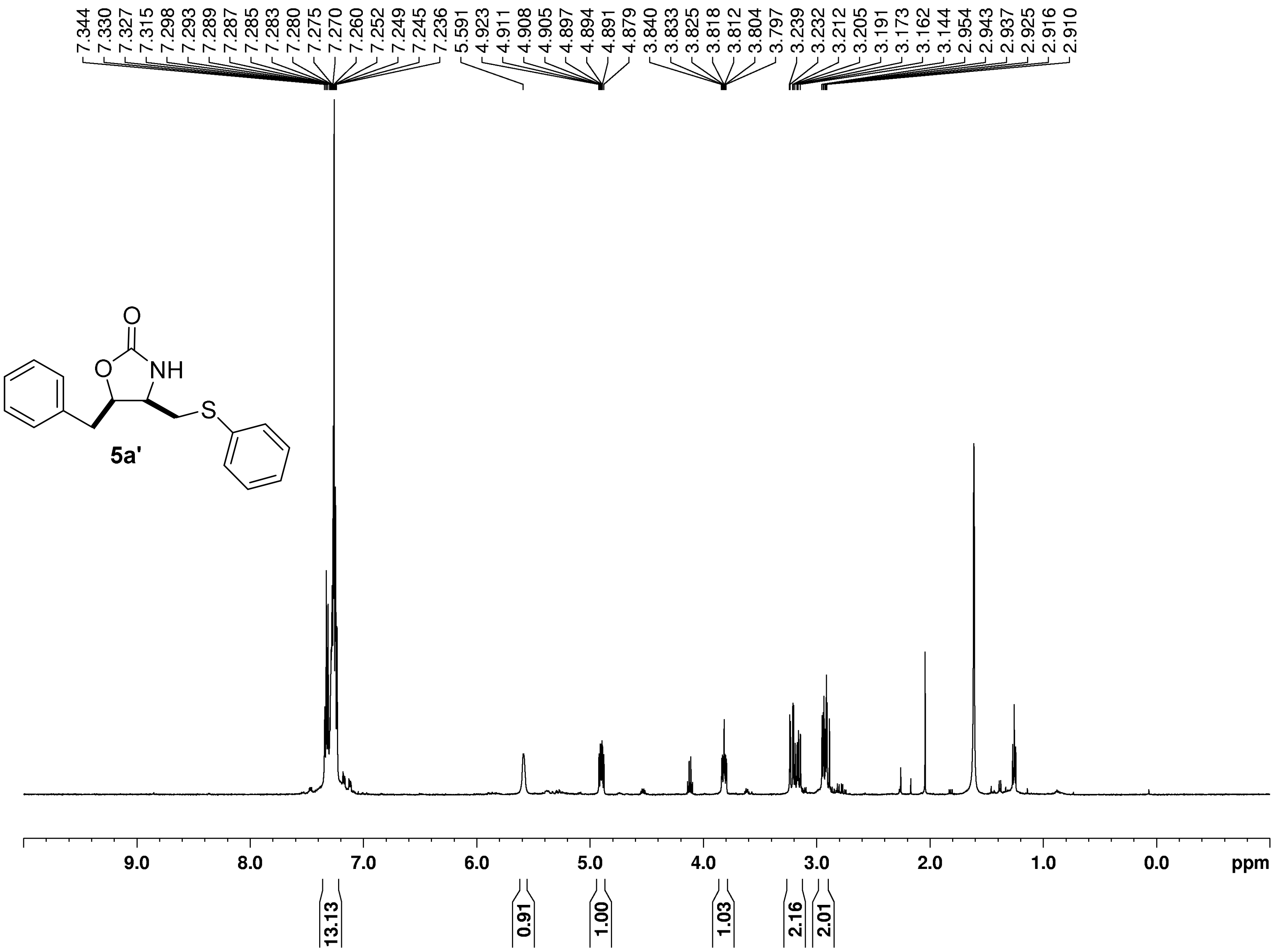

^{1}H NMR of compound of **5a'** (500 MHz, $CDCl_3$)

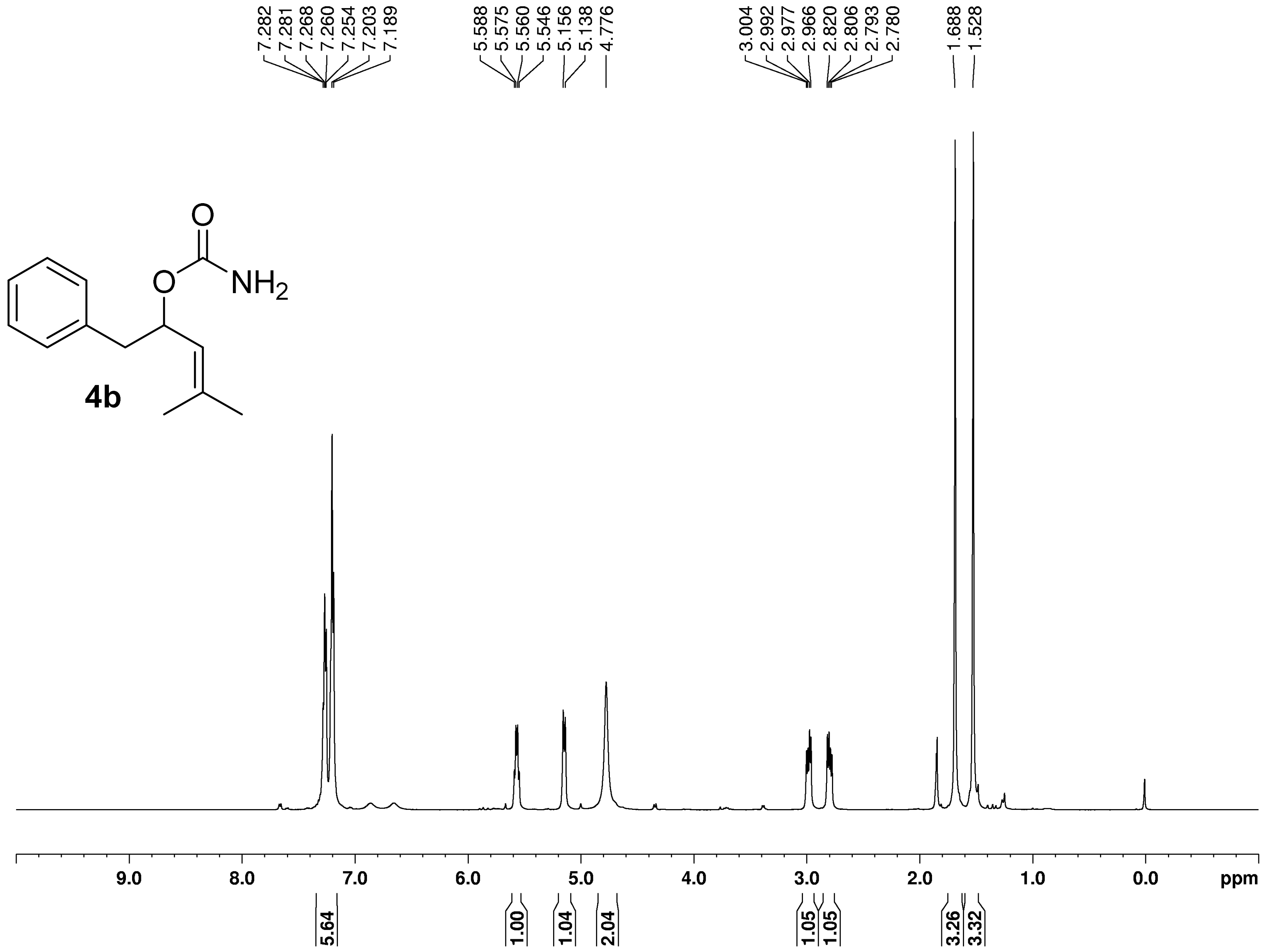

^{1}H NMR of compound **4b** (500 MHz, $CDCl_3$)

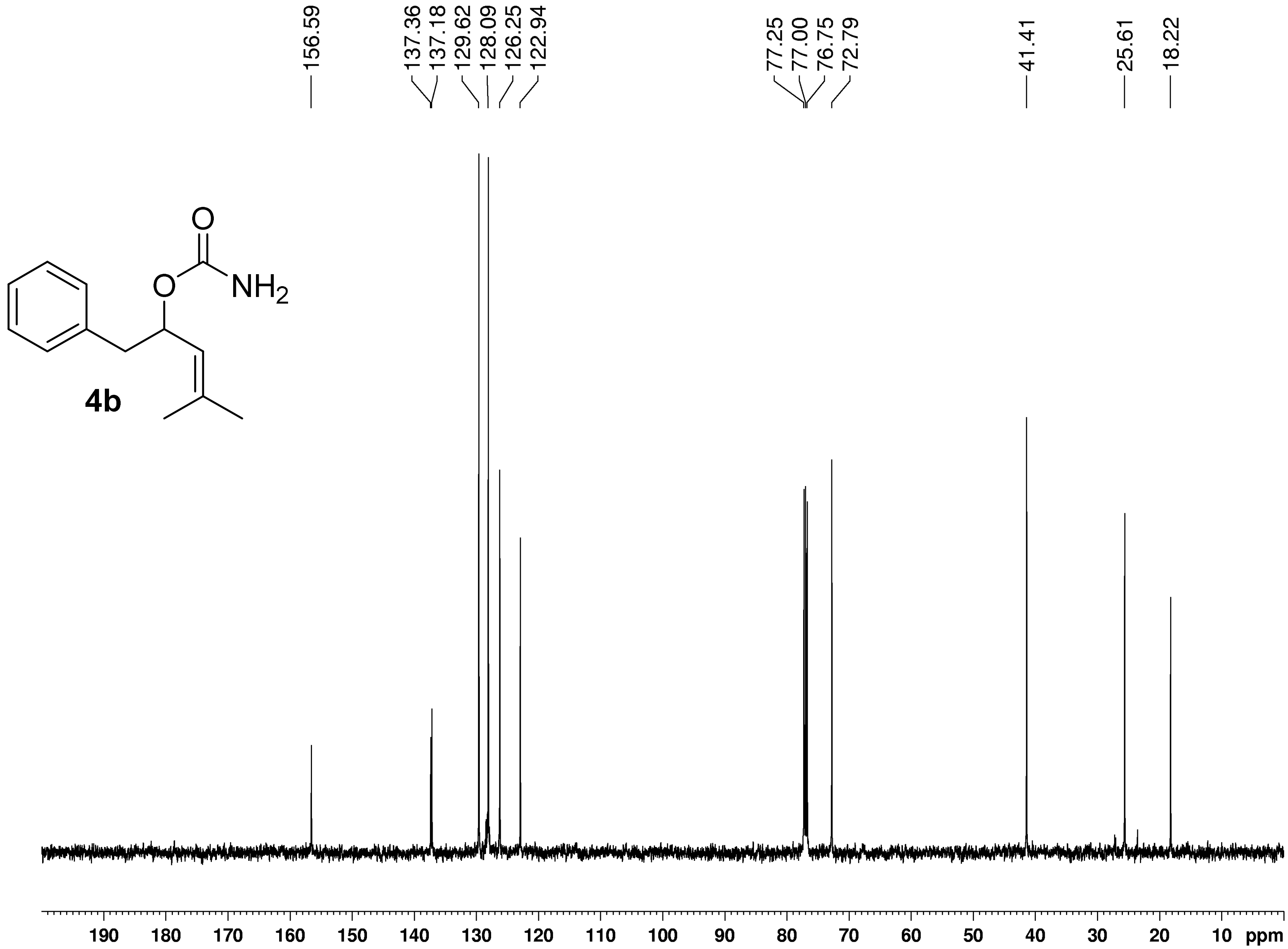

$^{13}C\{^{1}H\}$ NMR of compound **4b** (126 MHz, $CDCl_3$)

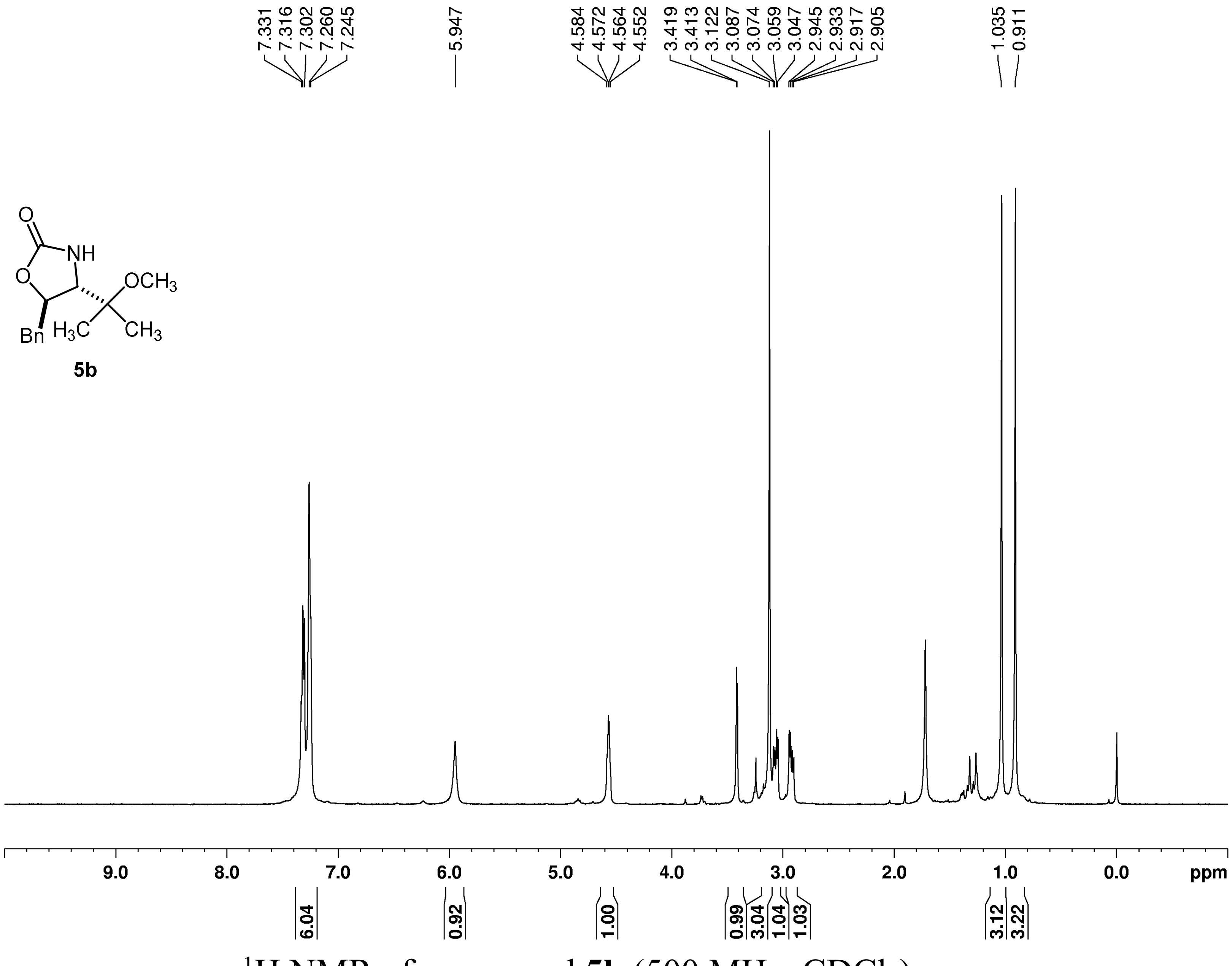

^{1}H NMR of compound **5b** (500 MHz, $CDCl_3$)

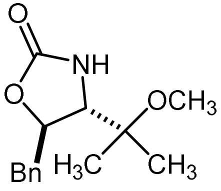

5b

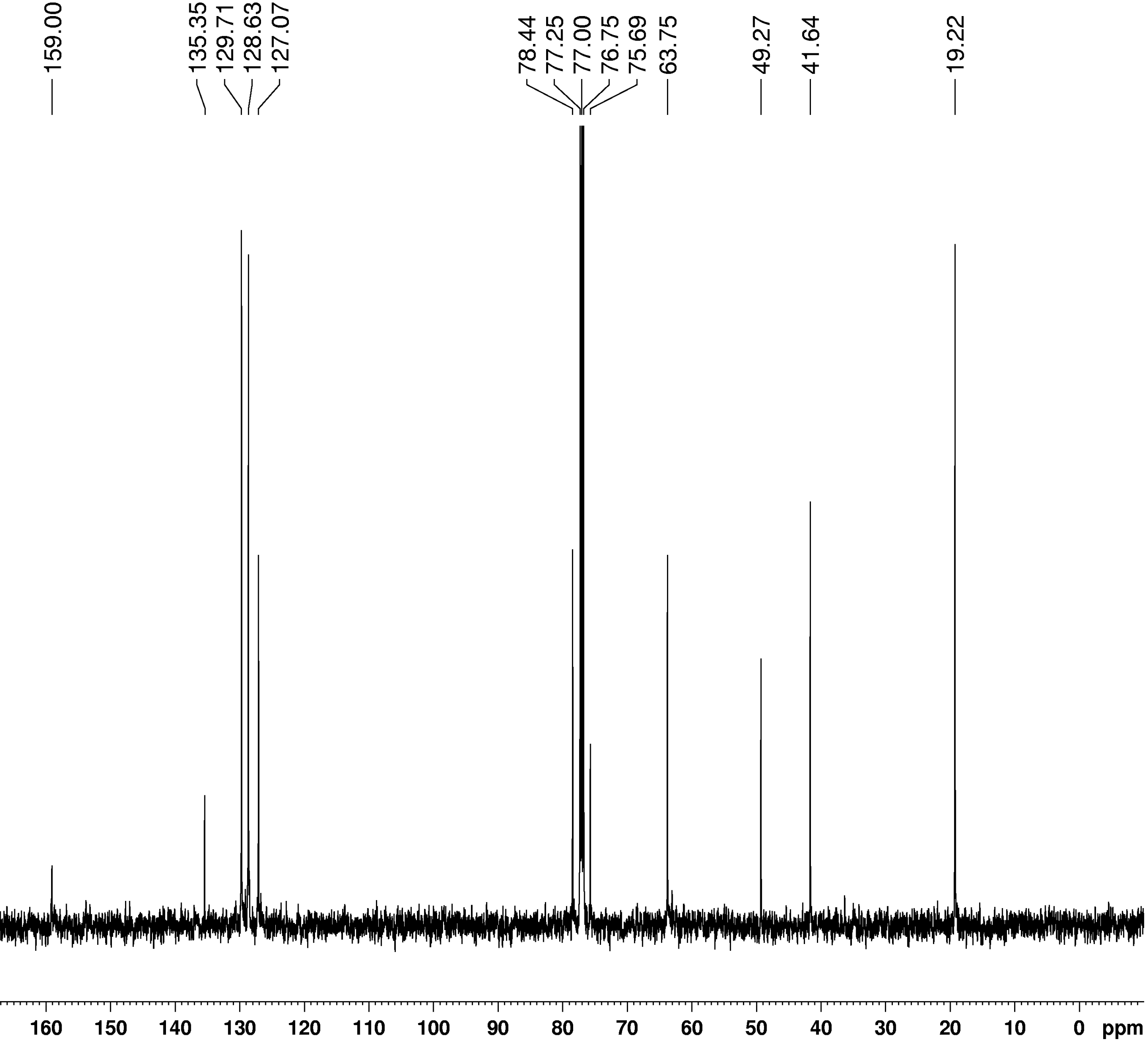

$^{13}C\{^1H\}$ NMR of compound **5b** (126 MHz, $CDCl_3$)

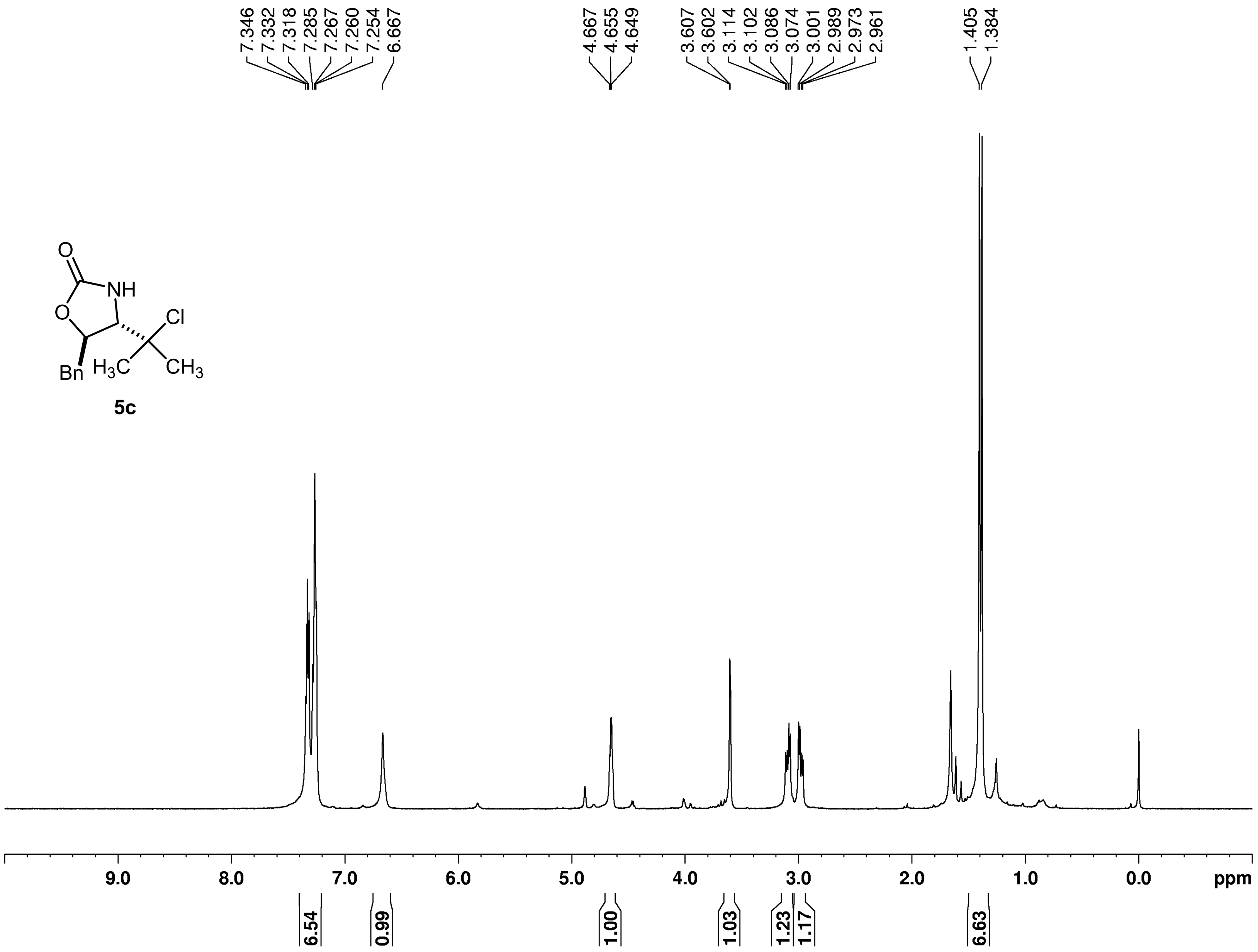

^{1}H NMR of compound **5c** (500 MHz, $CDCl_3$)

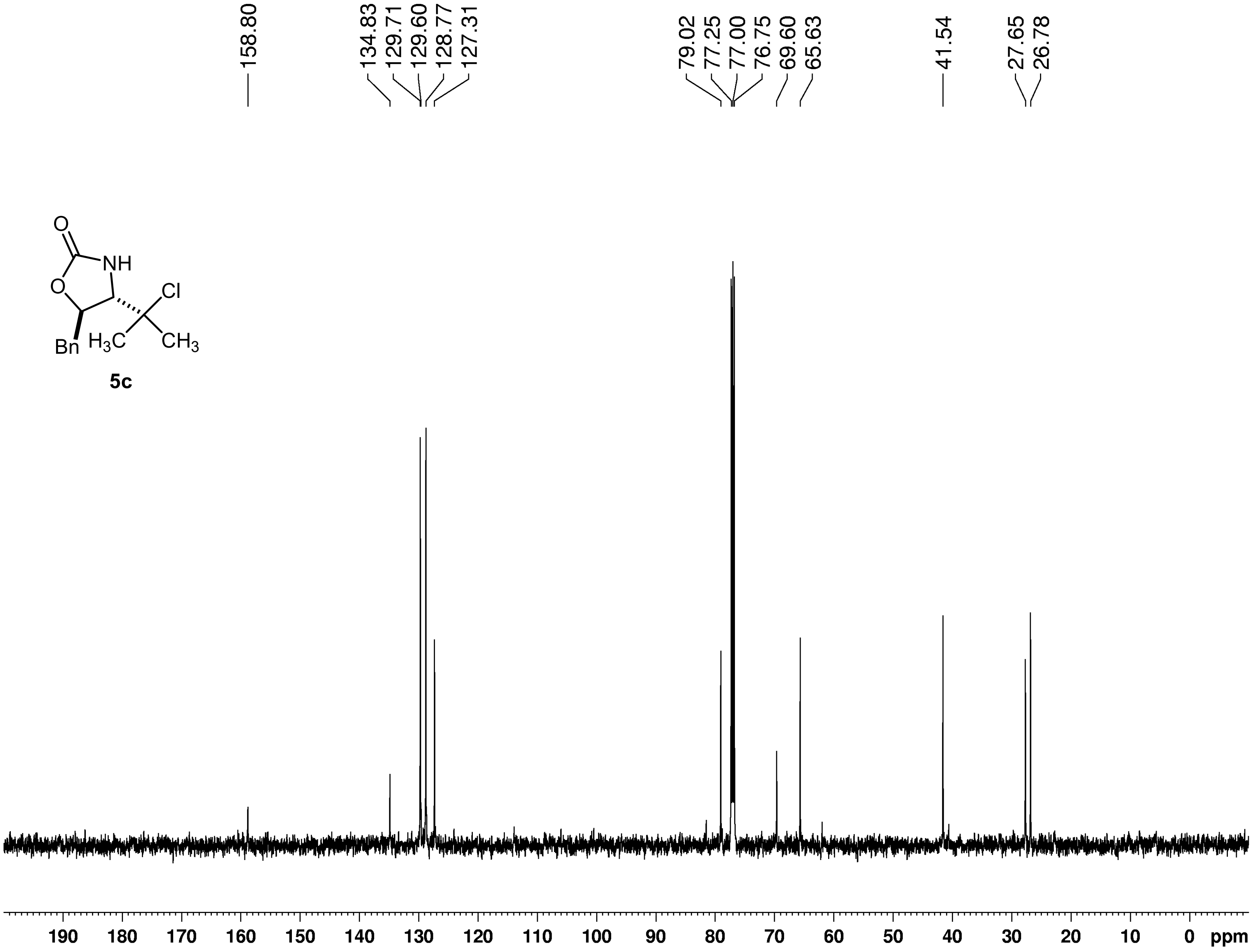

$^{13}C\{^{1}H\}$ NMR of compound **5c** (126 MHz, $CDCl_3$)

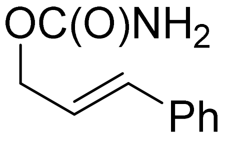

4d

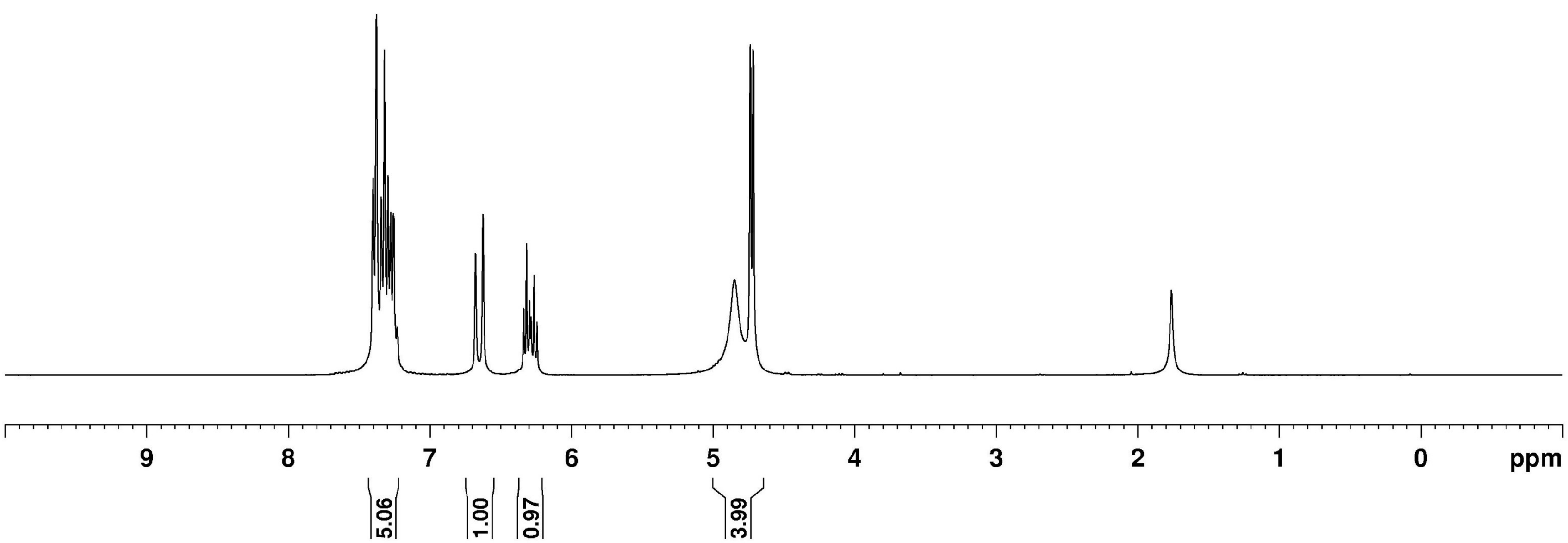

^{1}H NMR of compound **4d** (300 MHz, $CDCl_3$)

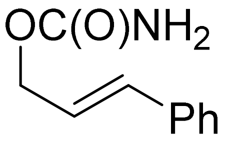

4d

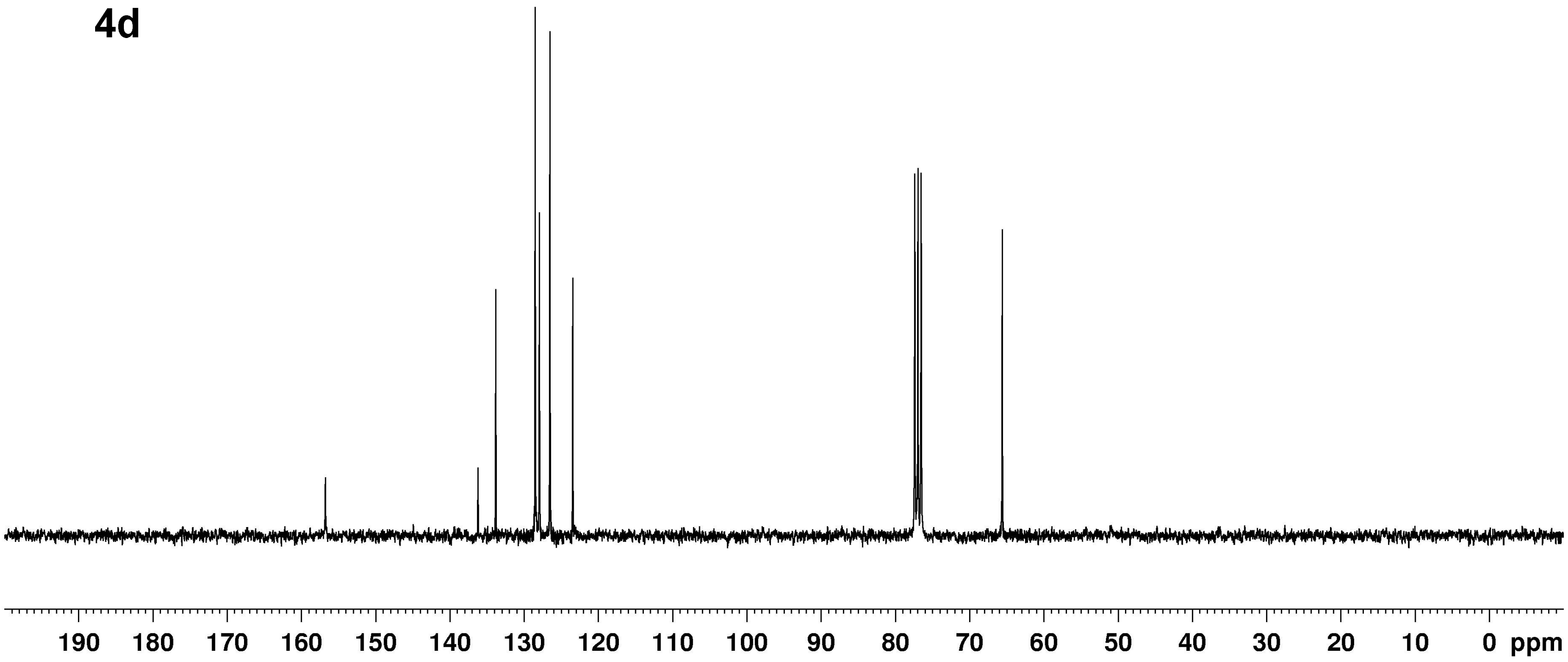

$^{13}C\{^{1}H\}$ NMR of compound **4d** (75 MHz, $CDCl_3$)

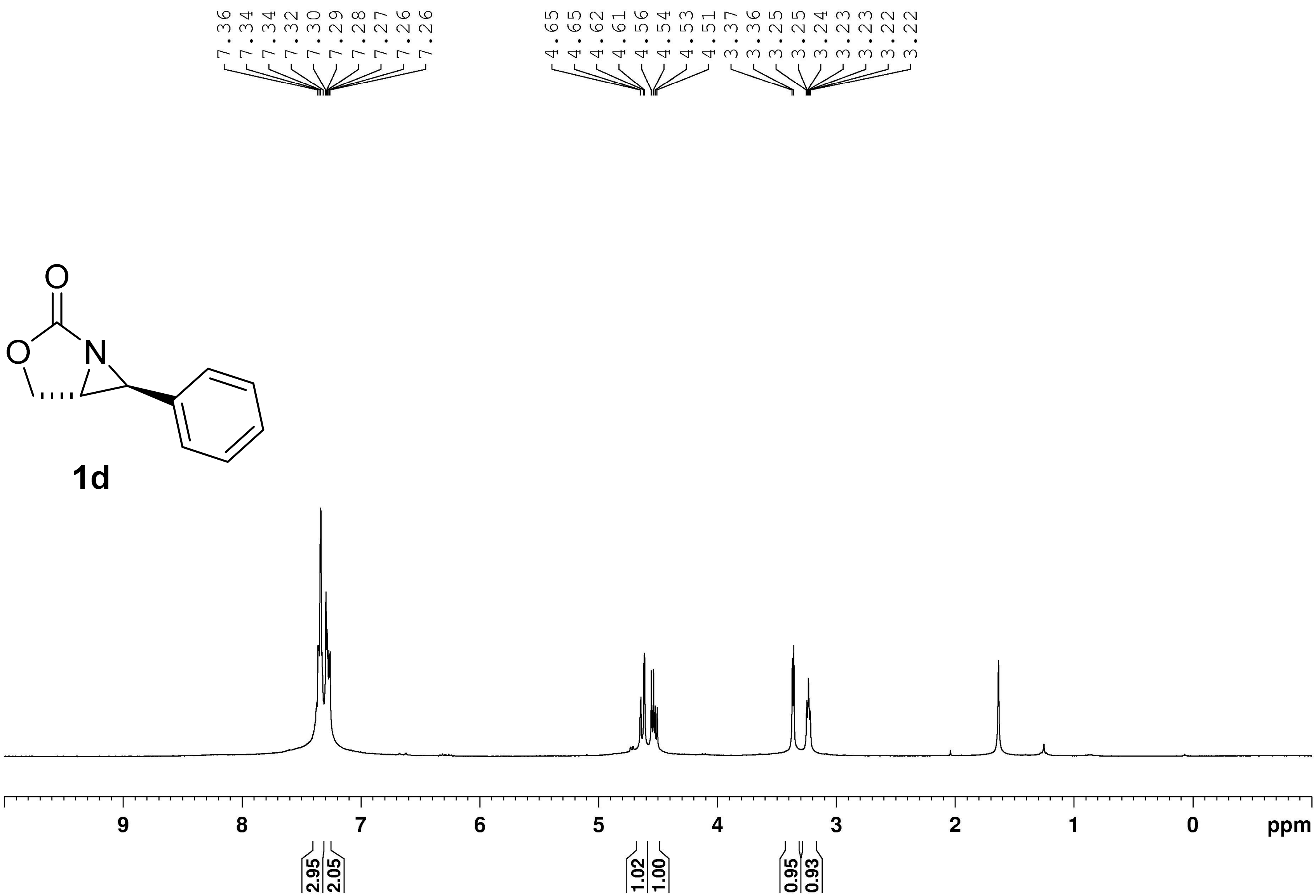

^{1}H NMR of compound **1d** (300 MHz, $CDCl_3$)

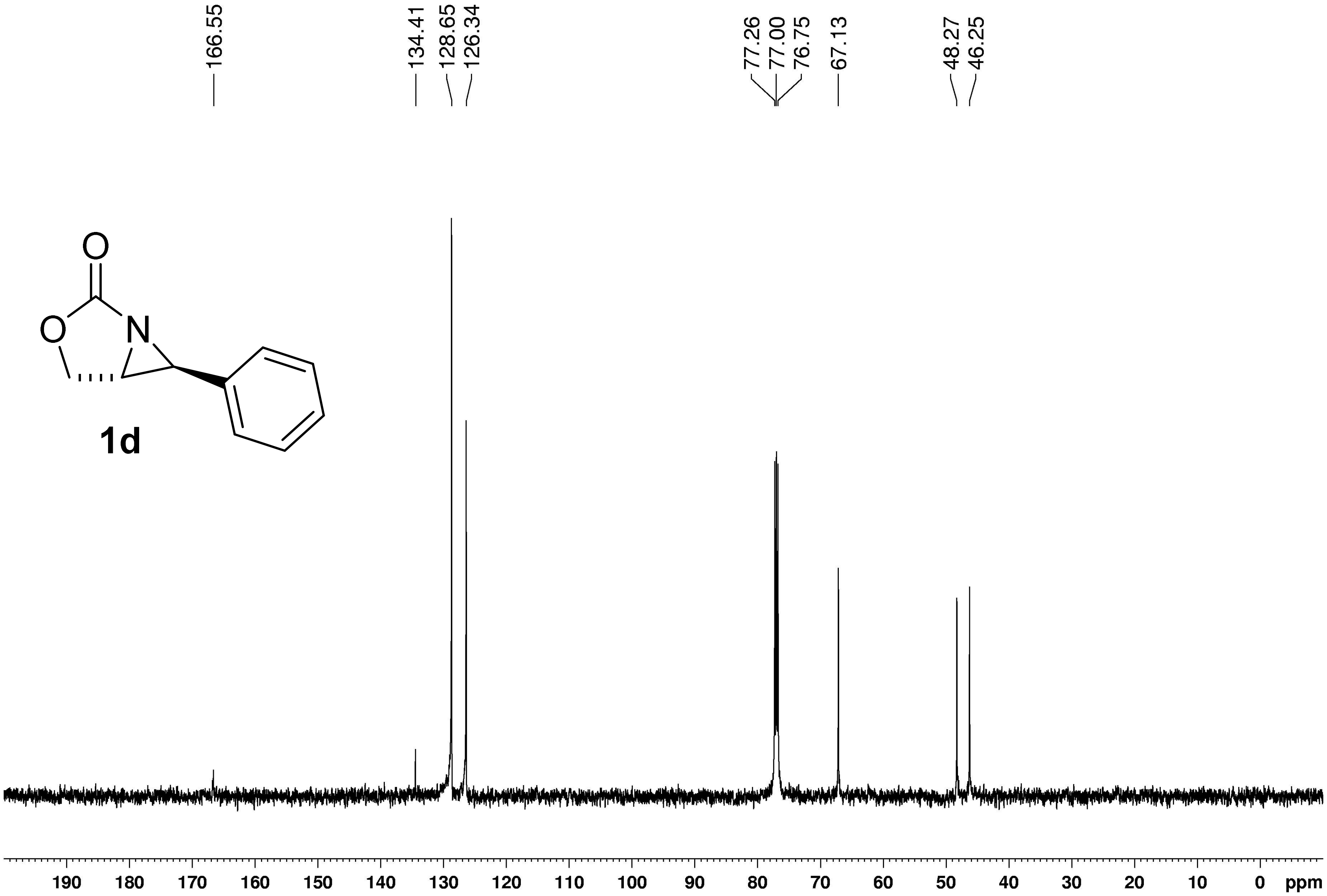

$^{13}C\{^{1}H\}$ NMRNMR of compound **1d** (126 MHz, $CDCl_3$)

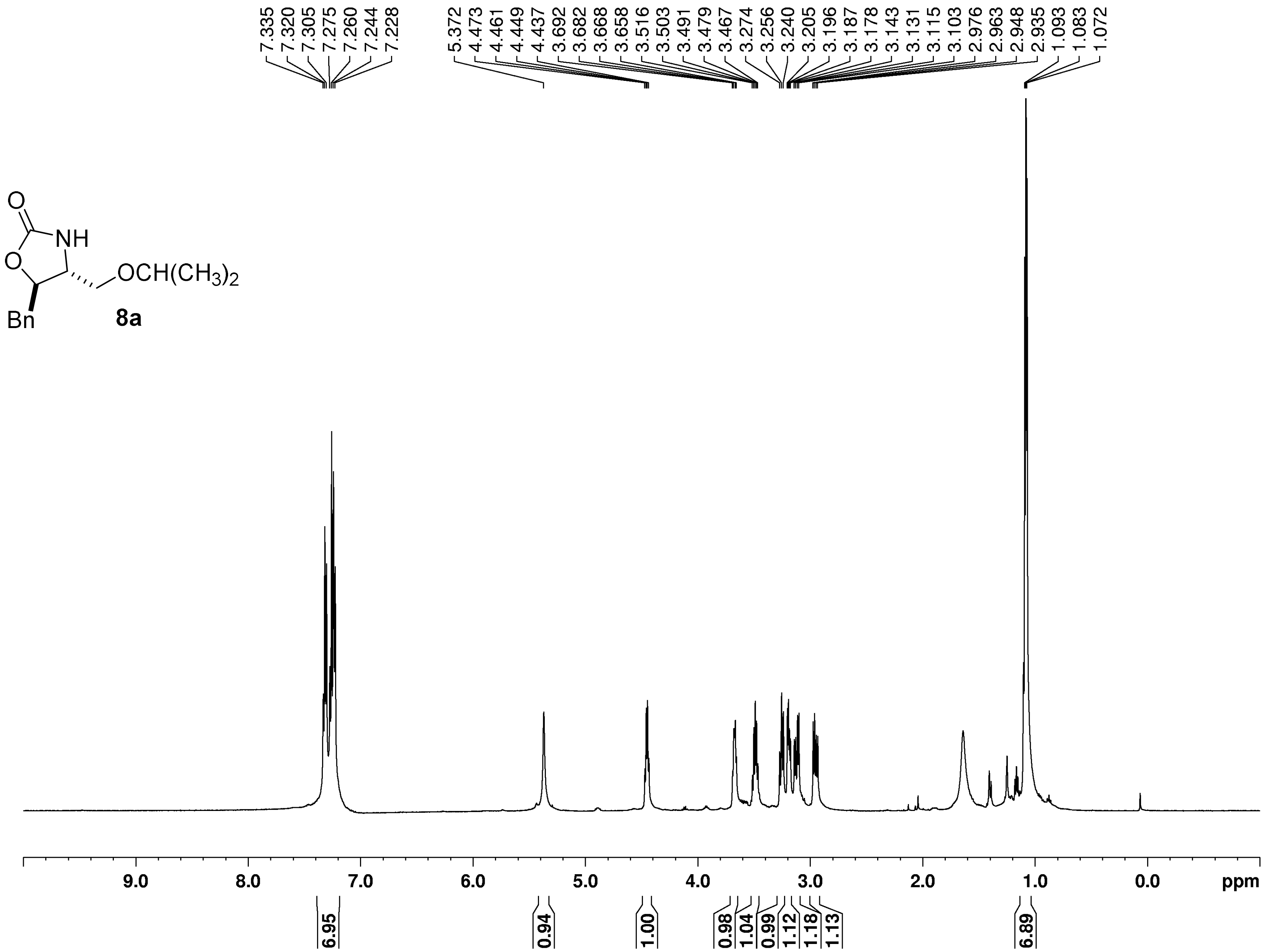

^{1}H NMR of compound **8a** (500 MHz, $CDCl_3$)

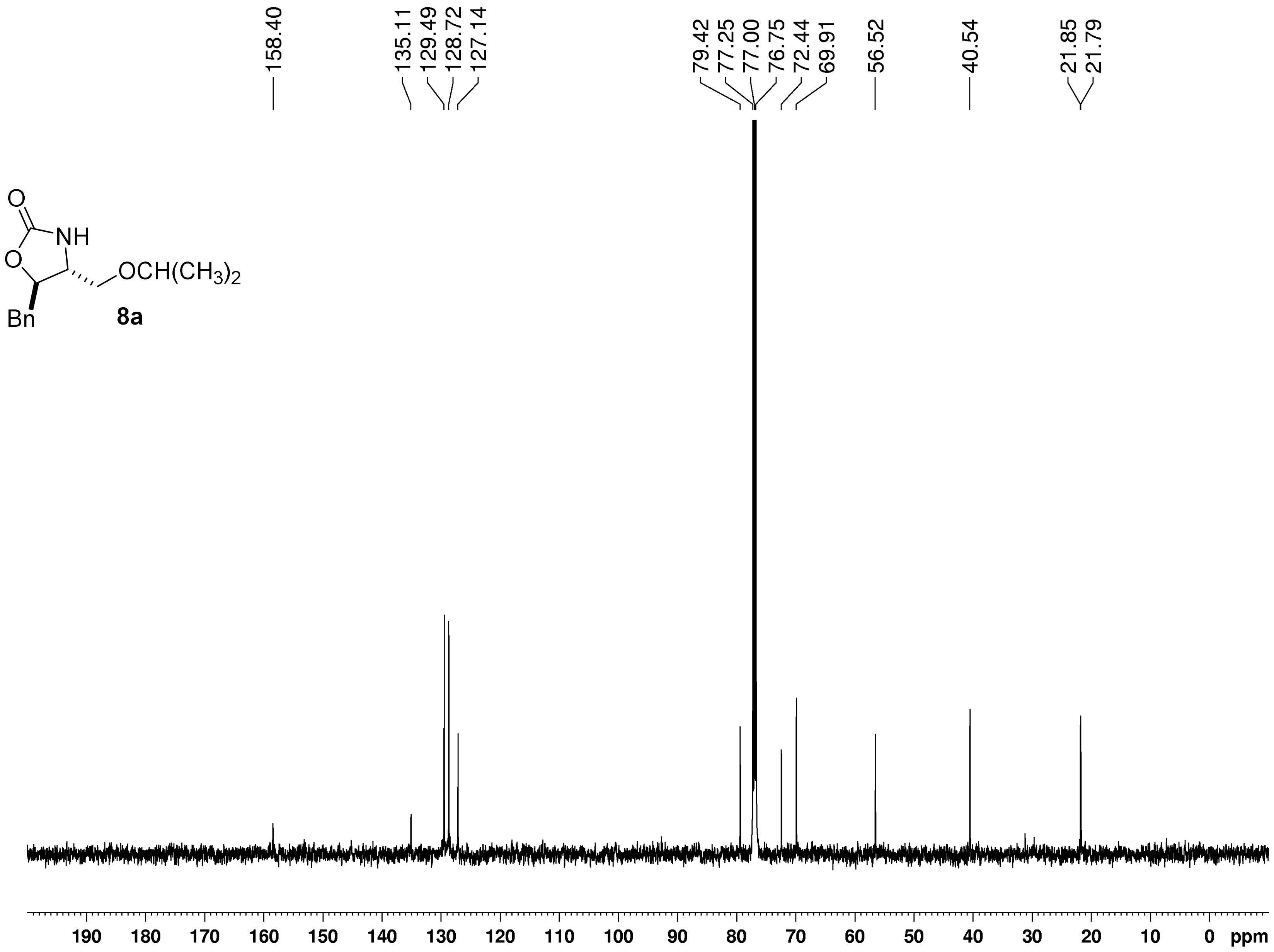

$^{13}C\{^{1}H\}$ NMR of compound **8a** (126 MHz, $CDCl_3$)

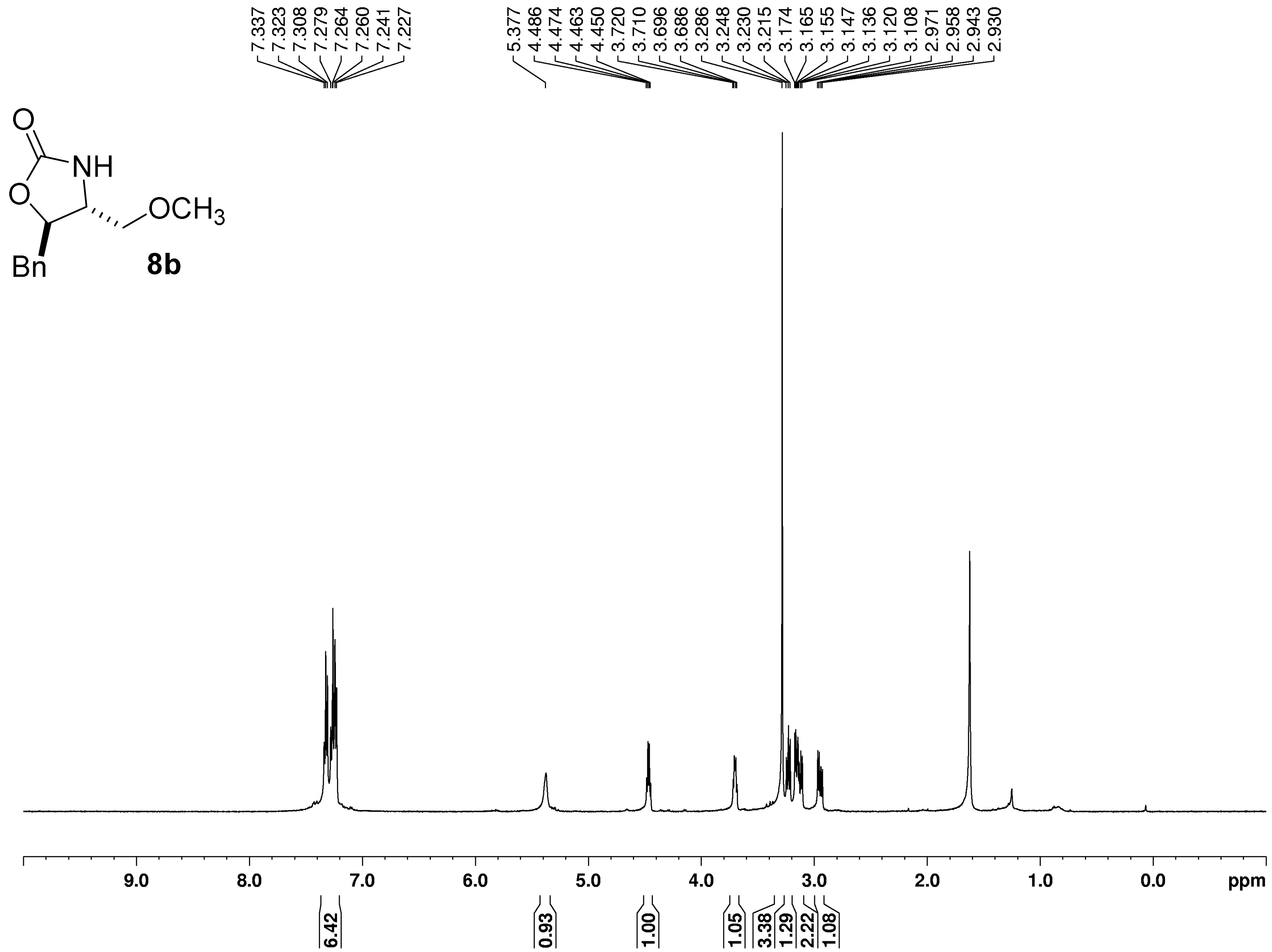

^{1}H NMR of compound **8b** (500 MHz, $CDCl_3$)

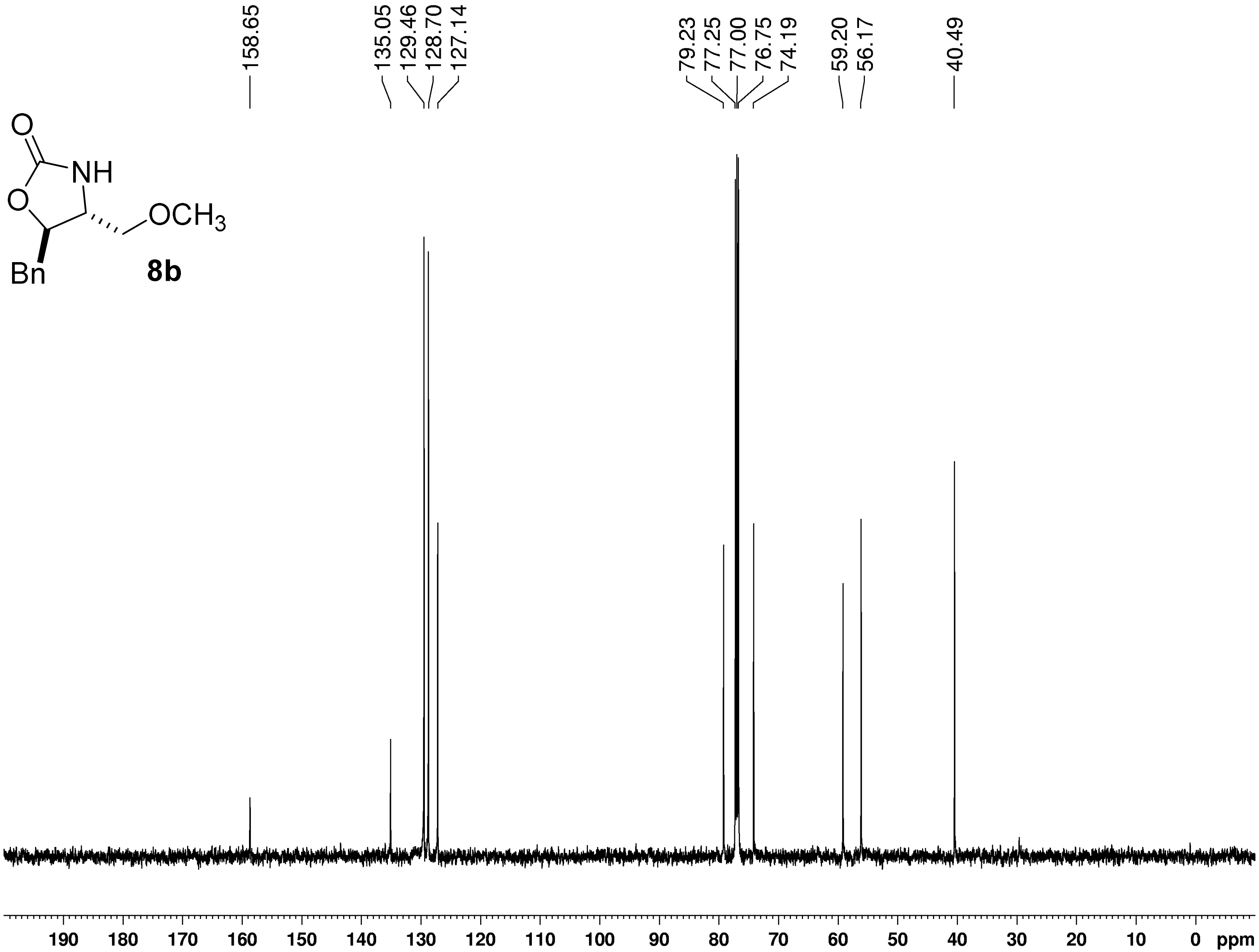

$^{13}C\{^{1}H\}$ NMR of compound **8b** (126 MHz, $CDCl_3$)

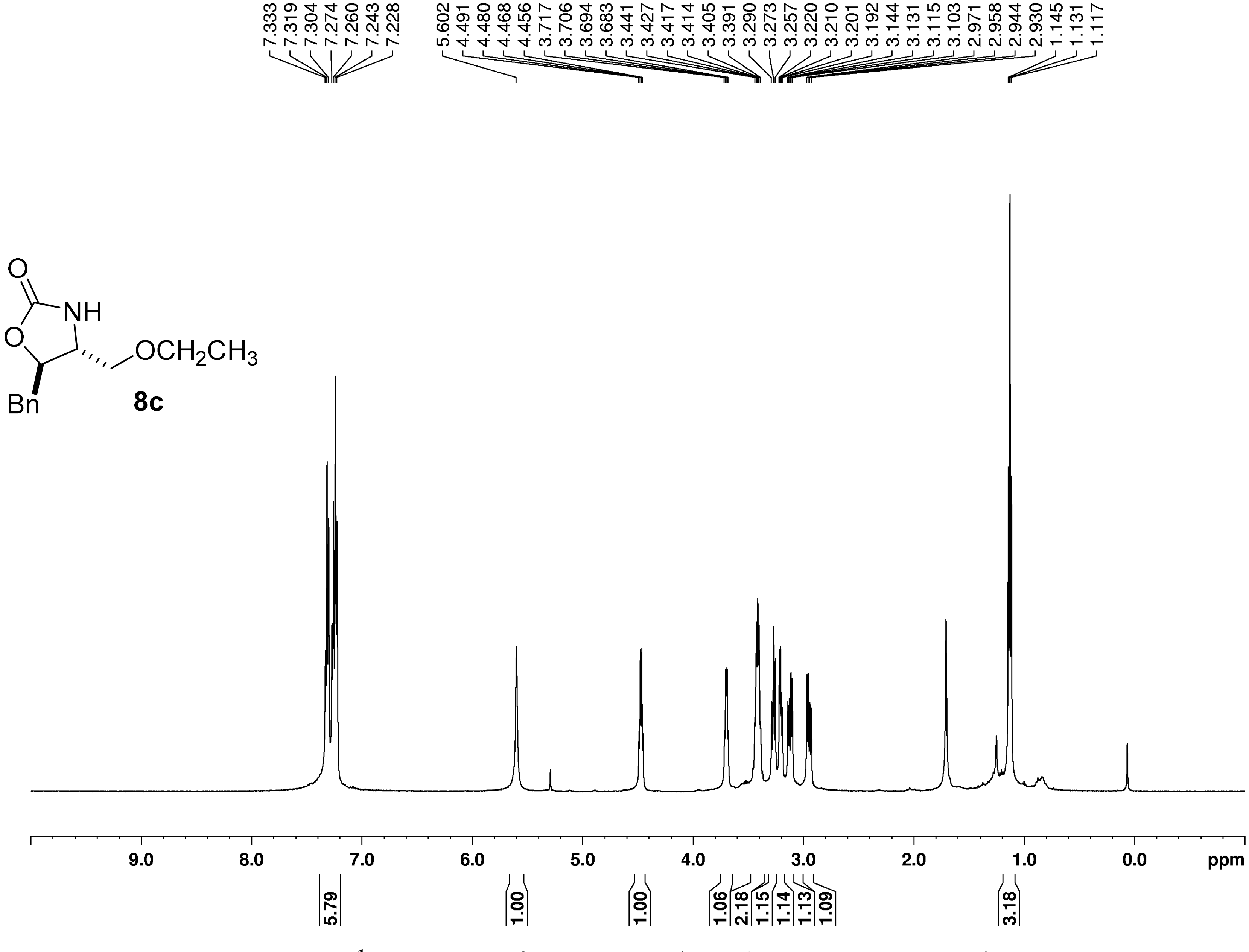

^{1}H NMR of compound **8c** (500 MHz, $CDCl_3$)

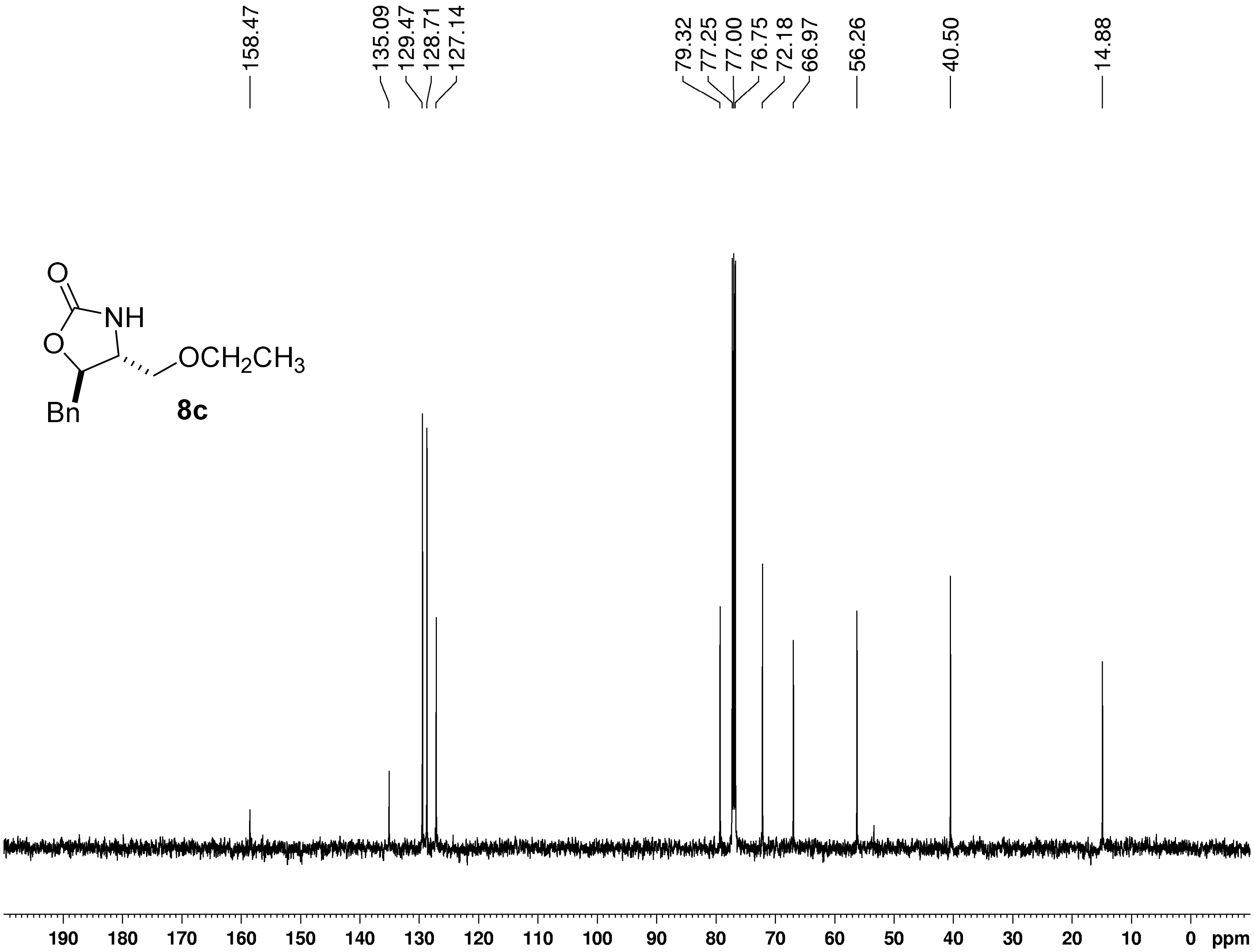

^{13}C {^{1}H} NMR of compound **8c** (126 MHz, $CDCl_3$)

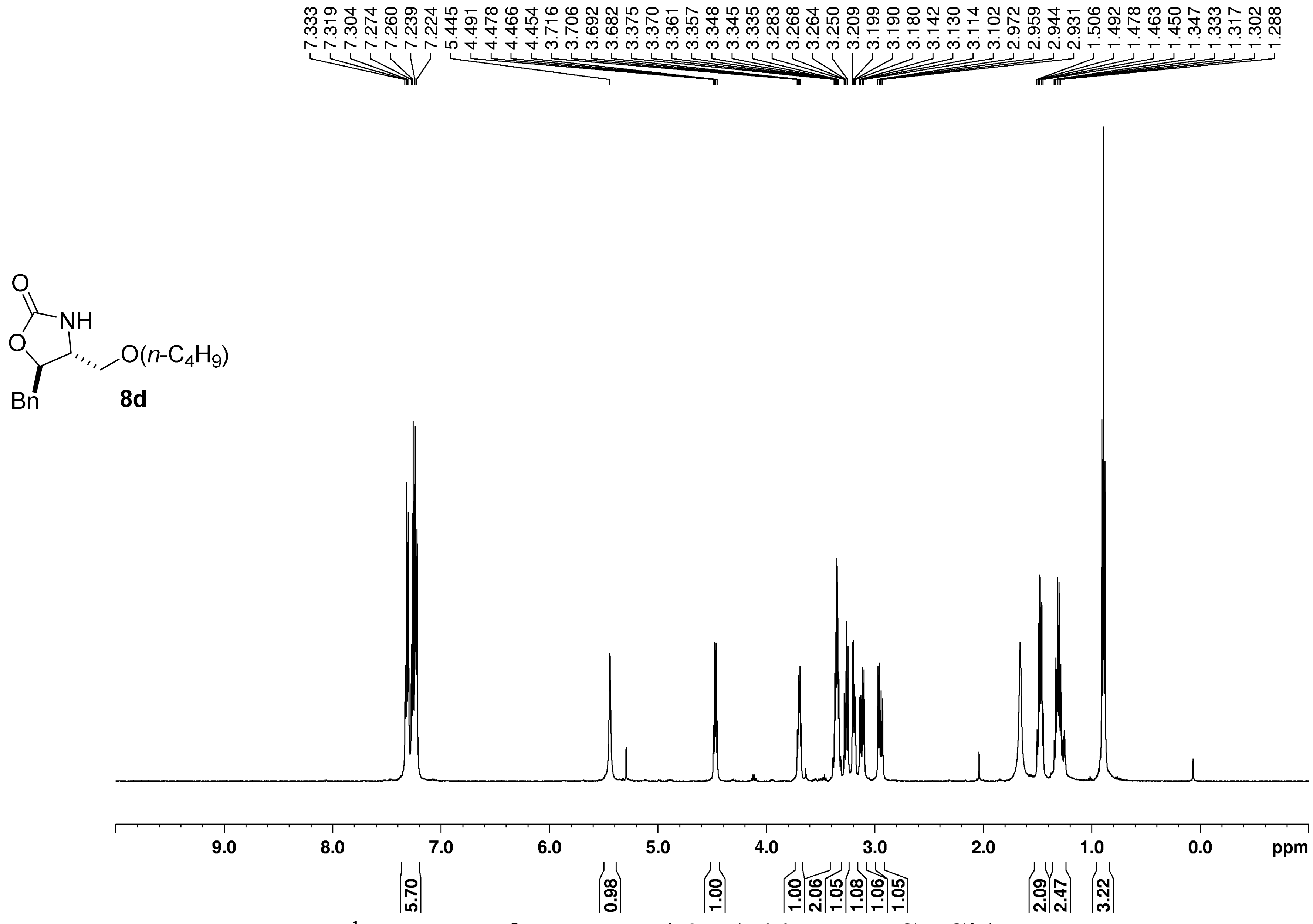

8d

^1H NMR of compound **8d** (500 MHz, $CDCl_3$)

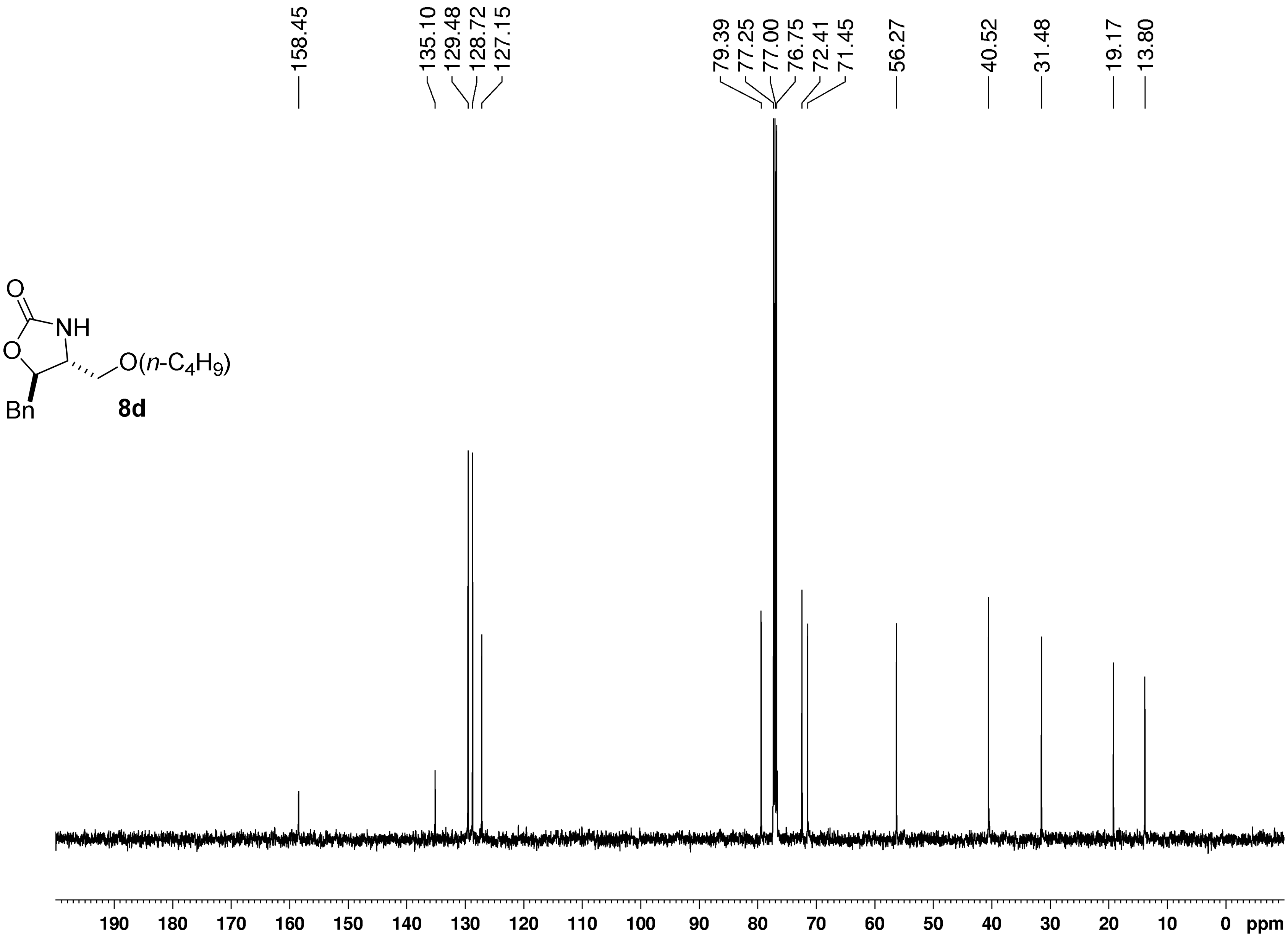

$^{13}C\{^{1}H\}$ NMR of compound **8d** (126 MHz, $CDCl_3$)

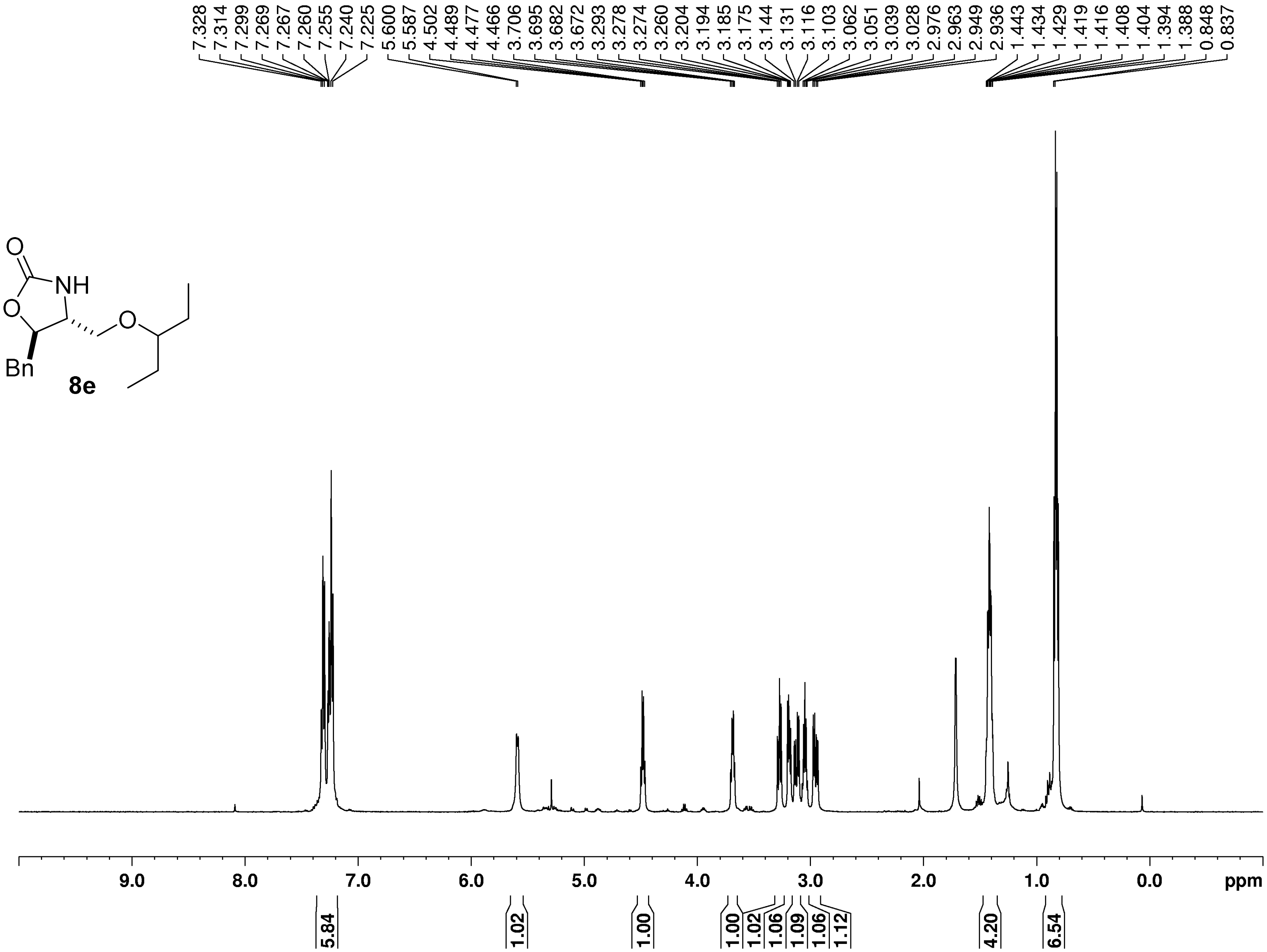

8e

^{1}H NMR of compound **8e** (500 MHz, $CDCl_3$)

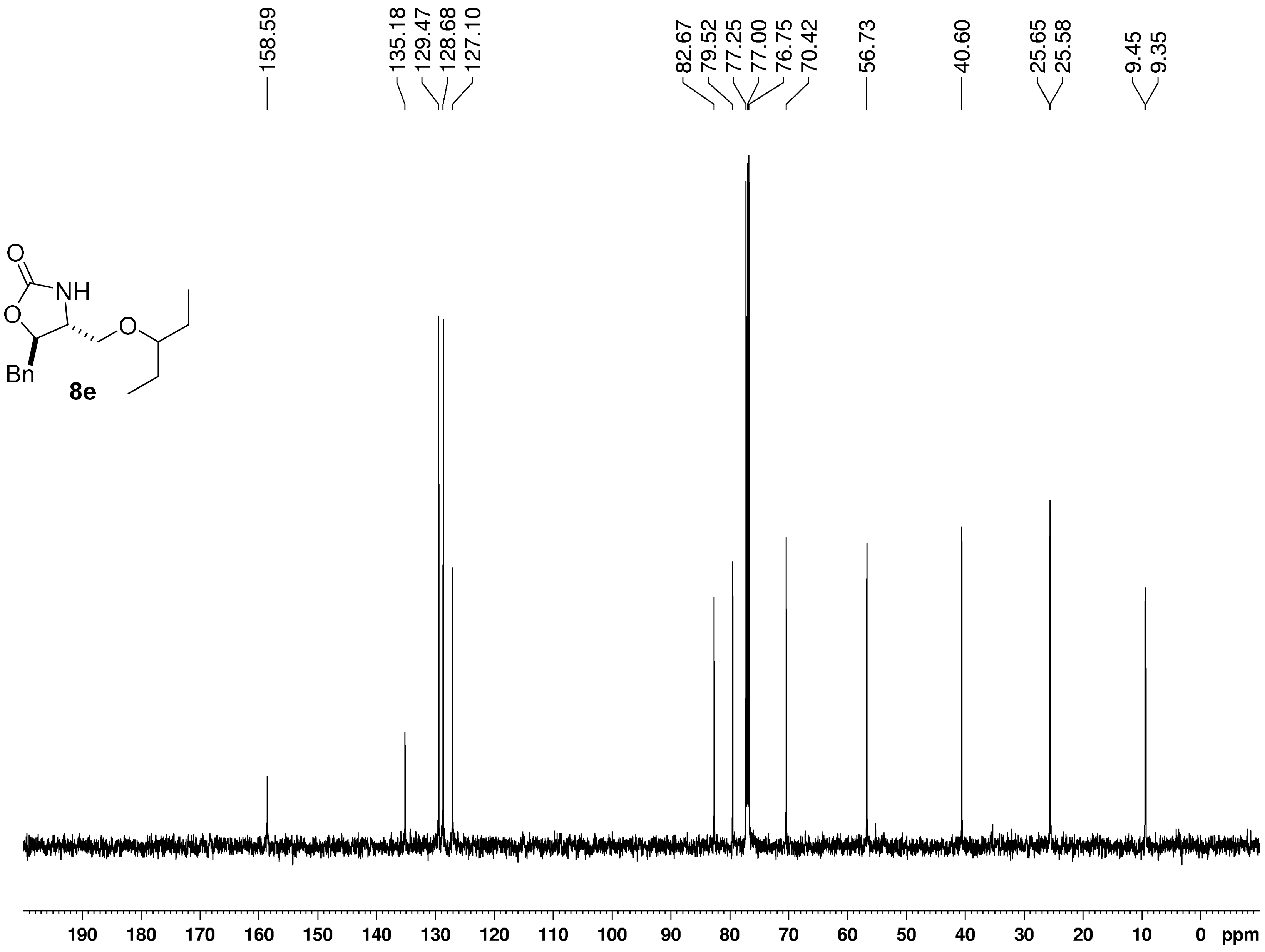

^{13}C {^{1}H} NMR of compound **8e** (126 MHz, $CDCl_3$)

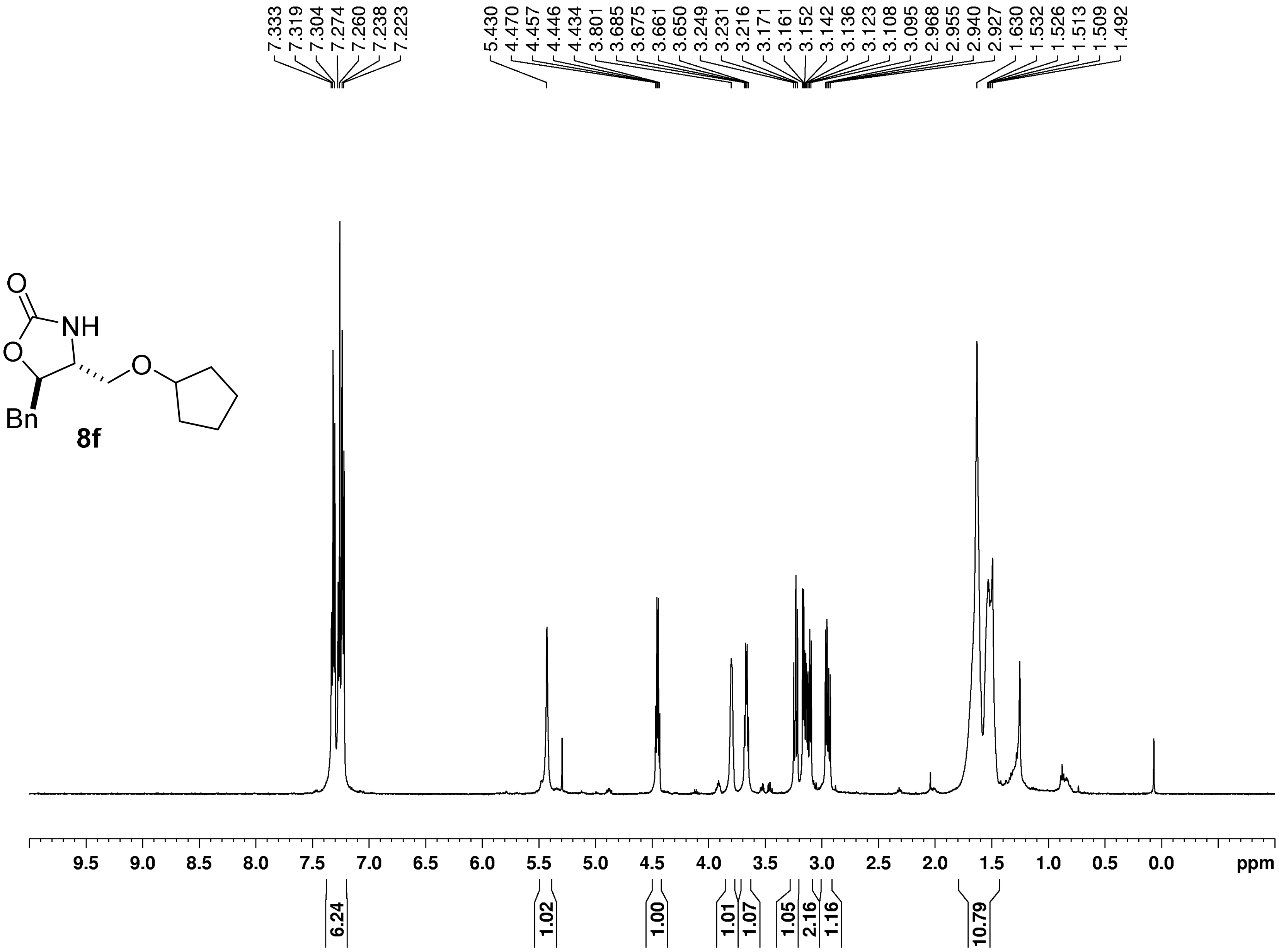

^{1}H NMR of compound **8f** (500 MHz, $CDCl_3$)

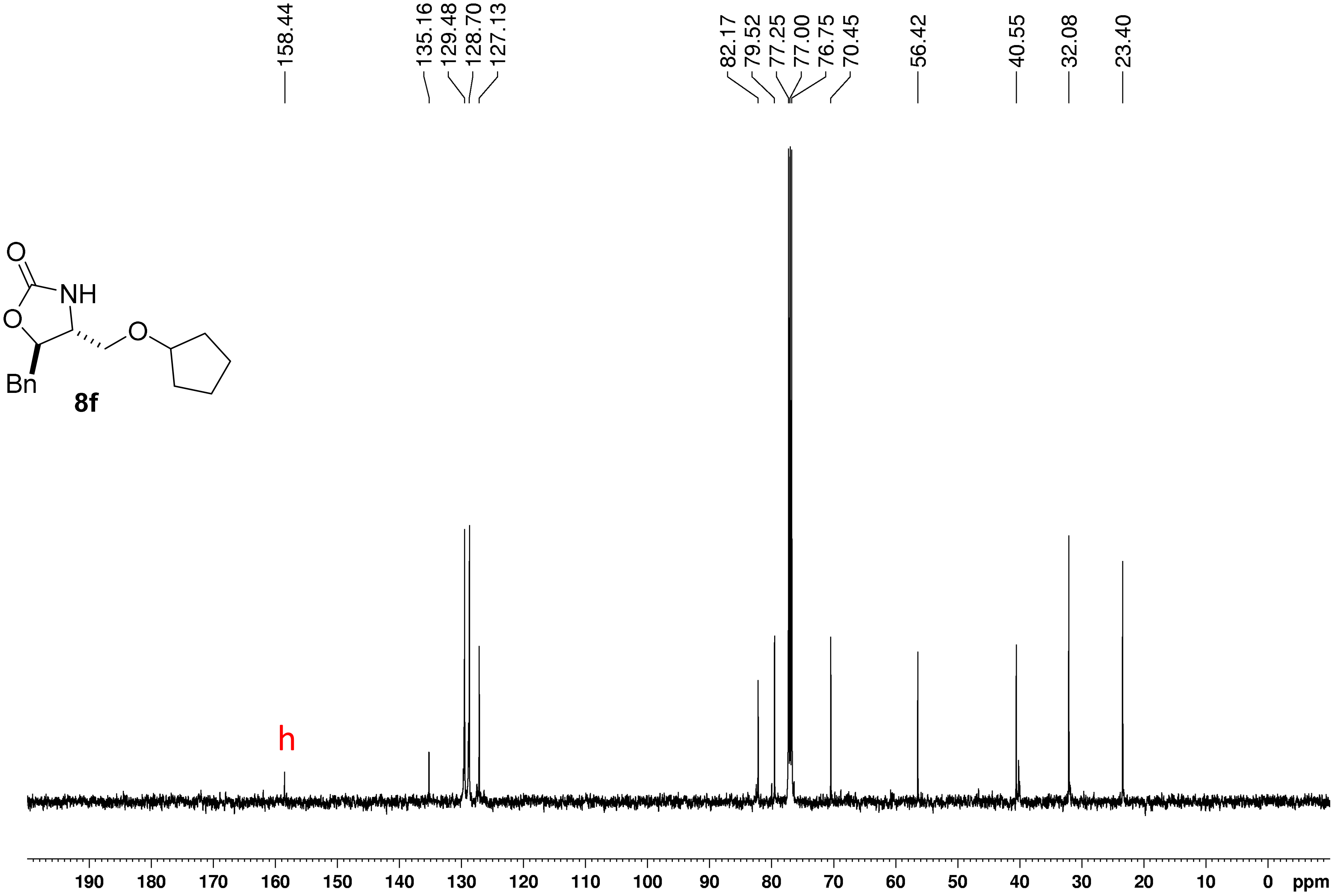

8f

$^{13}C\ \{^{1}H\}$ NMR of compound **8f** (126 MHz, $CDCl_3$)

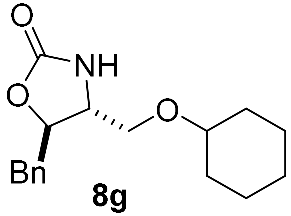

8g

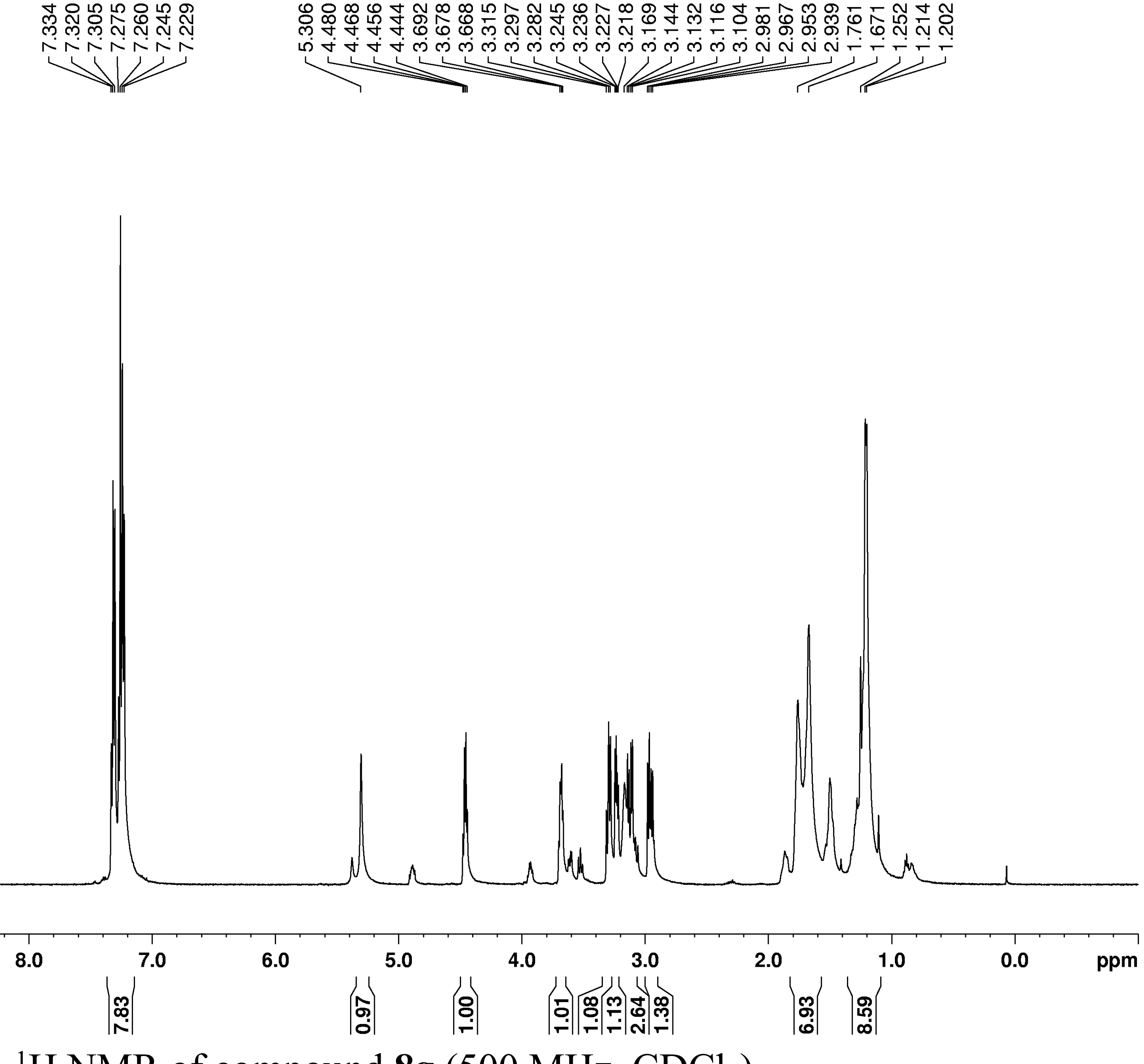

^{1}H NMR of compound **8g** (500 MHz, $CDCl_3$)

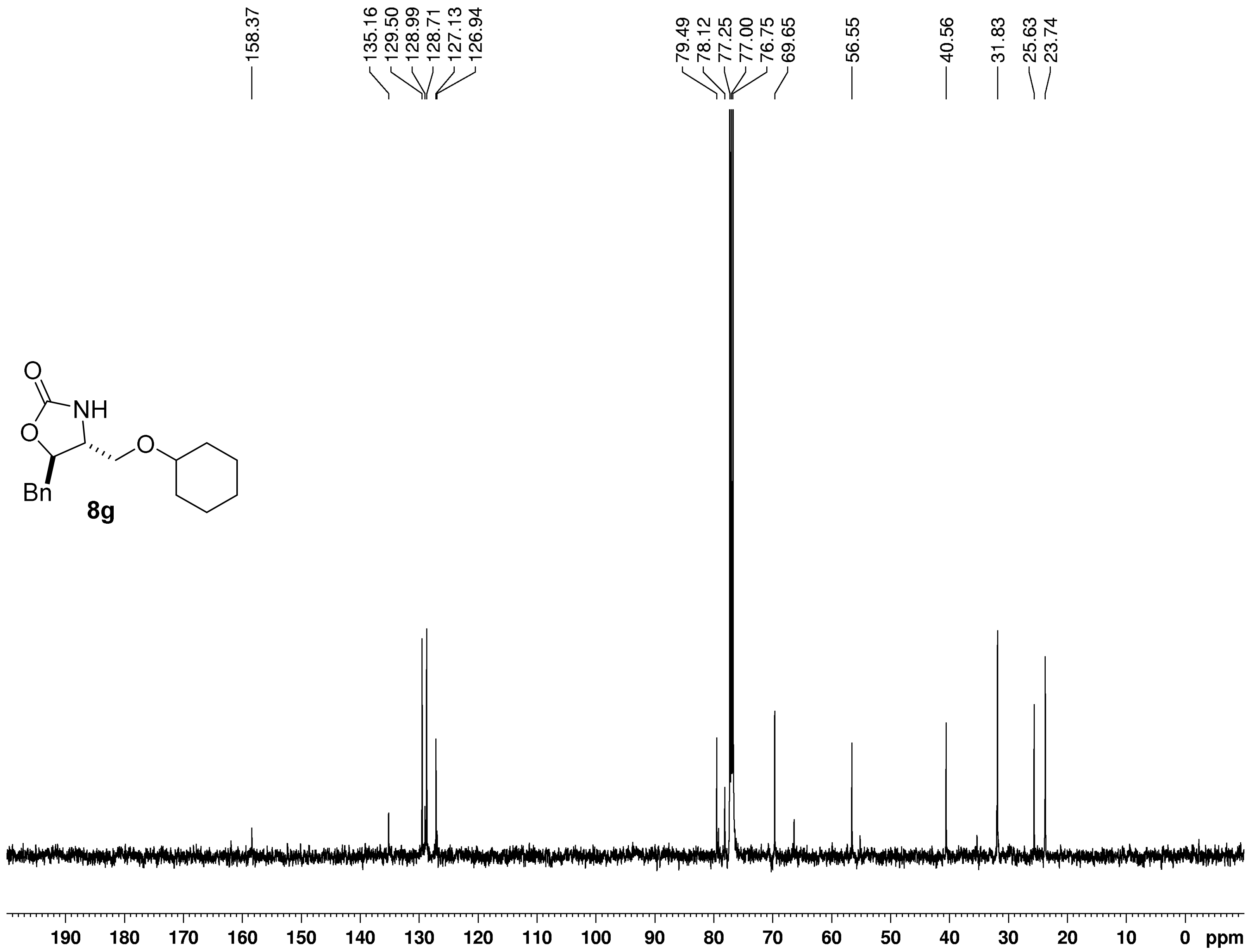

^{13}C {^1H} NMR of compound **8g** (126 MHz, $CDCl_3$)

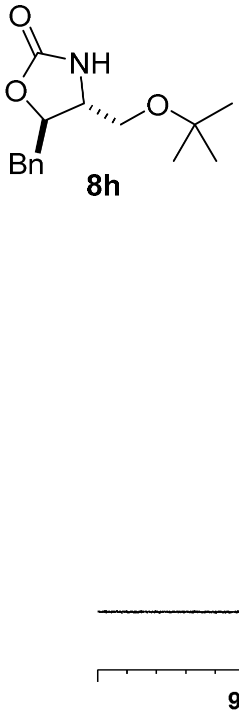

8h

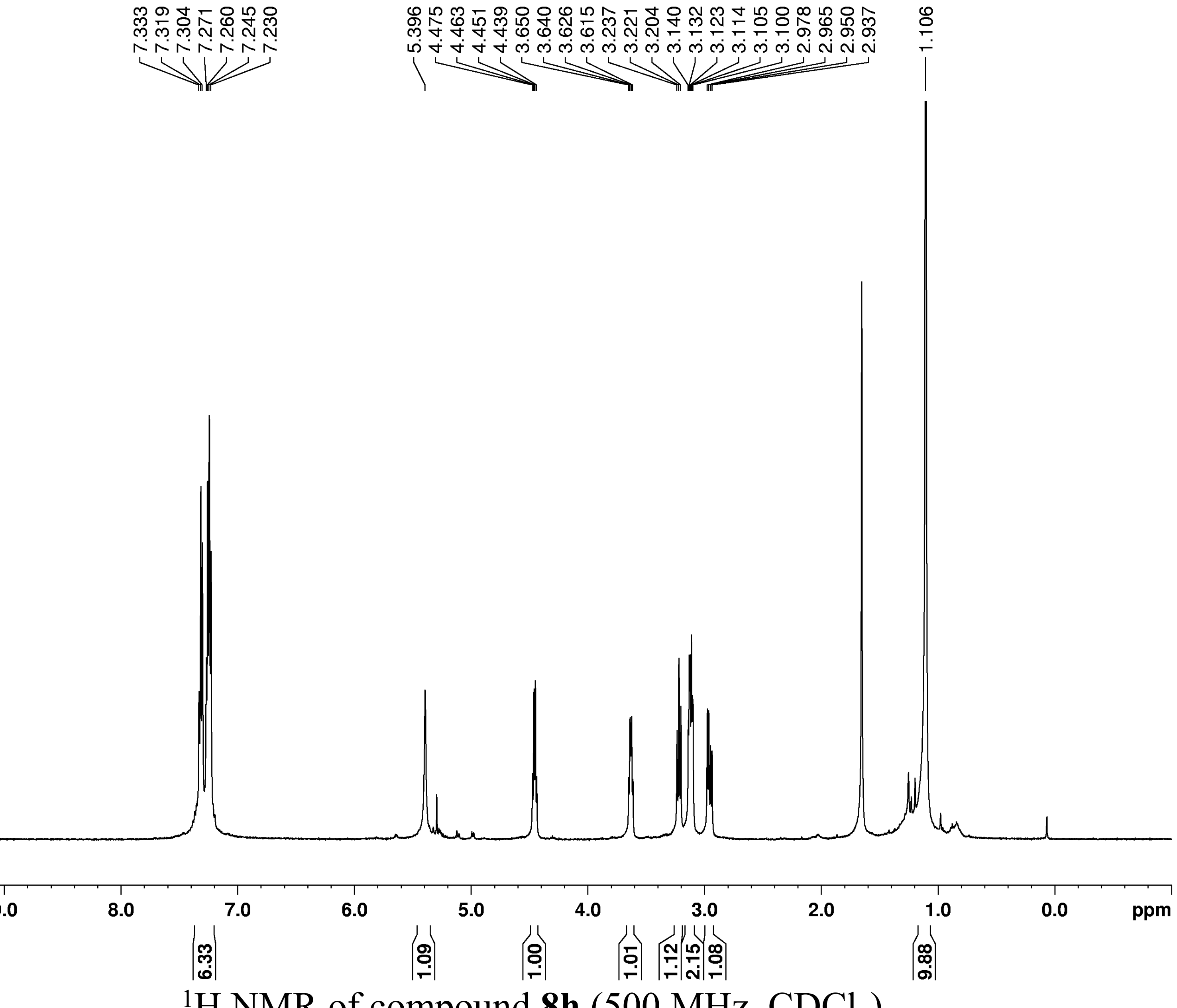

^{1}H NMR of compound **8h** (500 MHz, $CDCl_3$)

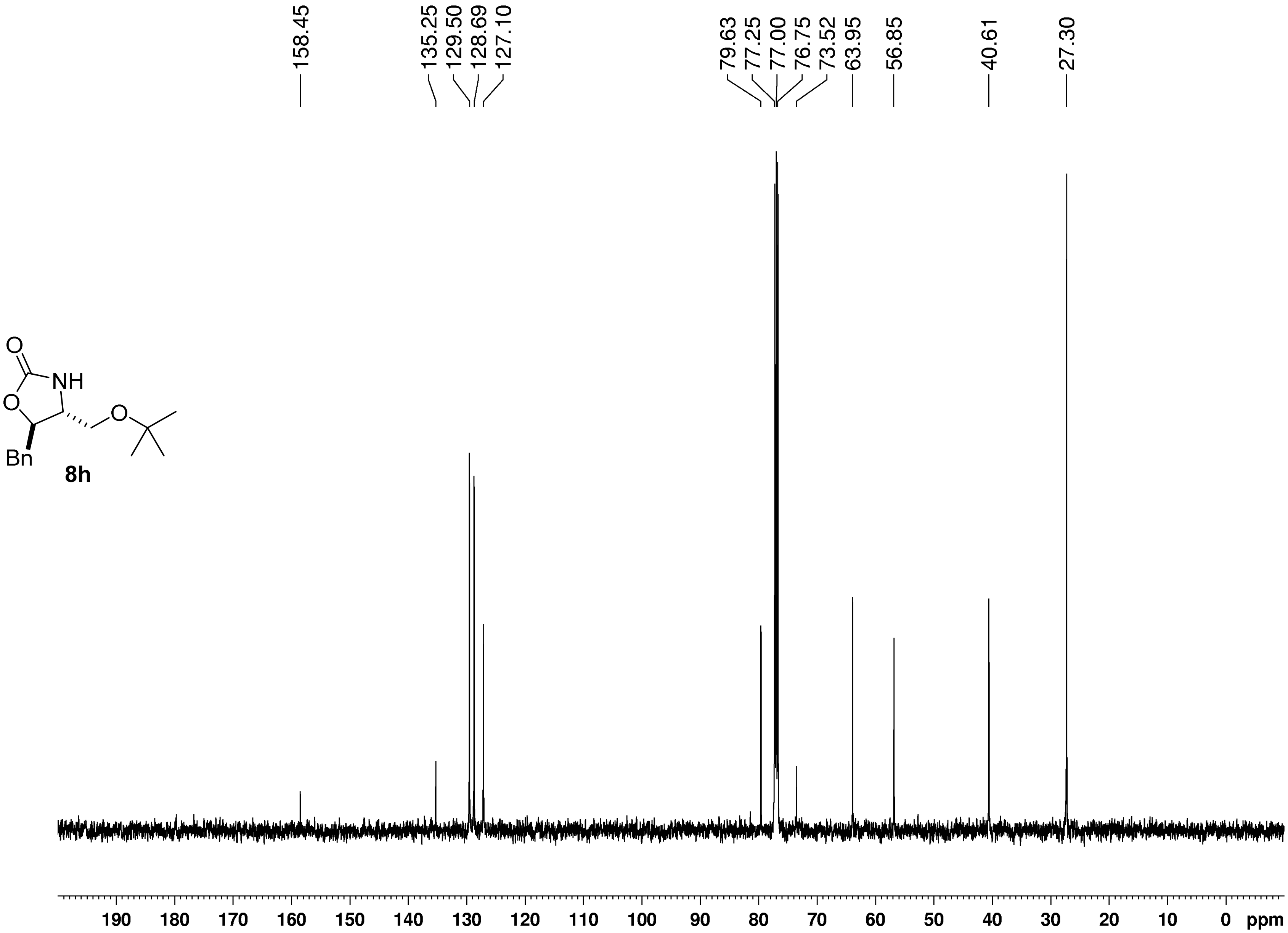

$^{13}C\ \{^{1}H\}$ NMR of compound **8h** (126 MHz, $CDCl_3$)

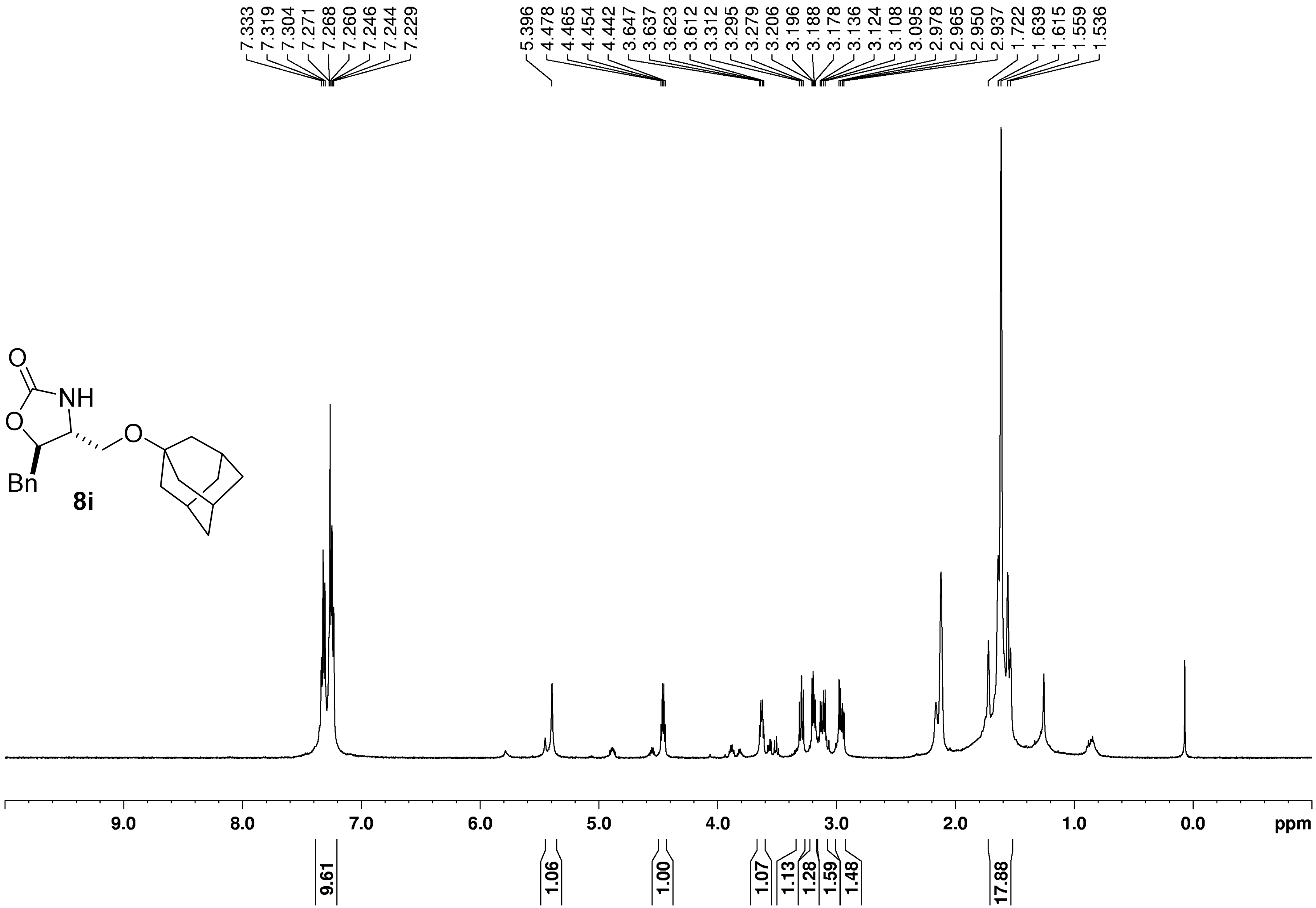

^{1}H NMR of compound **8i** (500 MHz, $CDCl_3$)

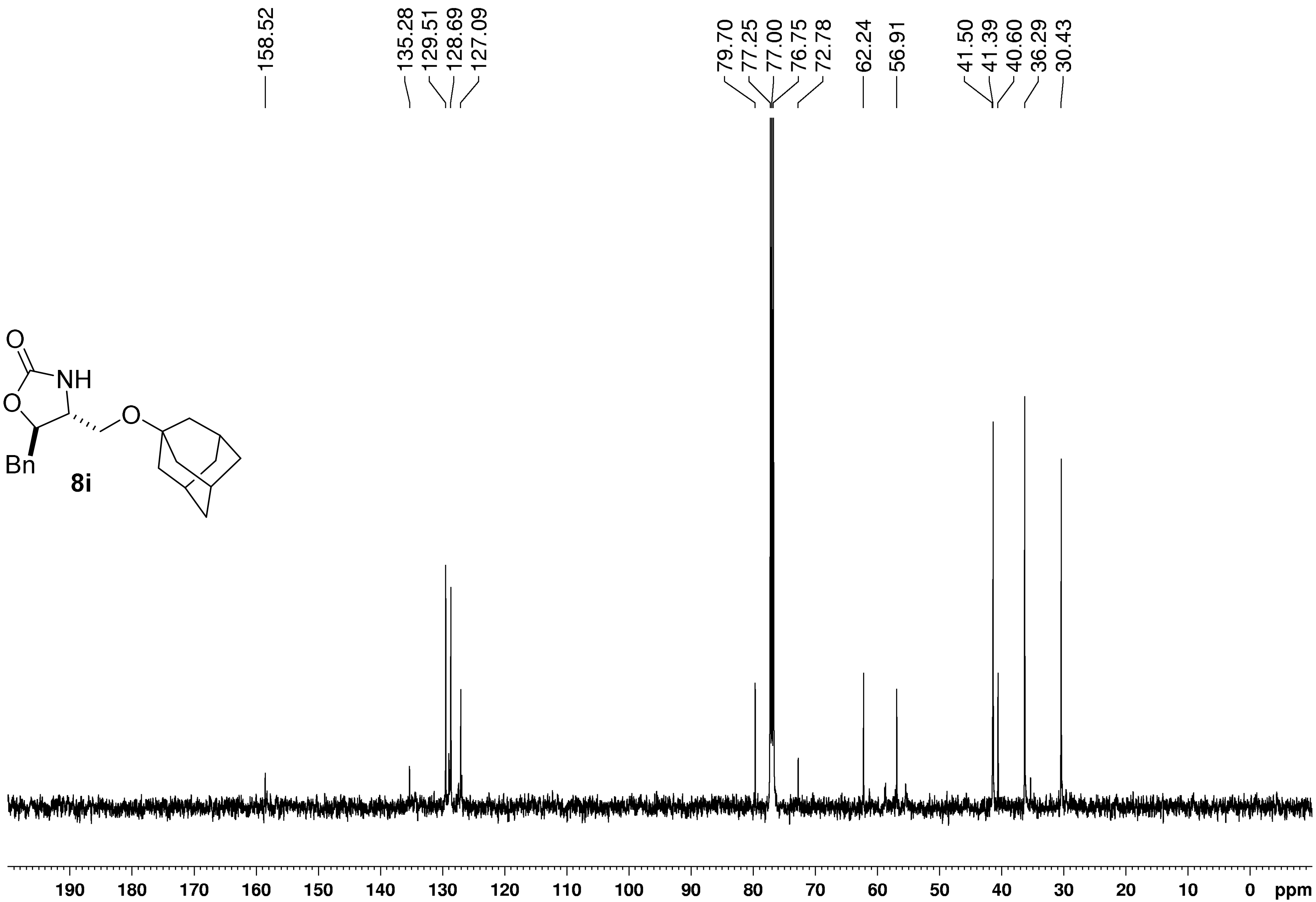

8i

$^{13}C\ \{^1H\}$ NMR of compound **8i** (126 MHz, $CDCl_3$)

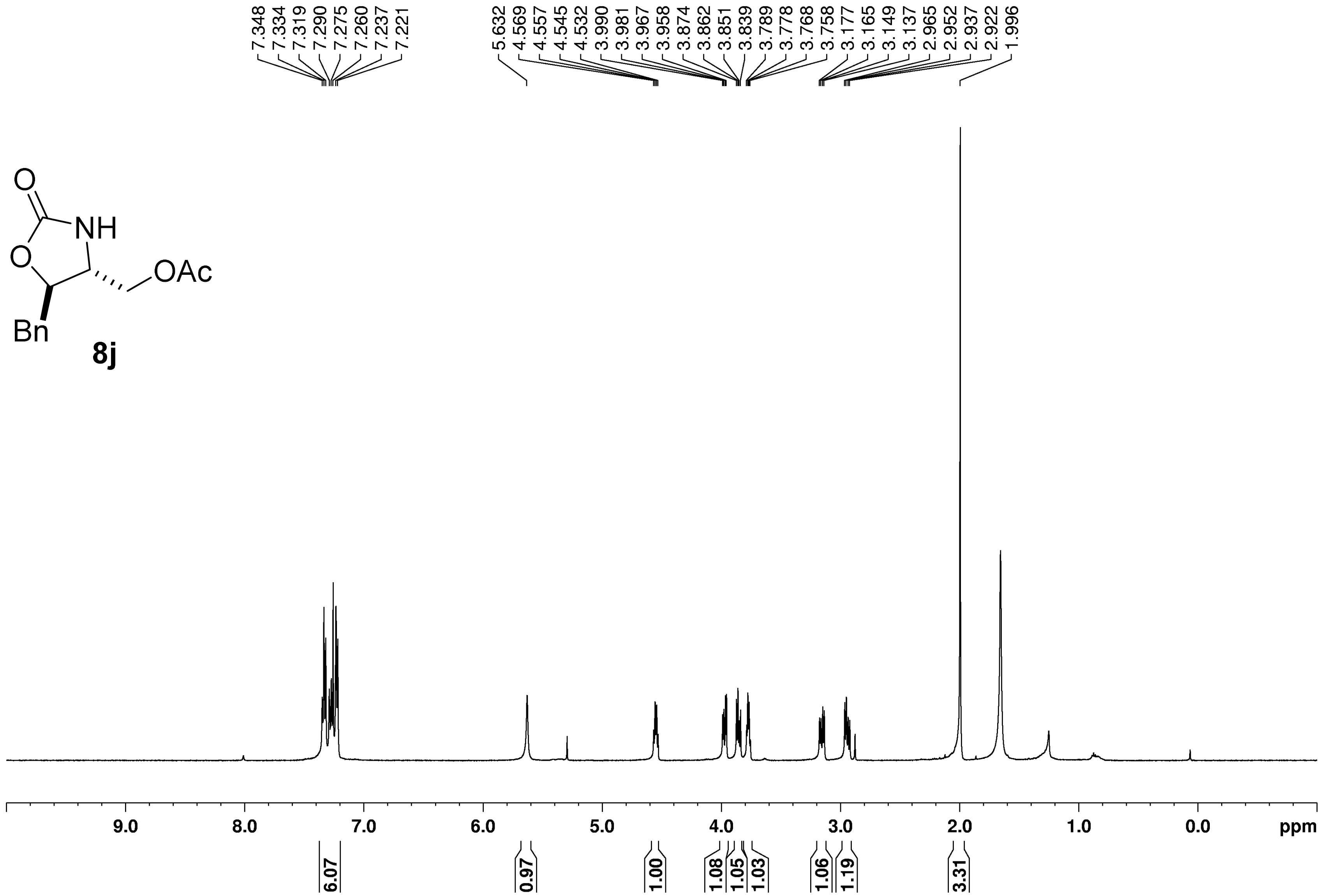

^{1}H NMR of compound **8j** (500 MHz, $CDCl_3$)

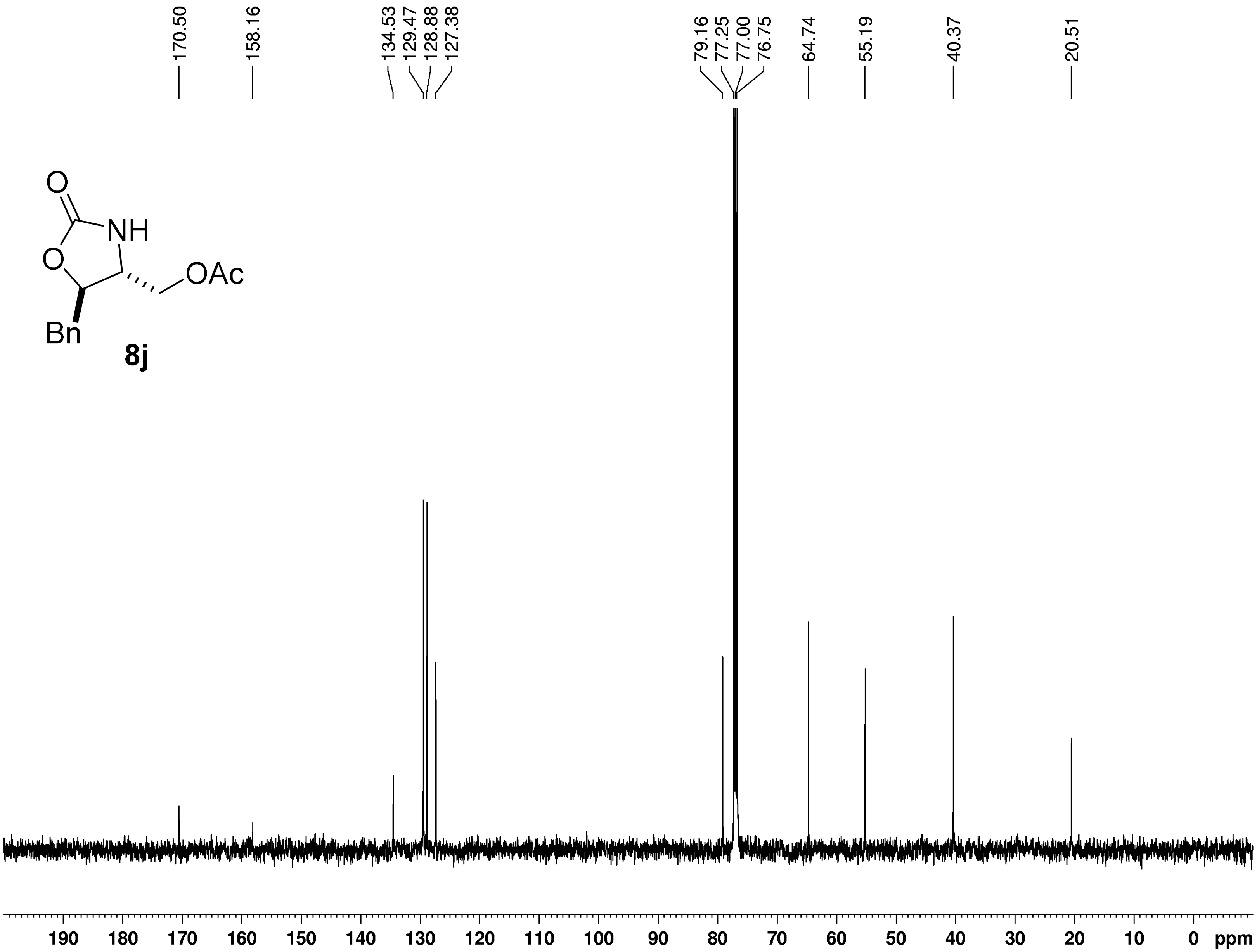

8j

$^{13}C\{^{1}H\}$ NMR of compound **8j** (126 MHz, $CDCl_3$)

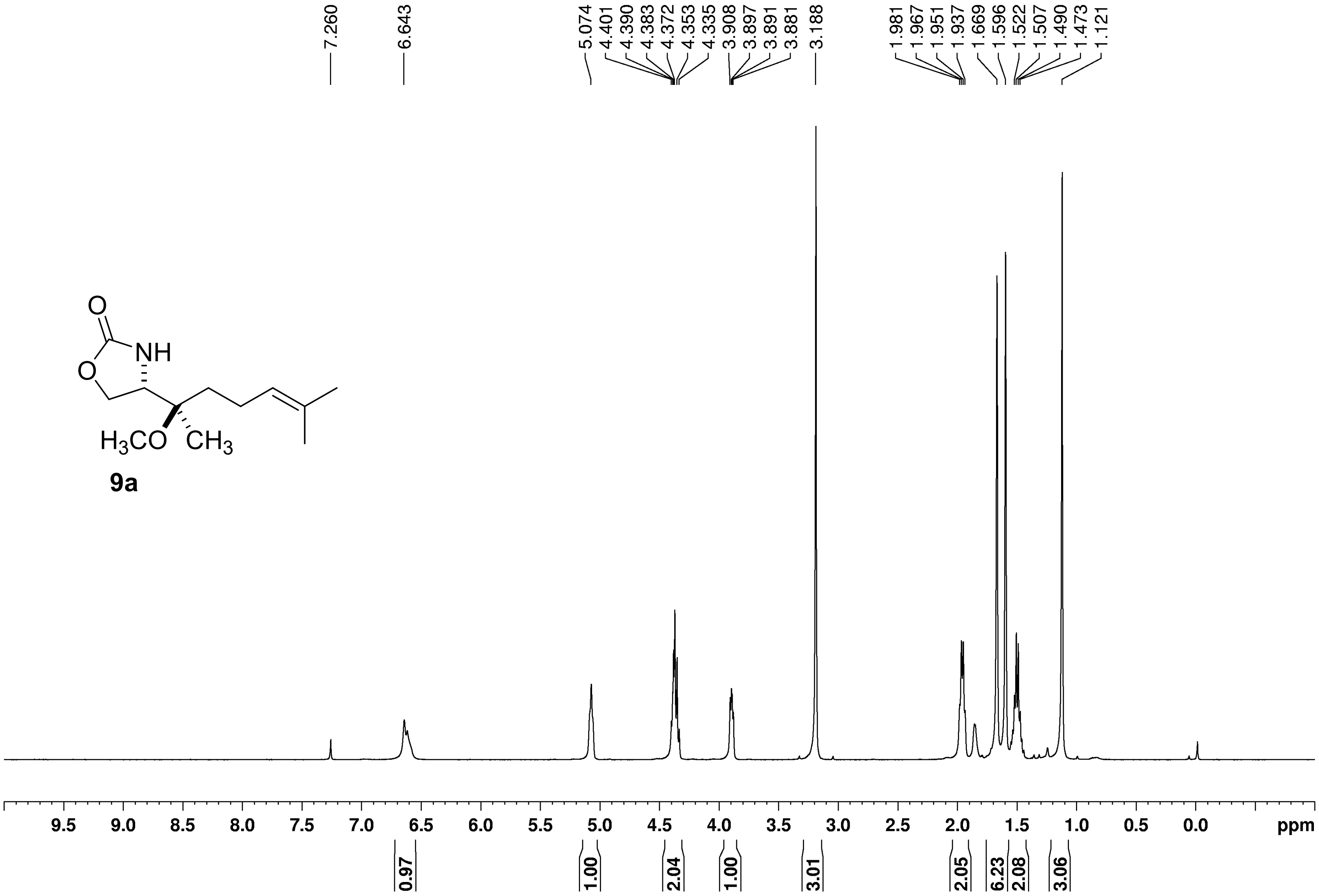

^{1}H NMR of compound **9a** (500 MHz, $CDCl_3$)

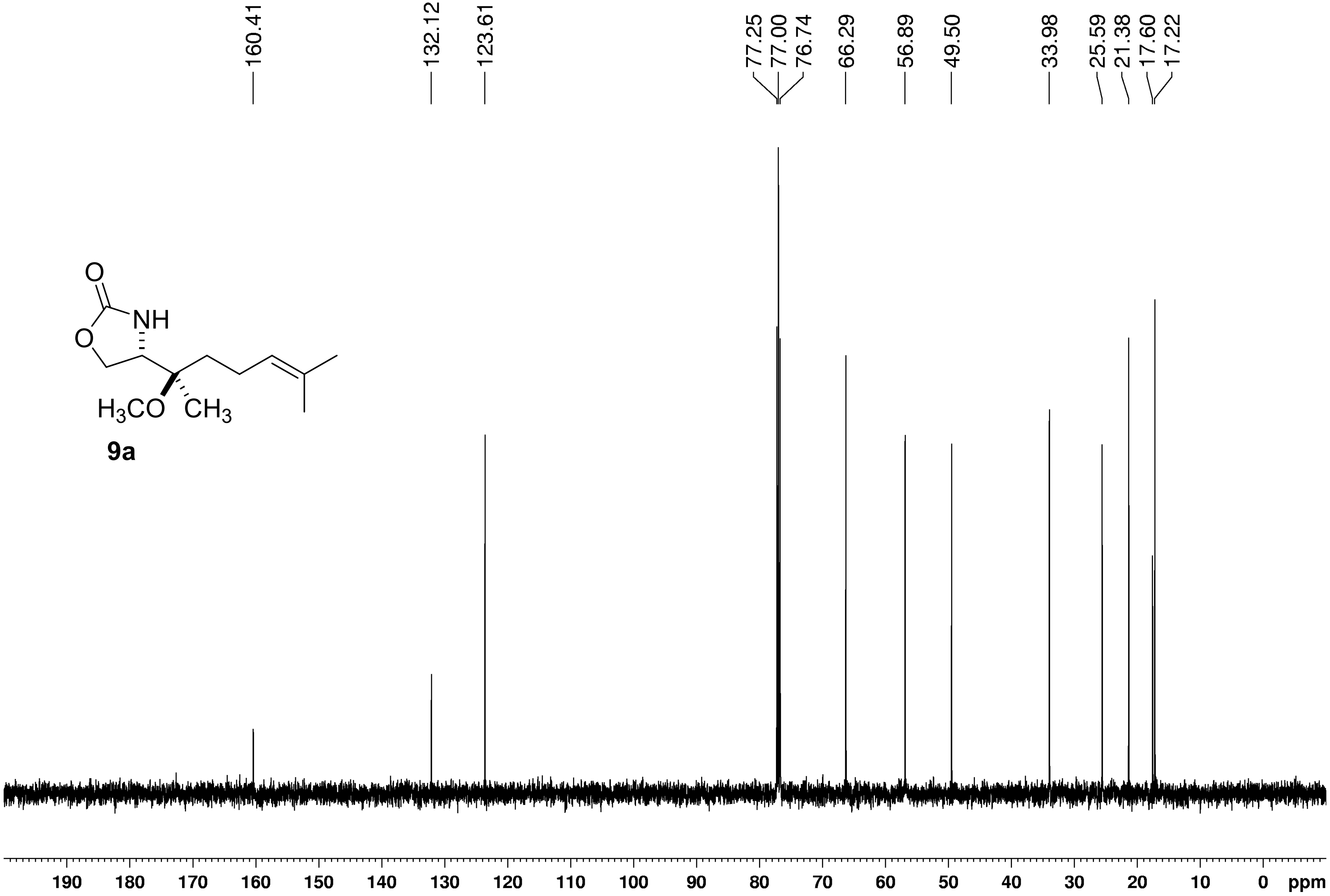

$^{13}C\{^{1}H\}$ NMR of compound **9a** (126 MHz, $CDCl_3$)

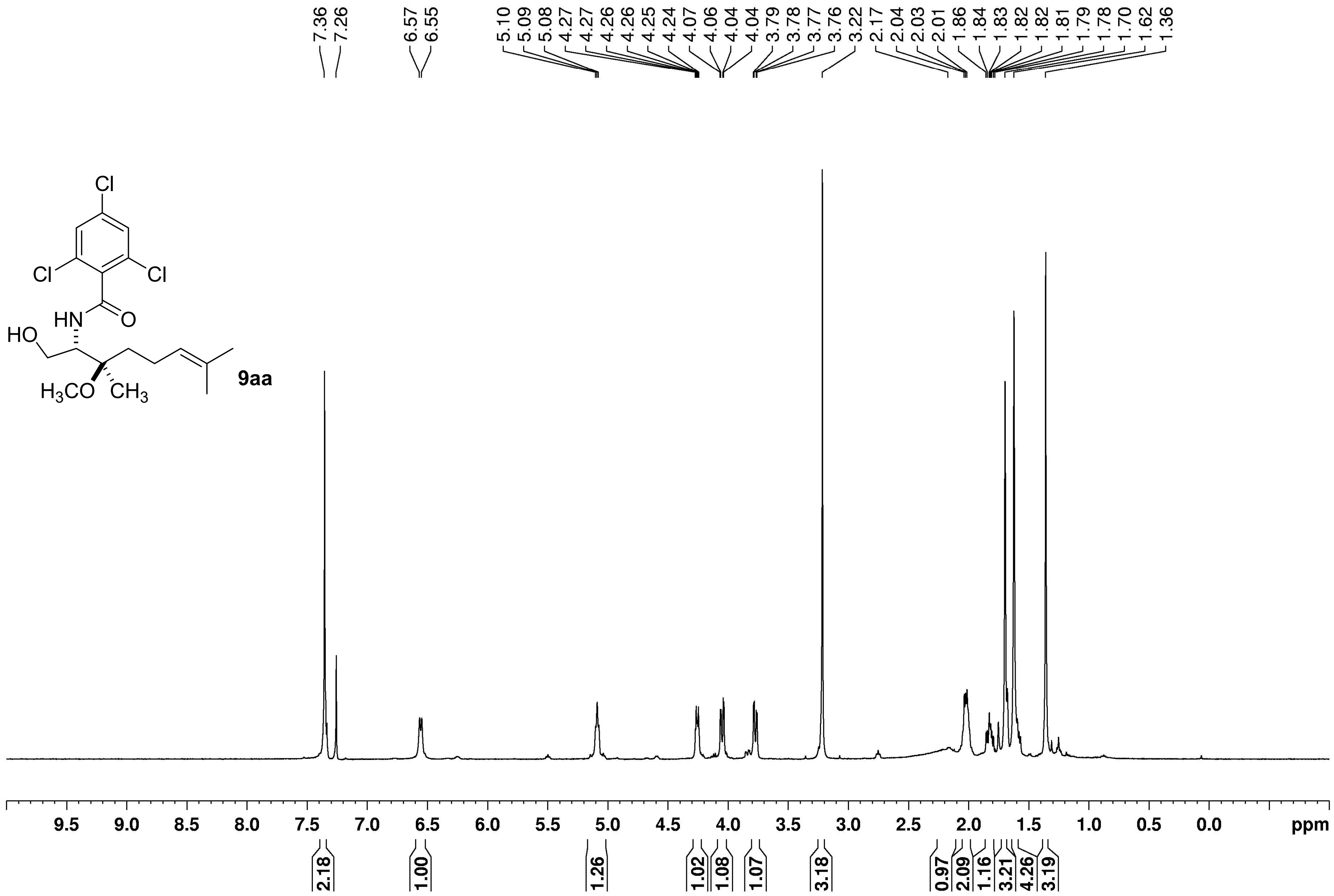

^{1}H NMR of compound **9aa** (500 MHz, $CDCl_3$)

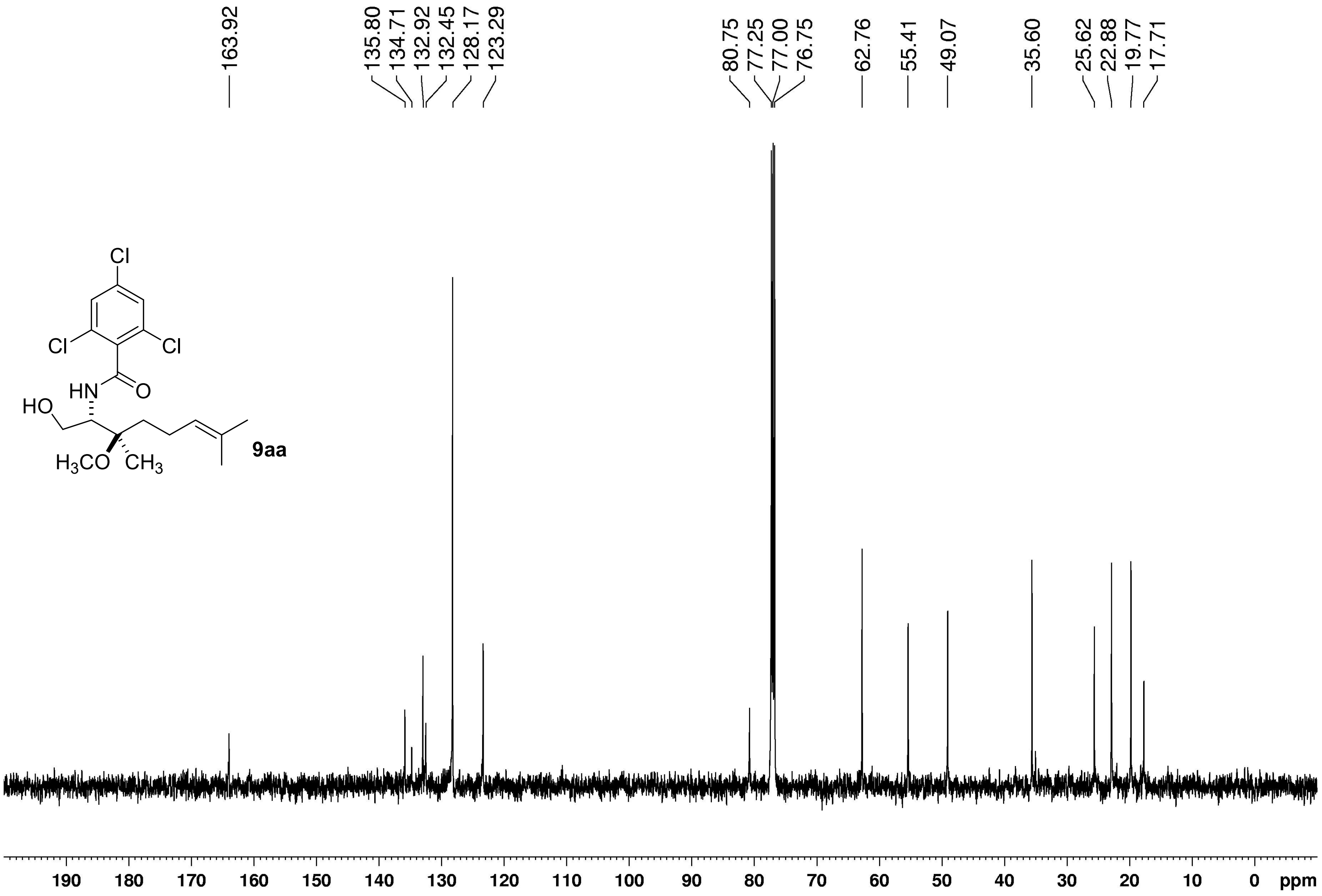

$^{13}C\{^{1}H\}$ NMR of compound **9aa** (126 MHz, $CDCl_3$)

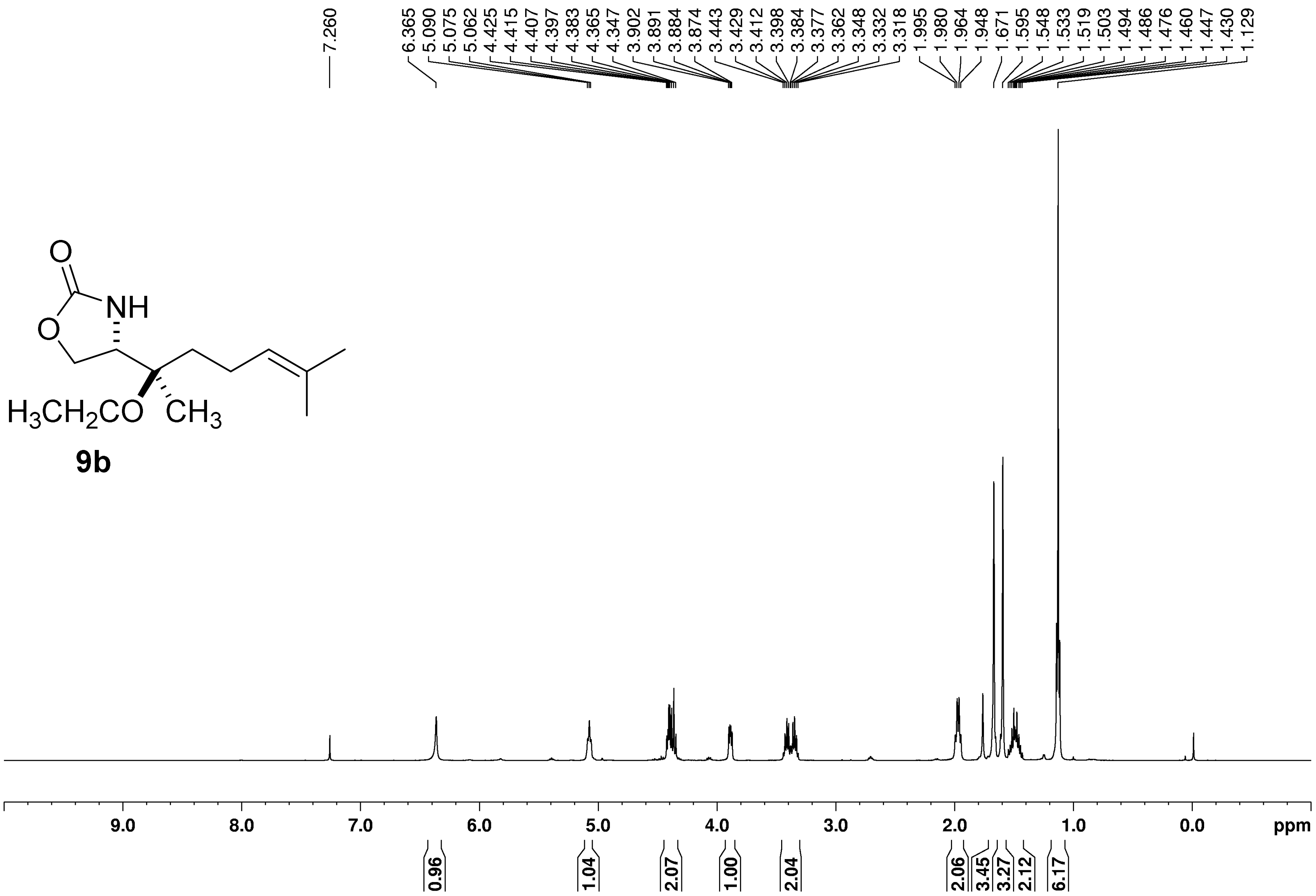

^{1}H NMR of compound **9b** (500 MHz, $CDCl_3$)

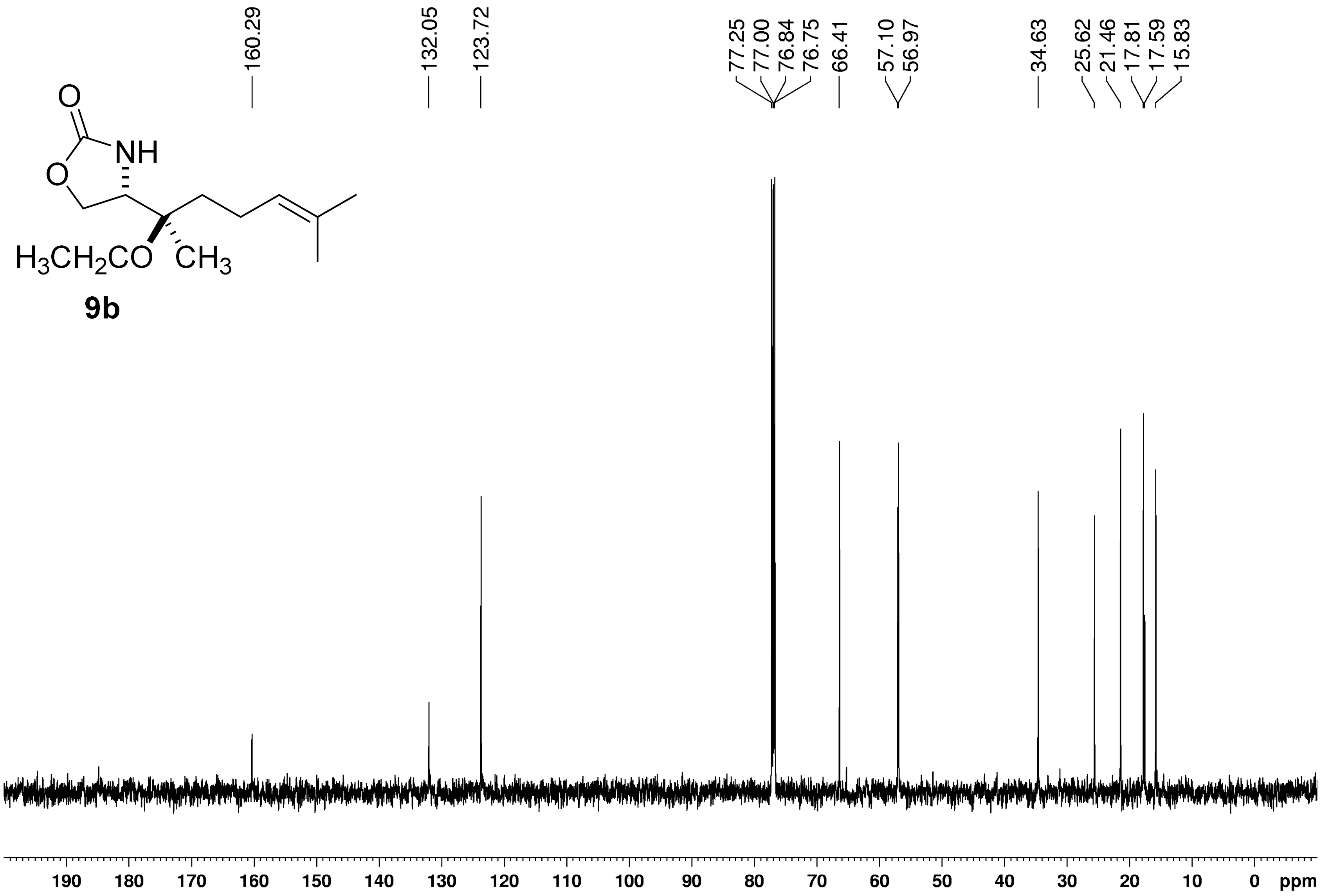

$^{13}C\{^{1}H\}$ NMR of compound **9b** (126 MHz, $CDCl_3$)

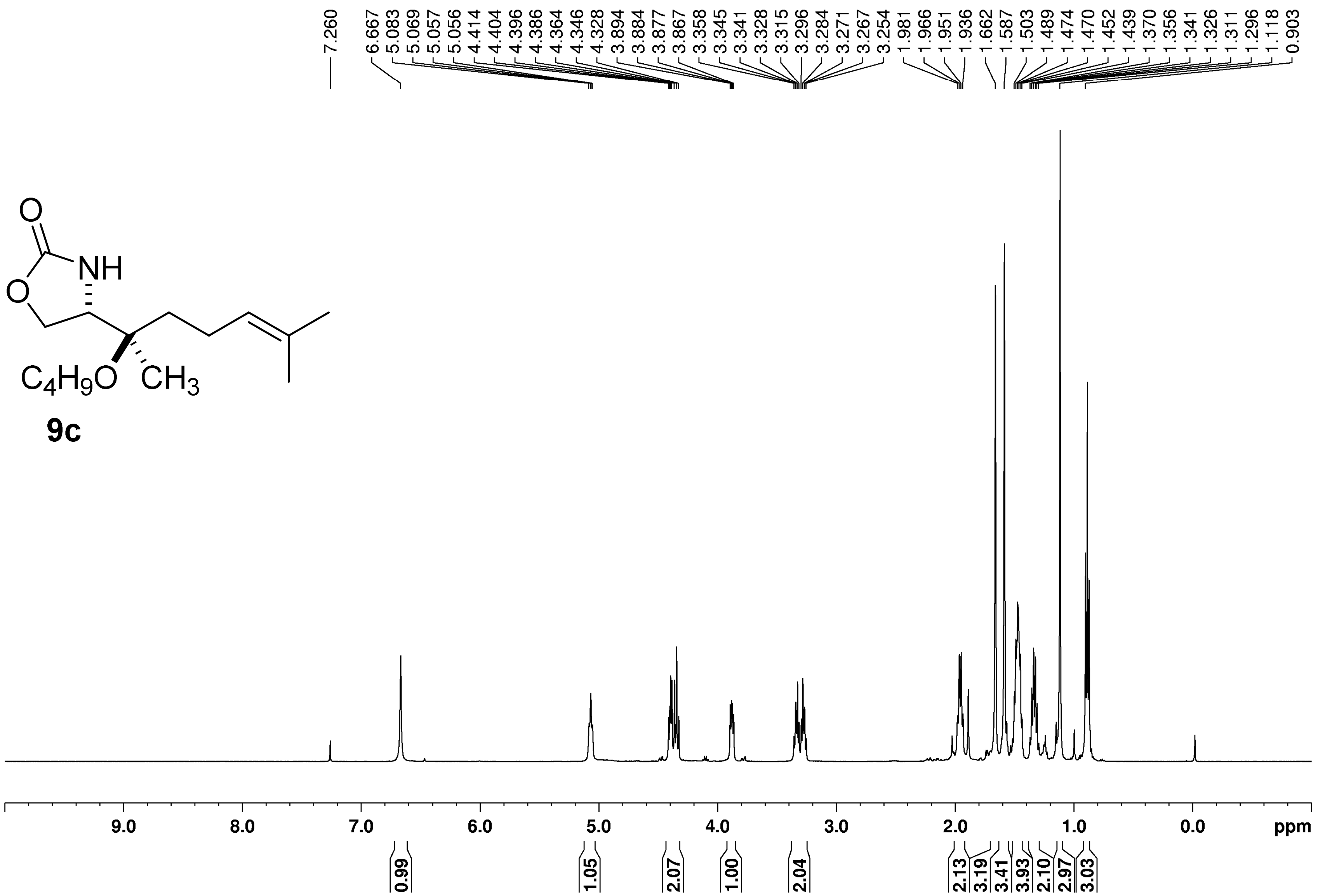

9c

^{1}H NMR of compound **9c** (500 MHz, $CDCl_3$)

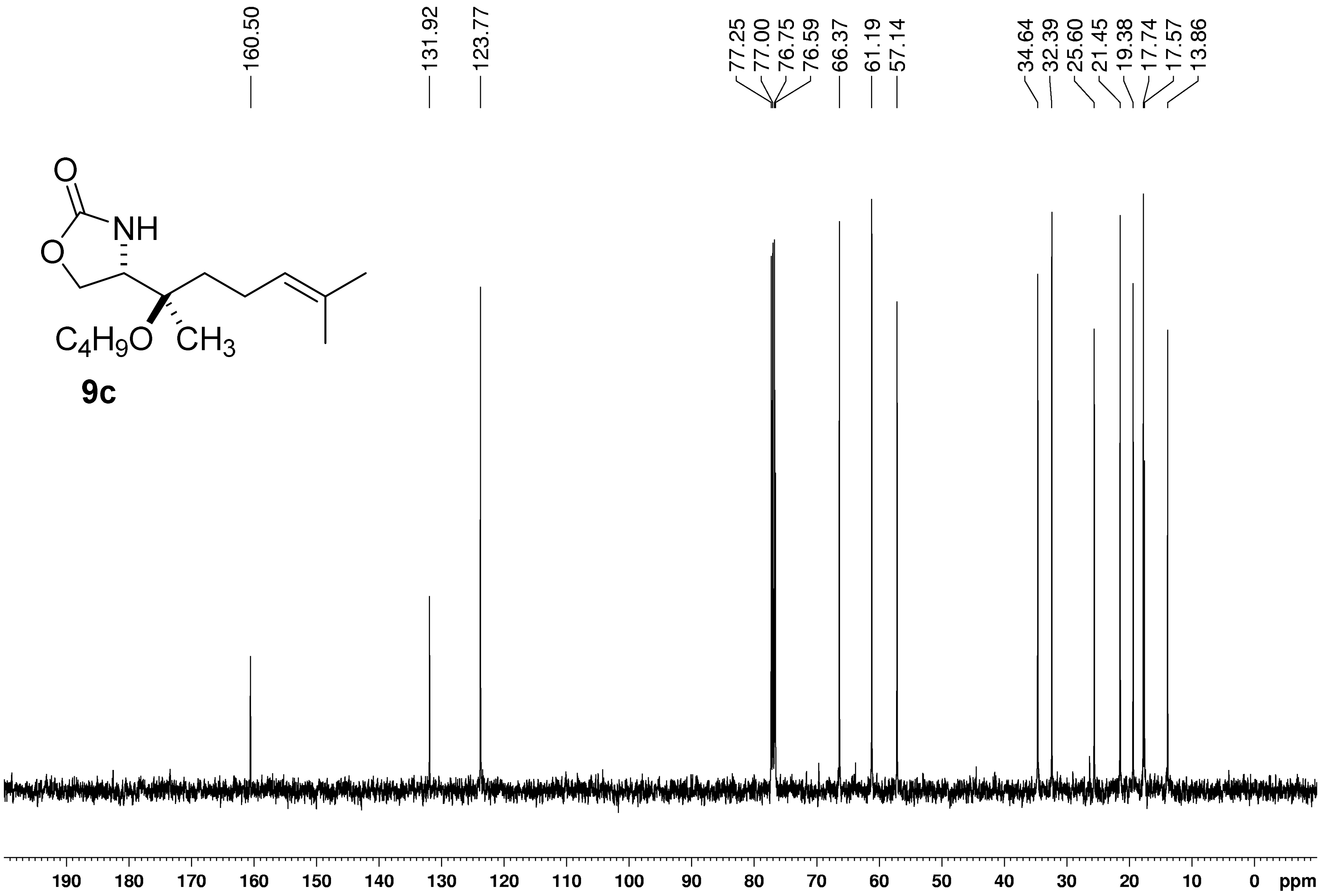

$^{13}C\{^{1}H\}$ NMR of compound **9c** (126 MHz, $CDCl_3$)

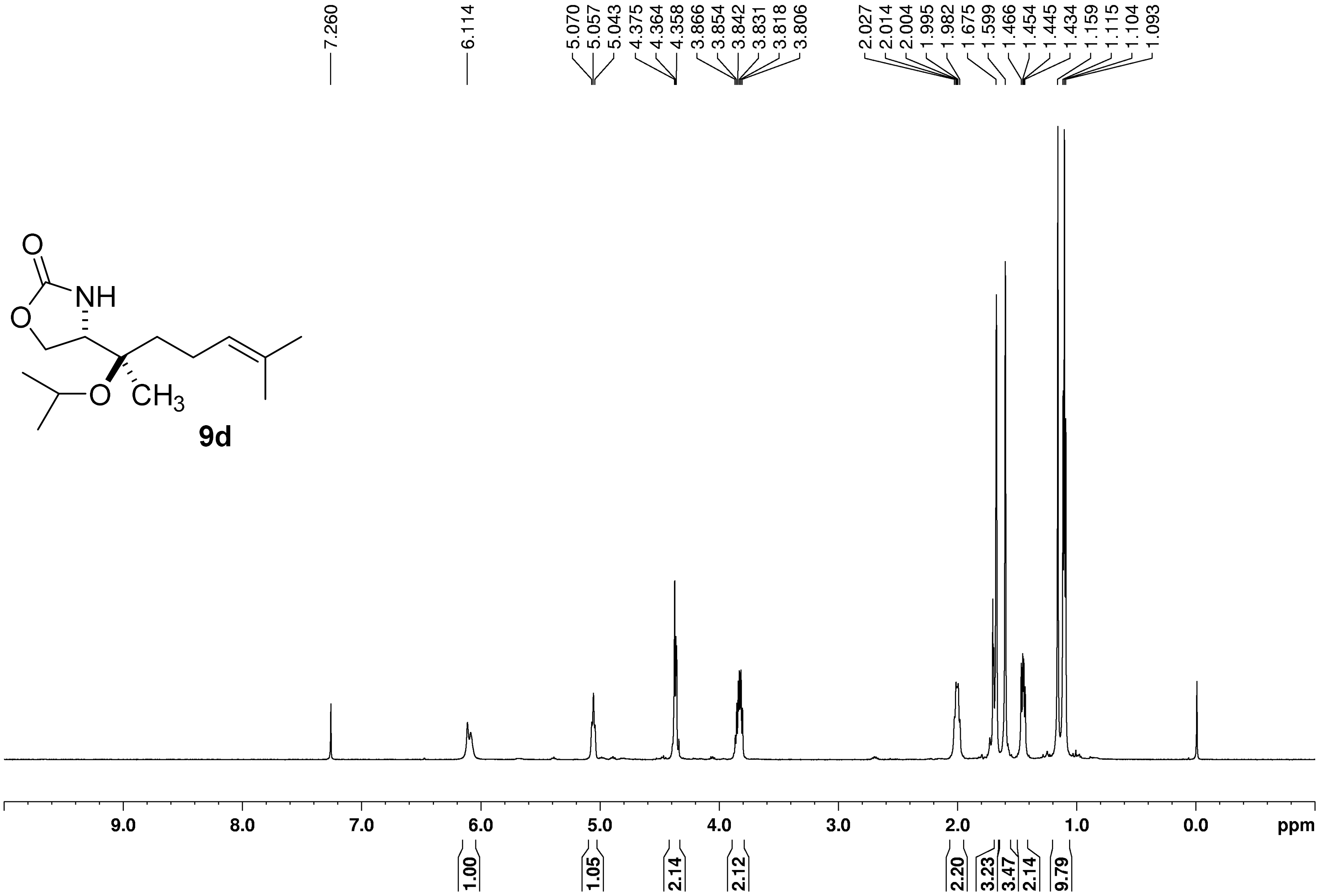

^{1}H NMR of compound **9d** (500 MHz, $CDCl_3$)

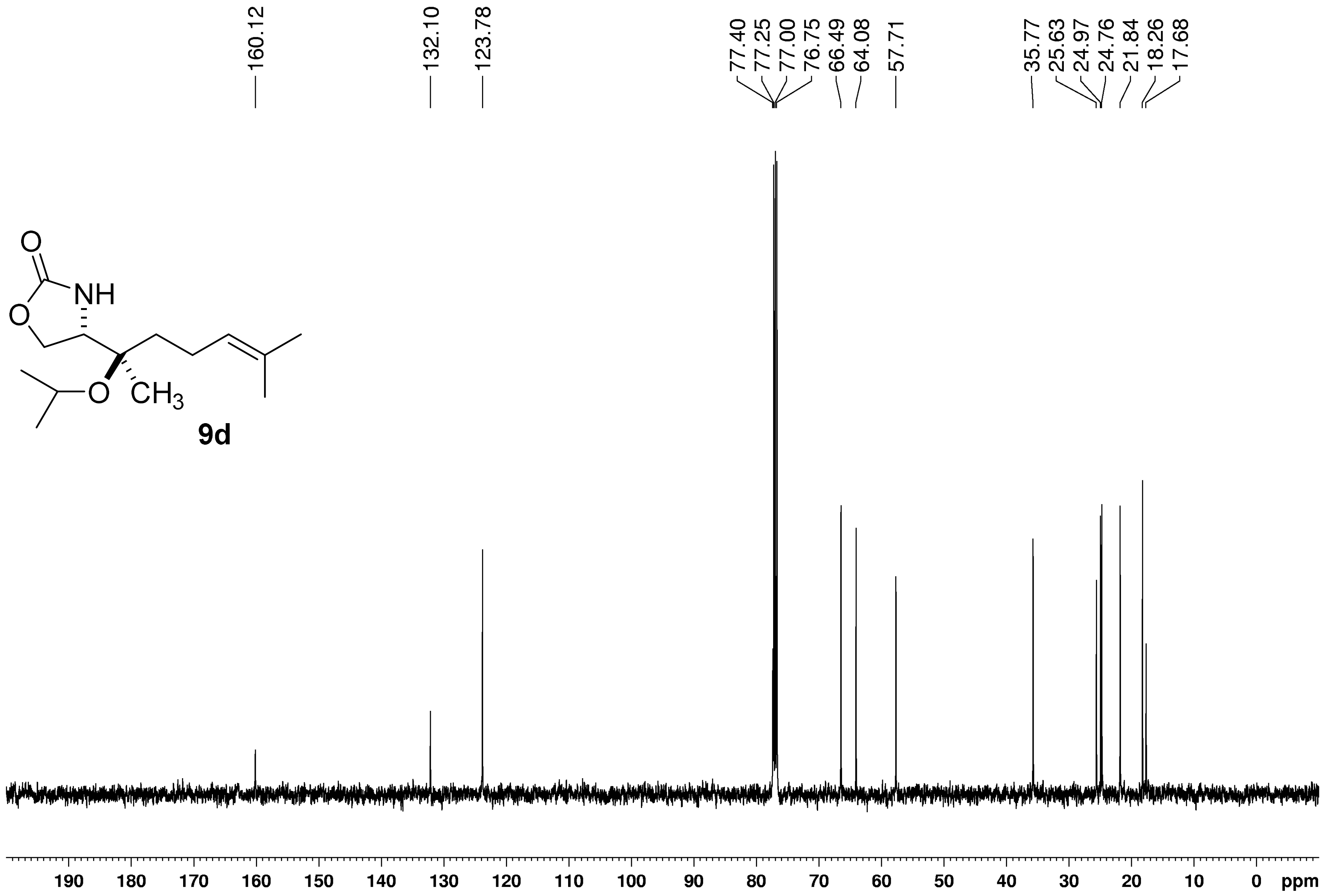

$^{13}C\{^{1}H\}$ NMR of compound **9d** (126 MHz, $CDCl_3$)

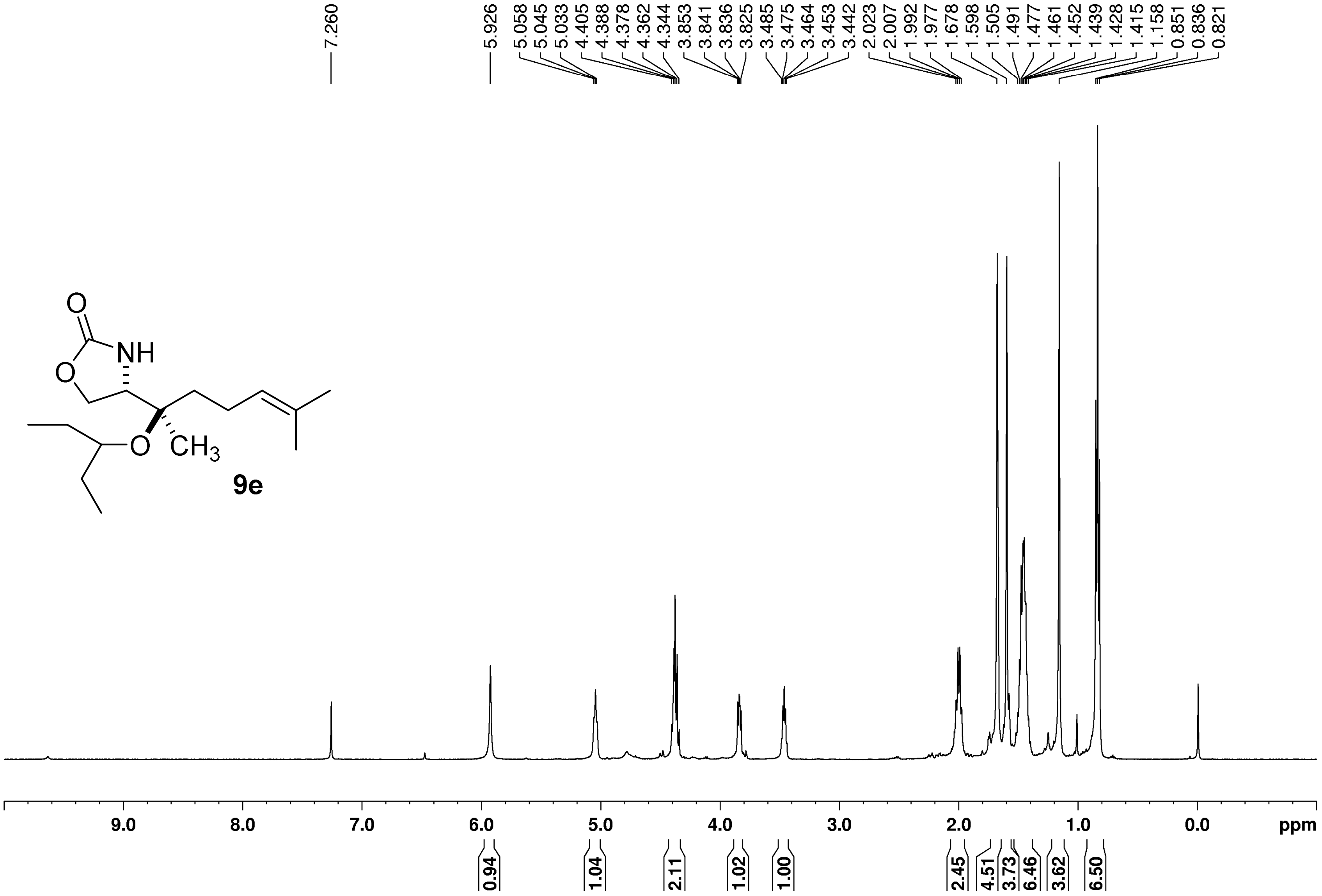

^{1}H NMR of compound **9e** (500 MHz, $CDCl_3$)

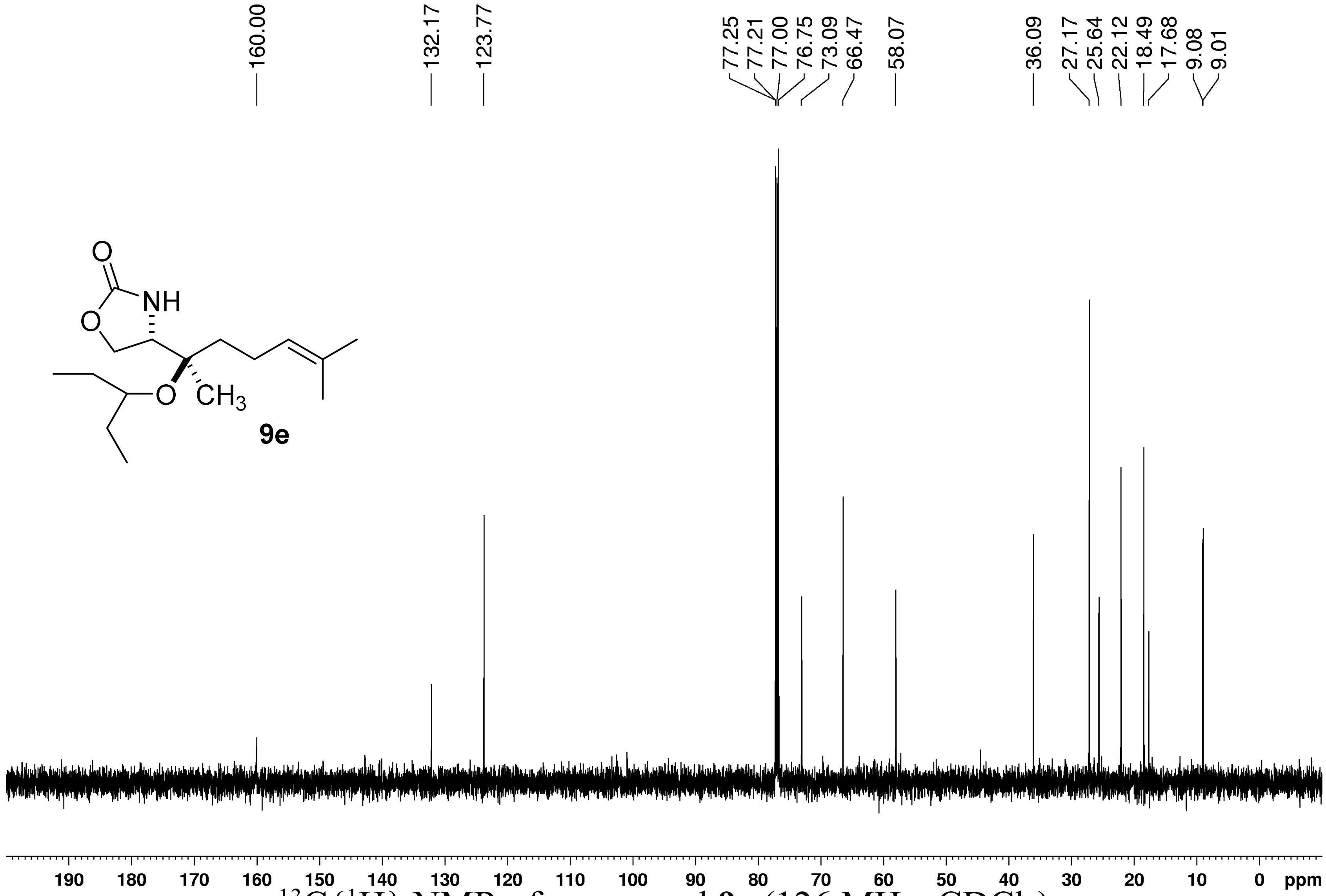

$^{13}C\{^1H\}$ NMR of compound **9e** (126 MHz, $CDCl_3$)

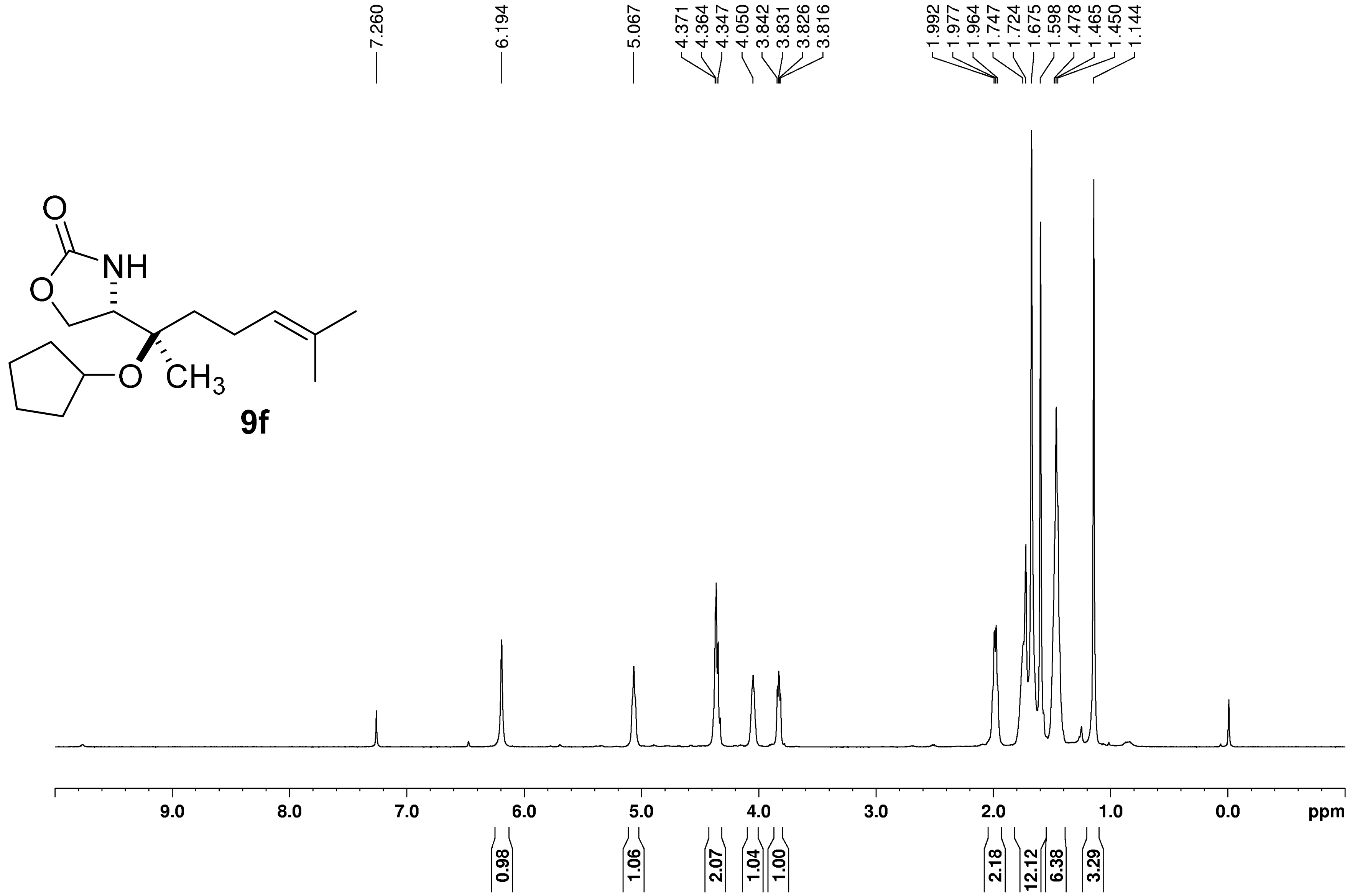

^{1}H NMR of compound **9f** (500 MHz, $CDCl_3$)

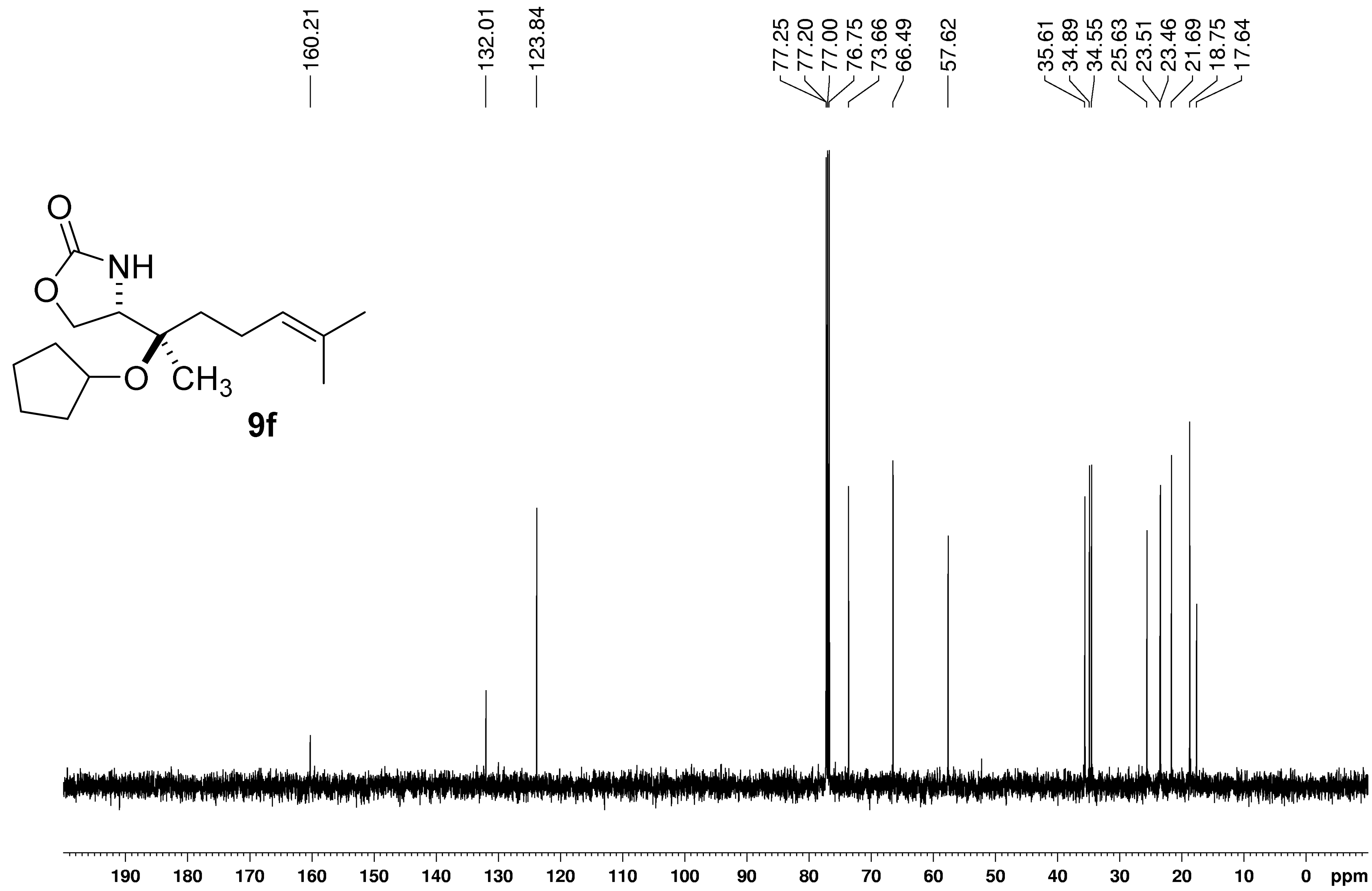

$^{13}C\{^{1}H\}$ NMR of compound **9f** (126 MHz, $CDCl_3$)

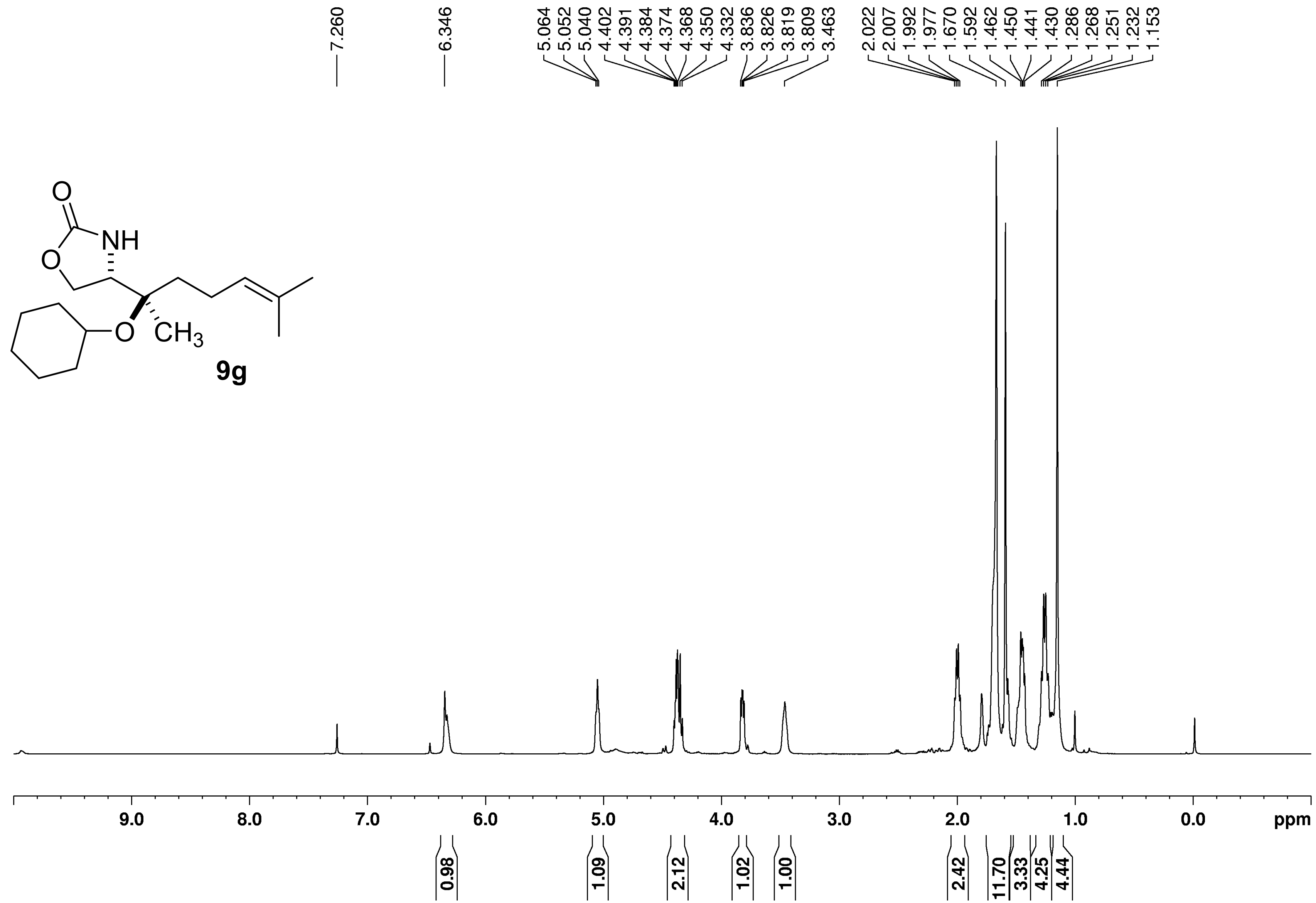

9g

^{1}H NMR of compound **9g** (500 MHz, $CDCl_3$)

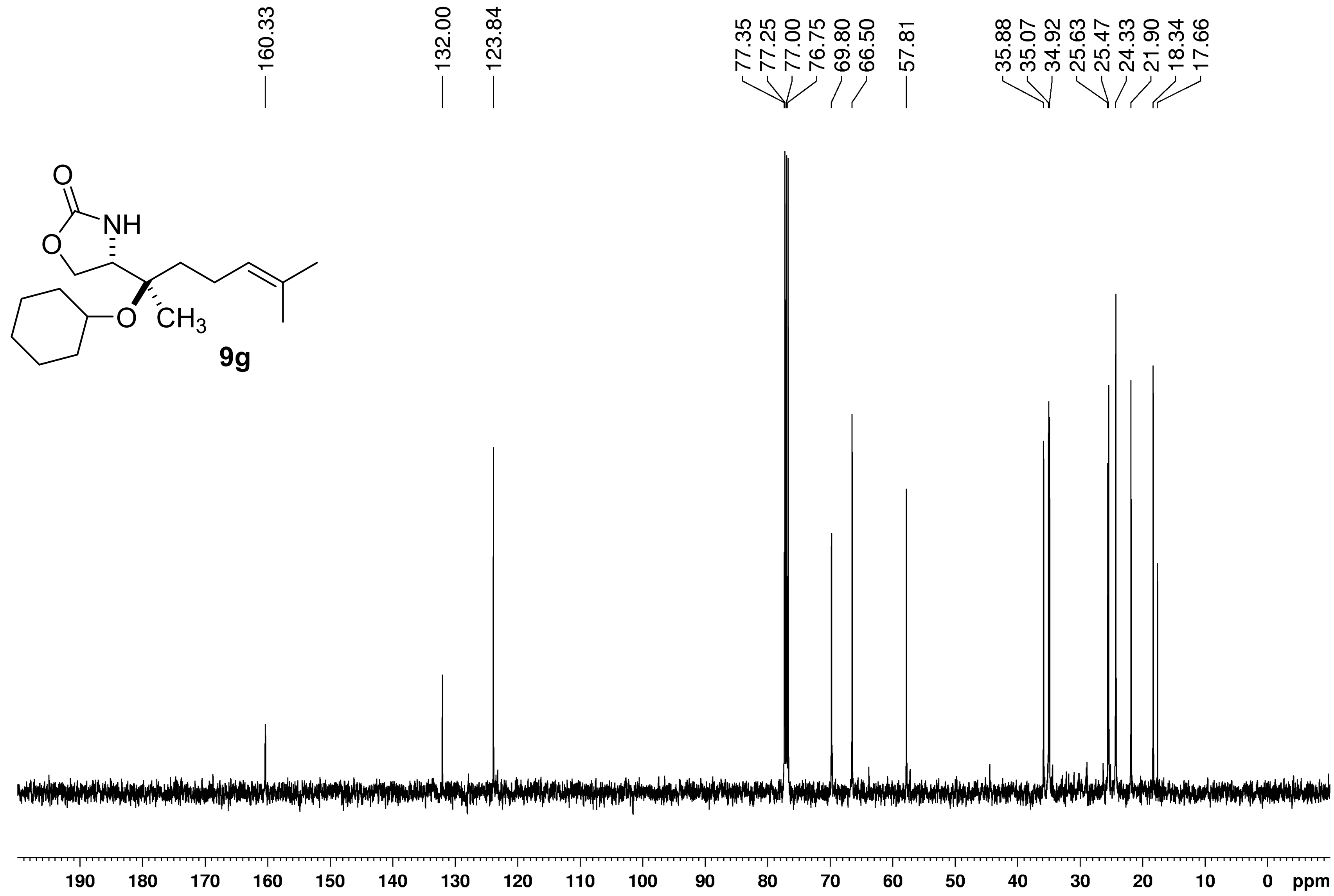

$^{13}C\{^{1}H\}$ NMR of compound **9g** (126 MHz, $CDCl_3$)

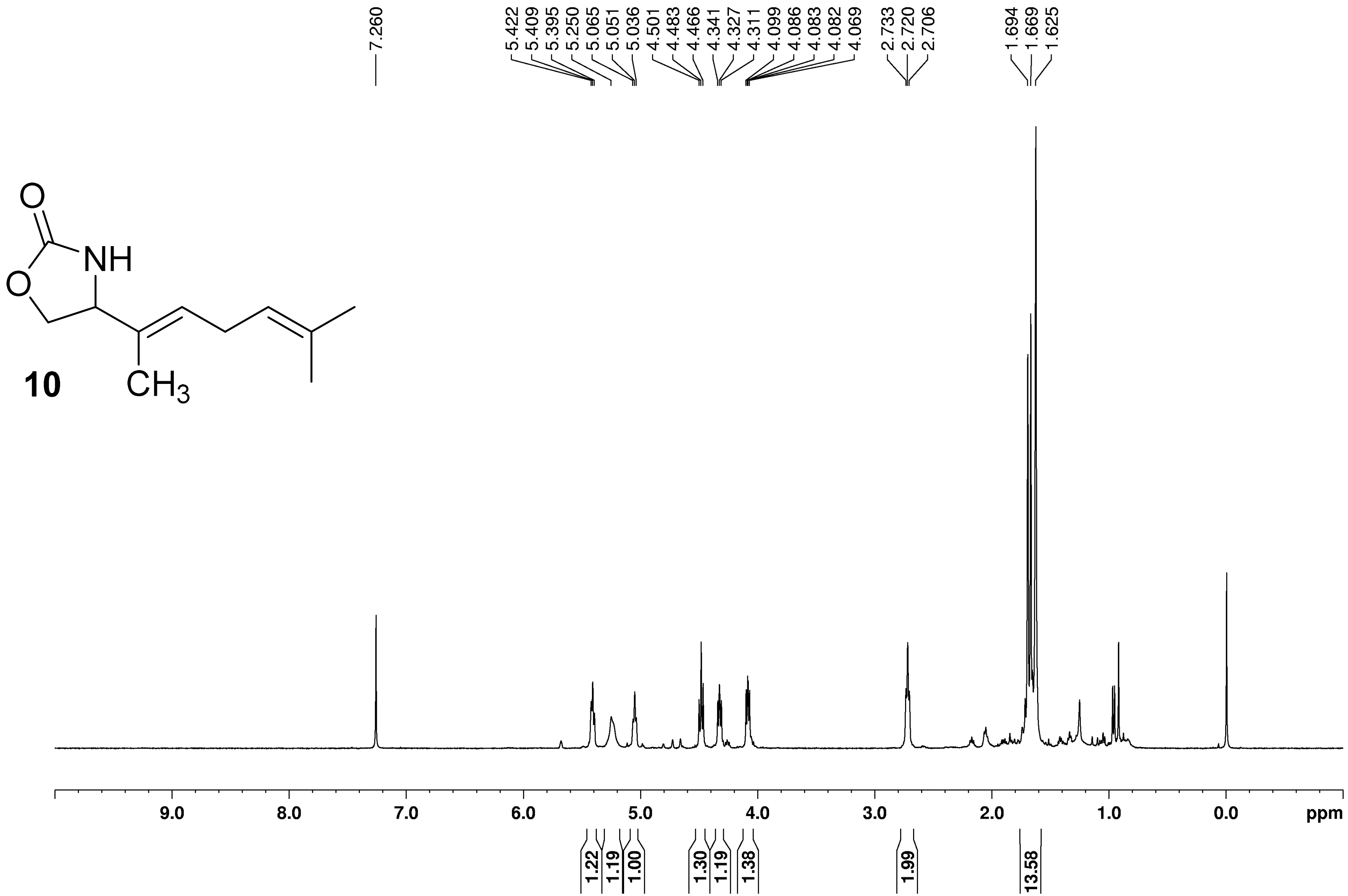

^{1}H NMR of compound **10** (500 MHz, $CDCl_3$)

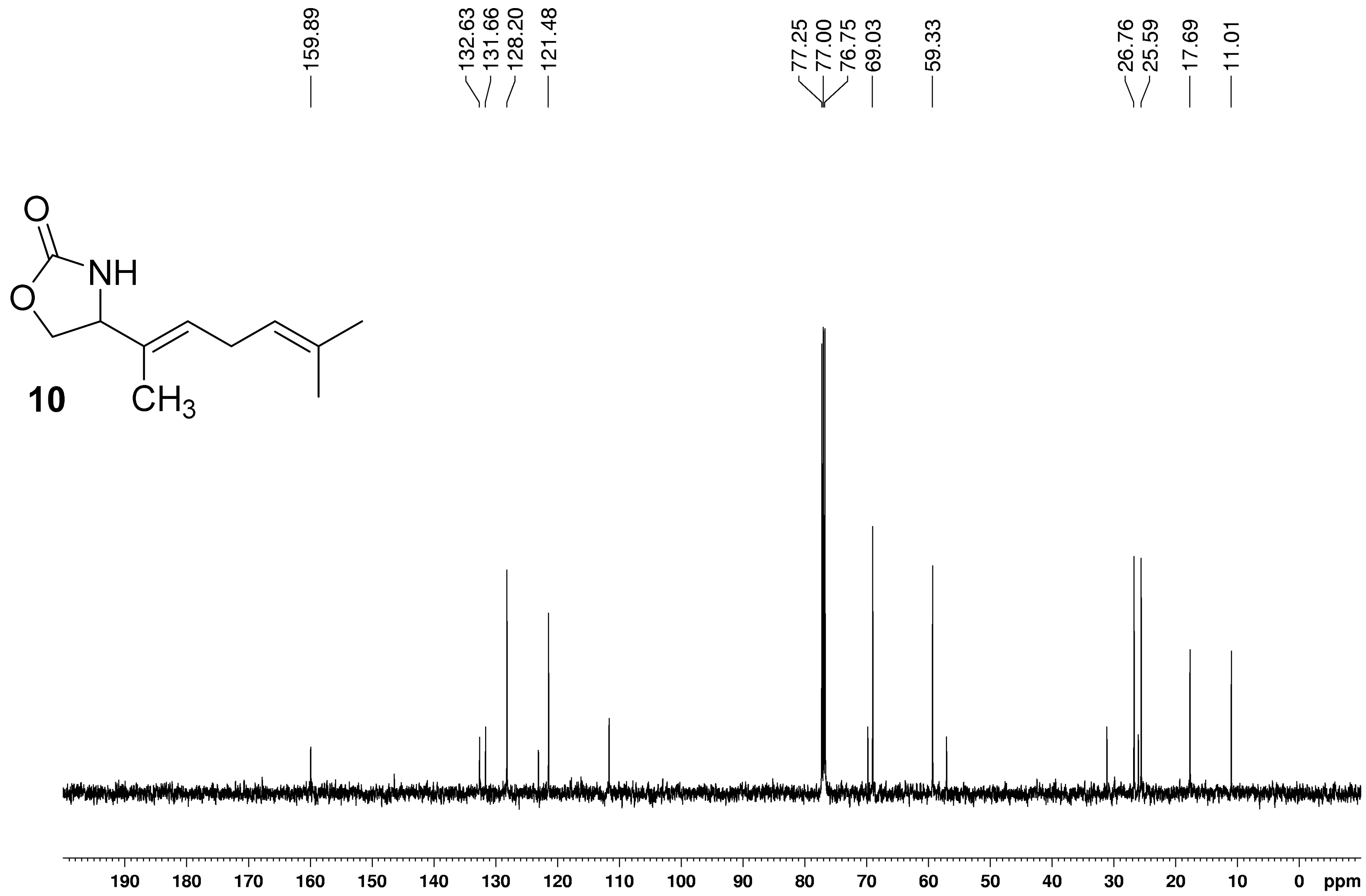

$^{13}C\{^{1}H\}$ NMR of compound **10** (126 MHz, $CDCl_3$)

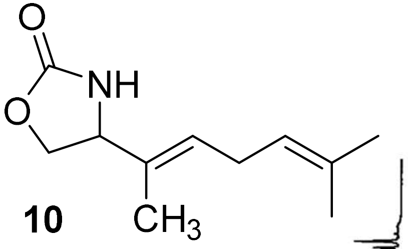

10

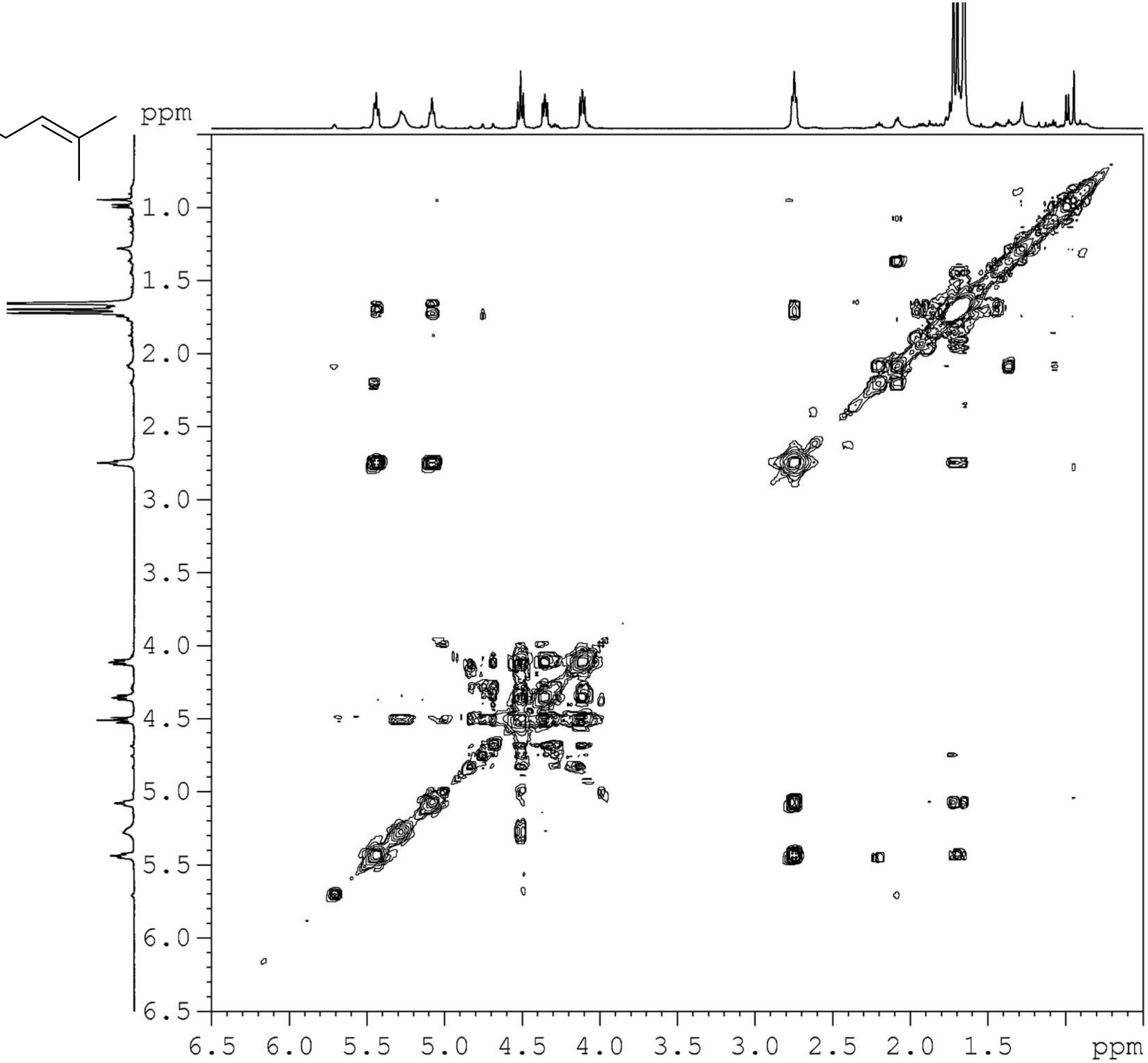

^{1}H-^{1}H COSY of compound **10** (500 MHz, $CDCl_3$)

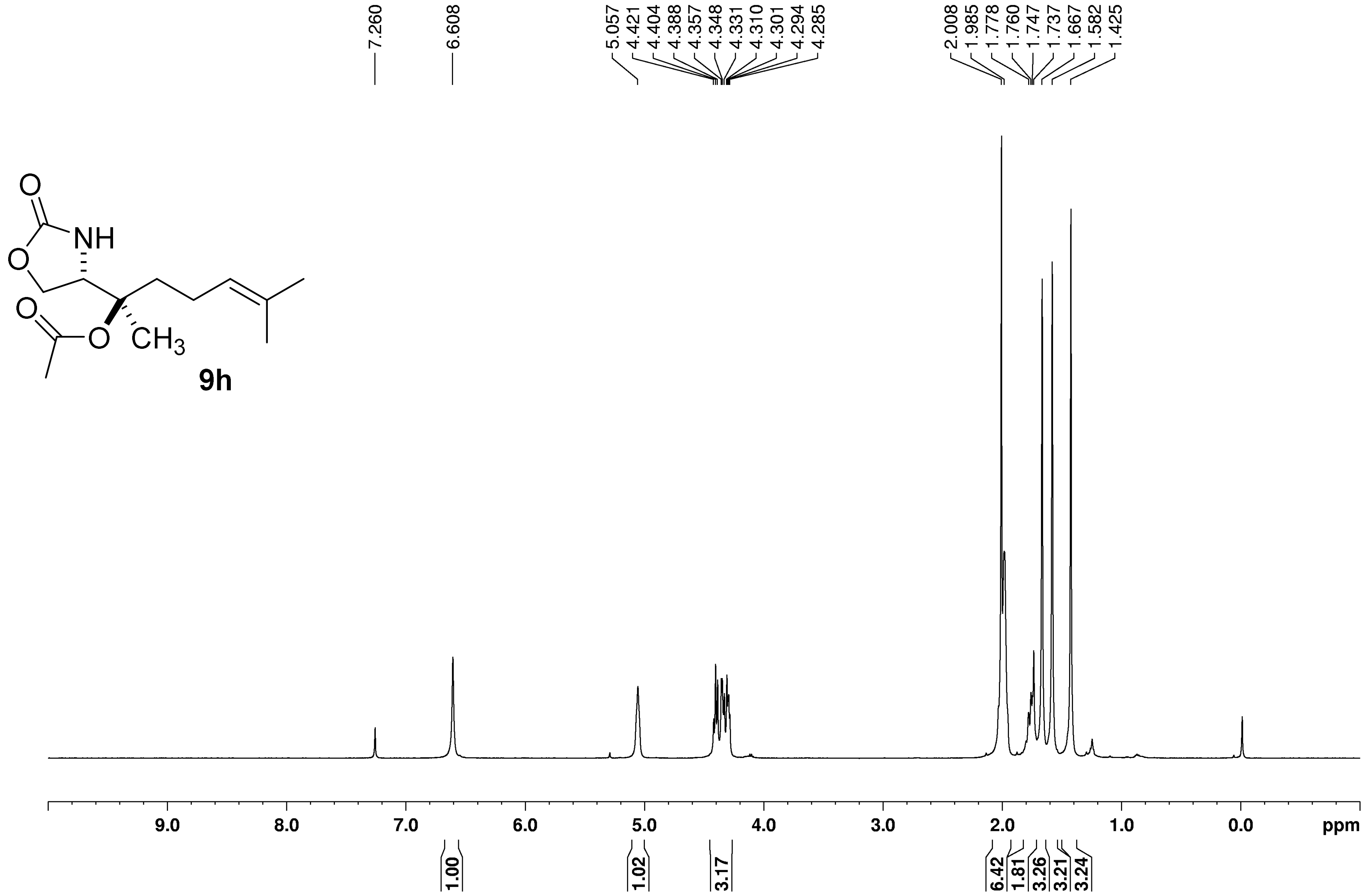

^{1}H NMR of compound **9h** (500 MHz, $CDCl_3$)

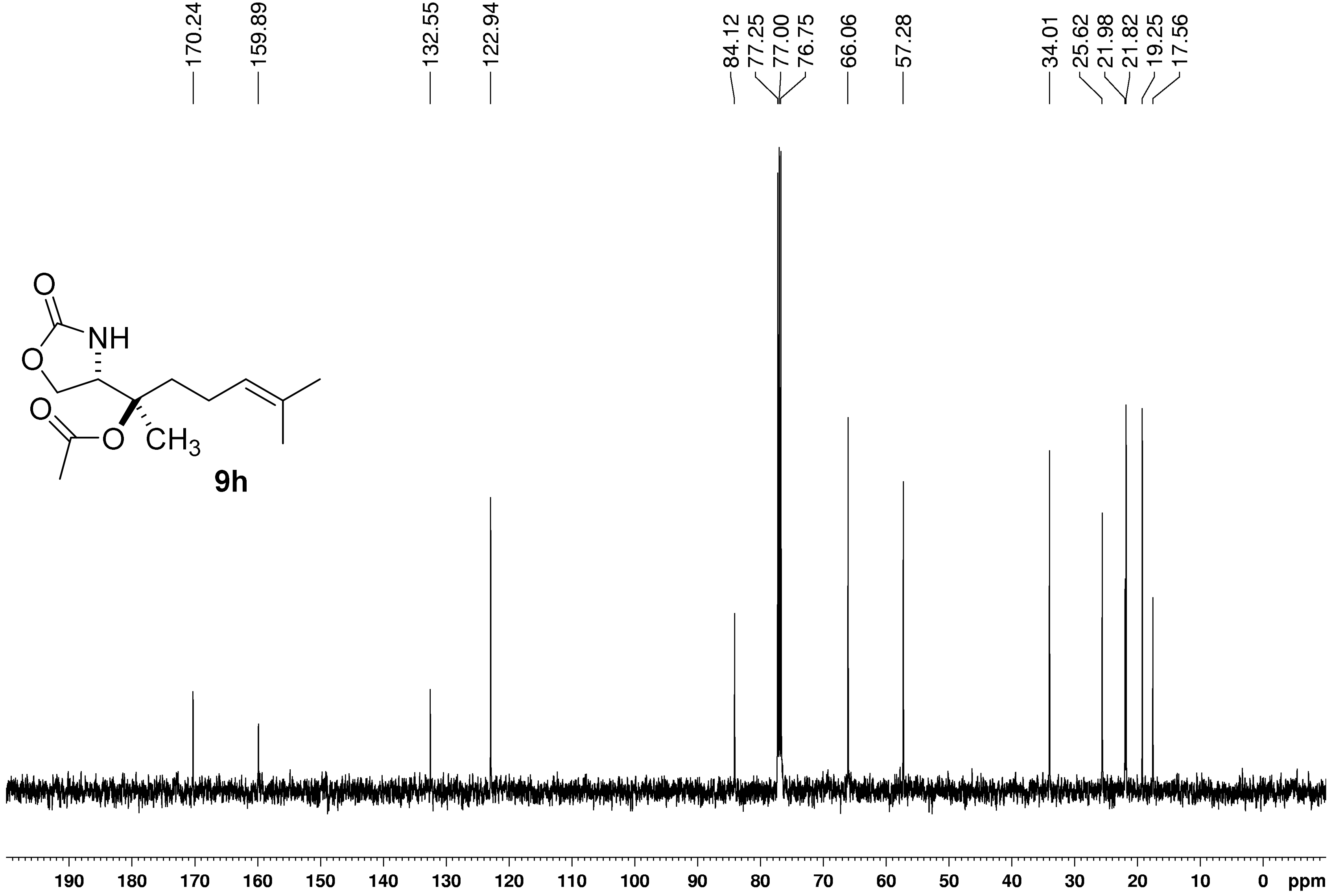

$^{13}C\{^{1}H\}$ NMR of compound **9h** (126 MHz, $CDCl_3$)

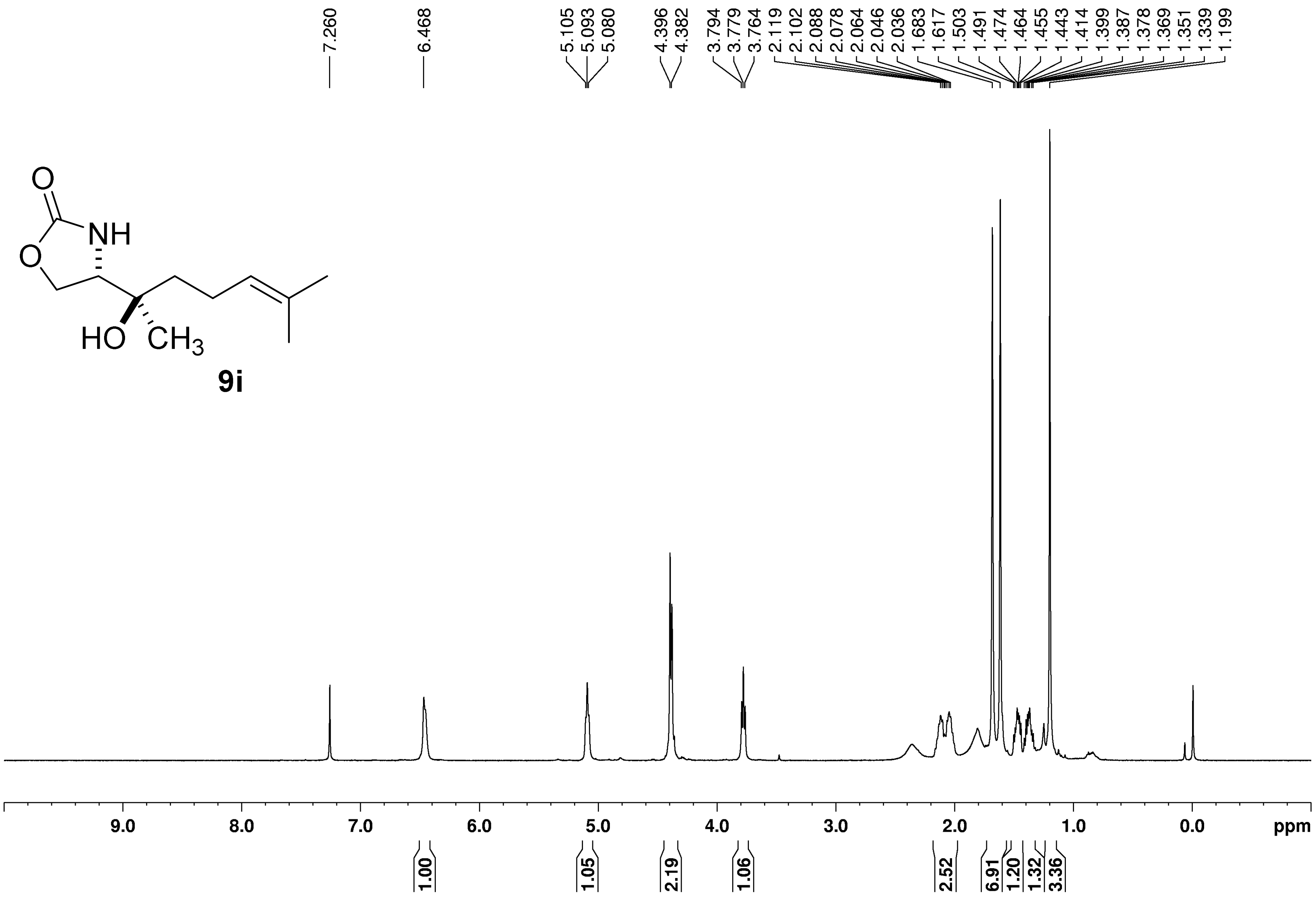

^{1}H NMR of compound **9i** (500 MHz, $CDCl_3$)

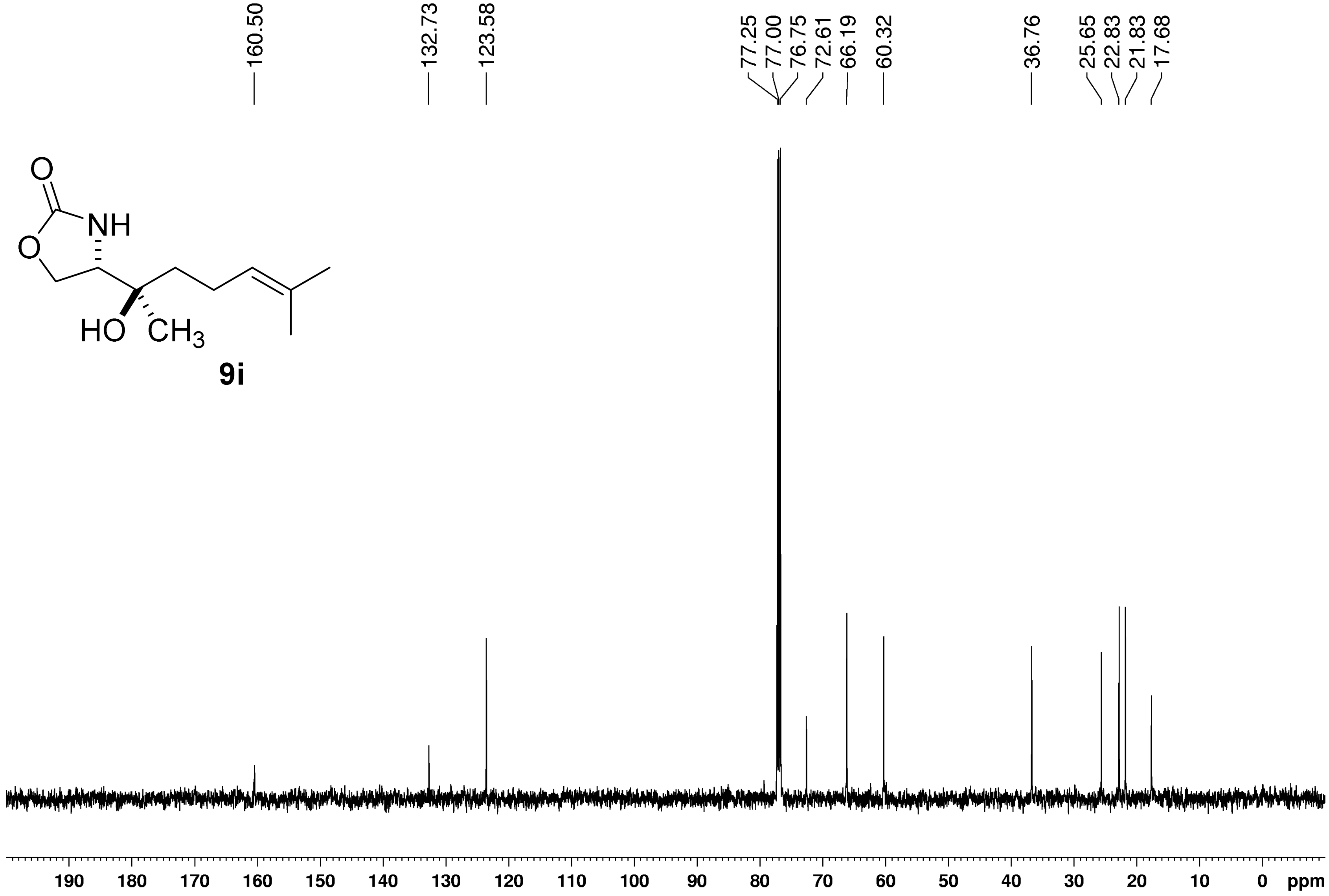

$^{13}C\{^{1}H\}$ NMR of compound **9i** (126 MHz, $CDCl_3$)

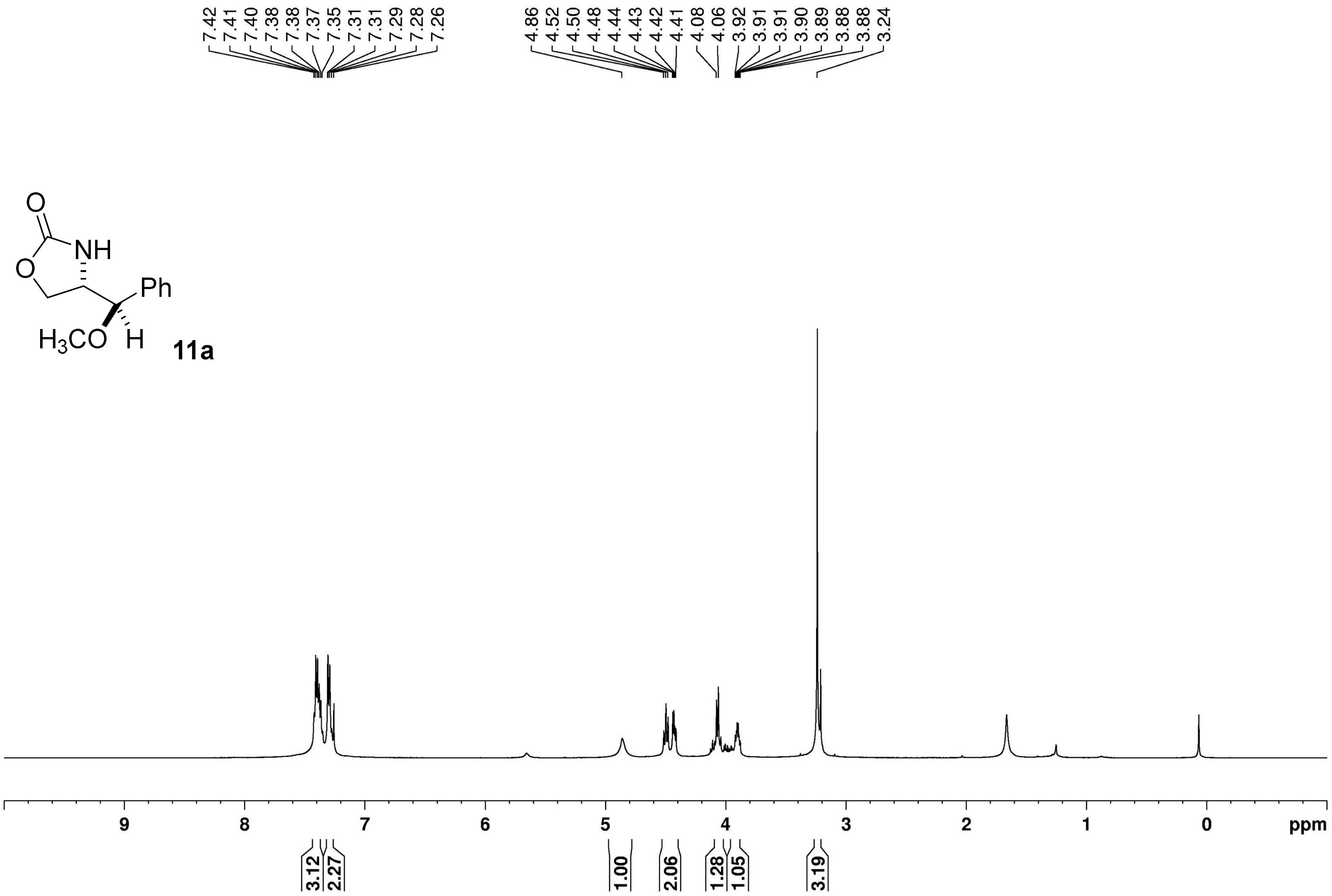

^{1}H NMR of compound **11a** (500 MHz, $CDCl_3$)

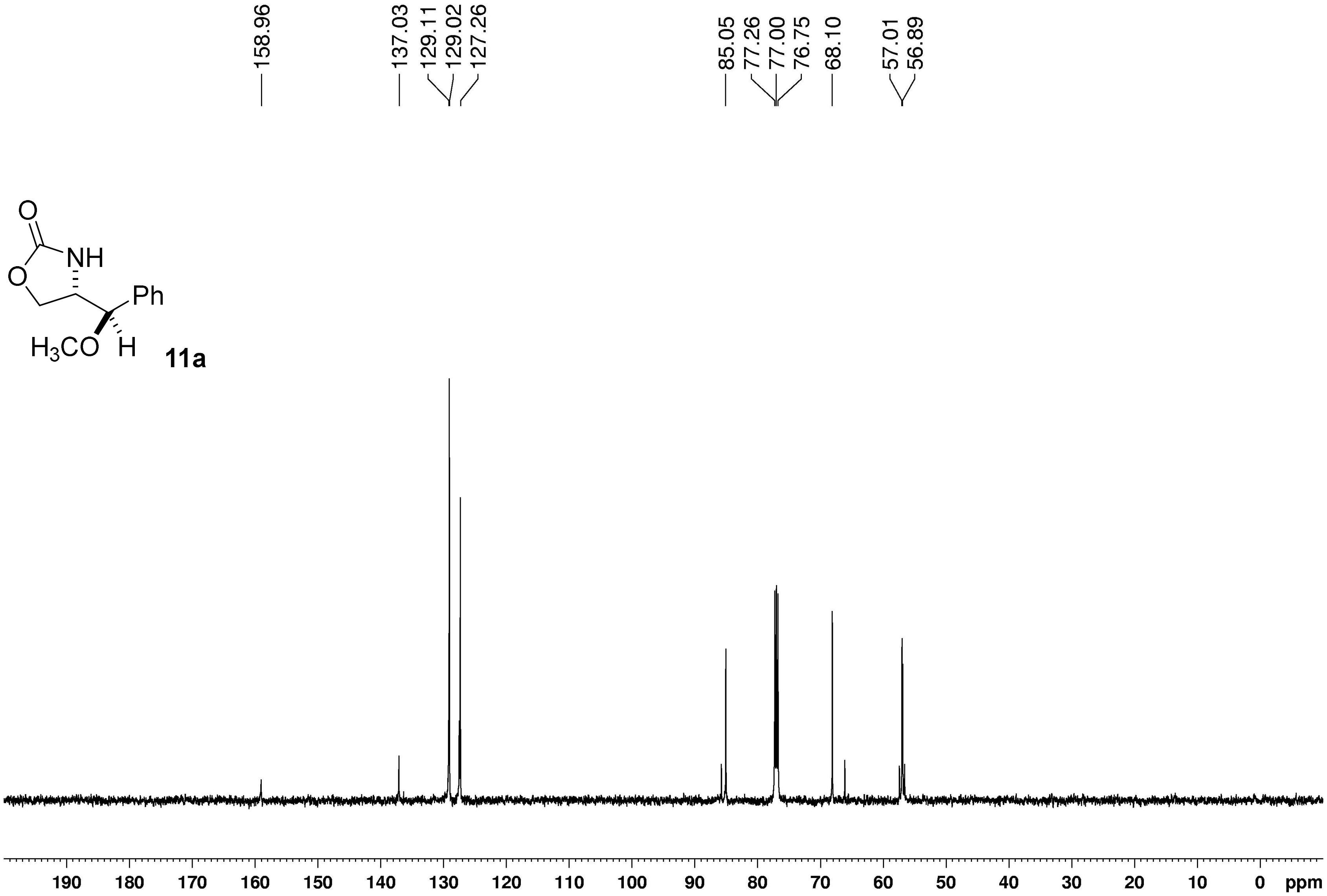

$^{13}C\{^1H\}$ NMR of compound **11a** (126 MHz, $CDCl_3$)

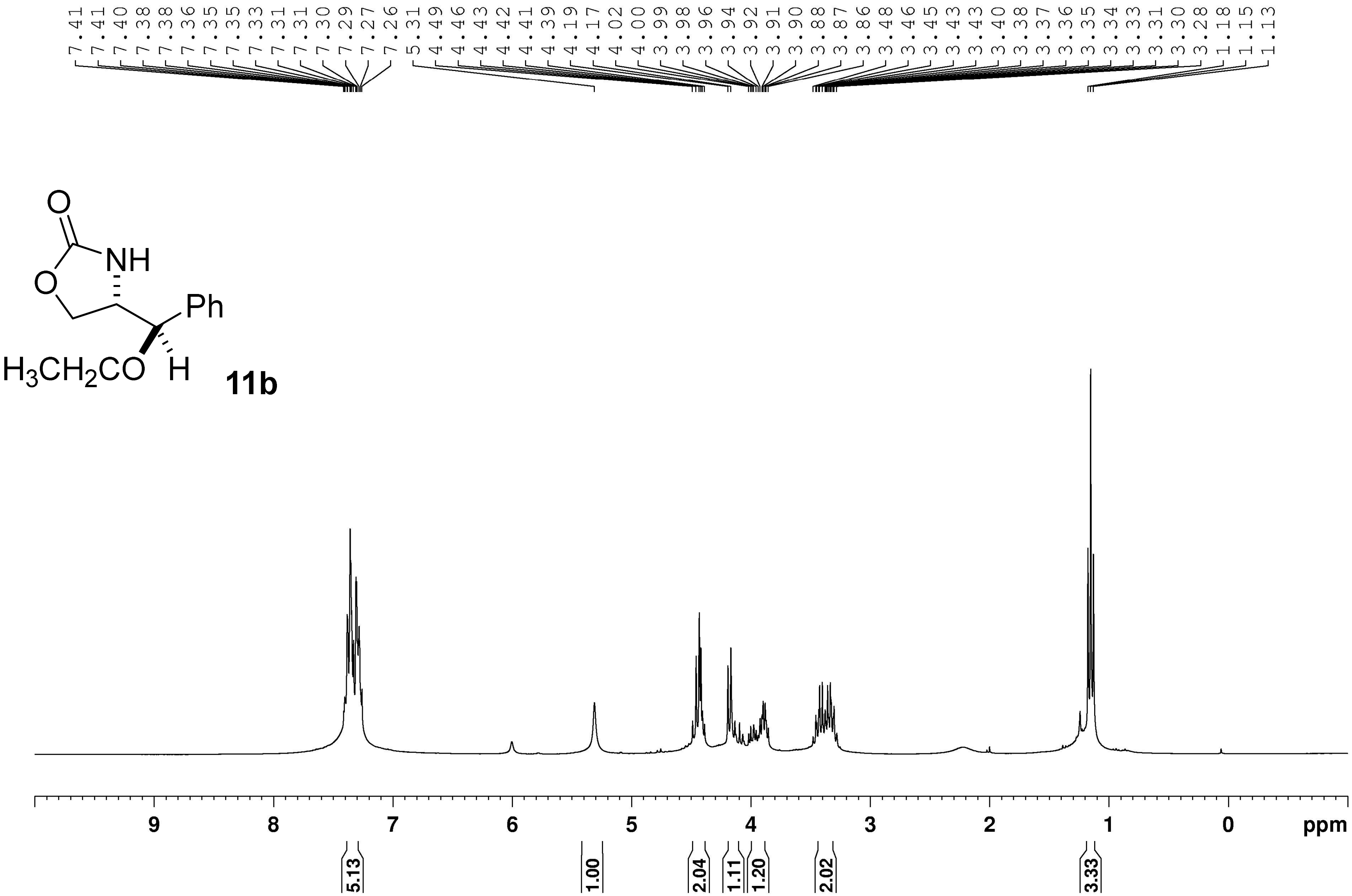

^{1}H NMR of compound **11b** (300 MHz, $CDCl_3$)

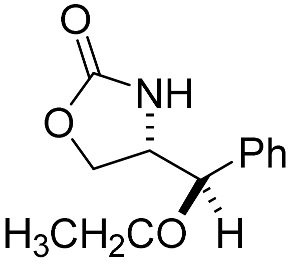

11b

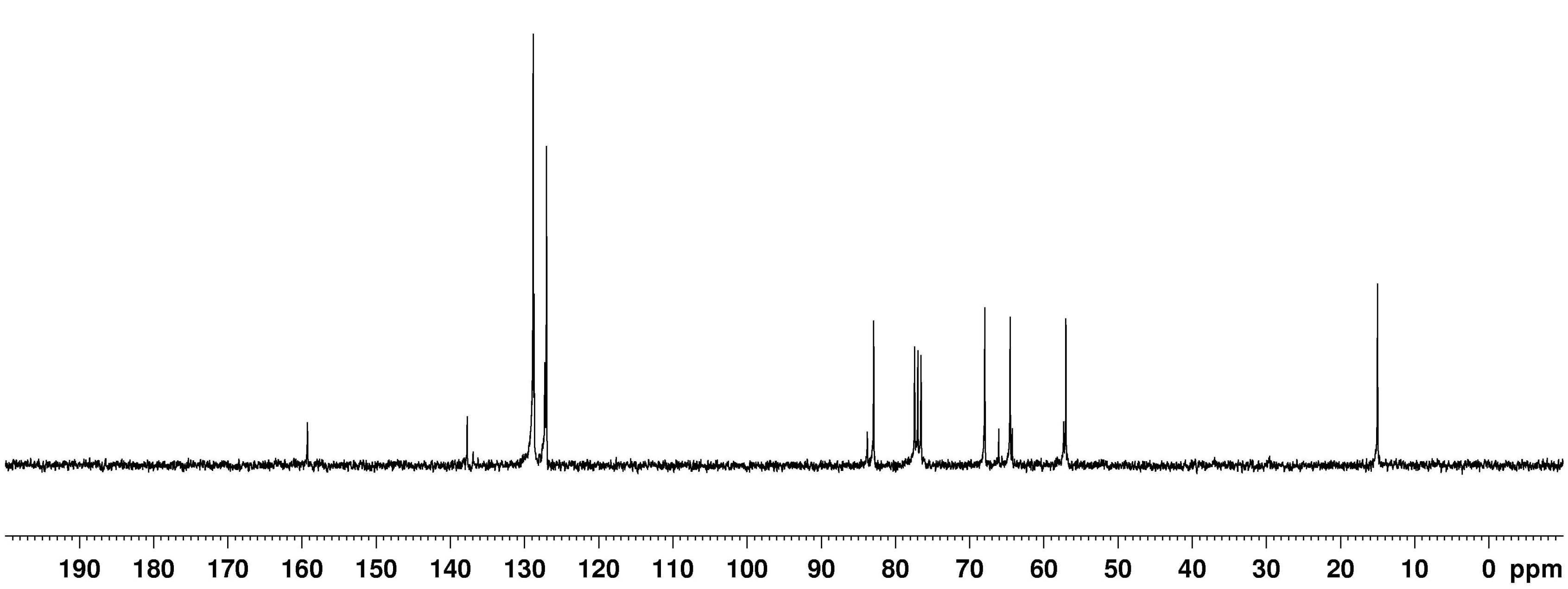

$^{13}C\{^{1}H\}$ NMR of compound **11b** (75 MHz, $CDCl_3$)

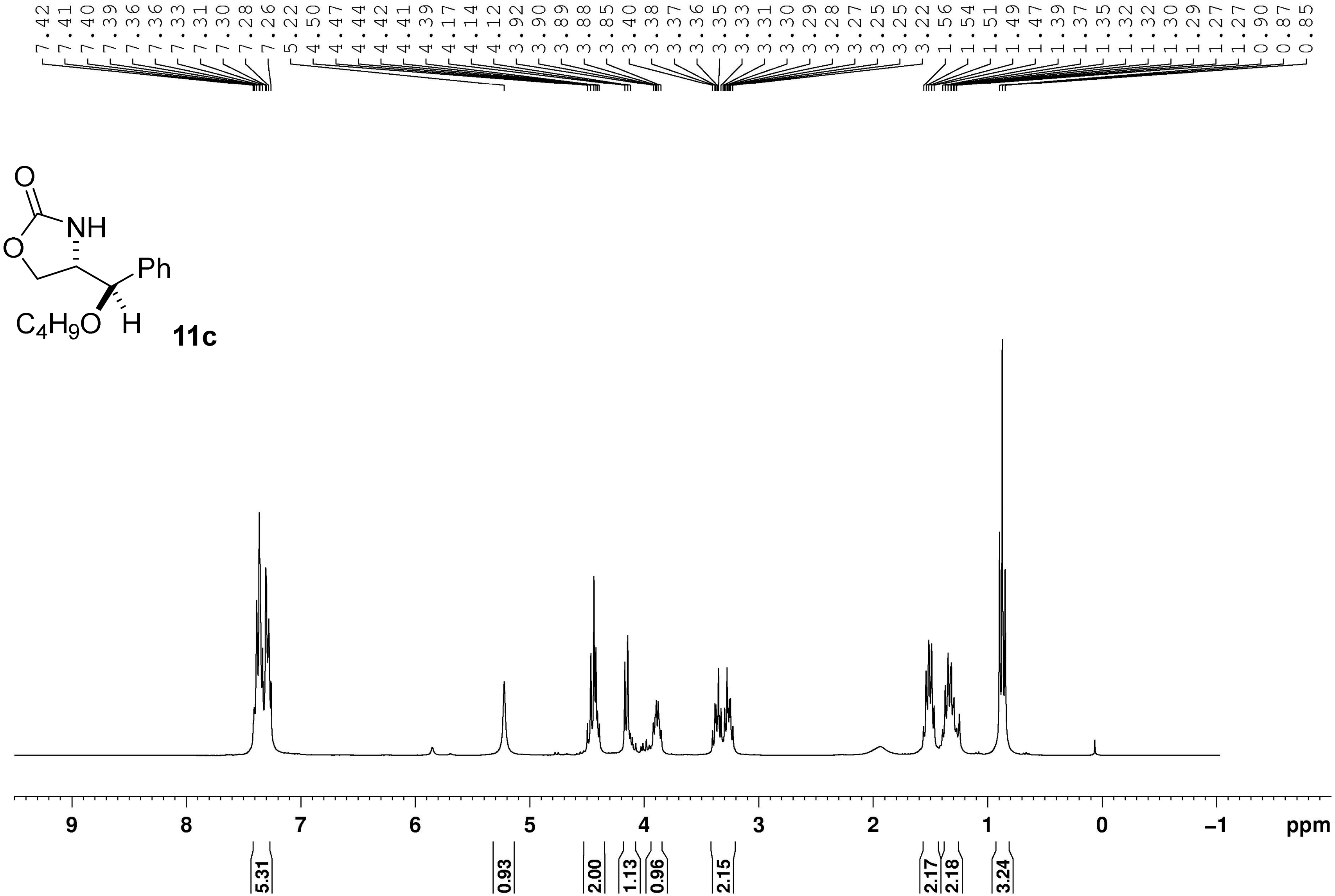

^{1}H NMR of compound **11c** (300 MHz, $CDCl_3$)

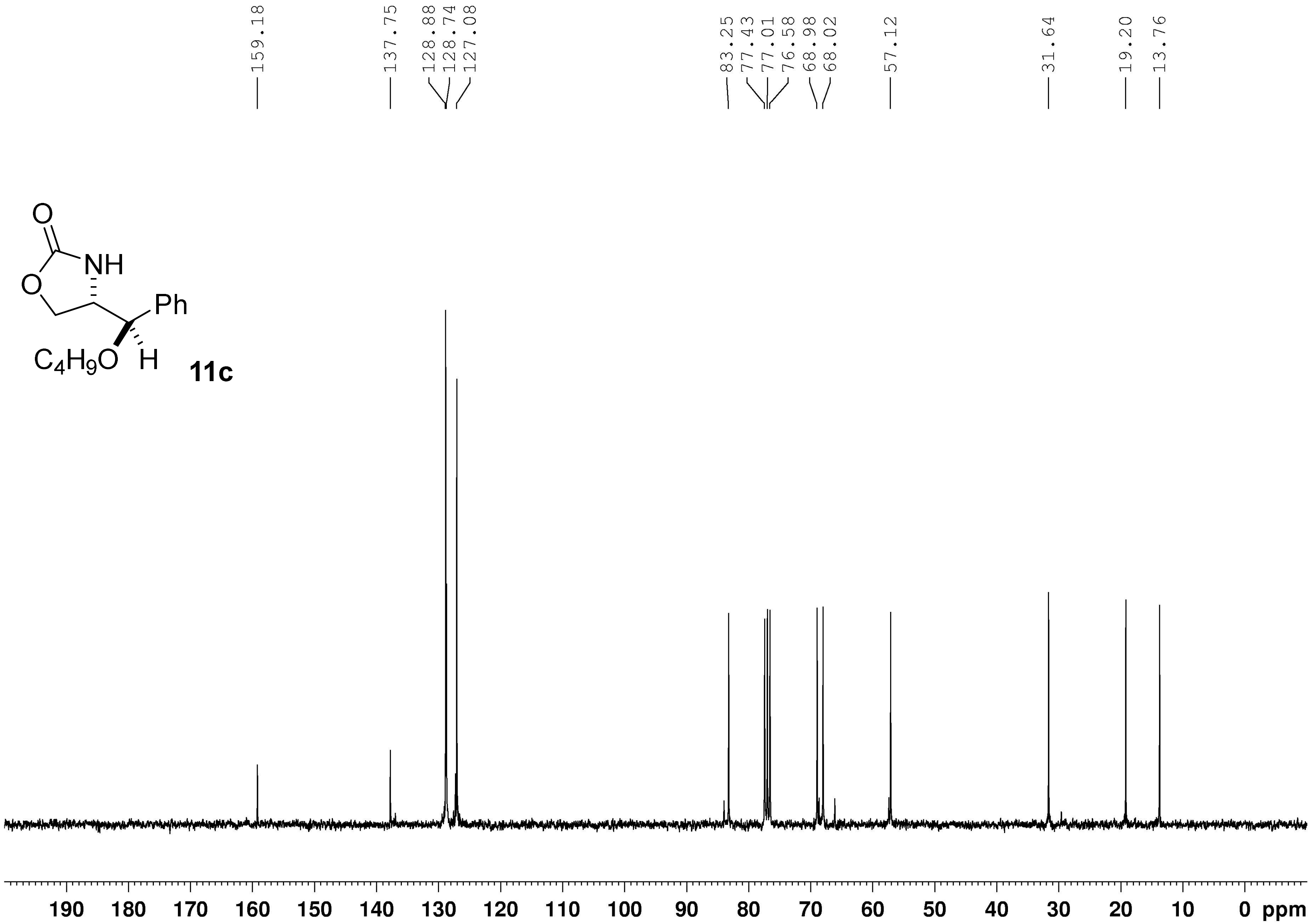

$^{13}C\{^{1}H\}$ NMR of compound **11c** (75 MHz, $CDCl_3$)

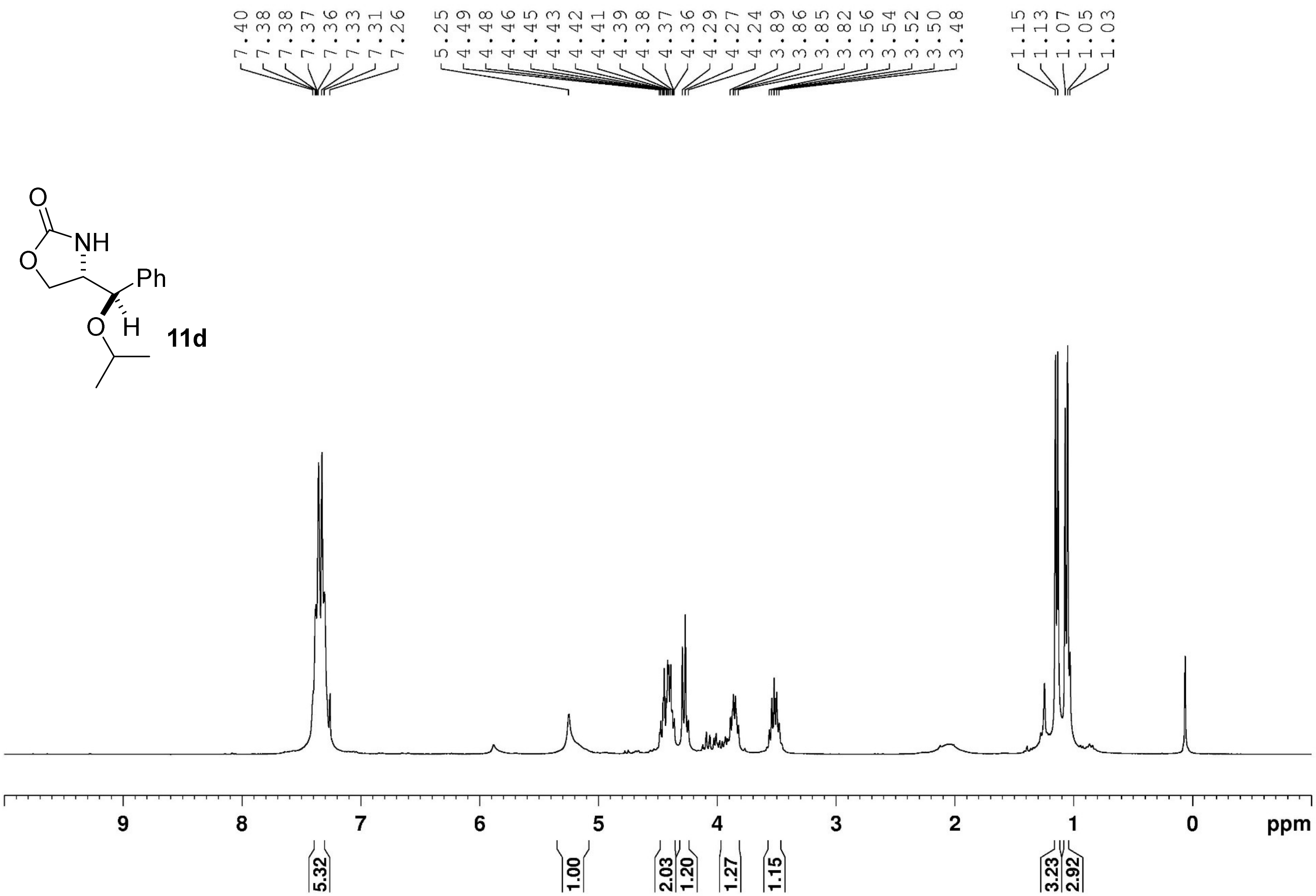

^{1}H NMR of compound **11d** (300 MHz, $CDCl_3$)

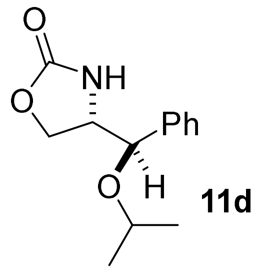

11d

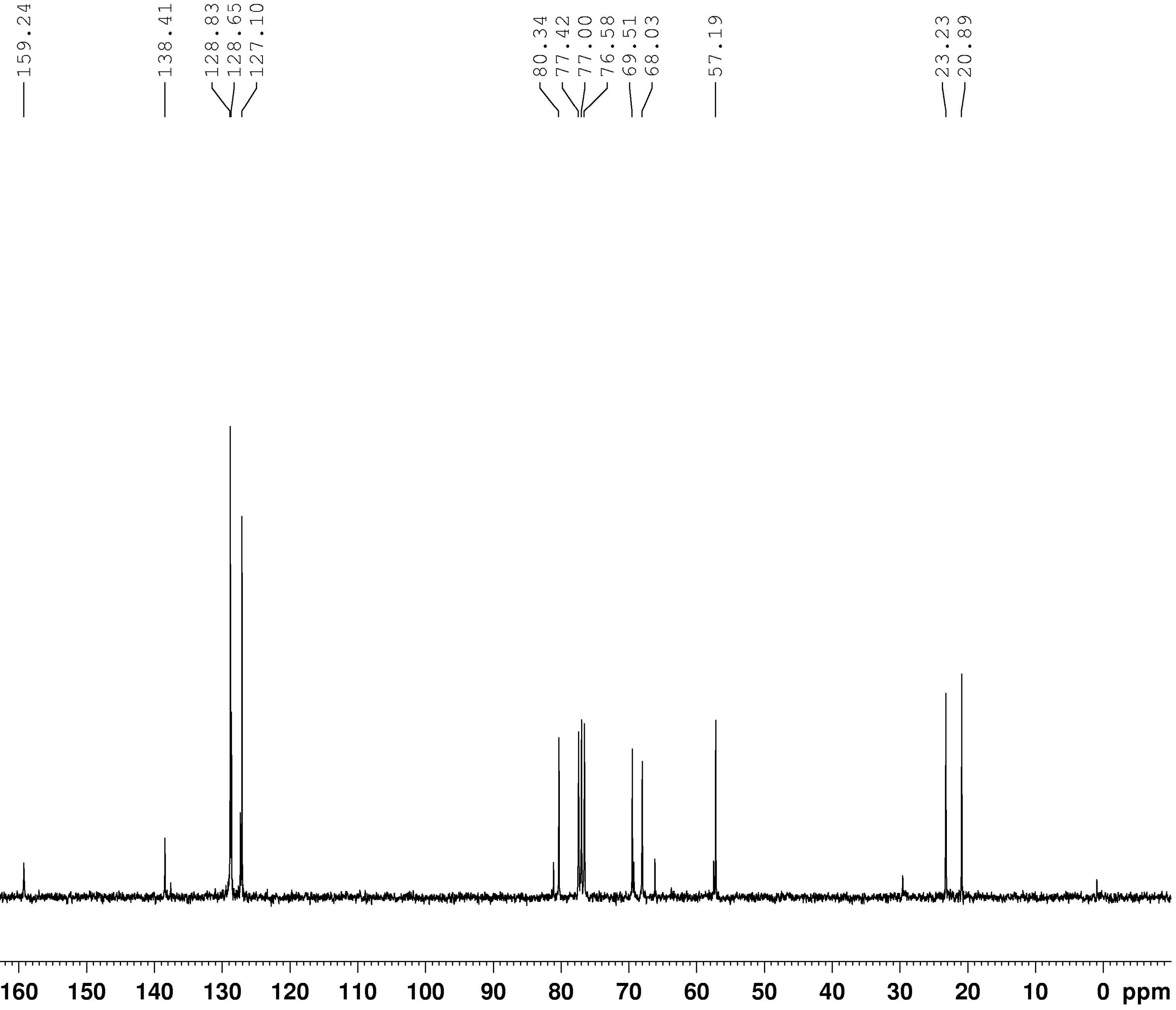

$^{13}C\{^1H\}$ NMR of compound **11d** (75 MHz, $CDCl_3$)

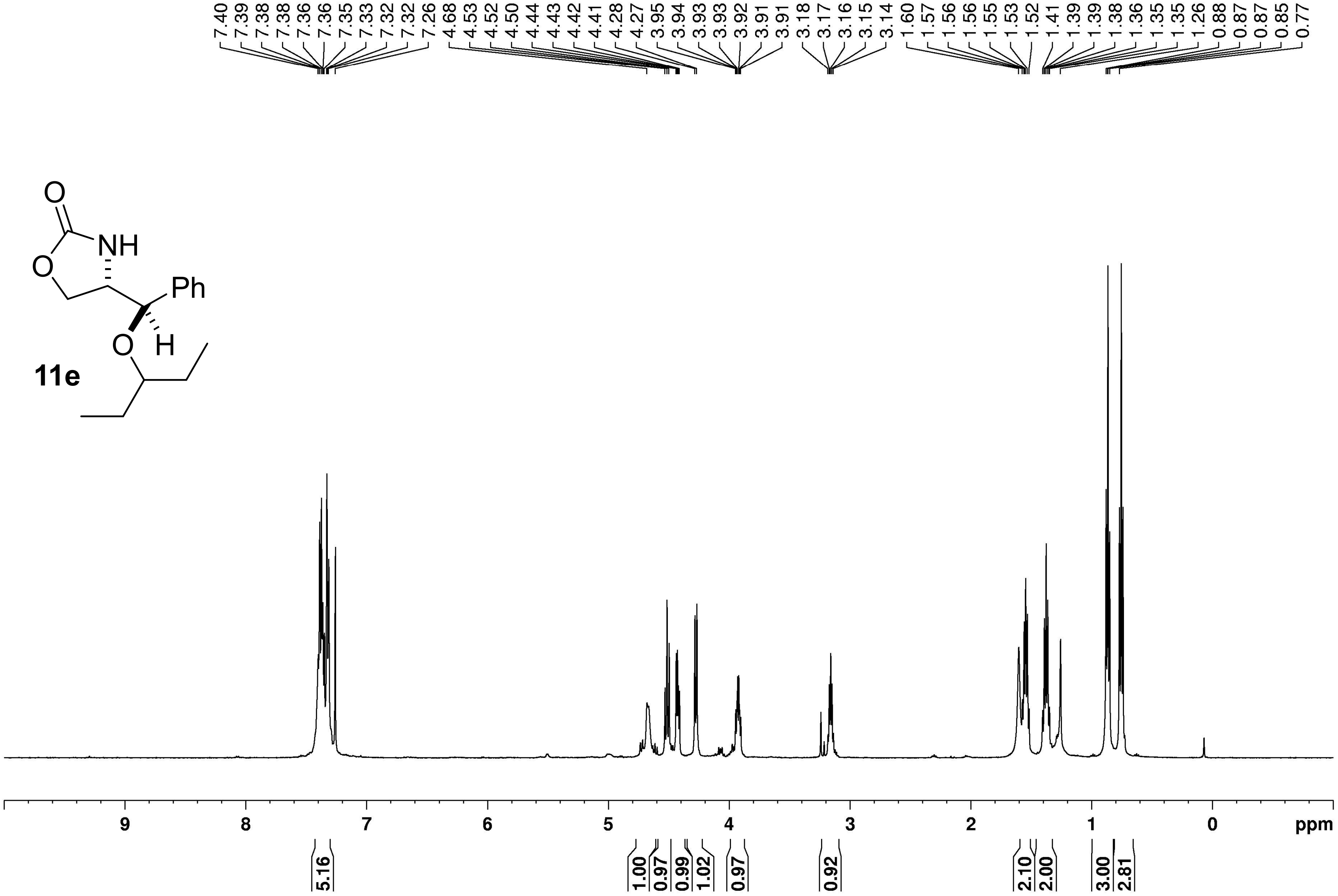

^{1}H NMR of compound **11e** (500 MHz, $CDCl_3$)

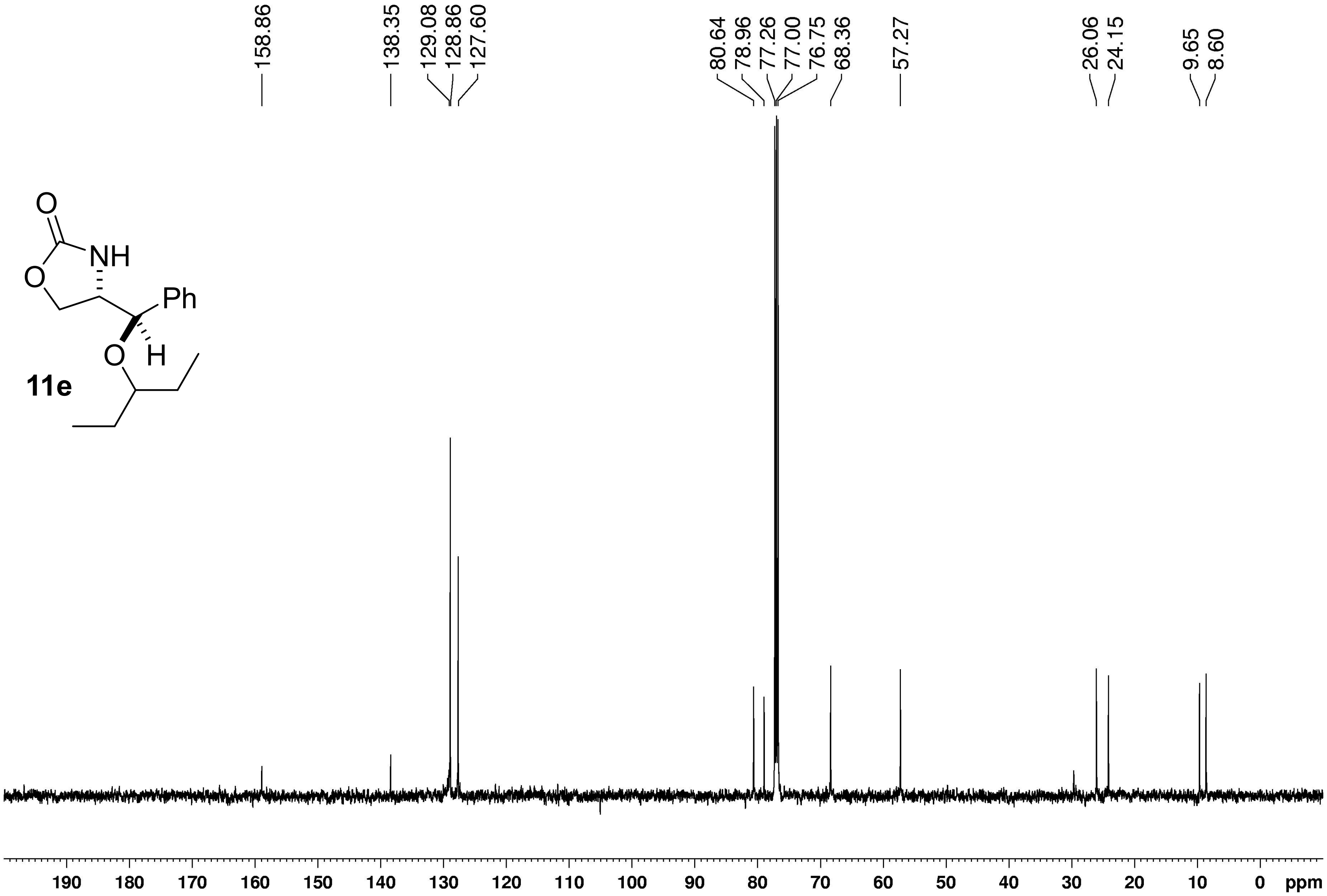

$^{13}C\{^{1}H\}$ NMR of compound **11e** NMR (126 MHz, $CDCl_3$)

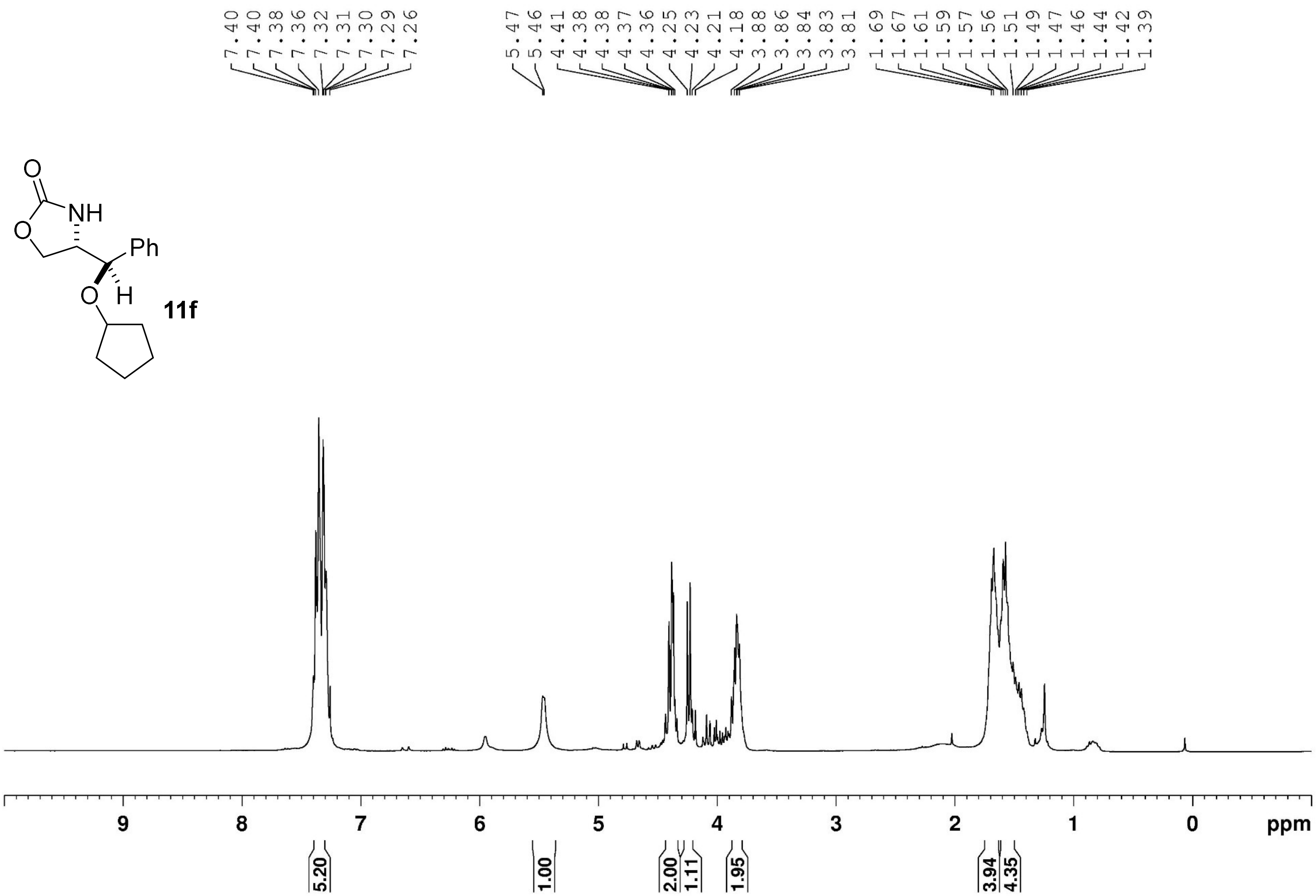

^{1}H NMR of compound **11f** (300 MHz, $CDCl_3$)

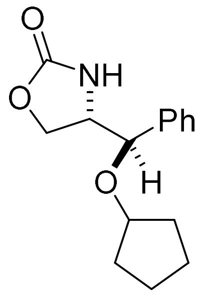

11f

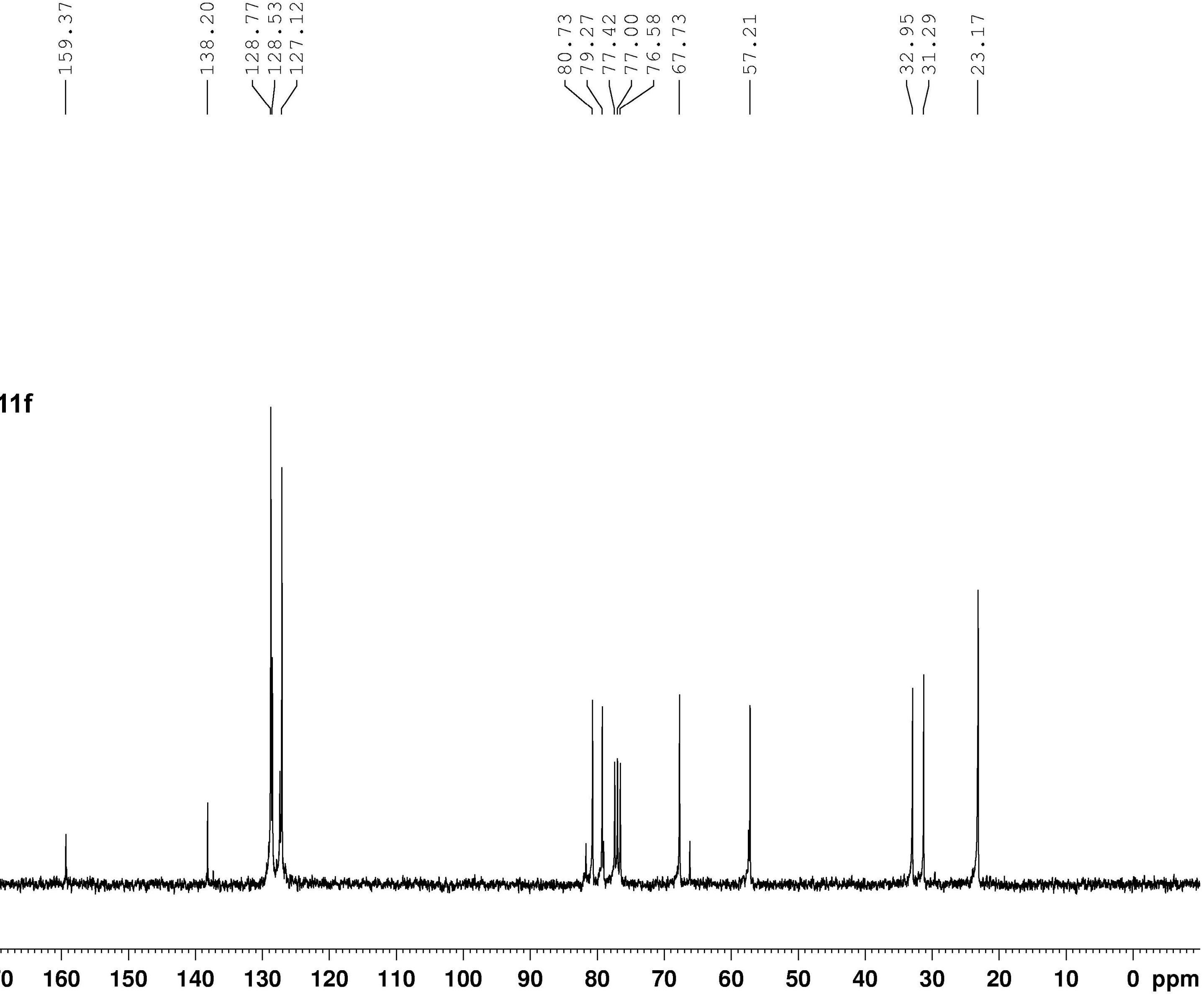

$^{13}C\{^1H\}$ NMR of compound **11f** (75 MHz, $CDCl_3$)

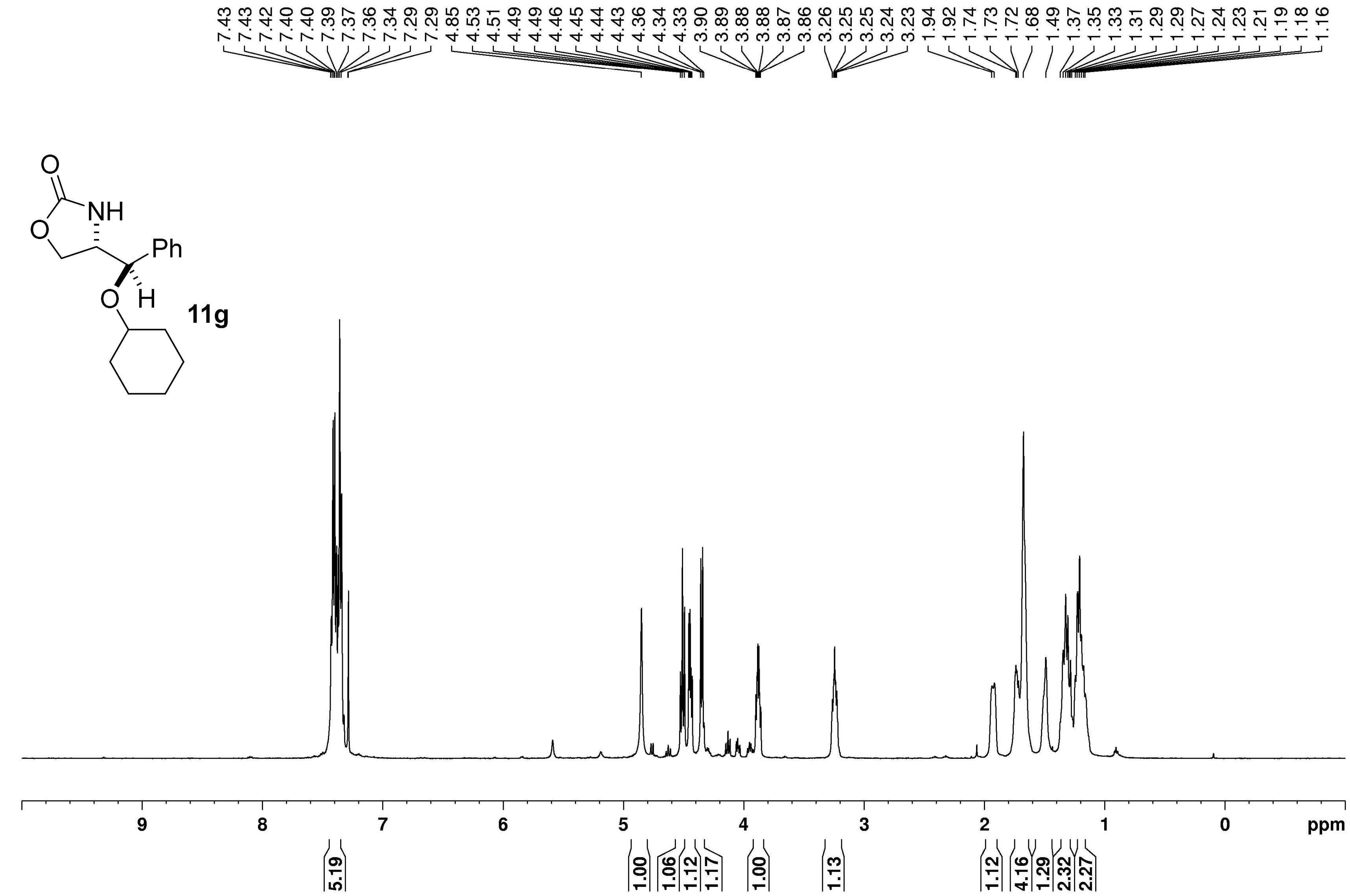

^{1}H NMR of compound **11g** (500 MHz, $CDCl_3$)

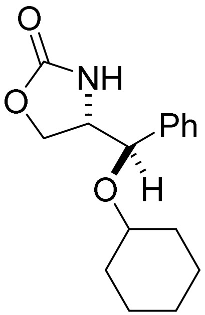

11g

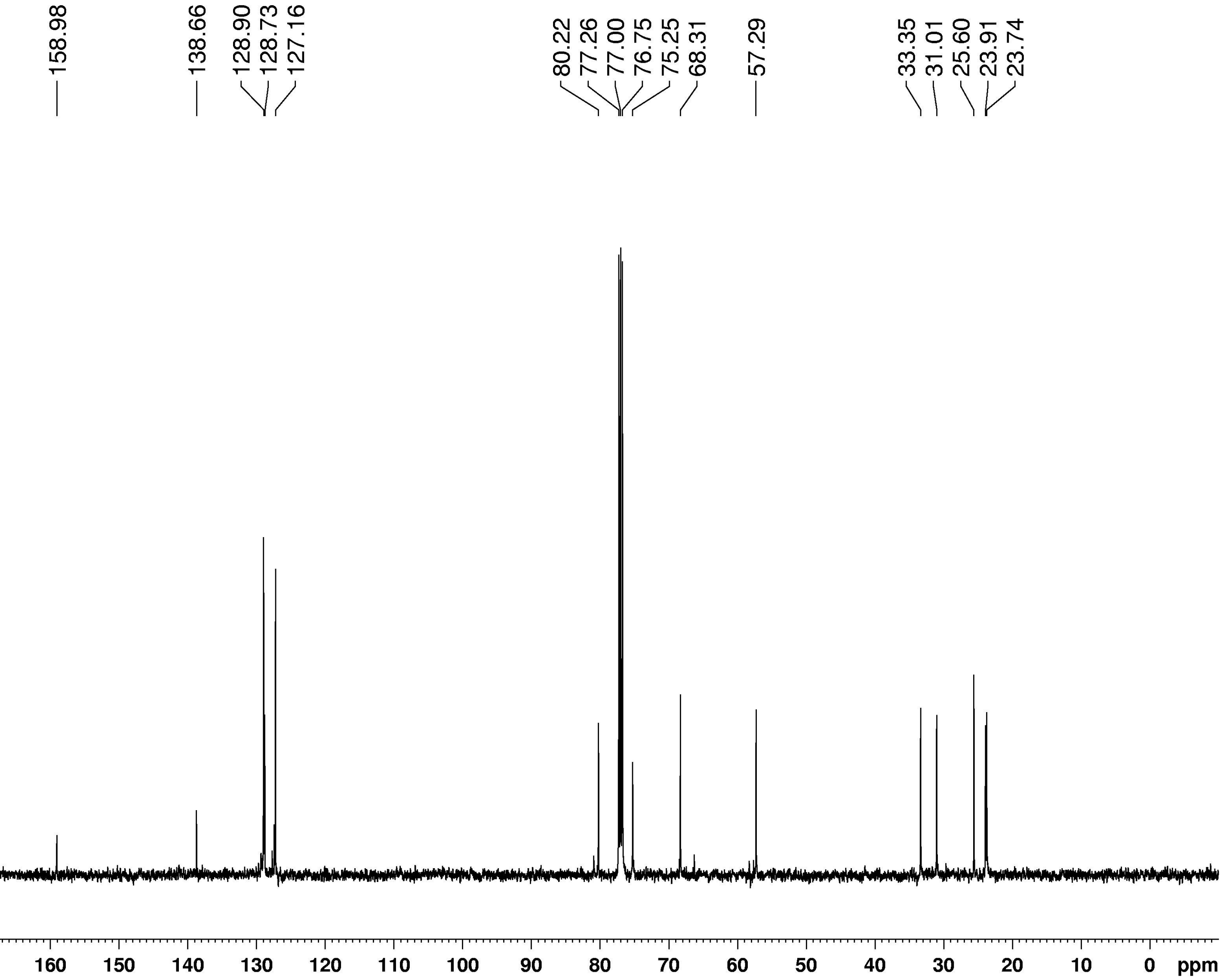

$^{13}C\{^{1}H\}$ NMR of compound **11g** (126 MHz, $CDCl_3$)

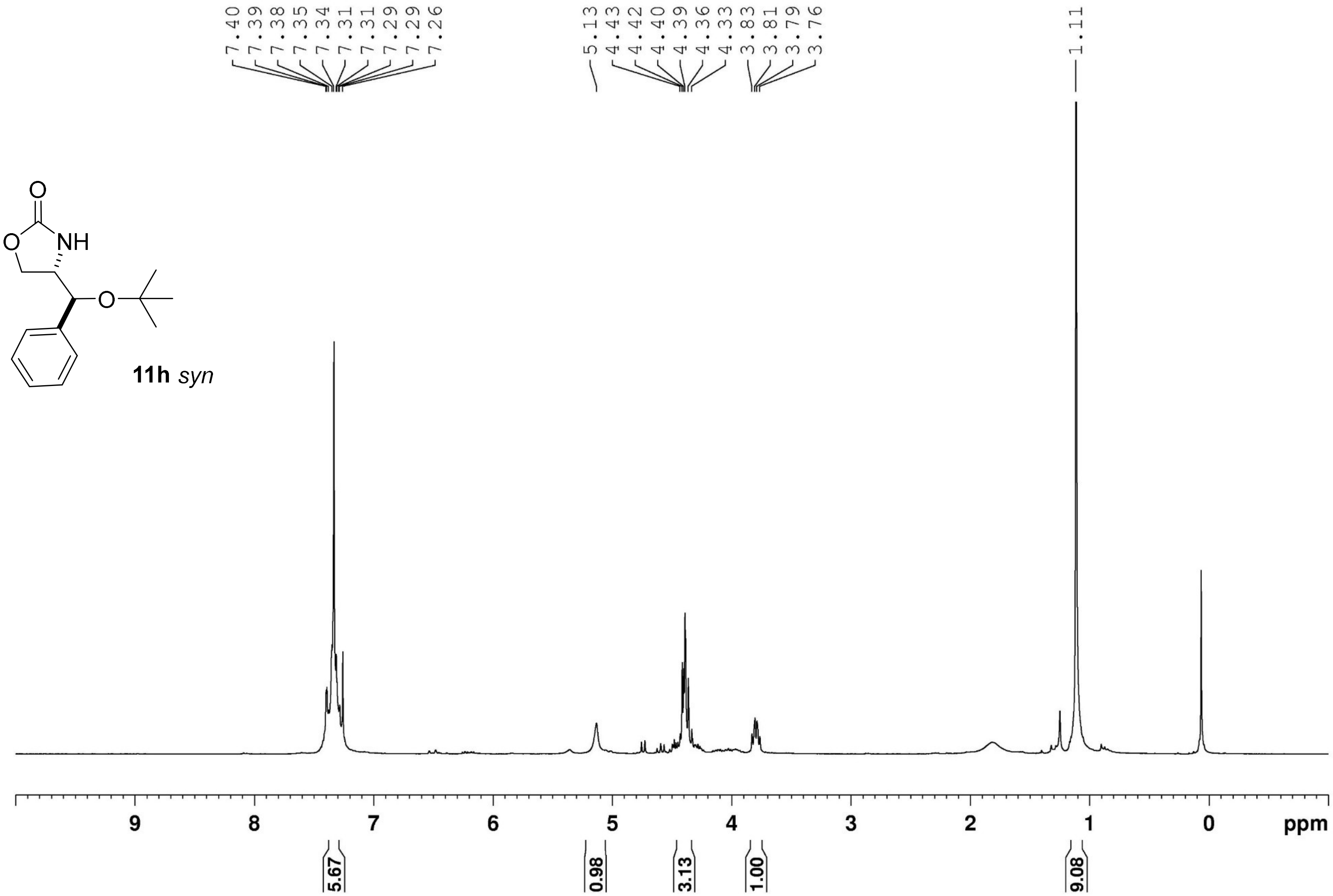

^{1}H NMR of compound **11h** *syn* (300 MHz, $CDCl_3$)

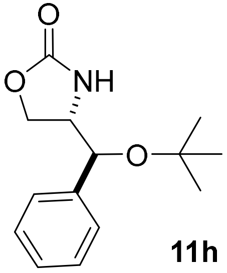

11h *syn*

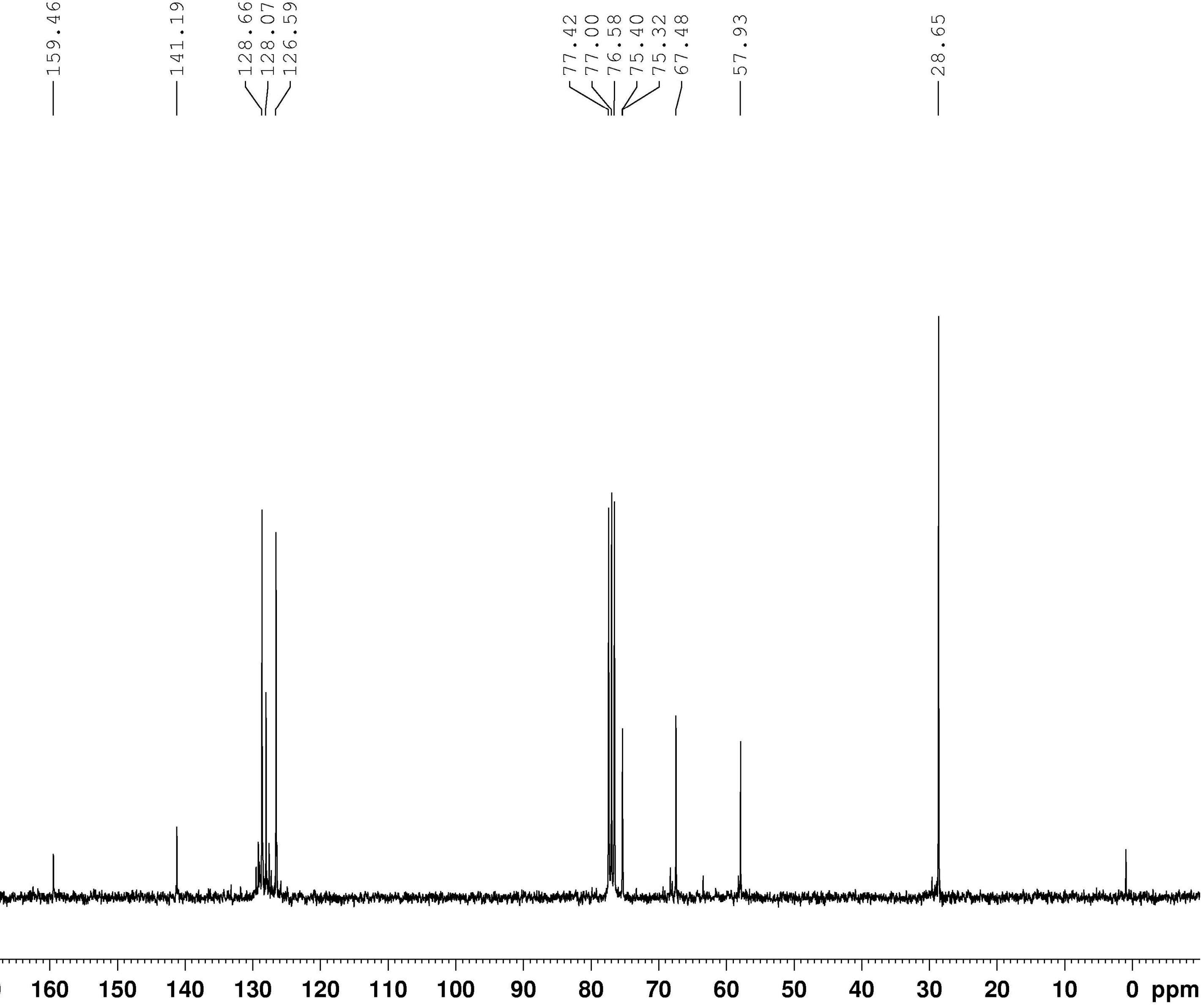

$^{13}C\{^{1}H\}$ NMR of compound **11h** *syn* (75 MHz, $CDCl_3$)

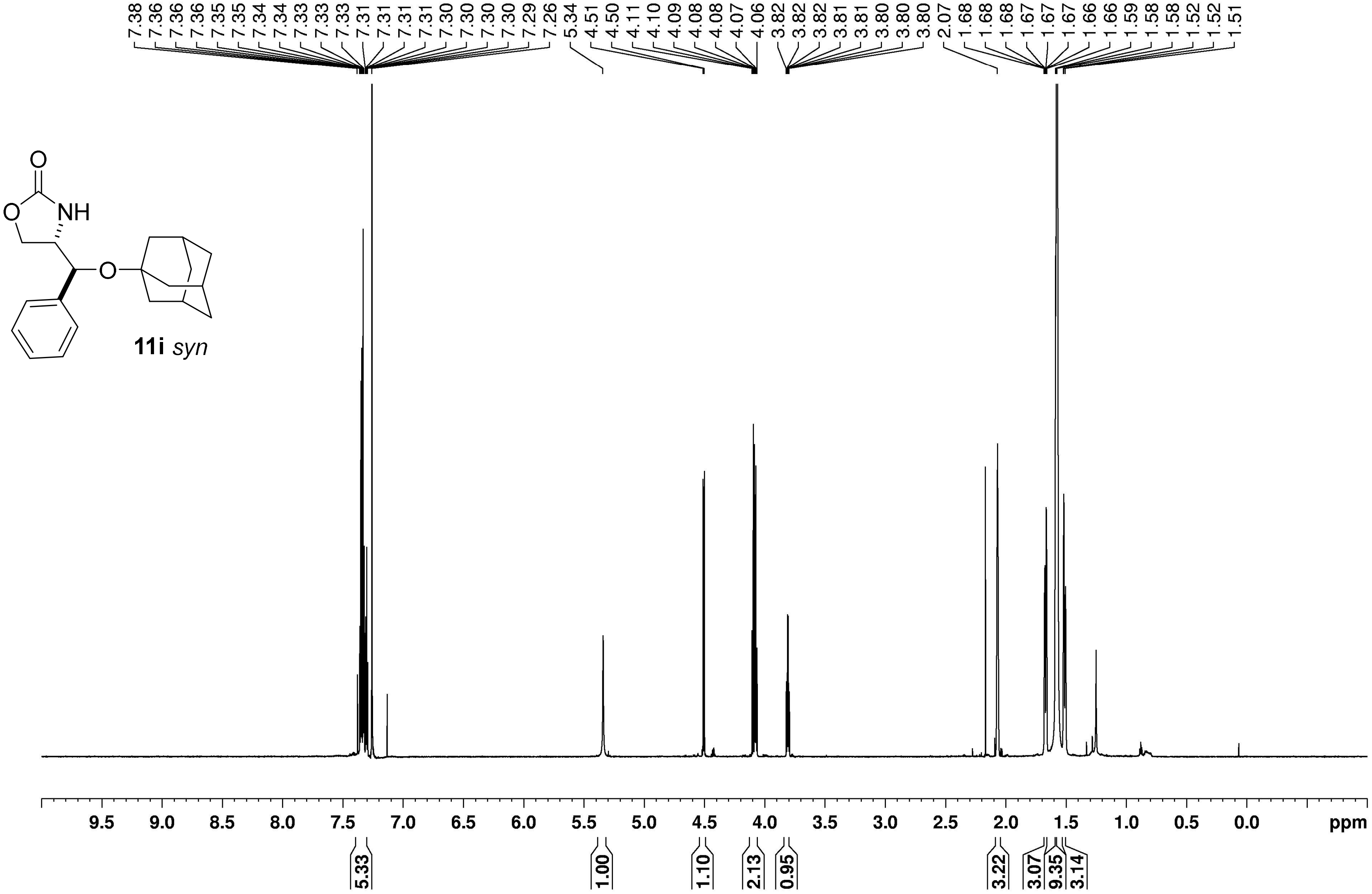

^{1}H NMR of compound **11i** *syn* (850 MHz , $CDCl_3$)

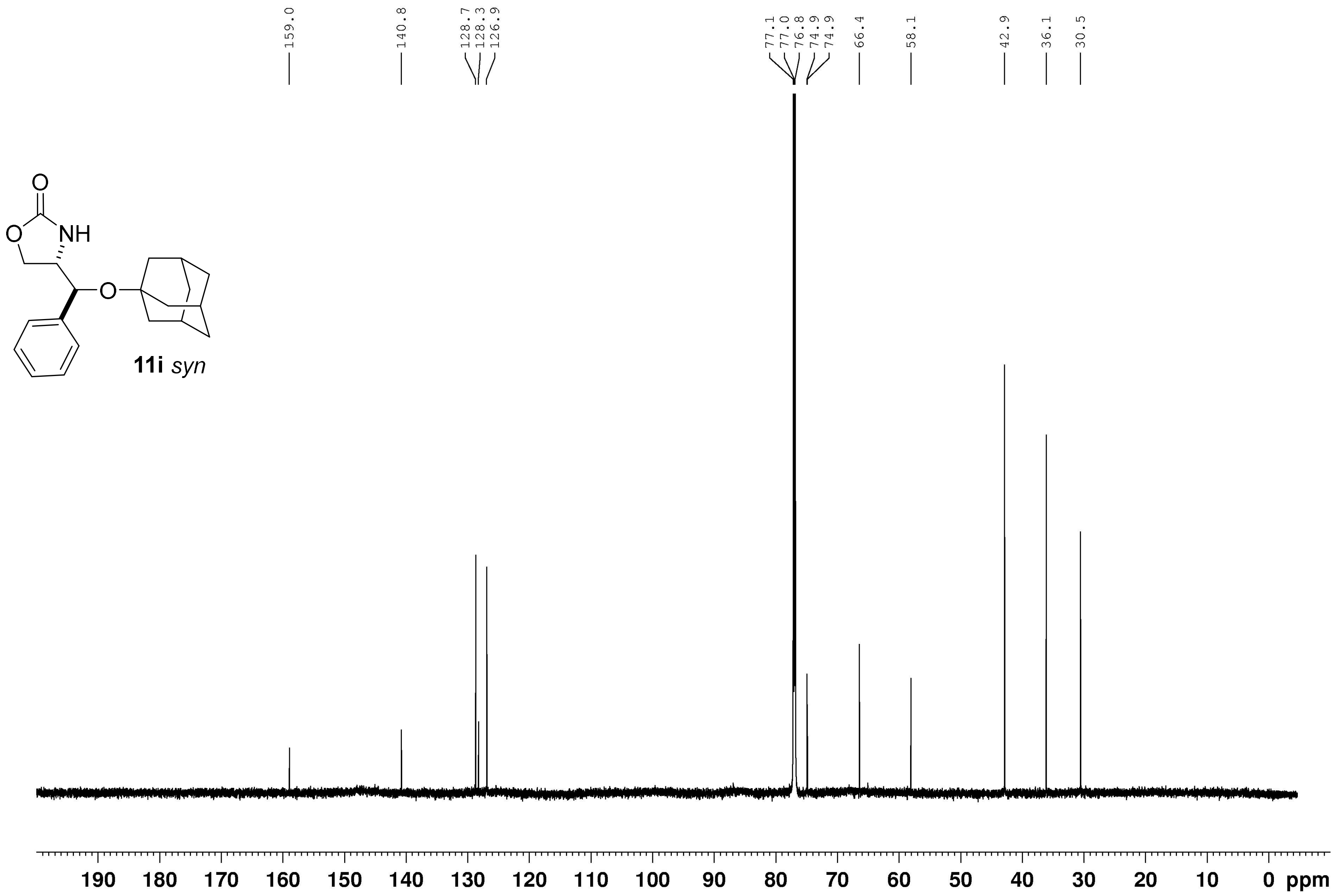

$^{13}C\{^{1}H\}$ NMR of compound **11i** *syn* (213 MHz, $CDCl_3$)

13

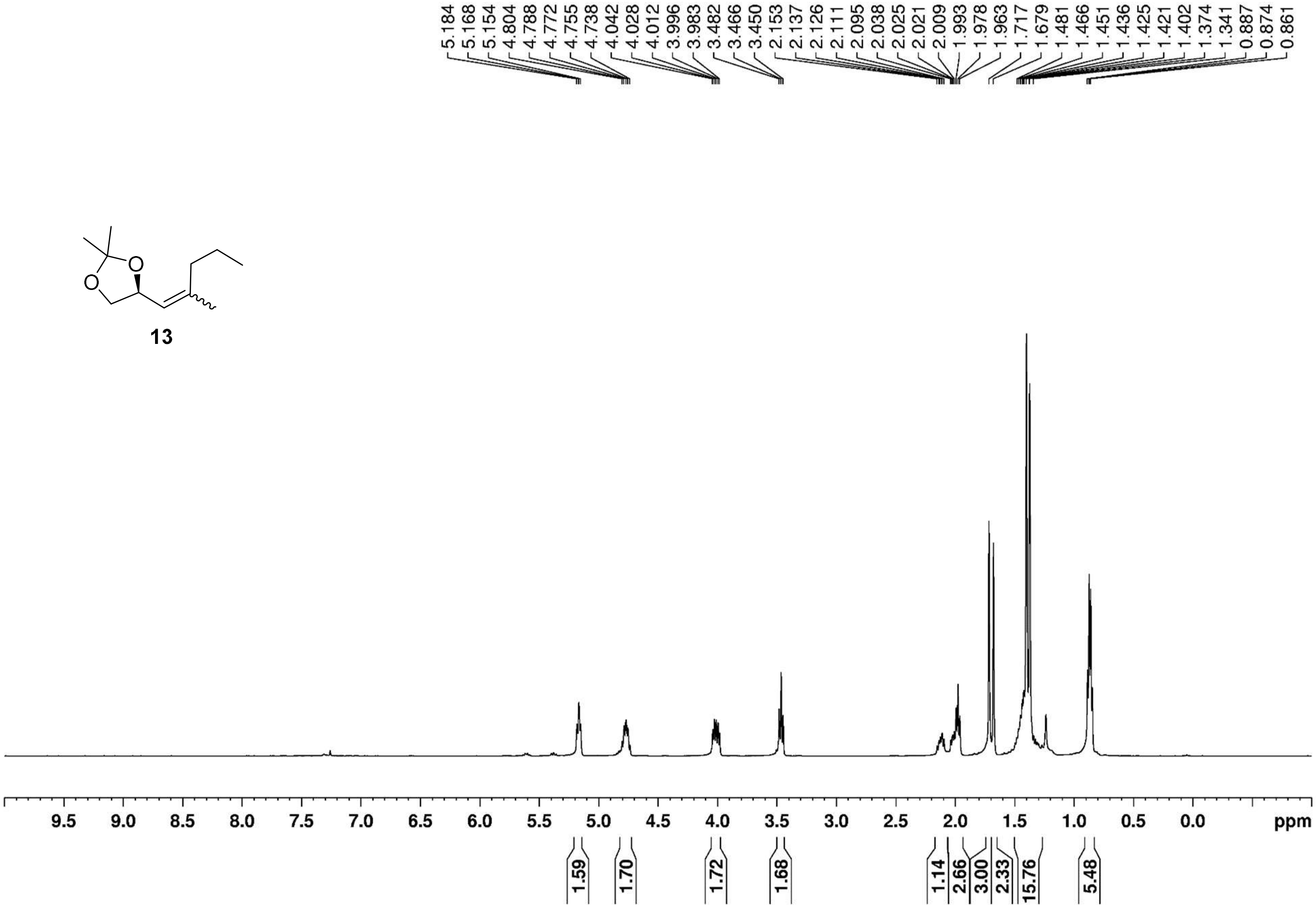

^{1}H NMR of compound **13** (500 MHz, $CDCl_3$)

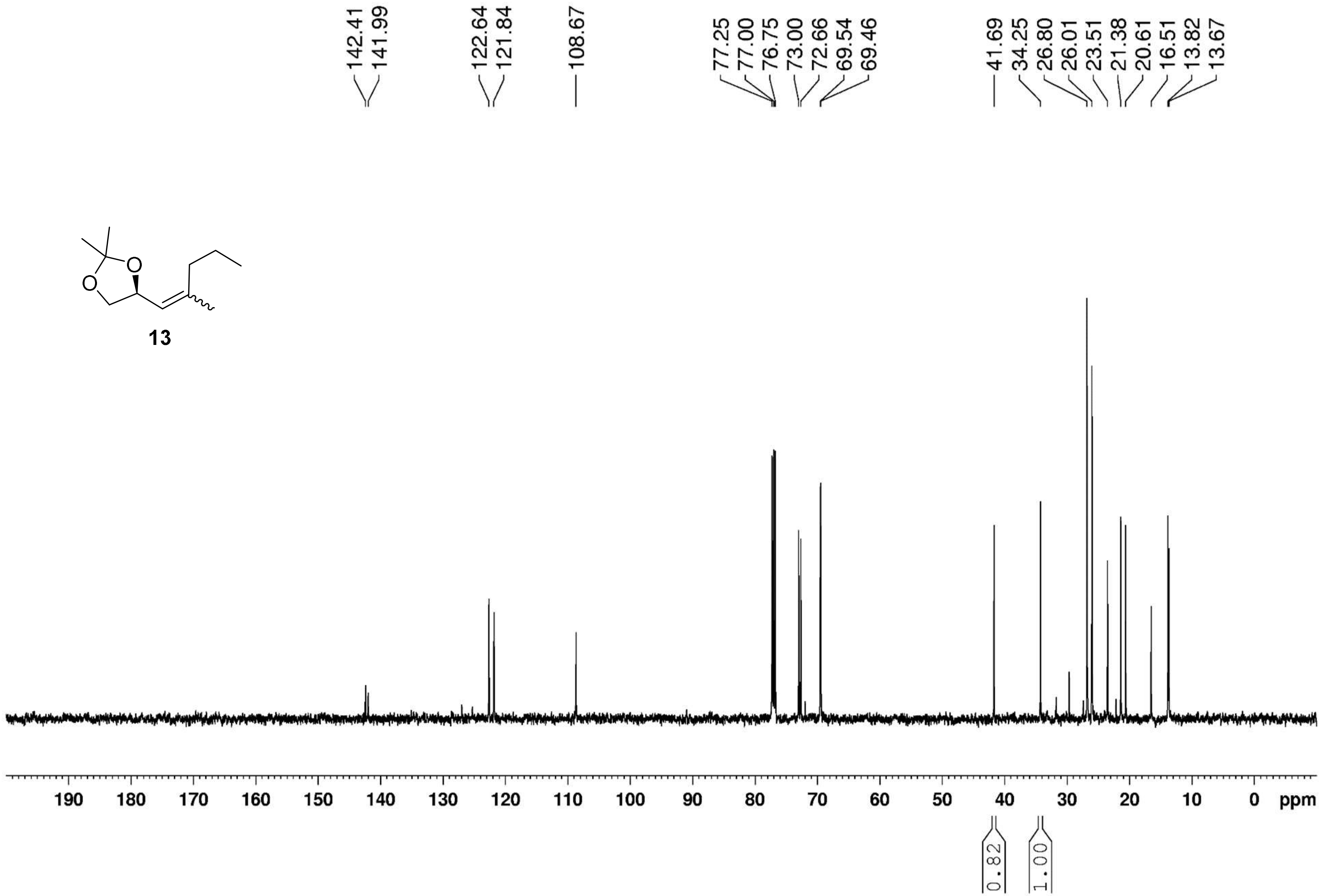

$^{13}C\{^{1}H\}$ NMR of compound **13** (126 MHz, $CDCl_3$)

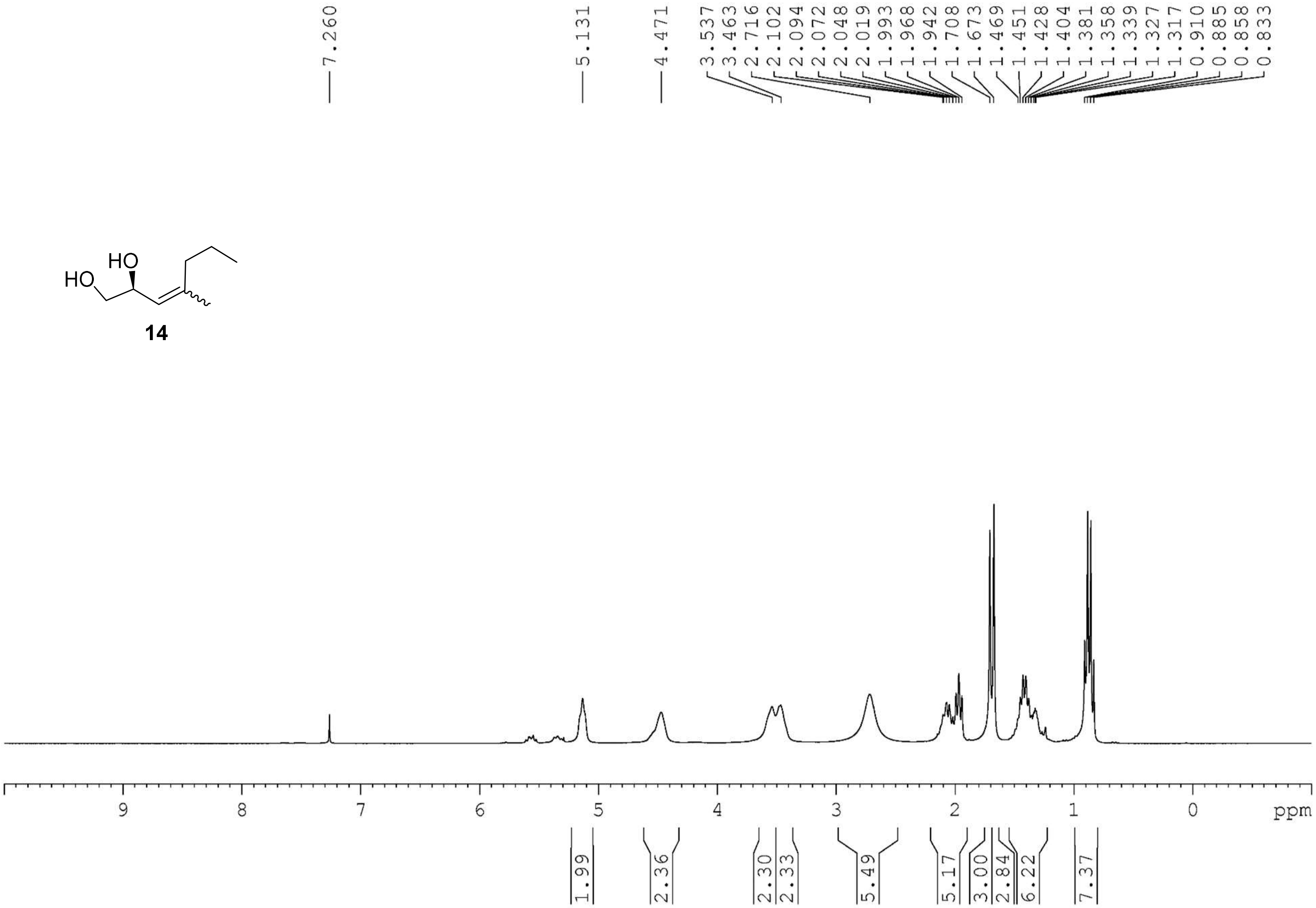

14

^{1}H NMR of compound **14** (300 MHz, $CDCl_3$)

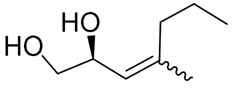

14

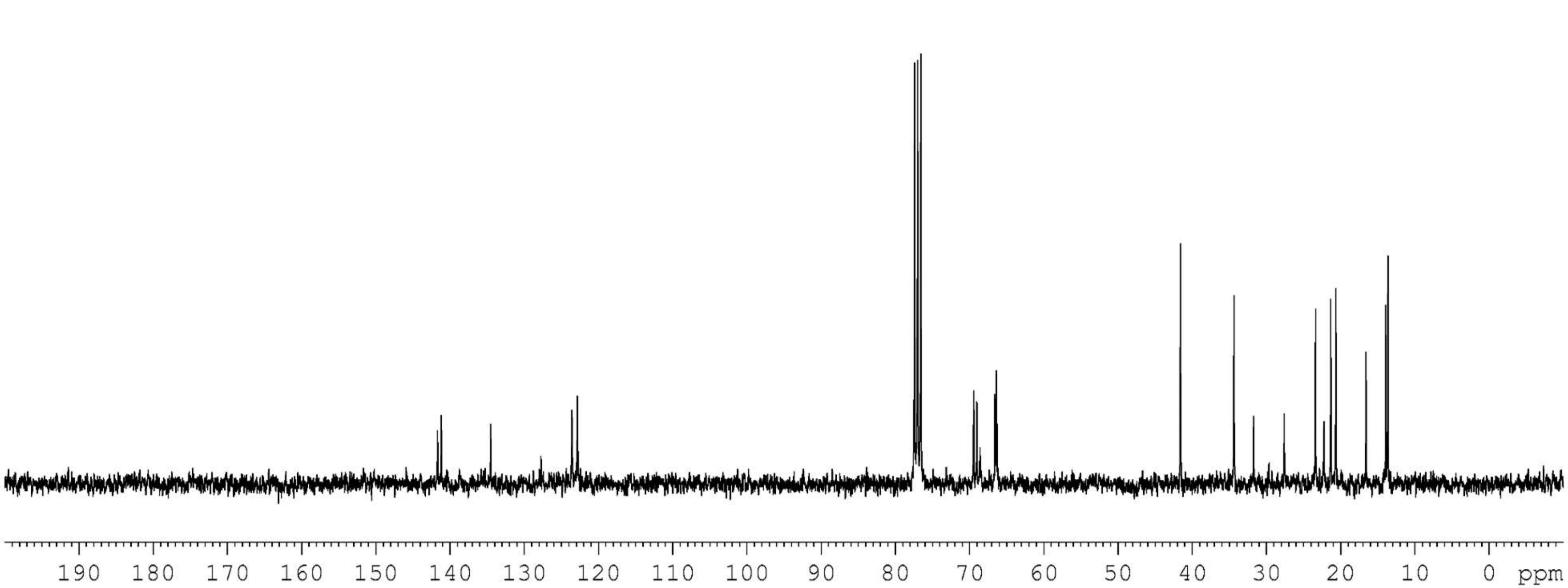

$^{13}C\{^{1}H\}$ NMR of compound **14** (75 MHz, $CDCl_3$)

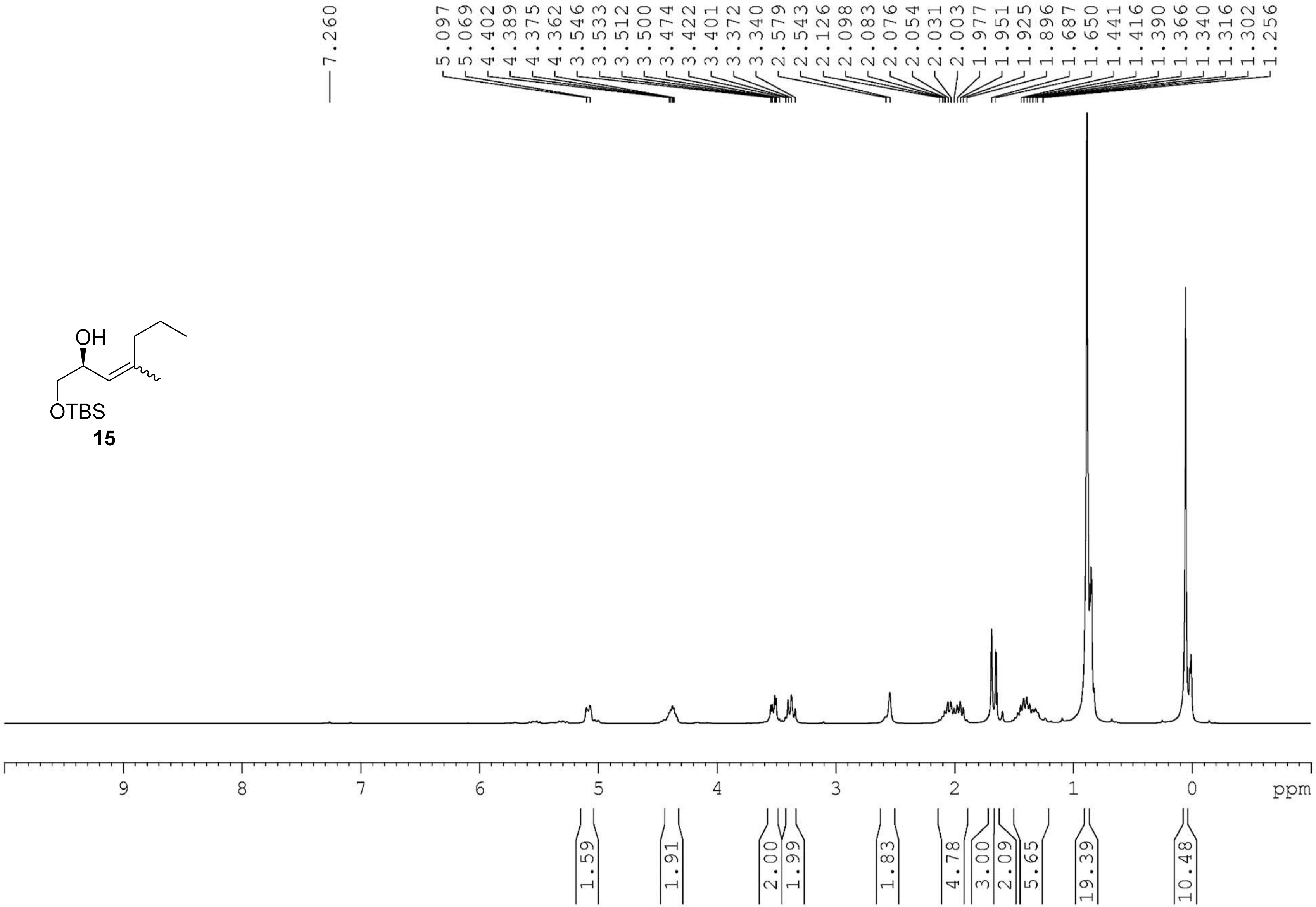
15

^{1}H NMR of compound **15** (300 MHz, $CDCl_3$)

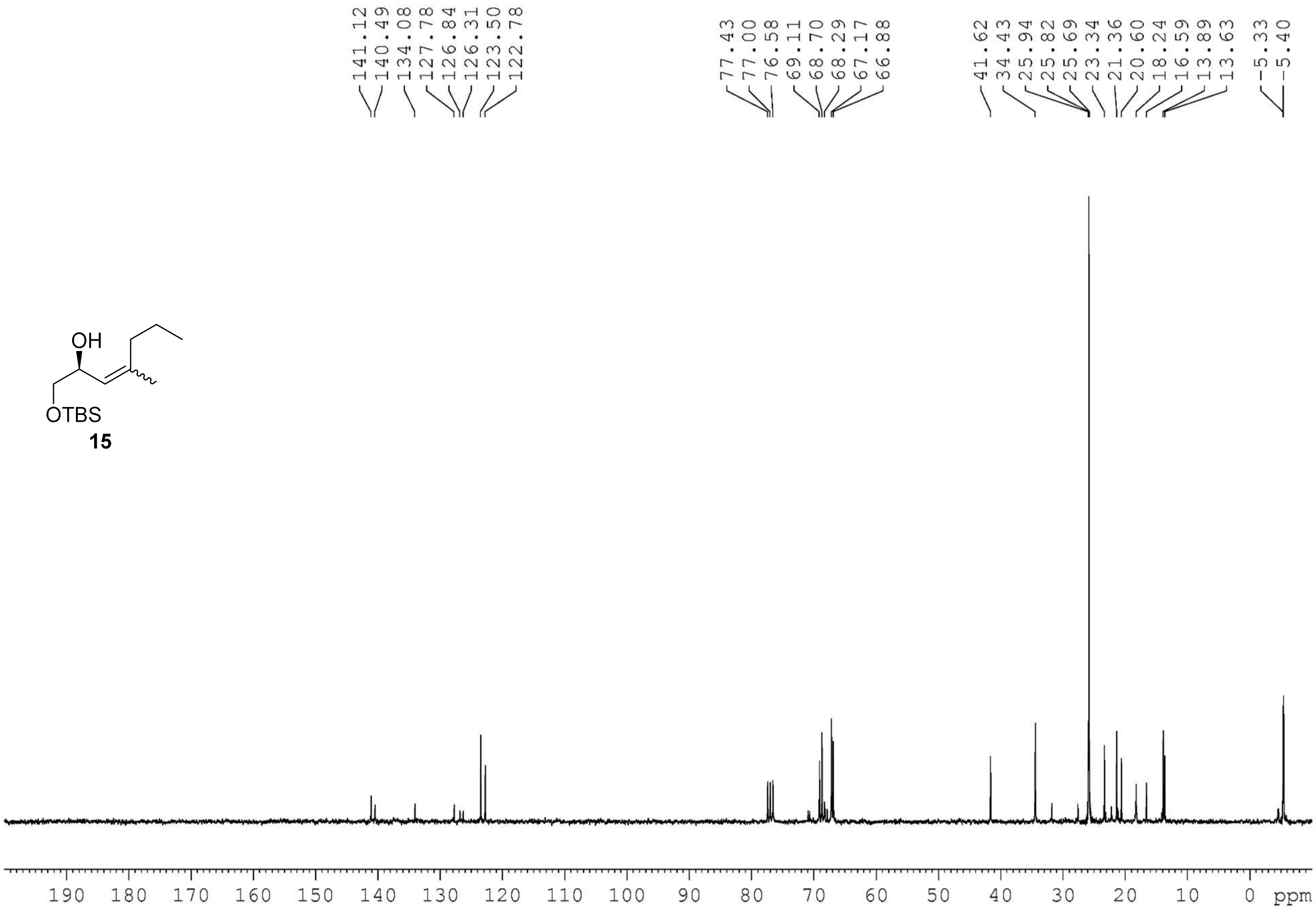

$^{13}C\{^1H\}$ NMR of compound **15** (75 MHz, $CDCl_3$)

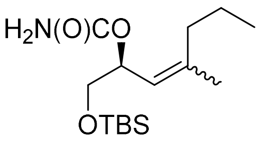

16

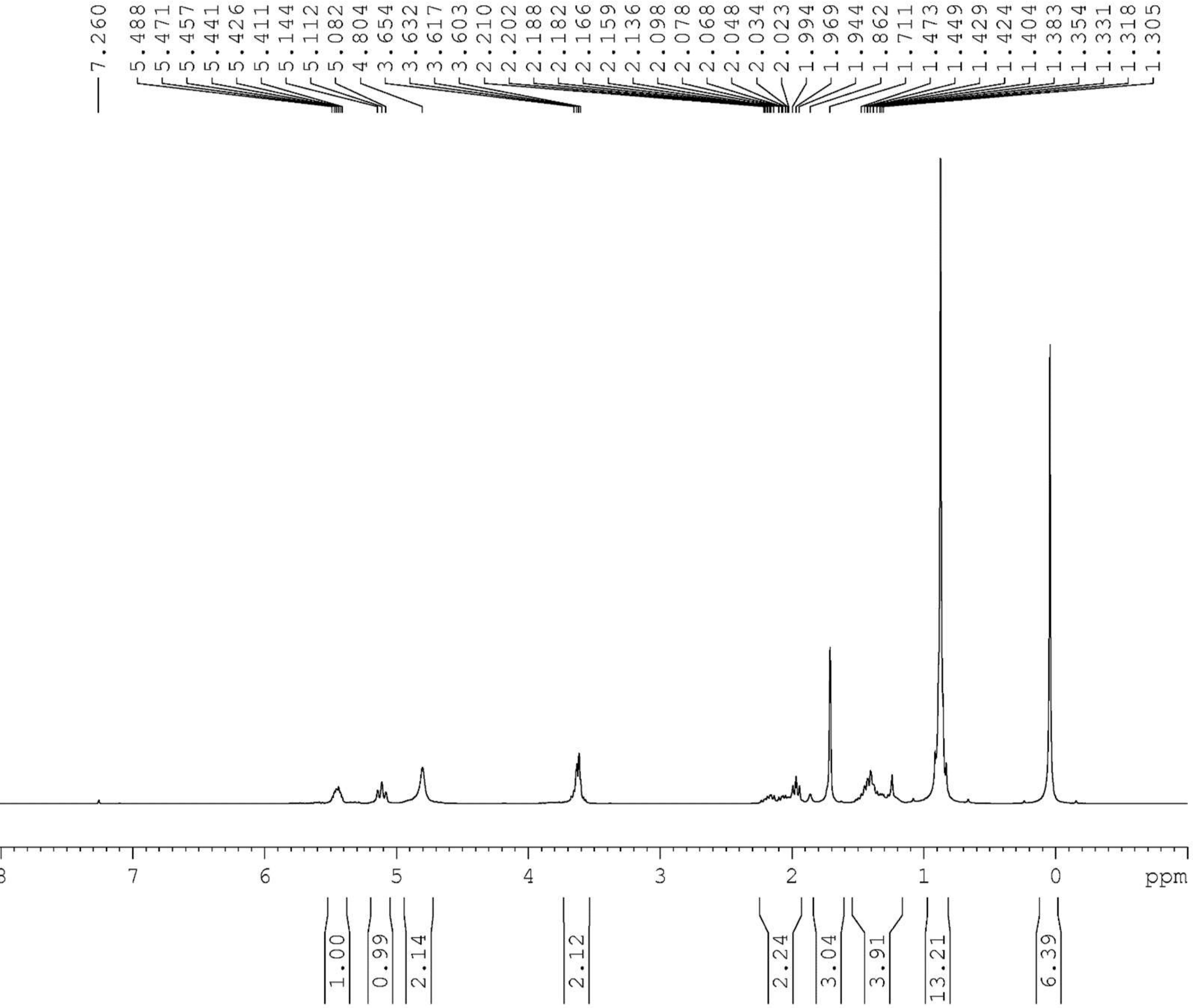

^{1}H NMR of compound **16** (300 MHz, $CDCl_3$)

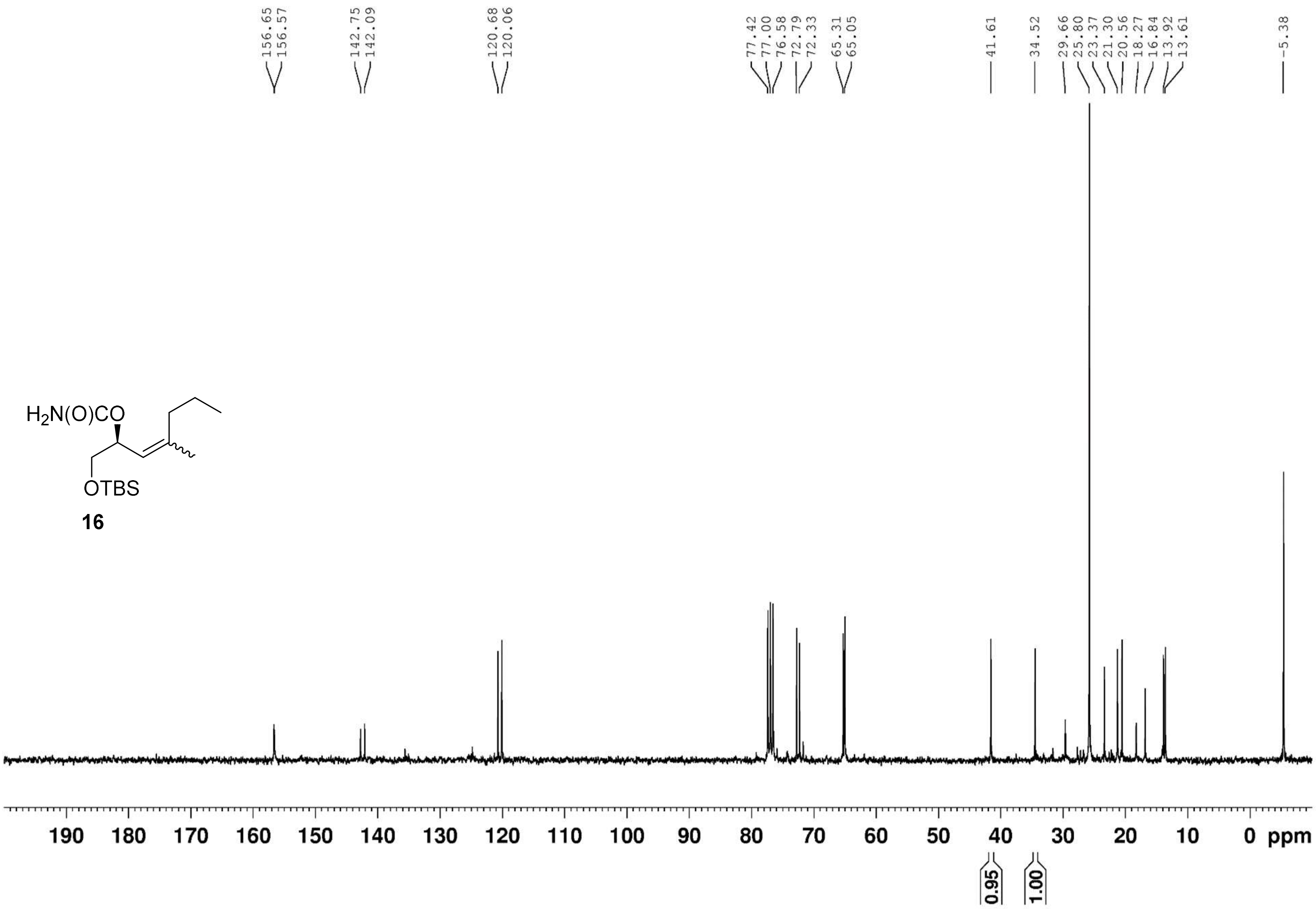

16

$^{13}C\{^1H\}$ NMR of compound **16** (75 MHz, $CDCl_3$)

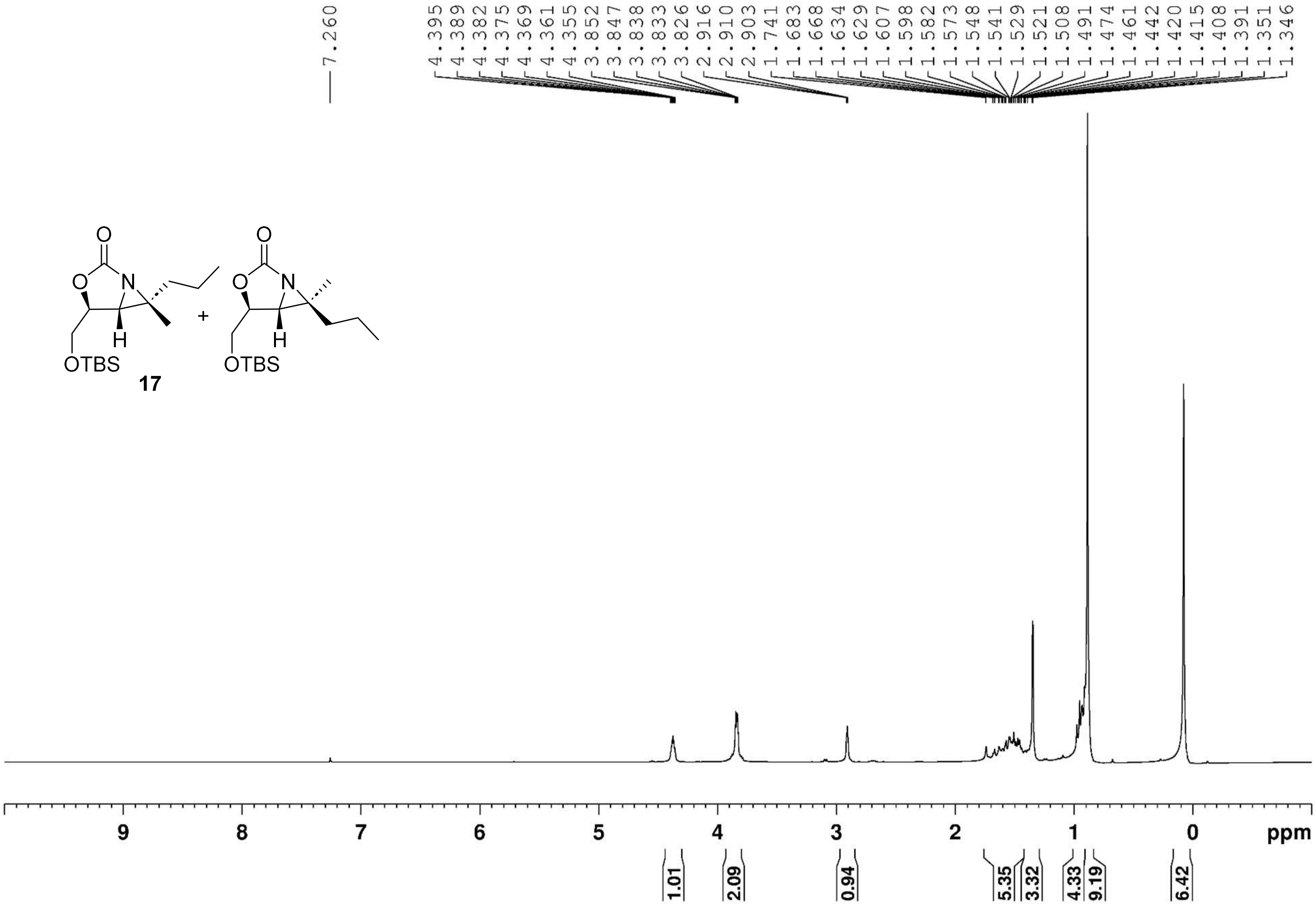

^{1}H NMR of compound **17** (300 MHz, $CDCl_3$)

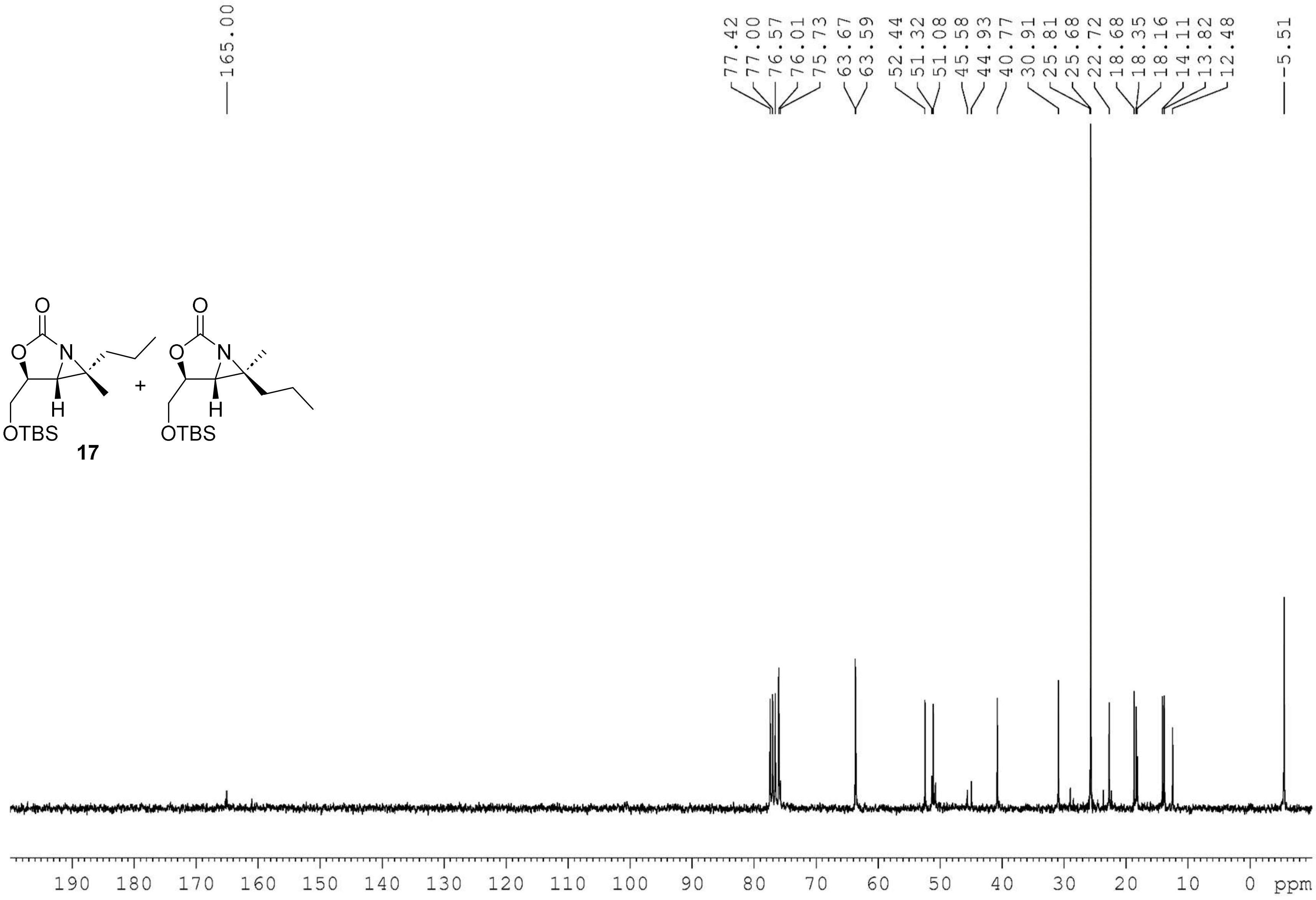

$^{13}C\{^{1}H\}$ NMR of compound **17** (75 MHz, $CDCl_3$)

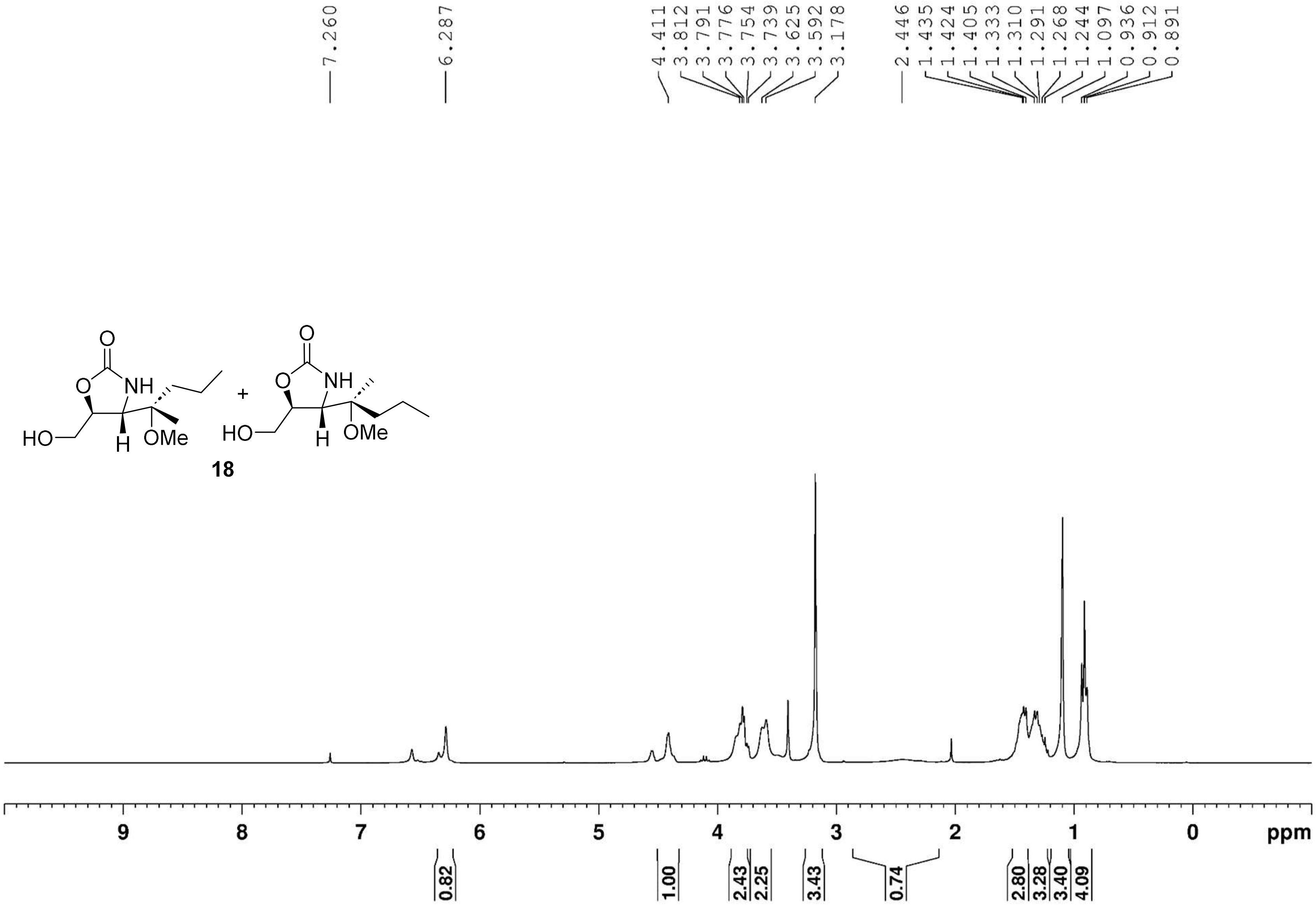

^{1}H NMR of compound **18** (300 MHz, $CDCl_3$)

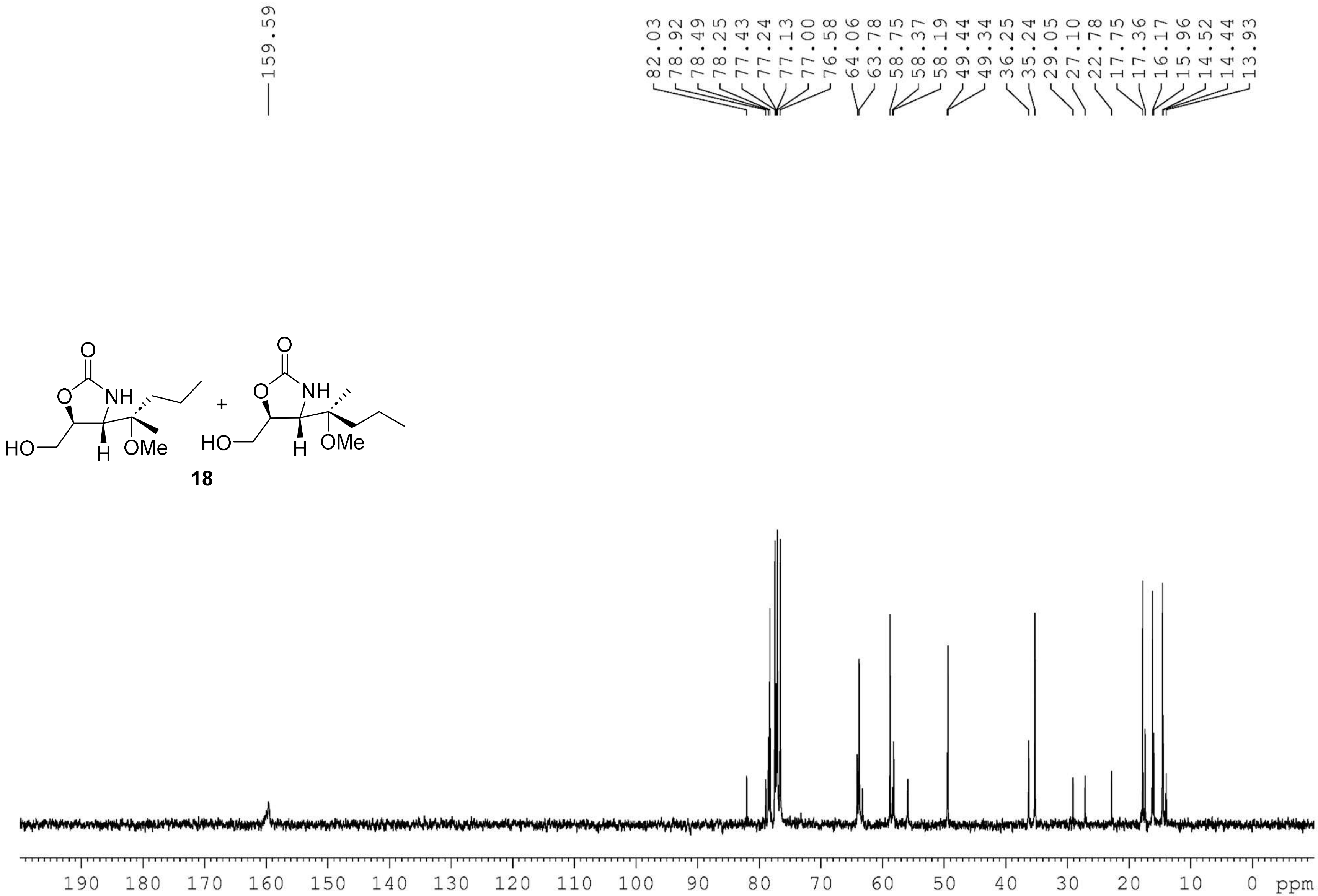

18

$^{13}C\{^{1}H\}$ NMR of compound **18** (75 MHz, $CDCl_3$)

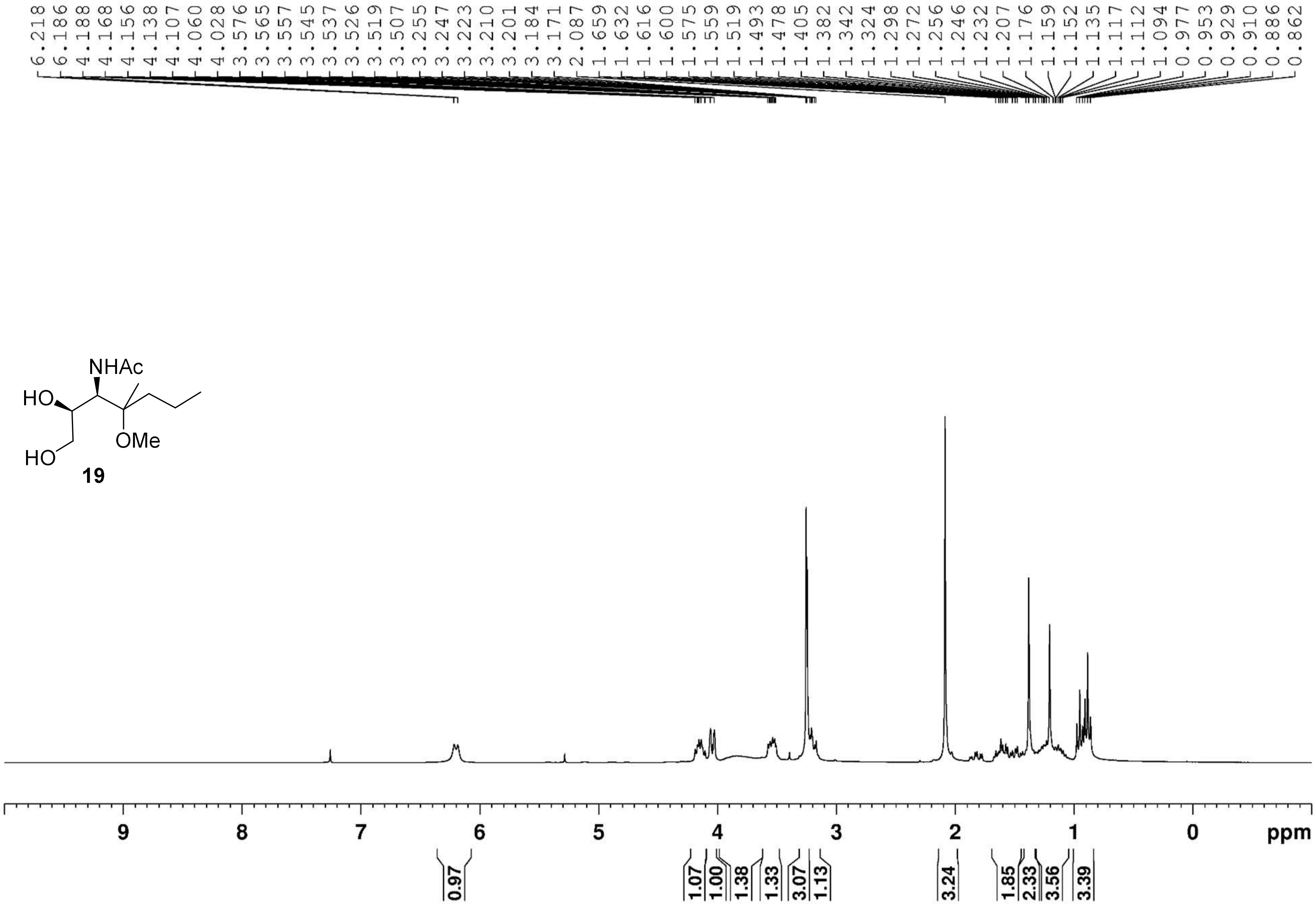

^{1}H NMR of compound **19** (300 MHz, $CDCl_3$)

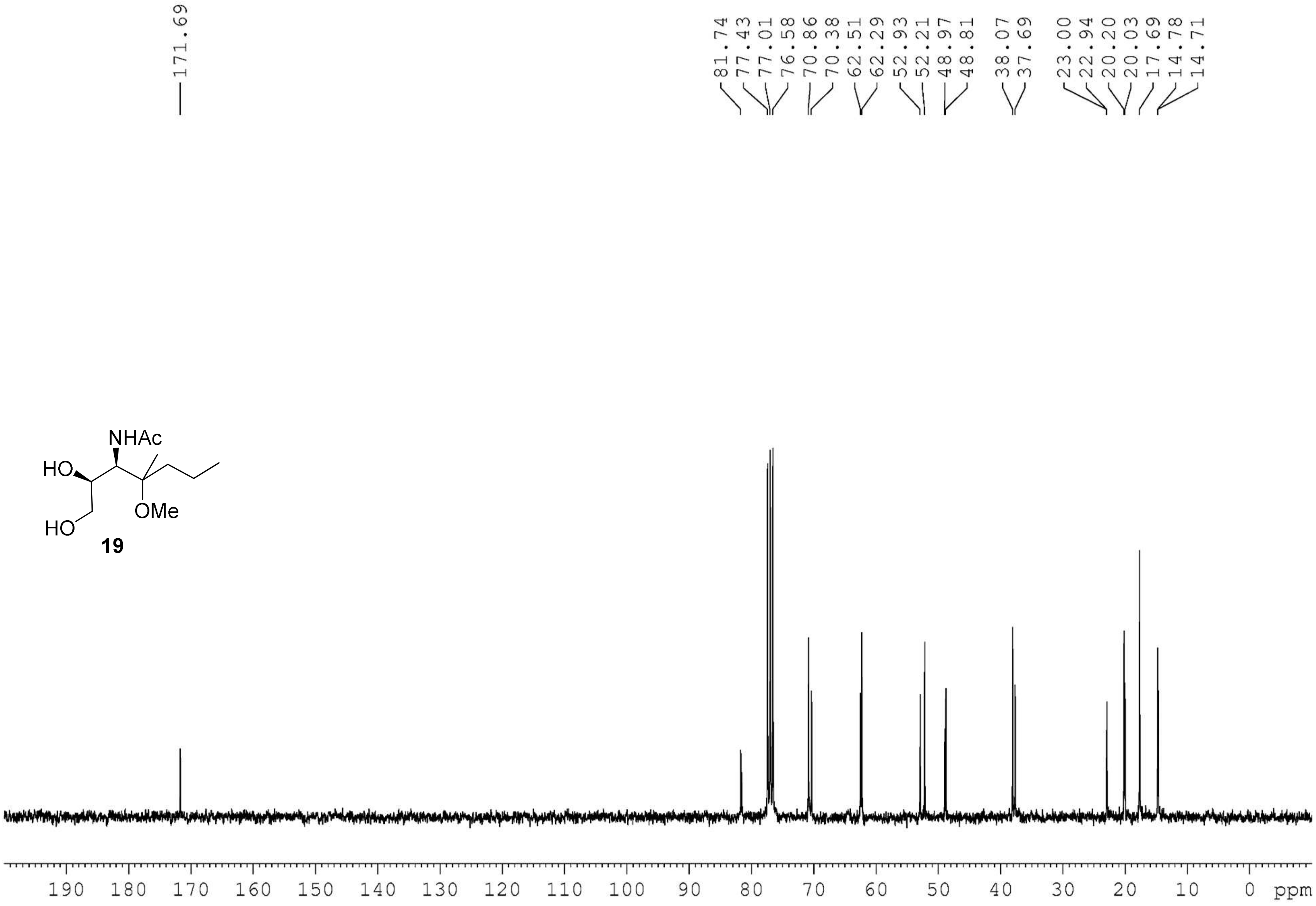

$^{13}C\{^{1}H\}$ NMR of compound **19** (75 MHz, $CDCl_3$)

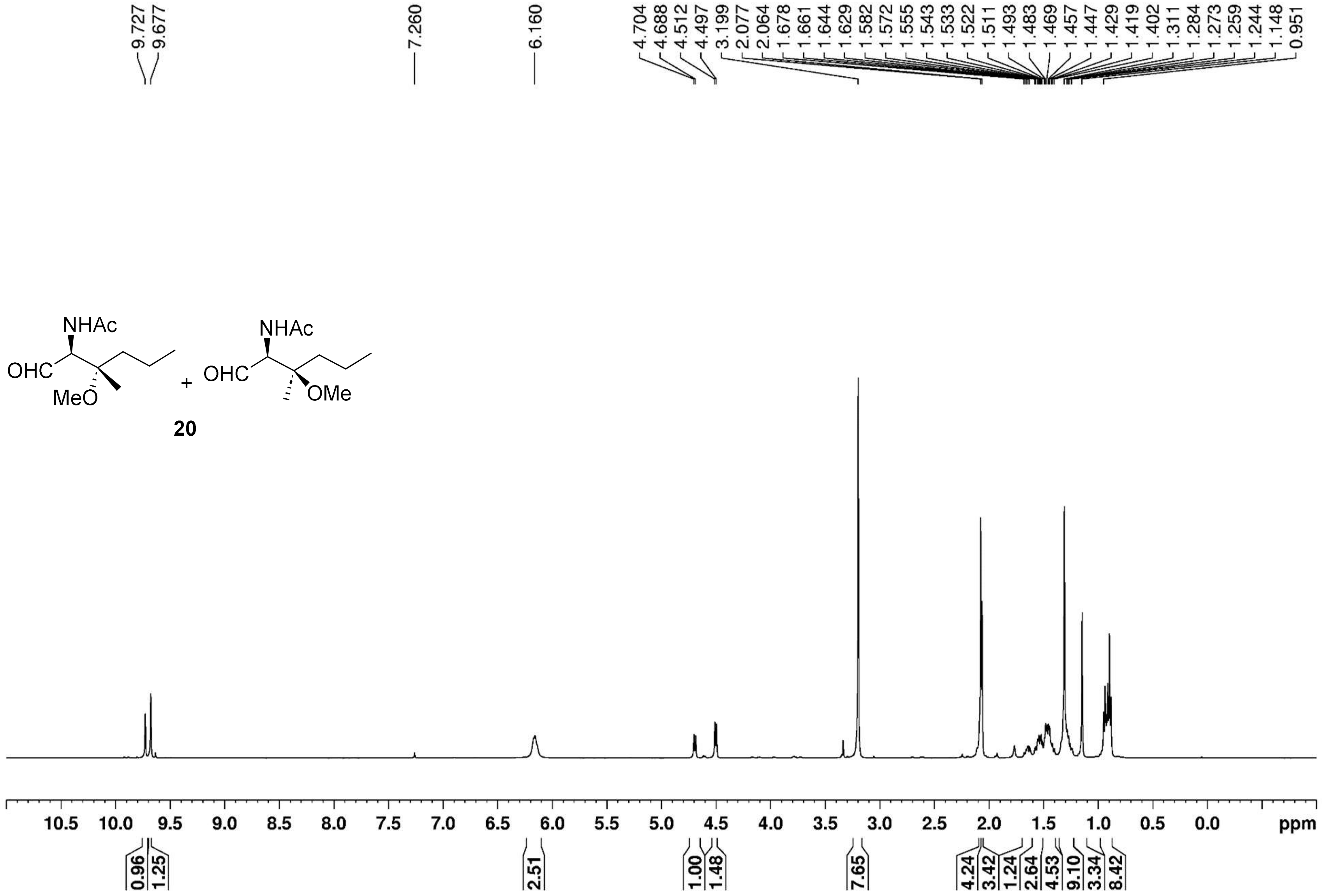

^{1}H NMR of compound **20** (500 MHz, $CDCl_3$)

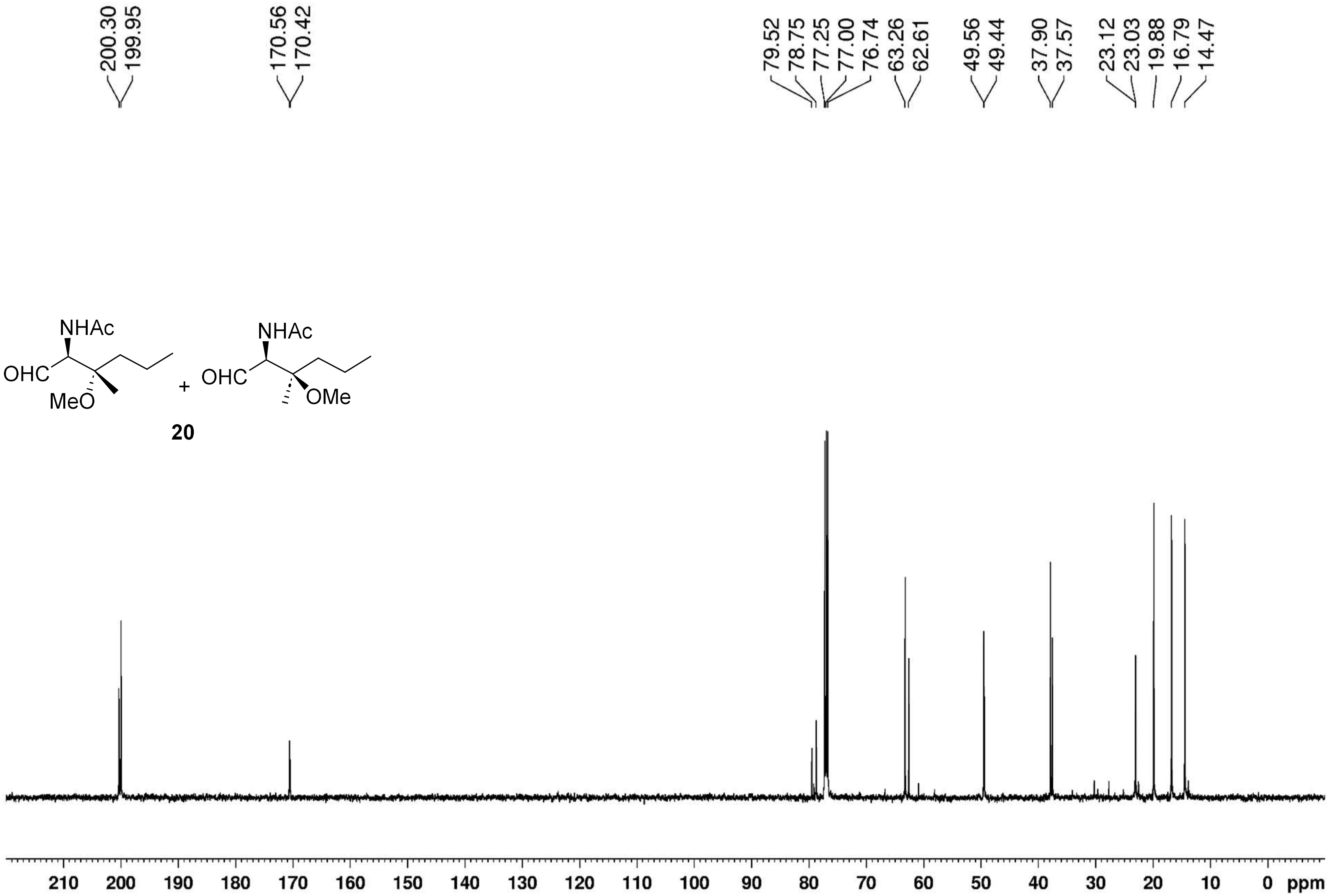

$^{13}C\{^{1}H\}$ NMR of compound **20** (126 MHz, $CDCl_3$)
